# Supplementary material for: Polyclonal selection of immune checkpoint mutations in thyroid autoimmunity
Source: Nature. 2026 Apr 14;654(8117):131–41. doi: 10.1038/s41586-026-10493-9 (PMC13233322; doi:10.1038/s41586-026-10493-9)
Supplement: Supplementary file 1 — This file contains Supplementary Notes 1–3 and additional references. Supplementary Note 1. Macfarlane Burnet's ideas on the role of somatic mutations in autoimmunity. Supplementary Note 2. Further description of the driver landscape in AITD. Supplementary Note 3. Mutational signature analysis in single nuclei. [file 41586_2026_10493_MOESM1_ESM.pdf]

nature portfolio

https://doi.org/10.1038/s41586-026-10493-9

Supplementary information

Polyclonal selection of immune checkpoint mutations in thyroid autoimmunity

In the format provided by the authors and unedited

Supplementary Note 1. Macfarlane Burnet's ideas on the role of somatic mutations in autoimmunity

Macfarlane Burnet is widely known for predicting acquired immune tolerance and for developing the clonal selection theory of acquired immunity. His clonal selection theory also led him to propose that autoimmune diseases may be the result of somatic mutations allowing lymphocytes to escape tolerance mechanisms, which he called the "forbidden clone" hypothesis. He proposed this idea in his 1959 book "*The clonal selection theory of acquired immunity*"[1] and subsequently expanded it and refined it over the next two decades in several articles and books, particularly in a book published in 1972 largely devoted to this hypothesis[2]. Since some of these books are not easily available, this supplementary note contains a few selected quotes from them. While many of Burnet's specific mechanistic proposals were wrong in their details due to the limited knowledge of the immune system at the time, his general insights into the potential role of somatic mutations and somatic evolution in the breakdown of self-tolerance appear prescient.

1959. The clonal selection theory of acquired immunity[1]

While listing five different processes that may lead to a loss of tolerance and autoimmune disease, Burnet listed two possible contributions of somatic mutations. First, somatic recombination and mutation of the B and T cell receptors could lead to the emergence of self-reactive cells; he wrote: "*On the clonal selection hypothesis, random somatic mutation in mesenchymal cells* [lymphocytes] *must continually allow the appearance of forbidden patterns. If, for local or general reasons, the normal elimination of such clones fails to take place, immune disease may result*". Second, he then proposed that somatic mutations could also occur outside of the BCR and TCR, in genes favouring the growth of a clone: "*Like all other body cells, mesenchymal cells may undergo mutation to a form approaching or reaching malignancy*" (pp 123-124).

Later in this book, Burnet predicted the need for peripheral tolerance mechanisms beyond central tolerance as a failsafe mechanism against the accidental production of BCRs and TCRs recognising self-antigens leading to an autoimmune disease: "*a third assumption is especially necessary to give the basic hypothesis any validity- that there must exist a homeostatic mechanism which prevents most of the potentially harmful mutant clones that must be continually arising from becoming dominant enough to produce symptoms*" (p. 134).

He then predicted that the emergence of a self-reactive clone may facilitate the growth of other clones: *"The possibility that the establishment of one clone of such tissue-pathogenic lymphocytes may provide a basis on which other clones with different tissue specificity are more likely to arise, may have some*

bearing on the multiplicity of manifestations which can be observed in some cases of collagen disease" (p. 135).

In his concluding remarks, Burnet wrote: *"I hope, too, that I have been able to show how the type of somatic mutation that must be postulated to account for the physiological aspects of immunity grades smoothly into those concerned with the appearance of auto-immune disease and some of the proliferative diseases of the mesenchymal cell system"* (p. 201).

1972. Auto-immunity and auto-immune disease[2]

In 1972, Burnet published an entire book devoted to discussing the contemporary knowledge on the origin of autoimmune diseases, where he expanded on his views on the possible role of somatic mutations in autoimmunity. Again, his ideas on specific mechanisms have been superseded by advances in our understanding of the cellular and molecular basis of immunity, but his conceptual framework about the possible role of somatic mutations and somatic evolution in autoimmunity still appears relevant.

Early in the book, Burnet introduced his model for autoimmunity in seven statements (pp. 4-6):

1. *"It is necessary for survival that neither immunocytes nor antibodies should exist in the body which are reactive to more than a minimal degree with any accessible body component.*
2. *Since immune pattern is generated by a random process a mechanism must exist by which any 'self-reactive' cells which may emerge can be eliminated or functionally inhibited. More than one mechanism may be needed to establish and maintain this intrinsic immunological tolerance toward self components.*
3. *There are many genetically determined anomalies in the functioning of the immune system (...). Genetic susceptibility to develop auto-immune disease undoubtedly exists. Its basis has not been elucidated at either the genetic or the molecular level, but it is reasonable to suggest that it functions by shifting the 'point of decision' determining whether an immunocyte reacting with its corresponding determinant will be stimulated to proliferation and functional activity or will be destroyed or functionally inhibited.*
4. *Changes due to somatic mutation or to physiological or pharmacological factors may render a newly differentiated immunocyte (...) insusceptible to elimination or inhibition by the 'censorship' mechanism concerned. As in virtually every statement one can make in an immunological context, the word 'insusceptible' is not an absolute but simply refers to a range of susceptibility below the level (...) of the normal newly differentiated immunocyte.*

5. *When, by somatic mutation, an immunocyte line develops which (a) has an undue resistance to elimination by antigenic contact and (b) reacts specifically with an accessible body component functioning as antigenic determinant, it is potentially capable of initiating a forbidden clone of directly or indirectly pathogenic cells. As such they will have all the essential characteristics of a conditioned malignancy.*
6. *In all probability the clinical manifestations of auto-immune disease are due (a) to aggressive T-immunocytes (...) producing damage to target cells, (b) to the deposition of antigen-antibody complexes in the kidney or elsewhere, and (c) to a directly damaging action of antibody (...).*
7. *The accessibility, amount, and physical character of the (auto-)antigen plays an essential part in determining both to what extent a potentially pathogenic clone is stimulated to proliferate and what opportunity it has to damage cells and tissues in the body."*

Later in the book, Burnet explained how the emergence of one self-reactive clone could initiate a cascade recruiting other clones, leading to epitope spreading and an oligoclonal or polyclonal autoimmune response: "*Another implication of this model is that, once a cytotoxic process is initiated, any other potential auto-antigens present in the cells will become accessible to any appropriate immunocytes of either T or B systems. It is very common, for instance, to find at least two types of thyroid auto-antibodies in thyrotoxicosis or Hashimoto's disease*" (p. 185).

Towards the end of the book, Burnet summarised his views as follows: "*After nearly fifteen years' almost uninterrupted thought about the nature of auto-immune disease I am still convinced that it must be looked at as basically the result of genetic anomaly in lymphoid cells of the immune system. The genetic anomalies may be of germinal origin in part, but always include one or more of somatic mutational origin.*". Burnet then included an acknowledgement of the role of environmental influences: "*Just as in the closely analogous field of malignant disease, the basic genetic and somatic genetic processes take place in organisms subject to infection and other impacts of the environment and their results are liable to be modified correspondingly.*" (p. 189).

Quotes from both books are reproduced with permission from Vanderbilt University Press (1959 book) and Springer Nature (1972 book).

Supplementary Note 2. Further description of the driver landscape in AITD

Here we discuss many of the putative drivers in detail, exploring their functions, disease associations, and specific aspects of the selection landscape. Several additional genes which did not reach the pre-defined significance threshold are included due to noteworthy features in their mutational profiles.

TNFRSF14 (*HVEM*)

Start losses affecting *TNFRSF14* were the most frequent coding single nucleotide variants (SNVs) in our dataset (n = 19, *codondnds* q-value = 1.9×10^{-4}). They were detected in 5 donors (H1, H2, H3, H6 and H8) and in H1 alone we observed 8 of the 9 possible codon substitutions. In addition to SNVs, start losses also occurred via deletions that fully excised the start codon (n = 4), a double base substitution (n = 1) and a frameshifting deletion within the start codon (n = 1). An example of a complex deletion spanning the *TNFRSF14* start codon observed in the single nucleus sequencing data is shown in **Extended Data Fig. 10e**. This high recurrence of *TNFRSF14* start loss mutations has previously been observed across leukaemia and lymphoma samples in the COSMIC database[3] (**Extended Data Fig. 3a**) and in thyroid MALT lymphoma specifically[4].

The expected consequence of a start loss mutation is that the 40S ribosomal subunit will continue scanning past the mutated start codon and attempt translation initiation at the next ATG it encounters. For *TNFRSF14*, the next available start codon occurs at the 2nd position in asparagine 88, which would produce a frameshift protein. However, the open reading frame (ORF) produced by this alternative start codon is extremely short, with an ochre stop codon encountered 6 bp later (2nd position of leucine 90). Translation reinitiation is known to occur after short ORFs[5], in which case the next start codon the ribosome encounters would be methionine 98, giving rise to an inframe protein with an N-terminal truncation. This putative protein would lack the canonical signal peptide (residues 1-38), cysteine-rich domain 1 (CRD1, residues 42-75) and part of cysteine-rich domain 2 (CRD2, residues 78-119) present in the full-length protein. It is noteworthy that the inhibitory interaction between BTLA and TNFRSF14 is mediated primarily by CRD1 (and to a lesser extent CRD2)[6], whereas the stimulatory interaction with LIGHT occurs mainly via CRD3[7]. However, using SignalP 6.0[8], this truncated protein is not predicted to have a signal peptide (likelihood = 0.0004) and so the cellular consequence of generating this putative truncated protein is unclear.

Enrichment of other truncating mutations within the first exon of *TNFRSF14* provide possible evidence that start loss mutations may have a consequence other than complete protein loss. Nonsense mutations affecting tryptophan 12 were also highly recurrent (n = 9, *codondnds* q-value = 6.5×10^{-3}), as has previously been described in lymphoma datasets (**Extended Data Fig. 3a**). Single nucleus sequencing data indicate that W12* mutations occurred at least three times independently in donor H1, as mutations were present at both the 2nd and 3rd positions in the codon (**Fig. 5a**) and occurred in and out of phase

with a nearby SNP (**Extended Data Fig. 10c,d**). These W12* mutations (along with other nonsense mutations in the first exon) may give rise to a short enough ORF to permit translation reinitiation. Indeed, 16/43 (37%) of nonsense mutations were observed within the first 30 amino acids (~10%) of the protein. Additionally, the most frequently mutated splice site of *TNFRSF14* was the splice donor site of intron 1, accounting for 17/45 (38%) essential splice site mutations. Although we cannot rule out that this region may have higher mutability, it is also possible that this enrichment of various mutation categories that all could give rise to a truncated protein suggests an alternative selective advantage for N-terminal truncation compared to complete protein loss. Of note, the mutation distribution across the gene body of *TNFRSF14* showed an extremely high degree of mutation enrichment within exons (**Extended Data Fig. 2a**), unlike in targets of somatic hypermutation, such as *LTB* (**Extended Data Fig. 2c**).

We observed additional recurrent mutational hotspots elsewhere in TNFRSF14, including at cysteine residues involved in disulphide bond formation within the cysteine-rich domains (**Fig. 3b**). This enrichment was particularly pronounced within CRD1, in which we also observed a significant excess of missense mutations at non-cysteine residues, suggesting that the selective advantage of *TNFRSF14* mutations may be due to loss of the inhibitory interaction with BTLA. Additional evidence for this hypothesis comes from recurrent mutations affecting glycine 72 (n = 4, *sitednds* *q*-value = 0.019). This residue is within a six-residue binding site in CRD1 that interacts with BTLA. Mutations here have been shown to reduce the binding affinity of TNFRSF14 with BTLA and CD160[9,10].

A small number of synonymous mutations in *TNFRSF14* were surprisingly recurrent or reached unusually high VAFs. However, each of these can be readily explained. A synonymous mutation at tyrosine 26 in donor H1 had a lower bound mutant cell fraction of ~2.2% (**Fig. 1d**). However, this mutation was consistently found to be in phase with a missense mutation affecting the same codon (Y26D) in the NanoSeq, spatial mapping and single nucleus sequencing datasets (**Extended Data Fig. 10a**). Recurrent synonymous mutations observed at leucine 23 (n = 2) and serine 91 (n = 2) are both expected to generate novel donor splice sites (SpliceAI donor gain delta = 0.91 and 0.86 respectively).

CD274 (PD-L1)

Start losses in *CD274* were also highly recurrent, with nine substitutions observed across four donors (H1, H2, H8 and H9) (*codondnds* q-value = 0.044) (**Fig. 3a**). Additionally, a frameshift insertion overlapping the start codon was identified in donor H1. The next available start codon in *CD274* occurs at methionine 10 and so start losses are predicted to result in truncation of the initial 9 residues. As for *TNFRSF14*, this is predicted to disrupt the canonical signal peptide, with the SignalP 6.0[8] likelihood decreasing from 0.9998 for the wild-type protein to 0.4997 for the putative truncated protein.

In the full gene mutation distribution plot for *CD274* (**Extended Data Fig. 2b**), we observed recurrent mutations upstream of the start codon. These mutations are expected to disrupt the splice acceptor site in intron 1. The loss of the splice acceptor site would be expected to lead to exon skipping and may result in N-terminal truncation. These mutations were not annotated as essential splice site mutations by *dNdScv* due to them occurring outside of the ORF.

We observed an excess of mutations in the disulphide bridge cysteines of PD-L1[11] (observed = 11, expected = 0.29, *withingenednds* q-value = 1.1×10^{-6}). By contrast, we observed no significant enrichment of mutations in free cysteines (observed = 1, expected = 0.09, *withingenednds* q-value = 0.53). Of note, within the single nucleus sequencing data, we observed multiple structural variants resulting in loss of *CD274*. As these are not detectable by NanoSeq, it is possible that our inferred mutant cell fractions may be underestimates.

KLHL6

KLHL6 (Kelch-like family member 6) is highly expressed in germinal centre B cells and is key to B cell maturation via signalling through the cell surface BCR[12]. Although *KLHL6* is a known target of activation-induced cytosine deaminase (AID) related somatic hypermutation (SHM), the distribution of mutations in exon 1 exhibited a greater excess of missense mutations than is observed for other targets of SHM (**Extended Data Fig. 2c,d**). Additionally, these missense mutations were specifically enriched within the bounds of the BTB domain (**Extended Data Fig. 3d**). Missense mutation hotspots were observed at codons 65, 90 and 96 of the BTB domain and codons 547 and 568 of the Kelch domain, the latter of which is in a region unaffected by SHM. These mutations have previously been shown to abrogate KLHL6 ubiquitin ligase activity, resulting in increased NF-κB activity via reduced degradation of Roquin2, a negative regulator of TNFAIP3[13]. Of note, we did not observe frequent recurrence of the Kelch domain hotspots in the sorted populations from normal peripheral blood [Lawson et al, *In preparation*]. Additionally, *TNFRSF14* and *TET2* driver mutations have specifically been associated with *KLHL6*-mutant DLBCLs harbouring Kelch domain mutations, rather than those with BTB domain mutations[14].

MAPK and PI3K signalling pathways

Gain of function mutations affecting codons 12 (G12S/V/D/N) and 13 (G13D) in *KRAS* were observed in two donors (H2 and H8). These mutations occur in the phosphate binding loop and reduce the intrinsic GTPase activity of KRAS, leading to a constitutively active KRAS-GTP[15]. Two mutations in codon 59 (A59G and an in-frame deletion), and one in codon 146, detected across three donors (H1, H8 and H9), likely impair GTP hydrolysis leading to constitutive activation[16]. Although not reaching significance in this cohort, four donors (H1, H2, H3 and H6) had activating mutations in *NRAS* at codons 12 (n = 1), 13 (n = 2), and 146 (n = 1).

Further activation of the MAPK pathway was evident via *BRAF* gain of function mutations. One donor (H5) demonstrated a canonical V600E substitution, which is known to cause constitutive activation of BRAF and MAPK-mediated cell proliferation[17] and is a target of precision therapeutics in a number of cancers[18,19]. Additional non-V600E gain of function *BRAF* variants were present across 2 donors (H1 and H8) at codons 464 (n = 1), 466 (n = 2), 469 (n = 1), 581 (n = 1), 594 (n = 2), 595 (n = 1), 597 (n = 2) and 601 (n = 1). Mutations in *BRAF* and the *RAS* genes have been shown to be present in three quarters of papillary thyroid carcinomas[20]. From our bulk NanoSeq data, we cannot determine whether a given mutation is present in thyrocytes, lymphocytes, or other cell types such as macrophages. The MAPK mutations detected in donors H5 and H6 are perhaps more likely to have occurred in thyrocytes given the low degree of lymphocytic infiltration in these biopsies. However, our single nucleus sequencing data confirmed that BRAF F595L and D594N mutations were present in three unrelated class-switched, mature B cells (nuclei 2, 31 and 43) from donor H1 (**Fig. 5a, Extended Data Fig. 7**).

The Src tyrosine phosphatases SHP-1 (PTPN6) and SHP-2 (PTPN11) are expressed throughout the haematopoietic compartment. These phosphatases are recruited to various transmembrane receptors including receptor tyrosine kinases[21] and the phosphotyrosine-rich ITAM and ITIM domains of immune checkpoint proteins, such as PD-1 and BTLA[22,23]. In *PTPN6*, we observed five mutations at codon 419. Substitutions at this residue in SHP-1 produce a phosphatase-inactive form that competes with wildtype SHP-1 for phosphorylated tyrosine substrates[24]. Loss of PTPN6 has been implicated in lymphomas such as DLBCL[25].

PTPN11 codes for SHP-2, a tyrosine phosphatase that acts on RAS isoforms and Src family kinases. In *PTPN11*, a gain of function mutation (E76K) was detected. The variant disinhibits SHP2 and promotes increased rates of catalysis, leading to upregulation of MAPK signalling and ERK activation. These substitutions are common in juvenile myelomonocytic leukaemia[26,27]. A further activating *PTPN11* mutation, associated with Noonan syndrome[28], was observed in the microdissected lymphocytes of one donor (N308D). This substitution stabilises PTPN11 in the active conformation leading to constitutive activity.

RASA2 limits T cell inactivation by inhibiting RAS signalling downstream of the T cell receptor[29]. We observed loss of function mutations distributed throughout *RASA2*. Missense mutations were also enriched in GRB2, an adaptor protein that links receptor activation to RAS signalling via stimulation of guanine nucleotide exchange factors[30]. Mutations in *RASA2* were observed exclusively in B cells in single-nucleus sequencing data for donors H1 and H8 (**Extended Data Fig. 7 and 8**). PTEN, a phosphatase that antagonises PI3K signalling, exhibited loss-of-function mutations throughout the gene, rather than the exon 7 clustered microdeletions observed in T cell acute lymphoblastic leukaemia[31].

RLTPR

RLTPR (RGD Motif, Leucine Rich Repeats, Tropomodulin Domain And Proline-Rich Containing), also known as CARMIL2 (capping protein Arp2/3 complex myosin-I linker), is a scaffold protein that brings together the stimulated cell surface CD28 receptor with the cytoplasmic CARD11[32]. This promotes NF-κB activation in T cells and subsequent differentiation into T cell subtypes[33]. A missense mutation in *RLTPR* was significantly recurrent across three donors (Q575E, *sitednds* *q*-value = 0.008). Variants at this site have been shown to be gain of function in adult T cell leukaemia/lymphoma[34] through increasing binding with CARD11, leading to T cell receptor activation and NF-*κ*B upregulation[34,35].

CARD11

CARD11 (caspase recruitment domain family member 11) is a cytoplasmic scaffold protein and membrane-associated guanylate kinase, which acts as mediator linking BCR stimulation to downstream NF-κB signalling[36]. Recurrent *CARD11* missense mutations at codon 123 (n = 4) were observed in two donors (H1 and H2). These appear to cause gain of function and are observed in lymphomas[37,38]. In the germline, the same gain-of-function variants manifest as polyclonal B cell lymphocytosis with constitutive NF-κB activity[39].

RRAGC

RRAGC is a small GTPase that regulates mTORC1 signalling. Mutations in the GDP/GTP nucleotide binding domain can alter nucleotide binding and upregulate mTOR signalling[40,41]. Gain of function mutations in this region have been recorded in 9 to 17% of follicular lymphomas[41,42]. Across three donors (H1, H2 and G5), we observed gain of function missense mutations at residues within the nucleotide binding domain including codons 90 (n = 1), 114 (n = 1), 115 (n = 1), 116 (n = 4), 118 (n = 1) and 119 (n = 1). Notably, two *RRAGC* mutations (I114R and W115R) were detected in the single nucleus sequencing data from donor H1. Both of these mutations were heterozygous and occurred in mature B cells (nuclei 44 and 45) that did not possess *TNFRSF14* mutations (**Fig. 5a**, **Extended Data Fig. 7**).

CXCR3

CXCR3 (C-X-C motif chemokine receptor 3) is a heterotrimeric transmembrane G-protein coupled receptor responsible for transducing extracellular chemokine signals from three ligands (CXCL9, CXCL10, CXCL11). All four unique nonsense mutations in *CXCR3*, observed in three donors, clustered within a 50 bp region at the terminus of the final exon. As *CXCR3* has only one coding exon, premature stop codons would be expected to escape nonsense-mediated mRNA decay thereby truncating some, or all, of the serine and threonine phosphorylation sites involved in beta-arrestin 2 recruitment and receptor

internalization[43], similar to the consequence of C-terminal truncation mutations in *CCR6*. This is expected to augment CXCR3 signalling and resembles observations in MALT lymphoma of the thyroid[4].

MYD88

MYD88 (myeloid differentiation primary response 88) is an intracellular mediator of Toll-like receptor (TLR) and interleukin-1 (IL-1) signalling via NF-κB activation[44]. A gain of function hotspot (L273P) promotes RNF138-mediated polyubiquitination of MYD88, thereby increasing IRAK1 and IRAK4 interactions with MYD88 and upregulating NF-κB signalling[44,45]. This hotspot is implicated in antigen-activated primary B cells and B cell malignancies such as Waldenstrom macroglobulinaemia and the activated B cell subtype of DLBCL[44,46]. We observed this mutation in one donor (H2).

Additional genes

IRF1 is a transcription factor that regulates interferon-responsive genes[47]. LYN and MAP4K1 are protein kinases that modulate signalling and activation thresholds in B cells, preventing excessive or autoreactive responses[48,49]. LYN has recently been shown to play a key role in establishing anergy in autoreactive B cells[50]. SH2B3 is an adaptor protein that negatively regulates various tyrosine kinases and cytokine signalling pathways, and variants in this gene have been associated with multiple autoimmune diseases[51,52]. PTPRC (CD45) is a receptor tyrosine phosphatase expressed on all nucleated hematopoietic cells, essential for antigen receptor signalling in T and B cells[53].

Evidence of positive selection of some driver genes in control non-autoimmune datasets

Reassuringly, in stark contrast to AITD, *TNFRSF14* and *CD274* mutations were very rare and not under significant positive selection in any of the control datasets (q-value > 0.10), appearing highly specific of AITD lymphocytes (**Fig. 3c**,**d, Extended Data Fig. 4**). Interestingly, however, some of the less frequent driver genes in AITD showed signals of positive selection in B or T memory cells during ageing, revealing that these mutations drive clonal expansions of mutant lymphocytes during normal ageing. As expected, *TET2* and *DNMT3A* mutations cause clonal haematopoiesis and so exhibit signals of positive selection across naive and memory B and T cells. *TNFAIP3*, *LTB*, *DUSP2*, *KLHL6*, *SBF1*, *GRB2*, *NFKBIA*, *TNIP1*, and *MAP4K1* are also under selection in peripheral blood B memory cells during normal ageing (**Fig. 3d**). *PTPRC*, *EEF1A1*, *ARID2* and *SH2B3* mutations are instead selected for in T memory cells (**Extended Data Fig. 4**), which provides suggestive information on the likely cell types that carry these driver mutations in AITD samples. The estimated fraction of lymphocytes with mutations in some of these genes appears to be on the same order of magnitude in this AITD cohort and in peripheral blood from non-autoimmune elderly donors. A direct comparison is complicated as we cannot sort individual cell types from snap-frozen thyroid samples, but this observation suggests

that some of the driver genes above are not enriched in disease, although this does not rule out a possible pathogenic role in the context of an autoimmune response.

Additional pairwise comparisons of the other five control datasets against AITD are shown in **Extended Data Fig. 4**, providing further assurance that the signal of selection in *TNFRSF14* and *CD274* mutations is highly specific of the autoimmune samples in this study. Of note, the enrichment of mutations in the thyroglobulin gene (*TG*) is particularly striking in the control (non-autoimmune) thyroid samples, consistent with these mutations occurring in thyrocytes due to a process of transcription-associated localised indel hypermutation described before for other lineage defining genes, as described in the main text.

Supplementary Note 3. Mutational signature analysis in single nuclei

Mutational signatures are distinct patterns of somatic mutations introduced by endogenous and exogenous mutagenic processes[54]. Studying individual nuclei, we used two different methods to de novo extract mutational signatures and fitted these onto branches of the phylogenetic tree of donor H1. This enables the timing of mutational signatures and somatic driver mutations. To prevent overfitting of signatures, only branches with >50 SNVs were used.

Hierarchical Dirichlet Process

The first method of mutational signature extraction used was hdp (v0.1.5, https://github.com/nicolaroberts/hdp), based on the Bayesian hierarchical Dirichlet process (HDP)[55]. First, de novo mutational signature extraction was performed on branches of the phylogenetic tree without a hierarchy. Each branch was treated as a separate sample to prevent shared mutations between nuclei being double counted. Twenty independent Markov chain Monte Carlo (MCMC) models were run with the following parameters: *burnin = 10,000*, *n = 100*, *space = 200*. Consensus components across all 20 chains were extracted and resulted in 7 distinct components.

Components 1 to 4 accounted for 86% of mutations altogether. Component 5 accounted for 10%. As components 6 and 7 accounted for a small proportion of mutations (2.3% and 0.74% respectively), these were excluded from further analysis to reduce overfitting. Cosine similarities were calculated between extracted signatures and a reference set derived from the COSMIC v3.5 database[56] (https://cancer.sanger.ac.uk), a published primary template-directed amplification (PTA) artefact signature called scF[57], and two B-cell-specific signatures previously identified in expanded single-cell lymphocyte cultures[58].

Component 5 had the highest cosine similarity to SBS95 (cosine similarity = 0.74). The mutational spectrum of SBS95 involves a significant C>A component, primarily in genic regions. Prior work has previously identified SBS95 in a single cohort of hepatocellular carcinomas (ICGC LINC-JP project) and without validation cohorts, SBS95 has been attributed to a sequencing artefact[59]. In keeping with component 5 likely being an artefact, this was present across all cell types, did not scale with mutation burden and demonstrated a batch effect between PTA plates (SBS95 median proportion: plate 1 = 0.051, plate 2 = 0.14, P = 1.7e-14, Wilcoxon rank-sum test). As such, component 5 was excluded from downstream analysis.

Based on their high cosine similarities (>0.8), biological plausibility, and visual inspection, the remaining four components were fitted to eight known reference signatures: SBS1, SBS5, SBS9, SBS17a, SBS17b, SBS85, SBSblood[60] and scF[57]. An expectation maximisation algorithm was used to deconvolute the HDP components into combinations of these reference signatures. Two rounds of

expectation maximisation were used. First, all eight reference signatures were fitted. Second, only those reference signatures contributing >10% to each component were fitted.

Non-negative matrix factorisation

An independent second method of mutational signature extraction, based on non-negative matrix factorisation, was undertaken using the SigProfiler suite[61] (v1.1.24, https://cancer.sanger.ac.uk/signatures/tools/). De novo extraction was performed with SigProfilerExtractor (v1.2.6, https://github.com/AlexandrovLab/SigProfilerExtractor) using the default parameters. The optimal number of signatures extracted was four. SigProfilerAssignment (v1.1.3, https://github.com/SigProfilerSuite/SigProfilerAssignment) was used to decompose these four components into reference signatures. All 8 reference signatures identified in the HDP method had a cosine similarity >0.8.

Signature fitting to phylogenetic branches

The 8 reference signatures common to both methods were fitted to branches of the phylogenetic tree using sigfit[62] (v2.2, https://github.com/kgori/sigfit). For each branch with >50 SNVs, all 8 signatures were fitted using a Poisson model (20,000 iterations, 10,000 warmup). Signatures contributing <5% to a branch were dropped, and fitting was re-run with the remaining signatures. For branches with ≤50 SNVs, their exposures were assigned as the average exposures of their child branches (or grandchild branches if no child branches had >50 SNVs).

Mutational signatures associated with drivers and cell types

SBS1 reflects the deamination of 5-methylcytosine and prominently features C>T transitions at CpG sites[63]. This contributed at least 5% of mutations to more than half of all cells, across all cell types (57/86, 66.3%). SBS5 is a ubiquitous clock-like signature observed across healthy and malignant tissues[63–65]. Although the mechanism is unknown, this may be the result of the misrepair of continuous DNA damage. Substantial fractions of SBS5 (>5% mutations) were present across all cell types (**Extended Data Fig. 11a,c**). Similarly, SBSblood[60], a signature thought to be related to the activity of SBS5 in blood, was detectable at significant proportions (>5% mutations) in all sequenced cells (**Extended Data Fig. 11a,c**).

AID-related SHM introduces point mutations not only in immunoglobulin genes, but throughout the genome[66]. Error-prone repair of these mutations by DNA polymerase-η leads to A>C and T>G transversions, characteristic of SBS9[54]. DNA polymerase-η is upregulated in proliferating germinal centre B cells undergoing SHM[67]. In keeping with this, non-naive B cells were enriched for SBS9 (>5% mutations, 58/61, 95.1%), compared to naive B cells, T cells and non-lymphocyte populations (**Extended Data Fig. 11a-c**). Although rare, another mutational signature reflecting AID-related SHM[68]

(SBS85) was detectable only in *TNFRSF14*-mutant non-naive B cells, albeit with far fewer mutations attributed (>1% mutations) compared to SBS9 (**Extended Data Fig. 11a,c**). SBS85 appeared to be present in ancestral cells prior to formation of clades, suggesting this mutational process may be more active in early development (**Extended Data Fig. 11a**).

Much like SBS9, SBS17b, a mutational signature of unknown aetiology and one previously linked to oesophageal adenocarcinoma, consists primarily of G[A>C]A and C[T>G]T transversions[69]. This signature was infrequent, contributed little to the overall mutation count but was exclusively in SBS9-positive *TNFRSF14*-mutant non-naive B cells (**Extended Data Fig. 11c**). In 9 of these cells, SBS17b contributed >5% of mutations. SBS17a, with a predominance of G[A>G]A and C[T>C]T transitions, demonstrated similar associations[69] in just 3 SBS9-positive *TNFRSF14*-mutant non-naive B cells.

In keeping with a known PTA-sequencing artefactual signature[57] (scF), the absolute number of mutations attributed to scF was similar across all cells. As such, scF[57] frequently contributed <5% mutations to the signature profiles of cells with higher mutation burdens, such as non-naive B cells (14/61, 23%). In nearly all naive B cells, T cells and non-lymphocyte populations, scF[57] contributed >5% mutations (23/25, 92%) (**Extended Data Fig. 11a,c**).

References for supplementary material

1. Burnet, M. *The Clonal Selection Theory of Acquired Immunity*. (Vanderbilt university press, Nashville, Tennessee, 1959).
2. Burnet, M. *Auto-Immunity and Auto-Immune Disease: A Survey for Physician or Biologist*. (Medical and Technical Publishing Co. Ltd., Lancaster, 1972).
3. Sondka, Z. *et al.* COSMIC: a curated database of somatic variants and clinical data for cancer. *Nucleic Acids Res.* **52**, D1210–D1217 (2024).
4. Wu, F. *et al.* Thyroid MALT lymphoma: self-harm to gain potential T-cell help. *Leukemia* **35**, 3497–3508 (2021).
5. Sherlock, M. E., Baquero Galvis, L., Vicens, Q., Kieft, J. S. & Jagannathan, S. Principles, mechanisms, and biological implications of translation termination-reinitiation. *RNA* **29**, 865–884 (2023).
6. Compaan, D. M. *et al.* Attenuating lymphocyte activity: the crystal structure of the BTLA-HVEM complex. *J. Biol. Chem.* **280**, 39553–39561 (2005).
7. Sarrias, M. R. *et al.* The three HveA receptor ligands, gD, LT-alpha and LIGHT bind to distinct sites on HveA. *Mol. Immunol.* **37**, 665–673 (2000).
8. Teufel, F. *et al.* SignalP 6.0 predicts all five types of signal peptides using protein language models. *Nat. Biotechnol.* **40**, 1023–1025 (2022).
9. Liu, W. *et al.* HVEM structures and mutants reveal distinct functions of binding to LIGHT and BTLA/CD160. *J. Exp. Med.* **218**, (2021).
10. Shrestha, R., Garrett-Thomson, S. C., Liu, W., Almo, S. C. & Fiser, A. Redesigning HVEM interface for selective binding to LIGHT, BTLA, and CD160. *Structure* **28**, 1197–1205.e2 (2020).
11. Zak, K. M. *et al.* Structure of the complex of human programmed death 1, PD-1, and its ligand PD-L1. *Structure* **23**, 2341–2348 (2015).
12. Bertocci, B. *et al.* Klhl6 deficiency impairs transitional B cell survival and differentiation. *J. Immunol.* **199**, 2408–2420 (2017).
13. Choi, J. *et al.* Loss of KLHL6 promotes diffuse large B-cell lymphoma growth and survival by stabilizing the mRNA decay factor roquin2. *Nat. Cell Biol.* **20**, 586–596 (2018).
14. Meriranta, L. *et al.* Disruption of KLHL6 fuels oncogenic antigen receptor signaling in B-cell lymphoma. *Blood Cancer Discov.* **5**, 331–352 (2024).
15. Hunter, J. C. *et al.* Biochemical and structural analysis of common cancer-associated KRAS mutations. *Mol. Cancer Res.* **13**, 1325–1335 (2015).
16. Hall, B. E., Bar-Sagi, D. & Nassar, N. The structural basis for the transition from Ras-GTP to Ras-GDP. *Proc. Natl. Acad. Sci. U. S. A.* **99**, 12138–12142 (2002).
17. Davies, H. *et al.* Mutations of the BRAF gene in human cancer. *Nature* **417**, 949–954 (2002).
18. Chapman, P. B. *et al.* Improved survival with vemurafenib in melanoma with BRAF V600E mutation. *N. Engl. J. Med.* **364**, 2507–2516 (2011).
19. Hauschild, A. *et al.* Dabrafenib in BRAF-mutated metastatic melanoma: a multicentre, open-label, phase 3 randomised controlled trial. *Lancet* **380**, 358–365 (2012).
20. Cancer Genome Atlas Research Network. Integrated genomic characterization of papillary thyroid carcinoma. *Cell* **159**, 676–690 (2014).
21. Zhang, J., Zhang, F. & Niu, R. Functions of Shp2 in cancer. *J. Cell. Mol. Med.* **19**, 2075–2083 (2015).
22. Xu, X. *et al.* PD-1 and BTLA regulate T cell signaling differentially and only partially through SHP1 and SHP2. *J. Cell Biol.* **219**, (2020).
23. Xu, X., Masubuchi, T., Cai, Q., Zhao, Y. & Hui, E. Molecular features underlying differential

SHP1/SHP2 binding of immune checkpoint receptors. *Elife* **10**, (2021).
24. Xie, Z. H., Zhang, J. & Siraganian, R. P. Positive regulation of c-Jun N-terminal kinase and TNF-alpha production but not histamine release by SHP-1 in RBL-2H3 mast cells. *J. Immunol.* **164**, 1521–1528 (2000).
25. Demosthenous, C., Han, J. J., Hu, G., Stenson, M. & Gupta, M. Loss of function mutations in PTPN6 promote STAT3 deregulation via JAK3 kinase in diffuse large B-cell lymphoma. *Oncotarget* **6**, 44703–44713 (2015).
26. Tartaglia, M. *et al.* Somatic mutations in PTPN11 in juvenile myelomonocytic leukemia, myelodysplastic syndromes and acute myeloid leukemia. *Nat. Genet.* **34**, 148–150 (2003).
27. Gupta, A. K., Meena, J. P., Chopra, A., Tanwar, P. & Seth, R. Juvenile myelomonocytic leukemia-A comprehensive review and recent advances in management. *Am. J. Blood Res.* **11**, 1–21 (2021).
28. Tartaglia, M. *et al.* Mutations in PTPN11, encoding the protein tyrosine phosphatase SHP-2, cause Noonan syndrome. *Nat. Genet.* **29**, 465–468 (2001).
29. Carnevale, J. *et al.* RASA2 ablation in T cells boosts antigen sensitivity and long-term function. *Nature* **609**, 174–182 (2022).
30. Tari, A. M. & Lopez-Berestein, G. GRB2: a pivotal protein in signal transduction. *Semin. Oncol.* **28**, 142–147 (2001).
31. Mendes, R. D. *et al.* PTEN microdeletions in T-cell acute lymphoblastic leukemia are caused by illegitimate RAG-mediated recombination events. *Blood* **124**, 567–578 (2014).
32. Liang, Y. *et al.* The lymphoid lineage-specific actin-uncapping protein Rltpr is essential for costimulation via CD28 and the development of regulatory T cells. *Nat. Immunol.* **14**, 858–866 (2013).
33. Lévy, R. *et al.* Human CARMIL2 deficiency underlies a broader immunological and clinical phenotype than CD28 deficiency. *J. Exp. Med.* **220**, (2023).
34. Uchida, Y. *et al.* RLTPR Q575E: A novel recurrent gain-of-function mutation in patients with adult T-cell leukemia/lymphoma. *Eur. J. Haematol.* **106**, 221–229 (2021).
35. Park, J. *et al.* Genomic analysis of 220 CTCLs identifies a novel recurrent gain-of-function alteration in RLTPR (p.Q575E). *Blood* **130**, 1430–1440 (2017).
36. Bertin, J. *et al.* CARD11 and CARD14 are novel caspase recruitment domain (CARD)/membrane-associated guanylate kinase (MAGUK) family members that interact with BCL10 and activate NF-kappa B. *J. Biol. Chem.* **276**, 11877–11882 (2001).
37. Lacy, S. E. *et al.* Targeted sequencing in DLBCL, molecular subtypes, and outcomes: a Haematological Malignancy Research Network report. *Blood* **135**, 1759–1771 (2020).
38. de Groen, R. A. L. *et al.* Frequent mutated B2M, EZH2, IRF8, and TNFRSF14 in primary bone diffuse large B-cell lymphoma reflect a GCB phenotype. *Blood Adv.* **5**, 3760–3775 (2021).
39. Brohl, A. S. *et al.* Germline CARD11 mutation in a patient with severe congenital B cell lymphocytosis. *J. Clin. Immunol.* **35**, 32–46 (2015).
40. Okosun, J. *et al.* Recurrent mTORC1-activating RRAGC mutations in follicular lymphoma. *Nat. Genet.* **48**, 183–188 (2016).
41. Ying, Z. X. *et al.* Recurrent mutations in the MTOR regulator RRAGC in follicular lymphoma. *Clin. Cancer Res.* **22**, 5383–5393 (2016).
42. Pasqualucci, L. *et al.* Genetics of follicular lymphoma transformation. *Cell Rep.* **6**, 130–140 (2014).
43. Eiger, D. S. *et al.* Phosphorylation barcodes direct biased chemokine signaling at CXCR3. *Cell Chem. Biol.* **30**, 362–382.e8 (2023).
44. Yu, X. *et al.* MYD88 L265P elicits mutation-specific ubiquitination to drive NF-κB activation and lymphomagenesis. *Blood* **137**, 1615–1627 (2021).

45. Minderman, M. *et al.* The oncogenic human B-cell lymphoma MYD88 L265P mutation genocopies activation by phosphorylation at the Toll/interleukin-1 receptor (TIR) domain. *Blood Cancer J.* **13**, 125 (2023).
46. Wang, J. Q., Jeelall, Y. S., Beutler, B., Horikawa, K. & Goodnow, C. C. Consequences of the recurrent MYD88(L265P) somatic mutation for B cell tolerance. *J. Exp. Med.* **211**, 413–426 (2014).
47. Negishi, H. *et al.* Evidence for licensing of IFN-gamma-induced IFN regulatory factor 1 transcription factor by MyD88 in Toll-like receptor-dependent gene induction program. *Proc. Natl. Acad. Sci. U. S. A.* **103**, 15136–15141 (2006).
48. Nishizumi, H., Horikawa, K., Mlinaric-Rascan, I. & Yamamoto, T. A double-edged kinase Lyn: a positive and negative regulator for antigen receptor-mediated signals. *J. Exp. Med.* **187**, 1343–1348 (1998).
49. Wang, X. *et al.* Down-regulation of B cell receptor signaling by hematopoietic progenitor kinase 1 (HPK1)-mediated phosphorylation and ubiquitination of activated B cell linker protein (BLNK). *J. Biol. Chem.* **287**, 11037–11048 (2012).
50. Fiske, B. E., Wemlinger, S. M., Crute, B. W. & Getahun, A. Lyn governs the establishment and maintenance of B cell anergy by suppressing PI3K signaling. *Nat. Commun.* (2026) doi:10.1038/s41467-026-70085-z.
51. Alcina, A. *et al.* The autoimmune disease-associated KIF5A, CD226 and SH2B3 gene variants confer susceptibility for multiple sclerosis. *Genes Immun.* **11**, 439–445 (2010).
52. Zhang, Y. *et al.* Rare SH2B3 coding variants in lupus patients impair B cell tolerance and predispose to autoimmunity. *J. Exp. Med.* **221**, (2024).
53. Hermiston, M. L., Xu, Z. & Weiss, A. CD45: a critical regulator of signaling thresholds in immune cells. *Annu. Rev. Immunol.* **21**, 107–137 (2003).
54. Alexandrov, L. B. *et al.* Signatures of mutational processes in human cancer. *Nature* **500**, 415–421 (2013).
55. Brunner, S. F. *et al.* Somatic mutations and clonal dynamics in healthy and cirrhotic human liver. *Nature* **574**, 538–542 (2019).
56. Tate, J. G. *et al.* COSMIC: The Catalogue Of Somatic Mutations In Cancer. *Nucleic Acids Res.* **47**, D941–D947 (2019).
57. Petljak, M. *et al.* Characterizing mutational signatures in human cancer cell lines reveals episodic APOBEC Mutagenesis. *Cell* **176**, 1282–1294.e20 (2019).
58. Machado, H. E. *et al.* Diverse mutational landscapes in human lymphocytes. *Nature* **608**, 724–732 (2022).
59. Islam, S. M. A. *et al.* Uncovering novel mutational signatures by de novo extraction with SigProfilerExtractor. *Cell Genom.* **2**, None (2022).
60. Lee-Six, H. *et al.* Population dynamics of normal human blood inferred from somatic mutations. *Nature* **561**, 473–478 (2018).
61. Alexandrov, L. B. *et al.* The repertoire of mutational signatures in human cancer. *Nature* **578**, 94–101 (2020).
62. Gori, K. & Baez-Ortega, A. sigfit: flexible Bayesian inference of mutational signatures. *bioRxiv* (2018) doi:10.1101/372896.
63. Alexandrov, L. B. *et al.* Clock-like mutational processes in human somatic cells. *Nat. Genet.* **47**, 1402–1407 (2015).
64. Abascal, F. *et al.* Somatic mutation landscapes at single-molecule resolution. *Nature* **593**, 405–410 (2021).
65. Moore, L. *et al.* The mutational landscape of human somatic and germline cells. *Nature* **597**, 381–386 (2021).

66. Liu, M. *et al.* Two levels of protection for the B cell genome during somatic hypermutation. *Nature* **451**, 841–845 (2008).
67. Zeng, X. *et al.* DNA polymerase eta is an A-T mutator in somatic hypermutation of immunoglobulin variable genes. *Nat. Immunol.* **2**, 537–541 (2001).
68. Kasar, S. *et al.* Whole-genome sequencing reveals activation-induced cytidine deaminase signatures during indolent chronic lymphocytic leukaemia evolution. *Nat. Commun.* **6**, 8866 (2015).
69. Secrier, M. *et al.* Mutational signatures in esophageal adenocarcinoma define etiologically distinct subgroups with therapeutic relevance. *Nat. Genet.* **48**, 1131–1141 (2016).
