# Supplementary material for: Polyclonal selection of immune checkpoint mutations in thyroid autoimmunity
Source: Nature. 2026 Apr 14;654(8117):131–41. doi: 10.1038/s41586-026-10493-9 (PMC13233322; doi:10.1038/s41586-026-10493-9)
Supplement: Supplementary file 2 — Reporting Summary [file 41586_2026_10493_MOESM2_ESM.pdf]

nature portfolio

Corresponding author(s): Andrew R. J. Lawson & Inigo Martincorena

Last updated by author(s): Apr 1, 2026

Reporting Summary

Nature Portfolio wishes to improve the reproducibility of the work that we publish. This form provides structure for consistency and transparency in reporting. For further information on Nature Portfolio policies, see our Editorial Policies and the Editorial Policy Checklist.

Statistics

For all statistical analyses, confirm that the following items are present in the figure legend, table legend, main text, or Methods section.

n/a	Confirmed	
☐	☒	The exact sample size (*n*) for each experimental group/condition, given as a discrete number and unit of measurement
☒	☐	A statement on whether measurements were taken from distinct samples or whether the same sample was measured repeatedly
☐	☒	The statistical test(s) used AND whether they are one- or two-sided<br>*Only common tests should be described solely by name; describe more complex techniques in the Methods section.*
☐	☒	A description of all covariates tested
☐	☒	A description of any assumptions or corrections, such as tests of normality and adjustment for multiple comparisons
☐	☒	A full description of the statistical parameters including central tendency (e.g. means) or other basic estimates (e.g. regression coefficient) AND variation (e.g. standard deviation) or associated estimates of uncertainty (e.g. confidence intervals)
☐	☒	For null hypothesis testing, the test statistic (e.g. *F*, *t*, *r*) with confidence intervals, effect sizes, degrees of freedom and *P* value noted<br>*Give P values as exact values whenever suitable.*
☐	☒	For Bayesian analysis, information on the choice of priors and Markov chain Monte Carlo settings
☒	☐	For hierarchical and complex designs, identification of the appropriate level for tests and full reporting of outcomes
☒	☐	Estimates of effect sizes (e.g. Cohen's *d*, Pearson's *r*), indicating how they were calculated

Our web collection on statistics for biologists contains articles on many of the points above.

Software and code

Policy information about availability of computer code

Data collection

No software was used for data collection.

Data analysis

Nanorate sequencing data analysis utilised the NanoSeq pipeline (v3.3.0, https://github.com/cancerit/NanoSeq). Targeted methylation analyses were performed with the UXM_deconv pipeline (https://github.com/nloyfer/UXM_deconv/) and the EpiDish R package (https://github.com/sjczheng/EpiDISH/tree/devel, v2.23.1), using the Robust Partial Correlations method and our modified atlas.

Bespoke analyses were performed with R (v4.5) and relevant code can be found as Supplementary Code (HTML). R packages used include: tidyverse (v2), GenomicRanges (v1.62.1), dndscv (v0.0.1.0), scales (v1.4.0), patchwork (v1.3.2), viridis (v0.6.5), RColorBrewer (v1.1-3), lattice (v0.22-6), latticeExtra (v0.6-31), vcfR (v1.15.0), MASS (v7.3-65), jsonlite (v2.0.0), ggforce (v0.5.0), stringi (v1.8.7), gtools (v3.9.5), drc (v3.0-1), pander (v0.6.6), ape (v.5.8-1), ggtree (v4.0.4), ggh4x (v0.3.1) and ggpubr (v0.6.2).

For single nuclei data, all analyses are available on GitHub (https://github.com/alextidd/nicola_et_al_2026). For the single nuclei data, the BaseJumper tool suite was used (https://github.com/alextidd/bj-somatic-variantcalling, commit hash: 2a5dbfd; https://github.com/alextidd/bj-dna-qc, commit hash: 3149537). A Nextflow pipeline was designed to genotype SNPs and NanoSeq mutations, and to calculate coverage in V(D)J regions of single nuclei (https://www.github.com/alextidd/nf-resolveome, commit hash: a785010).

Full code repository for all analyses included in the manuscript will be available at https://doi.org/10.5281/zenodo.19366069.

Bespoke single nuclei analyses were performed with R (v4.4.0). R packages used include: magrittr (v2.0.4), tidyverse (v2.0.0), data.table (v1.18.2.1), ape (v5.8), patchwork (v1.2.0), RColorBrewer (v1.1-3), lsa (v0.73.3), slider (v0.3.2), ggh4x (v0.2.8), janitor (v2.2.0), knitr (v1.51), seqinr (v4.2-36), VGAM (v1.1-12), MASS (v7.3-60.2), devtools (v2.4.6), optparse (v1.7.5), hdp (v0.1.5), sigfit (v2.2), GenomicRanges (v1.56.1),

rtracklayer (v1.64.0), biomaRt (v2.60.1), Rsamtools (v2.20.0), ggtree (v3.12.0), BiocManager (v1.30.26) and treemut (v1.1).

Phylogeny construction was performed using the Sequoia package with MPBoot (v1.1.0). Mutational signature extraction was undertaken with hdp (v0.1.5, https://github.com/nicolaroberts/hdp) and SigProfiler (https://cancer.sanger.ac.uk/signatures/tools/). De novo signatures were deconvoluted to reference signatures in COSMIC v3.5 database (https://cancer.sanger.ac.uk), and other published signatures. Signature fitting was completed with sigfit (v2.2, https://github.com/kgori/sigfit). Further detail can be found in Supplementary Note 2.

For manuscripts utilizing custom algorithms or software that are central to the research but not yet described in published literature, software must be made available to editors and reviewers. We strongly encourage code deposition in a community repository (e.g. GitHub). See the Nature Portfolio guidelines for submitting code & software for further information.

Data

Policy information about availability of data

All manuscripts must include a data availability statement. This statement should provide the following information, where applicable:

- Accession codes, unique identifiers, or web links for publicly available datasets
- A description of any restrictions on data availability
- For clinical datasets or third party data, please ensure that the statement adheres to our policy

All sequencing data has been deposited in EGA and is available within the following 8 datasets:
EGAD00001016058 Polyclonal selection of immune checkpoint mutations in thyroid autoimmunity - ExomeNanoSeq
EGAD00001016059 Polyclonal selection of immune checkpoint mutations in thyroid autoimmunity - TargetedNanoSeq
EGAD00001016060 Polyclonal selection of immune checkpoint mutations in thyroid autoimmunity - TargetedEMSeq
EGAD00001016061 Polyclonal selection of immune checkpoint mutations in thyroid autoimmunity - PTA_WGS
EGAD00001016062 Polyclonal selection of immune checkpoint mutations in thyroid autoimmunity - PTA_DNAHyb
EGAD00001016063 Polyclonal selection of immune checkpoint mutations in thyroid autoimmunity - PTA_RNA
EGAD00001016064 Polyclonal selection of immune checkpoint mutations in thyroid autoimmunity - LCMB_WGS
EGAD00001016065 Polyclonal selection of immune checkpoint mutations in thyroid autoimmunity - LCMB_WES

Xenium data can be found on the EMBL-EBI BioImage Archive (https://www.ebi.ac.uk/biostudies/bioimages/studies/S-BIAD3103, accession number S-BIAD3103, doi 10.6019/S-BIAD3103)

Research involving human participants, their data, or biological material

Policy information about studies with human participants or human data. See also policy information about sex, gender (identity/presentation), and sexual orientation and race, ethnicity and racism.

Reporting on sex and gender

We report sex, not gender, only for the purposes of calculating mutant cell fraction for X-linked genes (as this will vary in homozygous and hemizygous contexts).

Reporting on race, ethnicity, or other socially relevant groupings

We do not make any report on ethnicities or other socially relevant groupings.

Population characteristics

AITD cohort: 14 donors, 4 male, 10 female, age range from 10 - 20 years old to 70 - 79 years old.
Normal PBMC cohort: 28 donors, 13 male, 15 female, median age = 70 years old, range = 20 - 91 years old.
Microdissected lymphoid aggregate cohort: 5 donors, 3 male, 2 female, median age = 47 years old, range = 19 - 72 years old
Lymph node cohort: 4 donors, 2 male, 2 female, median age = 39 years old, range = 19 - 69 years old
Spleen cohort: 30 donors, 13 male, 17 female, median age = 50 years old, range = 20 - 69 years old
Tonsillitis cohort: 5 donors, 2 male, 3 female, median age = 28 years old, range = 20 - 38 years old
Thyroid goitre cohort: 5 donors, 1 male, 4 female, median age 71 years old, range = 56 - 80 years old
Normal thyroid cohort: 3 donors, 1 male, 2 female, median age = 47 years old, range = 19 - 54 years old

Associated metadata includes clinical diagnosis and autoantibody status (anti-TPO) where available.

Recruitment

AITD cohort: Donors were selected on the basis of having at least one of: a clinical diagnosis of autoimmune thyroid disease (either Hashimoto disease or Graves disease), histopathological evidence of intra-thyroidal lymphocytic infiltration, and positive serology for anti-TPO. Samples were provided in the course of thyroid biopsy or resection and donors gave consent for research purposes..
Normal PBMC cohort: Donors with available PBMC samples who were over 50 years old and without a clinical history of autoimmunity were purchased from a commercial supplier.
Microdissected lymphoid aggregate cohort: Donors with available tissue samples (tonsil, ileum, sigmoid colon) and without a clinical history of autoimmunity were purchased from a commercial supplier. Laser capture microdissection was used to excise lymphoid aggregates where present
Lymph node cohort: Donors with available tissue samples and without a clinical history of autoimmunity were purchased from a commercial supplier.
Spleen cohort: Three donors with available tissue samples and without a clinical history of autoimmunity were purchased from a commercial supplier. A further 27 donors were eligible organ transplant donors who donated tissue for research purposes. All donors had no clinical history of cancer or autoimmune disease.
Tonsillitis cohort: Donors with available chronically inflamed tonsil samples and without a clinical history of autoimmunity were purchased from a commercial supplier.
Thyroid goitre cohort: Donors were selected by having available thyroid tissue and a clinical and histopathological diagnosis of a nodular thyroid goitre. Samples were provided in the course of thyroid biopsy or resection and donors gave consent for

	research purposes.<br>Normal thyroid cohort: Donors with available tissue samples and without a clinical history of autoimmunity or other thyroid disease were purchased from a commercial supplier.
Ethics oversight	The use of these samples was approved by the South West - Central Bristol Research Ethics Committee (REC 19/SW/0031).<br>The use of these samples was approved by the London - Surrey Research Ethics Committee (REC 17/LO/1801).<br>The use of these samples was approved by the South Central - Oxford B Research Ethics Committee (REC 21/SC/0158).<br>The use of these samples was approved by the East of England - Cambridge South Research Ethics Committee (REC 15/EE/0152).

Note that full information on the approval of the study protocol must also be provided in the manuscript.

Field-specific reporting

Please select the one below that is the best fit for your research. If you are not sure, read the appropriate sections before making your selection.

☒ Life sciences ☐ Behavioural & social sciences ☐ Ecological, evolutionary & environmental sciences

For a reference copy of the document with all sections, see nature.com/documents/nr-reporting-summary-flat.pdf

Life sciences study design

All studies must disclose on these points even when the disclosure is negative.

Sample size	Information on sample sizes is provided for all analyses. The cohort size was limited by the scarcity of available inflamed thyroid samples across several sources and budgetary considerations.
Data exclusions	No samples were excluded from this study.
Replication	We haven't conducted explicit replication as this was not applicable for an observational study
Randomization	No randomization was performed as this was not applicable for an observational study
Blinding	No blinding was undertaken because this is an observational study.

Reporting for specific materials, systems and methods

We require information from authors about some types of materials, experimental systems and methods used in many studies. Here, indicate whether each material, system or method listed is relevant to your study. If you are not sure if a list item applies to your research, read the appropriate section before selecting a response.

Materials & experimental systems

n/a	Involved in the study	
☐	☒	Antibodies
☒	☐	Eukaryotic cell lines
☒	☐	Palaeontology and archaeology
☒	☐	Animals and other organisms
☒	☐	Clinical data
☒	☐	Dual use research of concern
☒	☐	Plants

Methods

n/a	Involved in the study	
☒	☐	ChIP-seq
☐	☒	Flow cytometry
☒	☐	MRI-based neuroimaging

Antibodies

Antibodies used	Anti-CD20 primary antibody (PA0359, Leica Biosystems, Germany)<br>Anti-CD3 primary antibody (PA0122, Leica Biosystems, Germany)<br>Anti-BCL6 primary antibody (PA0204, Leica Biosystems, Germany)<br>Fluorescein-conjugated oligonucleotide probes targeting Kappa and Lambda light chain mRNA (PB0645 and PB0669, Leica Biosystems, Germany)<br>Anti-fluorescein antibodies (AR0222, Leica Biosystems, Germany)<br>Anti-CD19 primary antibody (ab270715, Abcam, UK)<br>Anti-CD3 primary antibody (ab11089, Abcam, UK)<br>Anti-TNFRSF14 primary antibody (ab314494, Abcam, UK)<br>Anti-thyroglobulin (ab156008, Abcam, UK)<br>Anti-thyroid peroxidase (ab109383, Abcam, UK)<br>Anti-CD19 primary antibody (14-0194-82, Invitrogen, Thermo Fisher Scientific Inc, Waltham, MA, USA)<br>Anti-TNFRSF14 primary antibody (ab314494, Abcam, UK)

Anti-Rat IgG H&L secondary antibody (Alexa Fluor® 568, ab175476, Abcam, UK)
Anti-Rabbit IgG H&L secondary antibody (Alexa Fluor® 647, ab150083, Abcam, UK).

Custom recombinant antibodies were generated by Biointron Biological Inc (Shanghai, China) based on reconstructed heavy and light chain sequences. Positive control nucleotide sequences were obtained from the literature, by searching GenBank for paired heavy and light chain sequences known to bind human TPO or thyroglobulin: AJ238327.1 and AJ238330.1 (clone ICA5, anti-human TPO); AJ399834.1 and AJ399876.1 (clone T8, anti-human TPO); AY365327.1 and AY365334.1 (clone #6, anti-human thyroglobulin); AY365330.1 and AY365338.1 (clone #26, anti-human thyroglobulin).

For ELISA, recombinant antigens used were: human thyroid peroxidase (NM_000547, OriGene, MD, USA, TP310659) and human thyroglobulin (NM_003235, OriGene, MD, USA, TP316216). Recombinant monoclonal antibodies and isotype control antibodies were also used (Biointron Biological Inc, Shanghai, ChinaBiointron, B117901, B646501, B730001). Horseradish peroxidase-conjugated secondary antibodies: goat anti-human IgG (Fc-specific) (Sigma-Aldrich, Merck KGaA, Germany A0170, 1:10,000 dilution) or goat anti-rabbit IgG antibody (Arigo Biolaboratories, Taiwan, China, ARG65351, 1:10,000 dilution).

Validation

Data provided in the manuscript and on manufacturer's website.

Plants

Seed stocks

Report on the source of all seed stocks or other plant material used. If applicable, state the seed stock centre and catalogue number. If plant specimens were collected from the field, describe the collection location, date and sampling procedures.

Novel plant genotypes

Describe the methods by which all novel plant genotypes were produced. This includes those generated by transgenic approaches, gene editing, chemical/radiation-based mutagenesis and hybridization. For transgenic lines, describe the transformation method, the number of independent lines analyzed and the generation upon which experiments were performed. For gene-edited lines, describe the editor used, the endogenous sequence targeted for editing, the targeting guide RNA sequence (if applicable) and how the editor was applied.

Authentication

Describe any authentication procedures for each seed stock used or novel genotype generated. Describe any experiments used to assess the effect of a mutation and, where applicable, how potential secondary effects (e.g. second site T-DNA insertions, mosiacism, off-target gene editing) were examined.

Flow Cytometry

Plots

Confirm that:

☒ The axis labels state the marker and fluorochrome used (e.g. CD4-FITC).

☒ The axis scales are clearly visible. Include numbers along axes only for bottom left plot of group (a 'group' is an analysis of identical markers).

☒ All plots are contour plots with outliers or pseudocolor plots.

☒ A numerical value for number of cells or percentage (with statistics) is provided.

Methodology

Sample preparation

Nuclei dissociation was performed using a modified Slide-tags protocol, a single-nucleus barcoding technique developed for multimodal spatial genomics. The main deviation from this protocol was the absence of mounting tissue sections on the proprietary pucks necessary for spatial mapping. Dissociated nuclei were stained with a fluorescent intercalating dye (propidium iodide) for 30 minutes at 4 degrees and then filtered through a 30 µm filter prior to flow sorting.

Instrument

Single nuclei were sorted using a Bigfoot spectral cell sorter (Invitrogen, Thermo Fisher Scientific Inc, Waltham, MA, USA).

Software

Sorting was carried out using Sasquatch (SQ) software (Invitrogen, Thermo Fisher Scientific Inc, Waltham, MA, USA). Representative gating plots were prepared using FCS Express 7 Research.

Cell population abundance

Flow sorting was only being used to separate intact individual nuclei from doublets, non-nuclear debris and nuclei that were not intact due to cryosectioning. Representative proportions of intact nuclei are shown in Extended Data Fig. 6a.

Gating strategy

Propidium iodide was excited using a 561 nm laser and emission collected using a 625/15 bandpass filter. Gating was set on PI (area) vs PI (width) to discriminate doublets. Single nuclei were sorted into pre-calibrated 96 well plates on a single sorting mode. Representative gating strategy is shown in Extended Data Fig. 6a.

☒ Tick this box to confirm that a figure exemplifying the gating strategy is provided in the Supplementary Information.
