# Supplementary material for: Polyclonal selection of immune checkpoint mutations in thyroid autoimmunity
Source: Nature. 2026 Apr 14;654(8117):131–41. doi: 10.1038/s41586-026-10493-9 (PMC13233322; doi:10.1038/s41586-026-10493-9)
Supplement: Supplementary file 11 — This zipped file contains Supplementary Code. [file 41586_2026_10493_MOESM11_ESM.zip › HashimotoAnalysis.html]

 

 

 

 
 
 


 

 Polyclonal selection of immune checkpoint mutations in thyroid autoimmunity 

 
 
 
 
 
 
 
 
 
 
 
 
 
 
 

 

 
 


 


 

 

 
 


 


 

 


 


 
 
 
 
 
 

 


 

 
  Code     
 
  Show All Code  
  Hide All Code  
 
 


 Polyclonal selection of immune checkpoint
mutations in thyroid autoimmunity 
 01 April 2026 

 


   
 
 1. Input file paths and environment 
 
 Load packages 
  # Loading the required packages
library(readxl)
library(GenomicRanges)
library(tidyverse)
library(dndscv)
library(scales)
library(patchwork)
library(viridis)
library(RColorBrewer)
library(lattice)
library(latticeExtra)
library(knitr)
library(vcfR)
library(MASS)
library(jsonlite)
library(ggforce)
library(XML)
library(stringi)
library(gtools)
library(drc)
library(pander)
library(ape)
library(ggtree)
library(ggh4x)
library(ggpubr)  
   
 
 
 Input files and environment set-up 
  # Key input files
metadata_file <- "./input/hashimoto_exome_targeted_combined_summary_stats.tsv"
mutations_file <- "./input/hashimoto_exome_targeted_combined_muts.tsv"
discarded_variants_file <- "./input/discarded_variants_high_vaf_drivers.tsv"
coverage_file <- "./input/hashimoto_exome_targeted_combined_per_sample_cov.tsv"
emseq_file <- "./input/hashimoto_emseq_cell_fractions_updated_atlas_epidish.tsv"
targeted_panel_file <- "./input/Sanger_Immune-v1_TE-91661256_hg19_gene_list.tsv"
B_mem_int_syn_burden_file <- "./input/combined_B_mem_1_final.tsv"

# dNdS SHM input files
RefCDS_GRCh37_NSX <- "./input/RefCDS_GRCh37_v1.NSXupdate.Rdat"
RefCDS_GRCh37_codon <- "./input/RefCDS_GRCh37_v1_NSX_codon.Rdat"
RefCDS_shm_exome <- "./input/shm_exome_5e-07_B_mem_v3.Rdat"
RefCDS_noshm_exome <- "./input/noshm_exome_5e-07_B_mem_v3.Rdat"
RefCDS_noshm_only_codon <- "./input/RefCDS_GRCh37_v1_NSX_noshm_codon.Rdat"

# Within gene dNdS input files
genomeFile <- "./input/hs37d5.fa"
per_gene_cov_dir <- "./input/per_gene_per_site_cov/"
TNFRSF14_ptm_json <- "./input/2025-07-18_TNFRSF14_Uniprot_Q92956_PTM.json"
TNFRSF14_domain_json <- "./input/2025-07-18_TNFRSF14_Uniprot_Q92956_Domains.json"
CD274_ptm_json <- "./input/2025-07-22_CD274_Uniprot_Q9NZQ7_PTM.json"
CBL_domain_json <- "./input/2025-07-22_CBL_Uniprot_P22681_Domains.json"

# Normal lymphocyte Exome and Targeted NanoSeq data
normal_mut_file <- "./input/normal_lymphocytes/normal_lymphocytes_exome_targeted_combined_muts.tsv"
normal_cov_file <- "./input/normal_lymphocytes/normal_lymphocytes_exome_targeted_combined_per_sample_cov.tsv"
normal_metadata_file <- "./input/normal_lymphocytes/normal_lymphocytes_exome_targeted_combined_summary_stats.tsv"

# Additional control samples
lcm_lymphocyte_mut_file <- "./input/other_normal_samples/lcm_lymphocyte_combined_muts.tsv"
lcm_lymphocyte_cov_file <- "./input/other_normal_samples/lcm_lymphocyte_combined_per_sample_cov.tsv"
lcm_lymphocyte_metadata_file <- "./input/other_normal_samples/lcm_lymphocyte_combined_summary_stats.tsv"
lymph_node_mut_file <- "./input/other_normal_samples/lymph_node_combined_muts.tsv"
lymph_node_cov_file <- "./input/other_normal_samples/lymph_node_combined_per_sample_cov.tsv"
lymph_node_metadata_file <- "./input/other_normal_samples/lymph_node_combined_summary_stats.tsv"
spleen_mut_file <- "./input/other_normal_samples/spleen_combined_muts.tsv"
spleen_cov_file <- "./input/other_normal_samples/spleen_combined_per_sample_cov.tsv"
spleen_metadata_file <- "./input/other_normal_samples/spleen_combined_summary_stats.tsv"
tonsillitis_mut_file <- "./input/other_normal_samples/tonsillitis_combined_muts.tsv"
tonsillitis_cov_file <- "./input/other_normal_samples/tonsillitis_combined_per_sample_cov.tsv"
tonsillitis_metadata_file <- "./input/other_normal_samples/tonsillitis_combined_summary_stats.tsv"
thyroid_goitre_mut_file <- "./input/other_normal_samples/thyroid_goitre_combined_muts.tsv"
thyroid_goitre_cov_file <- "./input/other_normal_samples/thyroid_goitre_combined_per_sample_cov.tsv"
thyroid_goitre_metadata_file <- "./input/other_normal_samples/thyroid_goitre_combined_summary_stats.tsv"

# Spatial mapping of mutations to LCM microdissections
LCM_record_file <- "./input/spatial_mapping/LCM_database.xlsx"
pileups_dir <- "./input/spatial_mapping/"
PD63118b_outline_file <- "./input/spatial_mapping/PD63118b_Thyroid_LCM_BiopsyOutline.ndpi.ndpa"
PD63118b_cuts_file <- "./input/spatial_mapping/PD63118b_Thyroid_LCM_Microdissections.ndpi.ndpa"
PD63126b_outline_file <- "./input/spatial_mapping/PD63126b_Thyroid_LCM_BiopsyOutline.ndpi.ndpa"
PD63126b_cuts_file <- "./input/spatial_mapping/PD63126b_Thyroid_LCM_Microdissections.ndpi.ndpa"
PD66711a_outline_file <- "./input/spatial_mapping/PD66711a_Thyroid_LCM_BiopsyOutline.ndpi.ndpa"
PD66711a_cuts_file <- "./input/spatial_mapping/PD66711a_Thyroid_LCM_Microdissections.ndpi.ndpa"
PD66718b_outline_file <- "./input/spatial_mapping/PD66718b_Thyroid_LCM_BiopsyOutline.ndpi.ndpa"
PD66718b_cuts_file <- "./input/spatial_mapping/PD66718b_Thyroid_LCM_Microdissections.ndpi.ndpa"

# Spatial mapping for 2nd batch of LCM microdissections
LCM_record_batch_2_file <- "./input/spatial_mapping_batch_2/LCM_batch_2_database.xlsx"
pileups_batch_2_dir <- "./input/spatial_mapping_batch_2/"
PD63118b_batch_2_outline_file <- "./input/spatial_mapping_batch_2/PD63118b_s31s32_LCM_BiopsyOutline.ndpi.ndpa"
PD63118b_batch_2_cuts_file <- "./input/spatial_mapping_batch_2/PD63118b_s31s32_LCM_Microdissections.ndpi.ndpa"
PD63121b_batch_2_outline_file <- "./input/spatial_mapping_batch_2/PD63121b_s28s29_LCM_BiopsyOutline.ndpi.ndpa"
PD63121b_batch_2_cuts_file <- "./input/spatial_mapping_batch_2/PD63121b_s28s29_LCM_Microdissections.ndpi.ndpa"
PD63126b_batch_2_outline_file <- "./input/spatial_mapping_batch_2/PD63126b_s28s29_LCM_BiopsyOutline.ndpi.ndpa"
PD63126b_batch_2_cuts_file <- "./input/spatial_mapping_batch_2/PD63126b_s28s29_LCM_Microdissections.ndpi.ndpa"

# PTA input files for donor H1
pta_additional_annotation_H1_file <- "./input/pta/H1_PD63118_pta_additional_annotation.tsv"
pta_dna_hyb_mutation_H1_file <- "./input/pta/H1_PD63118_DNAHyb_filtered_mutations.tsv"
pta_dna_hyb_rescued_muts_H1_file <- "./input/pta/H1_PD63118_DNAHyb_rescued_mutations.xlsx"
pta_H1_de_novo_WGS_calls_phylogeny_file <- "./input/pta/H1_de_novo_WGS_calls_phylogeny.tree"
pta_H1_de_novo_trinuc_mut_mat_hdp_file <- "./input/pta/H1_de_novo_trinuc_mut_mat_hdp.txt"
pta_H1_de_novo_sigfit_exposures_file <- "./input/pta/H1_de_novo_sigfit_exposures_per_branch_with_indels.rds"
pta_H1_de_novo_per_branch_exposures_file <- "./input/pta/H1_de_novo_per_branch_exposures_filled.rds"
pta_H1_de_novo_exposures_per_cell_file <- "./input/pta/H1_de_novo_exposures_per_cell.rds"
pta_H1_reconstructed_BCR_shm_counts_file <- "./input/pta/H1_reconstructed_BCR_shm_counts_per_cell.tsv"

# PTA input files for donors H2, H3 and H8
pta_additional_annotation_H2_H8_file <- "./input/pta/H2_H8_pta_additional_annotation.tsv"
pta_dna_hyb_mutation_H2_file <- "./input/pta/H2_PD63121_DNAHyb_filtered_mutations.tsv"
pta_dna_hyb_mutation_H3_file <- "./input/pta/H3_PD63126_DNAHyb_filtered_mutations.tsv"
pta_dna_hyb_mutation_H8_file <- "./input/pta/H8_PD66718_DNAHyb_filtered_mutations.tsv"
pta_dna_hyb_rescued_muts_H8_file <- "./input/pta/H8_PD66718_DNAHyb_rescued_mutations.xlsx"

# Donor H1 synthesised antibody sequences ELISA results
synthesised_antibody_ELISA_file <- "./input/antibody_synthesis/SynthesisedAntibodies_ELISA_OD450.tsv"

# Load functions
source(file = "./code/SortedBloodFunctions.R")

# Save files (Use T when running the code manually to save figures as pdf)
runman = F   
 
 
 Load key input files 
  metadata <- read.table(metadata_file, sep = "\t", stringsAsFactors = F, header = T)
mutations <- read.table(mutations_file, sep = "\t", stringsAsFactors = F, header = T)
discarded_variants <- read.table(discarded_variants_file, sep = "\t", stringsAsFactors = F, header = T, tryLogical = F)
coverage <- read.table(coverage_file, sep = "\t", stringsAsFactors = F, header = T)
emseq <- read.table(emseq_file, sep = "\t", stringsAsFactors = F, header = T)
immune_genes <- read.table(targeted_panel_file, sep = "\t", stringsAsFactors = F, header = T)
B_mem_int_syn_burden <- read.table(B_mem_int_syn_burden_file, sep = "\t", stringsAsFactors = F, header = T)  
 
 
 Rescue genuine mutations that were discarded for exceeding VAF >
0.1 threshold during calling 
 Only one mutation rescued: DNMT3A mutation in PD66718. This is the
sole coding non-synonymous SNV in discarded variants with VAF > 0.1
and < 0.4 that is not in the HLA region. 
  mutations <- rbind(mutations,discarded_variants)  
 
 
 Annotate combined muts with donor, cell fraction and mutation site
columns 
  mutations$donor <- substr(mutations$paper_id,1,2)

mutations$mut_site <- paste(substr(mutations$sampleID,1,7),mutations$chr,mutations$pos,mutations$ref,mutations$mut,sep = "_")

males <- metadata$sample[which(metadata$sex == "Male")]
mutations$cellfraction = mutations$duplex_vaf * 2
mutations$cellfraction[mutations$chr %in% c("X","Y") & mutations$sampleID %in% males] = mutations$duplex_vaf[mutations$chr %in% c("X","Y") & mutations$sampleID %in% males]

mutations$bam_mut_adj = pmax(0,mutations$bam_mut-mutations$times_called)
mutations$bam_cov_adj = mutations$bam_cov-mutations$duplex_cov
mutations$bam_vaf_adj = mutations$bam_mut_adj / mutations$bam_cov_adj
mutations$bam_adj_cellfraction = mutations$bam_vaf_adj * 2
mutations$bam_adj_cellfraction[mutations$chr %in% c("X","Y") & mutations$sampleID %in% males] = mutations$bam_vaf_adj[mutations$chr %in% c("X","Y") & mutations$sampleID %in% males]  
 
 
 Annotate start losses 
 Impact of mutations annotated by dNdScv. This does not annotate start
losses separately from other missense mutations, so annotate those
separately here. 
  # Define start_losses
mutations$impact[substr(mutations$aachange,2,nchar(mutations$aachange)-1) == "1"] <- "Start_loss"  
 
 
 Define cohorts 
  # Pilot exome cohort
pilot_exome <- metadata$sample[metadata$cohort == "Pilot" & metadata$sequencing_type == "Exome"] 
pilot_targeted <- metadata$sample[metadata$cohort == "Pilot" & metadata$sequencing_type == "Targeted"] 
pilot_all <- metadata$sample[metadata$cohort == "Pilot"] 

extension_all <- metadata$sample[metadata$cohort == "Extension"] 
extension_hashimoto <- metadata$sample[metadata$cohort == "Extension" & metadata$pathology == "Hashimoto"] 
extension_graves <- metadata$sample[metadata$cohort == "Extension" & metadata$pathology == "Graves"] 

extension_bulk <- metadata$sample[metadata$cohort == "Extension" & metadata$sample_type == "Bulk"] 
extension_LCM <- metadata$sample[metadata$cohort == "Extension" &  metadata$sample_type == "LCM"]

emseq_normal_thyroid <- emseq$sampleID[substr(emseq$paper_id,1,1) == "N"]
emseq_normal_spleen <- emseq$sampleID[substr(emseq$paper_id,1,1) == "S"]
emseq_normal_lymph_node <- emseq$sampleID[substr(emseq$paper_id,1,2) == "LN"]
emseq_B_mem <- emseq$sampleID[substr(emseq$paper_id,1,1) == "B"]
emseq_CD4_mem <- emseq$sampleID[substr(emseq$paper_id,1,2) == "HT"]
emseq_CD8_mem <- emseq$sampleID[substr(emseq$paper_id,1,2) == "CT"]  
   
 
 
 
 2. Targeted enzymatic methylation sequencing (EM-Seq) 
 
 Pilot cohort cell type composition 
 Using Targeted EM-Seq, we assessed the cellular composition of the
tissue biopsies by comparing methylation patterns to a published
reference atlas (PMID: 36599988). 
  # Print EM-Seq cell proportions for pilot Hashimoto cohort (exclude normal thyroid and malignant samples)
emseq_pilot_summary <- summary(emseq[substr(emseq$sampleID,1,8) %in% substr(pilot_exome,1,8),!(colnames(emseq) %in% c("sampleID_emseq","sampleID","paper_id"))])
summary_row_names <- gsub(":.*","",emseq_pilot_summary[,1])
emseq_pilot_summary <- gsub(".*:","",emseq_pilot_summary)
emseq_pilot_summary <- gsub(" ","",emseq_pilot_summary)
emseq_pilot_summary <- apply(emseq_pilot_summary, 2, function(x) as.numeric(x))
row.names(emseq_pilot_summary) <- summary_row_names
kable(t(signif(emseq_pilot_summary)), digits =3, caption = "EM-Seq cell proportions for pilot Hashimoto cohort")  
 
 EM-Seq cell proportions for pilot Hashimoto cohort 
 
 
 
 
 
 
 
 
 
 
 
  
 Min. 
 1st Qu. 
 Median 
 Mean 
 3rd Qu. 
 Max. 
 
 
 
 
 Adipocytes 
 0.000 
 0.000 
 0.000 
 0.004 
 0.000 
 0.022 
 
 
 Blood_B 
 0.147 
 0.196 
 0.210 
 0.237 
 0.297 
 0.338 
 
 
 Blood_Granulocyte 
 0.000 
 0.000 
 0.000 
 0.000 
 0.000 
 0.000 
 
 
 Blood_MonocyteMacrophage 
 0.000 
 0.000 
 0.000 
 0.002 
 0.004 
 0.008 
 
 
 Blood_NK 
 0.000 
 0.000 
 0.000 
 0.004 
 0.003 
 0.022 
 
 
 Blood_T 
 0.432 
 0.458 
 0.469 
 0.471 
 0.491 
 0.504 
 
 
 Endothelial 
 0.001 
 0.032 
 0.038 
 0.039 
 0.043 
 0.081 
 
 
 Fibroblast 
 0.048 
 0.053 
 0.069 
 0.075 
 0.099 
 0.110 
 
 
 SmoothMuscle 
 0.000 
 0.000 
 0.000 
 0.005 
 0.000 
 0.032 
 
 
 ThyroidEpithelium 
 0.012 
 0.069 
 0.094 
 0.083 
 0.109 
 0.125 
 
 
 Other 
 0.038 
 0.078 
 0.088 
 0.080 
 0.091 
 0.097 
 
 
 
  # Print EM-Seq cell proportions for normal thyroid samples with no history of autoimmune thyroiditis
emseq_normal_summary <- summary(emseq[emseq$sampleID %in% emseq_normal_thyroid,!(colnames(emseq) %in% c("sampleID_emseq","sampleID","paper_id"))])
summary_row_names <- gsub(":.*","",emseq_normal_summary[,1])
emseq_normal_summary <- gsub(".*:","",emseq_normal_summary)
emseq_normal_summary <- gsub(" ","",emseq_normal_summary)
emseq_normal_summary <- apply(emseq_normal_summary, 2, function(x) as.numeric(x))
row.names(emseq_normal_summary) <- summary_row_names
kable(t(signif(emseq_normal_summary)), digits =3, caption = "EM-Seq cell proportions for normal thyroid")  
 
 EM-Seq cell proportions for normal thyroid 
 
 
 
 
 
 
 
 
 
 
 
  
 Min. 
 1st Qu. 
 Median 
 Mean 
 3rd Qu. 
 Max. 
 
 
 
 
 Adipocytes 
 0.000 
 0.000 
 0.000 
 0.003 
 0.004 
 0.009 
 
 
 Blood_B 
 0.008 
 0.017 
 0.026 
 0.021 
 0.028 
 0.030 
 
 
 Blood_Granulocyte 
 0.000 
 0.000 
 0.000 
 0.000 
 0.000 
 0.000 
 
 
 Blood_MonocyteMacrophage 
 0.000 
 0.000 
 0.000 
 0.003 
 0.005 
 0.010 
 
 
 Blood_NK 
 0.000 
 0.000 
 0.000 
 0.000 
 0.000 
 0.000 
 
 
 Blood_T 
 0.055 
 0.060 
 0.065 
 0.075 
 0.086 
 0.107 
 
 
 Endothelial 
 0.077 
 0.089 
 0.101 
 0.110 
 0.126 
 0.151 
 
 
 Fibroblast 
 0.001 
 0.004 
 0.007 
 0.006 
 0.009 
 0.012 
 
 
 SmoothMuscle 
 0.004 
 0.005 
 0.005 
 0.018 
 0.024 
 0.044 
 
 
 ThyroidEpithelium 
 0.592 
 0.674 
 0.757 
 0.704 
 0.760 
 0.763 
 
 
 Other 
 0.029 
 0.049 
 0.070 
 0.059 
 0.075 
 0.080 
 
 
 
 
 
 Extension cohort cell type composition 
 Next this was applied to the extension cohort, which included both
bulk and LCM samples. 
  # Print EM-Seq cell proportions for bulk samples from extension cohort
emseq_extension_summary <- summary(emseq[emseq$sampleID %in% substr(extension_bulk,1,16),!(colnames(emseq) %in% c("sampleID_emseq","sampleID","paper_id"))])
summary_row_names <- gsub(":.*","",emseq_extension_summary[,1])
emseq_extension_summary <- gsub(".*:","",emseq_extension_summary)
emseq_extension_summary <- gsub(" ","",emseq_extension_summary)
emseq_extension_summary <- apply(emseq_extension_summary, 2, function(x) as.numeric(x))
row.names(emseq_extension_summary) <- summary_row_names
kable(t(signif(emseq_extension_summary)), digits =3, caption = "EM-Seq cell proportions for extension Hashimoto + Graves cohort")  
 
 EM-Seq cell proportions for extension Hashimoto + Graves
cohort 
 
 
 
 
 
 
 
 
 
 
 
  
 Min. 
 1st Qu. 
 Median 
 Mean 
 3rd Qu. 
 Max. 
 
 
 
 
 Adipocytes 
 0.010 
 0.019 
 0.028 
 0.030 
 0.043 
 0.051 
 
 
 Blood_B 
 0.050 
 0.083 
 0.117 
 0.127 
 0.149 
 0.300 
 
 
 Blood_Granulocyte 
 0.000 
 0.001 
 0.004 
 0.003 
 0.005 
 0.009 
 
 
 Blood_MonocyteMacrophage 
 0.000 
 0.015 
 0.018 
 0.022 
 0.024 
 0.071 
 
 
 Blood_NK 
 0.000 
 0.000 
 0.000 
 0.002 
 0.003 
 0.011 
 
 
 Blood_T 
 0.033 
 0.106 
 0.150 
 0.154 
 0.194 
 0.292 
 
 
 Endothelial 
 0.069 
 0.082 
 0.103 
 0.100 
 0.109 
 0.137 
 
 
 Fibroblast 
 0.004 
 0.013 
 0.018 
 0.020 
 0.024 
 0.045 
 
 
 SmoothMuscle 
 0.021 
 0.041 
 0.055 
 0.055 
 0.070 
 0.091 
 
 
 ThyroidEpithelium 
 0.066 
 0.313 
 0.384 
 0.374 
 0.467 
 0.610 
 
 
 Other 
 0.075 
 0.096 
 0.118 
 0.112 
 0.125 
 0.145 
 
 
 
  # Print EM-Seq cell proportions for laser capture microdissected lymphoid aggregates from extension cohort
emseq_lcm_summary <- summary(emseq[emseq$sampleID %in% substr(extension_LCM,1,16),!(colnames(emseq) %in% c("sampleID_emseq","sampleID","paper_id"))])
summary_row_names <- gsub(":.*","",emseq_lcm_summary[,1])
emseq_lcm_summary <- gsub(".*:","",emseq_lcm_summary)
emseq_lcm_summary <- gsub(" ","",emseq_lcm_summary)
emseq_lcm_summary <- apply(emseq_lcm_summary, 2, function(x) as.numeric(x))
row.names(emseq_lcm_summary) <- summary_row_names
kable(t(signif(emseq_lcm_summary)), digits =3, caption = "EM-Seq cell proportions for laser-microdissected lymphocytes")  
 
 EM-Seq cell proportions for laser-microdissected
lymphocytes 
 
 
 
 
 
 
 
 
 
 
 
  
 Min. 
 1st Qu. 
 Median 
 Mean 
 3rd Qu. 
 Max. 
 
 
 
 
 Adipocytes 
 0.004 
 0.010 
 0.014 
 0.016 
 0.024 
 0.030 
 
 
 Blood_B 
 0.283 
 0.318 
 0.407 
 0.418 
 0.517 
 0.567 
 
 
 Blood_Granulocyte 
 0.000 
 0.000 
 0.000 
 0.000 
 0.000 
 0.000 
 
 
 Blood_MonocyteMacrophage 
 0.000 
 0.000 
 0.000 
 0.001 
 0.000 
 0.004 
 
 
 Blood_NK 
 0.000 
 0.000 
 0.000 
 0.001 
 0.000 
 0.005 
 
 
 Blood_T 
 0.253 
 0.290 
 0.322 
 0.374 
 0.472 
 0.547 
 
 
 Endothelial 
 0.017 
 0.020 
 0.032 
 0.033 
 0.043 
 0.051 
 
 
 Fibroblast 
 0.011 
 0.018 
 0.029 
 0.033 
 0.044 
 0.063 
 
 
 SmoothMuscle 
 0.002 
 0.011 
 0.018 
 0.018 
 0.024 
 0.036 
 
 
 ThyroidEpithelium 
 0.009 
 0.023 
 0.040 
 0.052 
 0.071 
 0.121 
 
 
 Other 
 0.025 
 0.031 
 0.046 
 0.055 
 0.069 
 0.110 
 
 
 
 
 
 Control datasets cell type composition 
  # Print EM-Seq cell proportions for normal bulk spleen samples used as control dataset
emseq_spleen_summary <- summary(emseq[emseq$sampleID %in% emseq_normal_spleen,!(colnames(emseq) %in% c("sampleID_emseq","sampleID","paper_id"))])
summary_row_names <- gsub(":.*","",emseq_spleen_summary[,1])
emseq_spleen_summary <- gsub(".*:","",emseq_spleen_summary)
emseq_spleen_summary <- gsub(" ","",emseq_spleen_summary)
emseq_spleen_summary <- apply(emseq_spleen_summary, 2, function(x) as.numeric(x))
row.names(emseq_spleen_summary) <- summary_row_names
kable(t(signif(emseq_spleen_summary)), digits =3, caption = "EM-Seq cell proportions for normal bulk spleen samples used as control dataset")  
 
 EM-Seq cell proportions for normal bulk spleen samples used as
control dataset 
 
 
 
 
 
 
 
 
 
 
 
  
 Min. 
 1st Qu. 
 Median 
 Mean 
 3rd Qu. 
 Max. 
 
 
 
 
 Adipocytes 
 0.000 
 0.000 
 0.000 
 0.003 
 0.000 
 0.048 
 
 
 Blood_B 
 0.070 
 0.159 
 0.216 
 0.210 
 0.259 
 0.408 
 
 
 Blood_Granulocyte 
 0.000 
 0.002 
 0.008 
 0.018 
 0.021 
 0.109 
 
 
 Blood_MonocyteMacrophage 
 0.000 
 0.020 
 0.033 
 0.035 
 0.054 
 0.096 
 
 
 Blood_NK 
 0.000 
 0.018 
 0.021 
 0.022 
 0.025 
 0.047 
 
 
 Blood_T 
 0.168 
 0.242 
 0.276 
 0.283 
 0.319 
 0.478 
 
 
 Endothelial 
 0.133 
 0.187 
 0.254 
 0.257 
 0.302 
 0.440 
 
 
 Fibroblast 
 0.022 
 0.063 
 0.075 
 0.079 
 0.094 
 0.137 
 
 
 SmoothMuscle 
 0.000 
 0.000 
 0.000 
 0.001 
 0.000 
 0.012 
 
 
 ThyroidEpithelium 
 0.001 
 0.003 
 0.005 
 0.005 
 0.008 
 0.012 
 
 
 Other 
 0.051 
 0.067 
 0.078 
 0.086 
 0.091 
 0.178 
 
 
 
  # Print EM-Seq cell proportions for normal lymph node samples used as control dataset
emseq_lymph_node_summary <- summary(emseq[emseq$sampleID %in% emseq_normal_lymph_node,!(colnames(emseq) %in% c("sampleID_emseq","sampleID","paper_id"))])
summary_row_names <- gsub(":.*","",emseq_lymph_node_summary[,1])
emseq_lymph_node_summary <- gsub(".*:","",emseq_lymph_node_summary)
emseq_lymph_node_summary <- gsub(" ","",emseq_lymph_node_summary)
emseq_lymph_node_summary <- apply(emseq_lymph_node_summary, 2, function(x) as.numeric(x))
row.names(emseq_lymph_node_summary) <- summary_row_names
kable(t(signif(emseq_lymph_node_summary)), digits =3, caption = "EM-Seq cell proportions for normal bulk lymph node samples used as control dataset")  
 
 EM-Seq cell proportions for normal bulk lymph node samples used
as control dataset 
 
 
 
 
 
 
 
 
 
 
 
  
 Min. 
 1st Qu. 
 Median 
 Mean 
 3rd Qu. 
 Max. 
 
 
 
 
 Adipocytes 
 0.000 
 0.000 
 0.000 
 0.004 
 0.004 
 0.018 
 
 
 Blood_B 
 0.143 
 0.197 
 0.275 
 0.262 
 0.340 
 0.356 
 
 
 Blood_Granulocyte 
 0.000 
 0.000 
 0.000 
 0.000 
 0.000 
 0.000 
 
 
 Blood_MonocyteMacrophage 
 0.000 
 0.000 
 0.027 
 0.032 
 0.059 
 0.073 
 
 
 Blood_NK 
 0.000 
 0.000 
 0.000 
 0.002 
 0.003 
 0.009 
 
 
 Blood_T 
 0.360 
 0.363 
 0.406 
 0.441 
 0.484 
 0.591 
 
 
 Endothelial 
 0.066 
 0.067 
 0.080 
 0.097 
 0.109 
 0.162 
 
 
 Fibroblast 
 0.020 
 0.035 
 0.042 
 0.048 
 0.055 
 0.086 
 
 
 SmoothMuscle 
 0.000 
 0.000 
 0.000 
 0.000 
 0.000 
 0.000 
 
 
 ThyroidEpithelium 
 0.001 
 0.004 
 0.005 
 0.004 
 0.005 
 0.007 
 
 
 Other 
 0.062 
 0.083 
 0.090 
 0.109 
 0.117 
 0.194 
 
 
 
  # Print EM-Seq cell proportions for sorted B memory cell samples used as control dataset
emseq_B_mem_summary <- summary(emseq[emseq$sampleID %in% emseq_B_mem,!(colnames(emseq) %in% c("sampleID_emseq","sampleID","paper_id"))])
summary_row_names <- gsub(":.*","",emseq_B_mem_summary[,1])
emseq_B_mem_summary <- gsub(".*:","",emseq_B_mem_summary)
emseq_B_mem_summary <- gsub(" ","",emseq_B_mem_summary)
emseq_B_mem_summary <- apply(emseq_B_mem_summary, 2, function(x) as.numeric(x))
row.names(emseq_B_mem_summary) <- summary_row_names
kable(t(signif(emseq_B_mem_summary)), digits =3, caption = "EM-Seq cell proportions for sorted B memory cell samples used as control dataset")  
 
 EM-Seq cell proportions for sorted B memory cell samples used
as control dataset 
 
 
 
 
 
 
 
 
 
 
 
  
 Min. 
 1st Qu. 
 Median 
 Mean 
 3rd Qu. 
 Max. 
 
 
 
 
 Adipocytes 
 0.000 
 0.004 
 0.004 
 0.004 
 0.006 
 0.007 
 
 
 Blood_B 
 0.790 
 0.805 
 0.848 
 0.854 
 0.881 
 0.945 
 
 
 Blood_Granulocyte 
 0.000 
 0.000 
 0.000 
 0.000 
 0.000 
 0.000 
 
 
 Blood_MonocyteMacrophage 
 0.000 
 0.000 
 0.000 
 0.004 
 0.000 
 0.020 
 
 
 Blood_NK 
 0.000 
 0.000 
 0.000 
 0.004 
 0.007 
 0.015 
 
 
 Blood_T 
 0.031 
 0.093 
 0.104 
 0.096 
 0.122 
 0.128 
 
 
 Endothelial 
 0.000 
 0.000 
 0.002 
 0.002 
 0.002 
 0.006 
 
 
 Fibroblast 
 0.000 
 0.003 
 0.008 
 0.011 
 0.017 
 0.025 
 
 
 SmoothMuscle 
 0.000 
 0.000 
 0.001 
 0.004 
 0.006 
 0.013 
 
 
 ThyroidEpithelium 
 0.000 
 0.000 
 0.000 
 0.001 
 0.000 
 0.003 
 
 
 Other 
 0.009 
 0.013 
 0.016 
 0.020 
 0.021 
 0.041 
 
 
 
  # Print EM-Seq cell proportions for sorted T CD4 memory cell samples used as control dataset
emseq_CD4_mem_summary <- summary(emseq[emseq$sampleID %in% emseq_CD4_mem,!(colnames(emseq) %in% c("sampleID_emseq","sampleID","paper_id"))])
summary_row_names <- gsub(":.*","",emseq_CD4_mem_summary[,1])
emseq_CD4_mem_summary <- gsub(".*:","",emseq_CD4_mem_summary)
emseq_CD4_mem_summary <- gsub(" ","",emseq_CD4_mem_summary)
emseq_CD4_mem_summary <- apply(emseq_CD4_mem_summary, 2, function(x) as.numeric(x))
row.names(emseq_CD4_mem_summary) <- summary_row_names
kable(t(signif(emseq_CD4_mem_summary)), digits =3, caption = "EM-Seq cell proportions for sorted T CD4 memory cell samples used as control dataset")  
 
 EM-Seq cell proportions for sorted T CD4 memory cell samples
used as control dataset 
 
 
 
 
 
 
 
 
 
 
 
  
 Min. 
 1st Qu. 
 Median 
 Mean 
 3rd Qu. 
 Max. 
 
 
 
 
 Adipocytes 
 0.000 
 0.001 
 0.002 
 0.002 
 0.004 
 0.006 
 
 
 Blood_B 
 0.005 
 0.040 
 0.052 
 0.042 
 0.054 
 0.057 
 
 
 Blood_Granulocyte 
 0.000 
 0.001 
 0.001 
 0.001 
 0.002 
 0.002 
 
 
 Blood_MonocyteMacrophage 
 0.000 
 0.000 
 0.000 
 0.002 
 0.002 
 0.009 
 
 
 Blood_NK 
 0.000 
 0.000 
 0.000 
 0.001 
 0.001 
 0.004 
 
 
 Blood_T 
 0.904 
 0.905 
 0.914 
 0.917 
 0.926 
 0.938 
 
 
 Endothelial 
 0.000 
 0.000 
 0.001 
 0.002 
 0.002 
 0.004 
 
 
 Fibroblast 
 0.001 
 0.003 
 0.005 
 0.005 
 0.007 
 0.010 
 
 
 SmoothMuscle 
 0.000 
 0.004 
 0.007 
 0.006 
 0.009 
 0.012 
 
 
 ThyroidEpithelium 
 0.000 
 0.000 
 0.000 
 0.000 
 0.000 
 0.000 
 
 
 Other 
 0.007 
 0.015 
 0.022 
 0.021 
 0.028 
 0.033 
 
 
 
  # Print EM-Seq cell proportions for sorted T CD8 memory cell samples used as control dataset
emseq_CD8_mem_summary <- summary(emseq[emseq$sampleID %in% emseq_CD8_mem,!(colnames(emseq) %in% c("sampleID_emseq","sampleID","paper_id"))])
summary_row_names <- gsub(":.*","",emseq_CD8_mem_summary[,1])
emseq_CD8_mem_summary <- gsub(".*:","",emseq_CD8_mem_summary)
emseq_CD8_mem_summary <- gsub(" ","",emseq_CD8_mem_summary)
emseq_CD8_mem_summary <- apply(emseq_CD8_mem_summary, 2, function(x) as.numeric(x))
row.names(emseq_CD8_mem_summary) <- summary_row_names
kable(t(signif(emseq_CD8_mem_summary)), digits =3, caption = "EM-Seq cell proportions for sorted T CD8 memory cell samples used as control dataset")  
 
 EM-Seq cell proportions for sorted T CD8 memory cell samples
used as control dataset 
 
 
 
 
 
 
 
 
 
 
 
  
 Min. 
 1st Qu. 
 Median 
 Mean 
 3rd Qu. 
 Max. 
 
 
 
 
 Adipocytes 
 0.000 
 0.000 
 0.000 
 0.001 
 0.002 
 0.004 
 
 
 Blood_B 
 0.014 
 0.016 
 0.018 
 0.032 
 0.044 
 0.068 
 
 
 Blood_Granulocyte 
 0.000 
 0.000 
 0.000 
 0.001 
 0.002 
 0.003 
 
 
 Blood_MonocyteMacrophage 
 0.000 
 0.000 
 0.000 
 0.003 
 0.002 
 0.012 
 
 
 Blood_NK 
 0.000 
 0.000 
 0.000 
 0.000 
 0.000 
 0.001 
 
 
 Blood_T 
 0.889 
 0.895 
 0.929 
 0.920 
 0.941 
 0.944 
 
 
 Endothelial 
 0.000 
 0.001 
 0.003 
 0.002 
 0.003 
 0.005 
 
 
 Fibroblast 
 0.001 
 0.004 
 0.006 
 0.009 
 0.012 
 0.023 
 
 
 SmoothMuscle 
 0.000 
 0.000 
 0.000 
 0.001 
 0.000 
 0.004 
 
 
 ThyroidEpithelium 
 0.000 
 0.000 
 0.000 
 0.000 
 0.001 
 0.002 
 
 
 Other 
 0.010 
 0.016 
 0.033 
 0.030 
 0.041 
 0.050 
 
 
 
 
 
 Cell type composition plot for pilot and normal thyroid samples 
  methylation_colours <- setNames(c(RColorBrewer::brewer.pal("Set3", n = ncol(emseq) - 3)),c("Blood_B","Blood_T","Blood_MonocyteMacrophage","Blood_NK","Blood_Granulocyte","ThyroidEpithelium","Endothelial","Fibroblast","Adipocytes","SmoothMuscle","Other"))

emseq_plot <- reshape2::melt(emseq, id.vars = c("sampleID_emseq","sampleID","paper_id"))

emseq_plot$cohort <- NA
emseq_plot$cohort[which(emseq_plot$sampleID %in% emseq_normal_thyroid)] <- "normal"
emseq_plot$cohort[which(emseq_plot$sampleID %in% substr(metadata$sample[metadata$cohort == "Pilot"],1,16))] <- "pilot"
emseq_plot$cohort[which(emseq_plot$sampleID %in% substr(metadata$sample[metadata$cohort == "Extension" & metadata$pathology == "Hashimoto" & metadata$sample_type == "Bulk"],1,16))] <- "ex_hash_bulk"
emseq_plot$cohort[which(emseq_plot$sampleID %in% substr(metadata$sample[metadata$cohort == "Extension" & metadata$pathology == "Graves" & metadata$sample_type == "Bulk"],1,16))] <- "ex_graves_bulk"
emseq_plot$cohort[which(emseq_plot$sampleID %in% substr(metadata$sample[metadata$cohort == "Extension" & metadata$sample_type == "LCM"],1,16))] <- "ex_lcm"
emseq_plot$cohort[which(emseq_plot$sampleID %in% emseq_normal_spleen)] <- "spleen"
emseq_plot$cohort[which(emseq_plot$sampleID %in% emseq_normal_lymph_node)] <- "lymph_node"
emseq_plot$cohort[which(emseq_plot$sampleID %in% emseq_B_mem)] <- "b_mem"
emseq_plot$cohort[which(emseq_plot$sampleID %in% emseq_CD4_mem)] <- "cd4_mem"
emseq_plot$cohort[which(emseq_plot$sampleID %in% emseq_CD8_mem)] <- "cd8_mem"

# Order by B-cell fraction pilot cohort
emseq_plot_order <- emseq_plot[which(emseq_plot$variable == "Blood_B"),] %>% arrange(desc(value), .by_group = TRUE)
emseq_plot$sampleID <- factor(emseq_plot$sampleID, levels = emseq_plot_order$sampleID)
emseq_plot$variable <- factor(emseq_plot$variable, levels = names(methylation_colours))
emseq_plot$cohort <- factor(emseq_plot$cohort, levels = c("normal","pilot","ex_hash_bulk","ex_graves_bulk","ex_lcm","spleen","lymph_node","b_mem","cd4_mem","cd8_mem"))

if (runman) { dev.new(width=5, height=6) }
ggplot(data = emseq_plot[which(emseq_plot$cohort %in% c("normal","pilot") & !(emseq_plot$paper_id %in% c("H3b","H3c"))),], aes(x = sampleID, y = value, fill = variable)) +
  geom_bar(position="fill", stat = "identity") +
  scale_y_continuous(expand = c(0,0)) +
  scale_fill_manual(values = methylation_colours) +
  scale_x_discrete(labels = setNames(emseq_plot$paper_id,emseq_plot$sampleID)) +
  labs(x = "", y = "Cell fraction", fill = "Cell type") +
  theme_minimal() +
  theme(axis.text = element_text(face = "bold", size = 12),axis.text.x = element_text(size = 12), axis.title = element_text(face = "bold", size = 12), plot.title = element_blank(), legend.position = "bottom") +
  guides(fill = guide_legend(nrow = 6)) +
  facet_grid(.~cohort, space = "free", scales = "free_x", labeller = as_labeller(c(normal = "Normal thyroid", pilot = "Pilot", ex_hash_bulk = "Extension Hashimoto (bulk)", ex_graves_bulk = "Extension Graves (bulk)", ex_lcm = "Extension (LCM)"))) +
  theme(strip.text.x = element_text(size = 12), strip.background = element_blank(), strip.text = element_text(face = "bold", size = 12), panel.grid.major.y = element_blank(), panel.grid.minor.y = element_blank())  
    
  if (runman) { dev.copy(pdf,"./emseq_barplot_normal_pilot.pdf",width=5,height=6); dev.off() }  
 
 
 Cell type composition plot for extension samples 
  if (runman) { dev.new(width=8, height=6) }
ggplot(data = emseq_plot[which(emseq_plot$cohort %in% c("ex_hash_bulk","ex_graves_bulk","ex_lcm")),], aes(x = sampleID, y = value, fill = variable)) +
  geom_bar(position="fill", stat = "identity") +
  scale_y_continuous(expand = c(0,0)) +
  scale_fill_manual(values = methylation_colours) +
  scale_x_discrete(labels = setNames(emseq_plot$paper_id,emseq_plot$sampleID)) +
  labs(x = "", y = "Cell fraction", fill = "Cell type") +
  theme_minimal() +
  theme(axis.text = element_text(face = "bold", size = 12),axis.text.x = element_text(size = 12), axis.title = element_text(face = "bold", size = 12), plot.title = element_blank(), legend.position = "bottom") +
  guides(fill = guide_legend(nrow = 6)) +
  facet_grid(.~cohort, space = "free", scales = "free_x", labeller = as_labeller(c(normal = "Normal thyroid", pilot = "Pilot", ex_hash_bulk = "Extension Hashimoto (bulk)", ex_graves_bulk = "Extension Graves (bulk)", ex_lcm = "Extension (LCM)"))) +
  theme(strip.text.x = element_text(size = 12), strip.background = element_blank(), strip.text = element_text(face = "bold", size = 12), panel.grid.major.y = element_blank(), panel.grid.minor.y = element_blank())  
    
  if (runman) { dev.copy(pdf,"./emseq_barplot_extension.pdf",width=8,height=6); dev.off() }  
 
 
 Cell type composition plot across all AITD samples 
  if (runman) { dev.new(width=20, height=4) }
ggplot(data = emseq_plot[which(emseq_plot$cohort %in% c("normal","pilot","ex_hash_bulk","ex_graves_bulk","ex_lcm")),], aes(x = sampleID, y = value, fill = variable)) +
  geom_bar(position="fill", stat = "identity") +
  scale_y_continuous(expand = c(0,0)) +
  scale_fill_manual(values = methylation_colours) +
  scale_x_discrete(labels = setNames(emseq_plot$paper_id,emseq_plot$sampleID)) +
  labs(x = "", y = "Cell fraction", fill = "Cell type") +
  theme_minimal() +
  theme(axis.text = element_text(face = "bold", size = 12),axis.text.x = element_text(size = 12), axis.title = element_text(face = "bold", size = 12), plot.title = element_blank(), legend.position = "bottom") +
  guides(fill = guide_legend(nrow = 1)) +
  facet_grid(.~cohort, space = "free", scales = "free_x", labeller = as_labeller(c(normal = "Normal thyroid", pilot = "Pilot", ex_hash_bulk = "Extension Hashimoto (bulk)", ex_graves_bulk = "Extension Graves (bulk)", ex_lcm = "Extension (LCM)"))) +
  theme(strip.text.x = element_text(size = 12), strip.background = element_blank(), strip.text = element_text(face = "bold", size = 12), panel.grid.major.y = element_blank(), panel.grid.minor.y = element_blank())  
    
  if (runman) { dev.copy(pdf,"./emseq_barplot_all.pdf",width=20,height=6); dev.off() }  
 
 
 Cell type composition plot across control dataset samples 
  if (runman) { dev.new(width=20, height=4) }
ggplot(data = emseq_plot[which(emseq_plot$cohort %in% c("spleen","lymph_node","b_mem","cd4_mem","cd8_mem")),], aes(x = sampleID, y = value, fill = variable)) +
  geom_bar(position="fill", stat = "identity") +
  scale_y_continuous(expand = c(0,0)) +
  scale_fill_manual(values = methylation_colours) +
  scale_x_discrete(labels = setNames(emseq_plot$paper_id,emseq_plot$sampleID)) +
  labs(x = "", y = "Cell fraction", fill = "Cell type") +
  theme_minimal() +
  theme(axis.text = element_text(face = "bold", size = 12),axis.text.x = element_blank(), axis.title = element_text(face = "bold", size = 12), plot.title = element_blank(), legend.position = "bottom") +
  guides(fill = guide_legend(nrow = 1)) +
  facet_grid(.~cohort, space = "free", scales = "free_x", labeller = as_labeller(c(spleen = "Spleen", lymph_node = "Lymph node", b_mem = "Sorted B memory", cd4_mem = "Sorted T CD4 memory", cd8_mem = "Sorted T CD8 memory"))) +
  theme(strip.text.x = element_text(size = 12), strip.background = element_blank(), strip.text = element_text(face = "bold", size = 12), panel.grid.major.y = element_blank(), panel.grid.minor.y = element_blank())  
    
  if (runman) { dev.copy(pdf,"./emseq_barplot_control.pdf",width=20,height=6); dev.off() }  
   
 
 
 
 3. Coverage metrics for exome pilot cohort 
  # Combined duplex coverage in pilot cohort exome data
cat(paste0("Cumulative exome NanoSeq duplex coverage in the pilot cohort = ", sum(metadata[which(metadata$sample %in% pilot_exome),]$median_duplex_cov), " dx"))  
  ## Cumulative exome NanoSeq duplex coverage in the pilot cohort = 3125 dx  
  # Individual duplex coverage in pilot cohort exome data
kable(metadata[which(metadata$sample %in% pilot_exome),] %>% group_by(substr(paper_id,1,2)) %>% summarise(dx = sum(median_duplex_cov)) %>% rename(Donor = `substr(paper_id, 1, 2)`), caption = "Duplex coverage per donor for pilot exome data")  
 
 Duplex coverage per donor for pilot exome data 
 
 
 Donor 
 dx 
 
 
 
 
 H1 
 770 
 
 
 H2 
 1254 
 
 
 H3 
 1101 
 
 
 
  # Total coding mutations (unique per donor) in pilot exome data
cat(paste0("Total coding mutations (unique per donor) = ", length(unique(mutations$mut_site[which(!(is.na(mutations$impact)) & mutations$sampleID %in% pilot_exome)])),"\n"))  
  ## Total coding mutations (unique per donor) = 28855  
  cat(paste0("Total non-synonymous mutations (unique per donor) = ", length(unique(mutations$mut_site[which(!(is.na(mutations$impact)) & mutations$impact != "Synonymous" & mutations$sampleID %in% pilot_exome)]))))  
  ## Total non-synonymous mutations (unique per donor) = 21431  
   
 
 
 4. dNdSshm for exome pilot cohort 
  sample_list <- pilot_exome

dnds_muts <- mutations[which(mutations$sampleID %in% sample_list),]
dnds_muts <- dnds_muts[which(!(duplicated(dnds_muts$mut_site))),]

dnds_cov <- rowSums(coverage[which(colnames(coverage) %in% sample_list)])
names(dnds_cov) <- coverage$gene
dnds_cov <- dnds_cov[which(dnds_cov != 0)]

# 2024-02-19 - Made decision to exclude IGLL5 and IGLL1 from dNdS analysis. Only reason these Ig genes (but not others) are in the dNdS object is they are in frame.
dnds_cov <- dnds_cov[which(!(names(dnds_cov) %in% c("IGLL5","IGLL1")))]

pilot_exome_dnds_shm <- dnds_shm(mutations = dnds_muts[,c("sampleID","chr","pos","ref","mut")],
                                 refdb_noshm = RefCDS_noshm_exome,
                                 refdb_shm = RefCDS_shm_exome,
                                 dc_noshm = dnds_cov,
                                 gene_list = names(dnds_cov),
                                 # Exons with just the start codon need to be excluded (synonymous mutations are not possible for dNdSloc)
                                 excl_shm = c("IGLL1","IGLL5","MYO1E","ZNF595","AC026202.1","EHBP1L1"),
                                 excl_noshm = c("IGLL1","IGLL5"),
                                 onesided = T,
                                 maxcovs = 10)  
  # Significant genes in pilot exome data
pilot_exome_drivers <- pilot_exome_dnds_shm$sel_merged$gene_name[pilot_exome_dnds_shm$sel_merged$qglobalpos_m < 0.01]

# Total number of non-synonymous mutations across driver genes in pilot exome data
pilot_driver_muts <- mutations[mutations$gene %in% pilot_exome_drivers & mutations$sampleID %in% pilot_exome,]
pilot_driver_muts_unique <- pilot_driver_muts[!(duplicated(pilot_driver_muts$mut_site)),]

pilot_driver_counts <- sort(colSums(table(substr(pilot_driver_muts_unique$paper_id[pilot_driver_muts_unique$impact != "Synonymous"],1,2), pilot_driver_muts_unique$gene[pilot_driver_muts_unique$impact != "Synonymous"])), decreasing = T)

# Sort driver list by decreasing number of mutations
pilot_exome_drivers <- names(pilot_driver_counts)

kable(as.data.frame(pilot_driver_counts), caption = "Non-synonymous driver gene mutations (unique per donor) in pilot exome data", col.names = c("Gene","Mutation count"))  
 
 Non-synonymous driver gene mutations (unique per donor) in
pilot exome data 
 
 
 Gene 
 Mutation count 
 
 
 
 
 TNFRSF14 
 102 
 
 
 CD274 
 40 
 
 
 TET2 
 18 
 
 
 TNFAIP3 
 11 
 
 
 
  pilot_exome_no_shm_snv_ci <- dndscv::geneci(dndsout = pilot_exome_dnds_shm, gene_list = pilot_exome_drivers)
pilot_exome_no_shm_indel_ci <- geneindelci(geneindels = pilot_exome_dnds_shm$geneindels, gene_list = pilot_exome_drivers, theta = pilot_exome_dnds_shm$nbregind$theta)

pilot_exome_no_shm_gene_ci <- cbind(pilot_exome_no_shm_snv_ci,pilot_exome_no_shm_indel_ci[,c(2:ncol(pilot_exome_no_shm_indel_ci))])

pilot_exome_shm_gene_ci <- geneciloc(pilot_exome_dnds_shm$genemuts_shm)  
  # Number of non-synonymous mutations per donor across driver genes in pilot exome data
kable(table(substr(pilot_driver_muts_unique$paper_id[pilot_driver_muts_unique$impact != "Synonymous"],1,2), pilot_driver_muts_unique$gene[pilot_driver_muts_unique$impact != "Synonymous"])[,pilot_exome_drivers], caption = "Per donor non-synonymous mutations in driver genes from pilot exome data")  
 
 Per donor non-synonymous mutations in driver genes from pilot
exome data 
 
 
  
 TNFRSF14 
 CD274 
 TET2 
 TNFAIP3 
 
 
 
 
 H1 
 72 
 17 
 7 
 7 
 
 
 H2 
 16 
 20 
 2 
 2 
 
 
 H3 
 14 
 3 
 9 
 2 
 
 
 
  # dN/dS ratios across driver genes in pilot exome data
dnds_ratios <- pilot_exome_dnds_shm$sel_merged[pilot_exome_dnds_shm$sel_merged$gene_name %in% pilot_exome_drivers,c("gene_name","wmis_cv","wnon_cv","wspl_cv","wind_cv","qglobalpos_m")]
dnds_ratios$qglobalpos_m <- format(dnds_ratios$qglobalpos_m, digits = 3)
kable(dnds_ratios, caption = "dN/dS ratios for driver genes in pilot exome data", digits = 1, row.names = F, col.names = c("Gene","dN/dS missense","dN/dS nonsense", "dN/dS essential splice", "dN/dS indels","Q-value (global positive merged)"))  
 
 dN/dS ratios for driver genes in pilot exome data 
 
 
 
 
 
 
 
 
 
 
 Gene 
 dN/dS missense 
 dN/dS nonsense 
 dN/dS essential splice 
 dN/dS indels 
 Q-value (global positive merged) 
 
 
 
 
 CD274 
 34.9 
 243.5 
 243.5 
 251.2 
 0.00e+00 
 
 
 TNFRSF14 
 148.3 
 1608.6 
 1608.6 
 333.7 
 0.00e+00 
 
 
 TET2 
 0.8 
 19.9 
 19.9 
 21.9 
 1.25e-06 
 
 
 TNFAIP3 
 1.2 
 31.4 
 31.4 
 44.5 
 9.06e-06 
 
 
 
  plotpanels = letters[c(1,8,9)]
plotwidth = 3
plotheight = as.numeric(2.5 * length(plotpanels))

driver_discovery = driverplot(
  genes2plot=list(pilot_exome_drivers),
  muts=mutations,
  exome_dnds_sel_cv = list(pilot_exome_dnds_shm$sel_merged),
  exome_dnds_sel_cv_gene_ci = list(pilot_exome_no_shm_gene_ci),
  exome_dnds_loc = vector("list",1),
  exome_dnds_loc_gene_ci = vector("list",1),
  targeted_dnds_sel_cv = vector("list",1),
  targeted_dnds_sel_cv_gene_ci = vector("list",1),
  targeted_dnds_loc = vector("list",1),
  targeted_dnds_loc_gene_ci = vector("list",1),
  combined_targeted_only_dnds_sel_cv = vector("list",1),
  combined_targeted_only_dnds_sel_cv_gene_ci = vector("list",1),
  combined_targeted_only_dnds_loc = vector("list",1),
  combined_targeted_only_dnds_loc_gene_ci = vector("list",1),
  driverdensity_sampleIDs = list(pilot_exome),
  full_sampleIDs = list(pilot_exome),
  gene2dc_array = coverage,
  min_syn_loc = 3,
  max_genes = Inf,
  sortbyfreq = T,
  onlysignifdnds = F,
  logscale = F,
  logscale_dnds = T,
  dnds_plot_cap = 3000,
  nmuts_cap = 500,
  nonsyn_mcf_cap = 25,
  max_duplex_cov = 80000,
  runman = runman,
  plotfilename = "./output/Fig1C_IndexSamplesExomeDriverDiscovery.pdf",
  plotpanels = plotpanels,
  plottitles =  "Pilot exome",
  highlighted_genes = vector("list",1))  
    
  if (runman) { dev.new() }

samples2plot <- pilot_exome
genes2plot <- pilot_exome_drivers

muts2plot <- mutations[which(mutations$gene %in% genes2plot & mutations$sampleID %in% samples2plot),]
uniq2plot <- muts2plot[which(!(duplicated(muts2plot$mut_site))),]
donors2plot <- unique(substr(muts2plot$paper_id,1,2))

plot_rows <- 3
plot_cols <- length(genes2plot)

layout(matrix(c(1:(plot_rows*plot_cols)), nrow = plot_rows, ncol = plot_cols, byrow = F), widths = rep(4,times = length(genes2plot)))
par(mar = c(5,5.5,2,2))

nmut_array <- table(substr(uniq2plot$paper_id,1,2),
                    uniq2plot$impact,
                    uniq2plot$gene)

genes2plot = names(sort(apply(nmut_array, MARGIN = c(3), sum),decreasing=T)) # Sorting the genes in decreasing order of their number of mutations

nmuts_cap <- ceiling(max(apply(nmut_array, MARGIN = c(1,3), sum)) / 10) * 10

# Calculate cell fractions
cellfraction = array(NA, dim = c(length(donors2plot),8,length(genes2plot)), dimnames = list(donors2plot, c("mis","non","spl","ind","mislow","nonlow","spllow","indlow"),genes2plot))

for(x in 1:length(genes2plot)) {  
  for (j in 1:length(donors2plot)) {
    aux = muts2plot[which(muts2plot$gene==genes2plot[x] & substr(muts2plot$paper_id,1,2) == donors2plot[j]),c("sampleID","paper_id","impact","cellfraction","duplex_vaf")]
    per_sample_gene_cov <- coverage[which(coverage$gene == genes2plot[x]),colnames(coverage) %in% samples2plot[samples2plot %in% muts2plot$sampleID[substr(muts2plot$paper_id,1,2) == donors2plot[j]]]]
    aux$sample_gene_cov <- NA
    aux$cov_weighting <- NA
    for(k in 1:length(per_sample_gene_cov)){
      aux$sample_gene_cov[which(aux$sampleID == names(per_sample_gene_cov)[k])] <- as.numeric(per_sample_gene_cov[k])
    }
    aux$cov_weighting <- aux$sample_gene_cov / sum(per_sample_gene_cov)
    
    aux$weighted_cellfraction <- aux$cellfraction * aux$cov_weighting
    aux$weighted_duplex_vaf <- aux$duplex_vaf * aux$cov_weighting
    
    cellfraction[j,c("mis","mislow"),x] = colSums(aux[aux$impact %in% c("Missense","Start_loss","Stop_loss"),c("weighted_cellfraction","weighted_duplex_vaf")], na.rm = T)
    cellfraction[j,c("non","nonlow"),x] = colSums(aux[aux$impact=="Nonsense",c("weighted_cellfraction","weighted_duplex_vaf")], na.rm = T)
    cellfraction[j,c("spl","spllow"),x] = colSums(aux[aux$impact=="Essential_Splice",c("weighted_cellfraction","weighted_duplex_vaf")], na.rm = T)
    cellfraction[j,c("ind","indlow"),x] = colSums(aux[aux$impact=="no-SNV",c("weighted_cellfraction","weighted_duplex_vaf")], na.rm = T)
  }
}

# Object to output median adjusted bam VAF per gene across donors
median_bam_vaf_adj <- vector(mode = "numeric",length = length(genes2plot))
names(median_bam_vaf_adj) <- genes2plot
max_bam_vaf_adj <- median_bam_vaf_adj
below_1_bam_vaf_adj <- median_bam_vaf_adj

# Create three plots per donor: number of mutations, per mutation (mean across samples) unbiased VAF and mutant cell fraction
for(x in 1:length(genes2plot)){
  nmuts <- nmut_array[,,genes2plot[x]]
  subs_per_donor = t(nmuts)
  subs_per_donor = subs_per_donor[c("Synonymous","Missense","Start_loss","Stop_loss","Nonsense","Essential_Splice","no-SNV"),]
  rownames(subs_per_donor)[rownames(subs_per_donor) == "Essential_Splice"] <- "Splice"
  rownames(subs_per_donor)[rownames(subs_per_donor) == "no-SNV"] <- "Indels"
  
  colvec = c("Synonymous"="grey70","Missense"="cadetblue","Start_loss"="firebrick","Stop_loss"="red","Nonsense"="darkorchid4","Splice"="darkorchid2","Indels"="chocolate3")
  
  pos = barplot(subs_per_donor, las = 1, col=colvec, border=NA, ylim=c(0,nmuts_cap), ylab="Mutations per donor", main = genes2plot[x])
  
  if(x == 1){
    legend("topright",y=max(apply(subs_per_donor,2,sum))*1.09,legend=rownames(subs_per_donor),fill=colvec,border=NA,box.col=NA,bg="transparent")
  }
  
  # Calculate the mean unbiased VAF for mutations detected in multiple sequencing libraries from a donor
  # This avoids representing the same mutation multiple times in the scatter plot
  uniq2plot$mean_bam_vaf_adj <- NA
  for(y in 1:nrow(uniq2plot)){
    uniq2plot$mean_bam_vaf_adj[y] <- mean(pilot_driver_muts$bam_vaf_adj[pilot_driver_muts$mut_site == uniq2plot$mut_site[y]])
  }
  
  uniq2plot_cap <- 1.1*max(uniq2plot$mean_bam_vaf_adj)
  
  df_plot <- uniq2plot[uniq2plot$gene == genes2plot[x],]
  df_plot <- df_plot[order(df_plot$paper_id,decreasing = F),]
  df_plot$idx <- jitter(pos[as.numeric(substr(df_plot$paper_id,2,2))])
  df_plot$impact[df_plot$impact == "Essential_Splice"] <- "Splice"
  df_plot$impact[df_plot$impact == "no-SNV"] <- "Indels"
  df_plot$colour <- colvec[df_plot$impact]
  
  plot(df_plot$idx,df_plot$mean_bam_vaf_adj,col=df_plot$colour,xlab=NA, 
       xlim=c(0.2,3.6), ylim=c(0,uniq2plot_cap),ylab = "Unbiased VAF", bg="transparent", pch = 19, cex = 2, frame.plot = FALSE, axes = F)
  axis(side = 2, las = 1)
  axis(side = 1, at=pos, labels=unique(substr(df_plot$paper_id,1,2)), tick = F, pos = NA)
  
  for(y in 1:length(pos)){
    segments(x0 = pos[y] - 0.5, x1 = pos[y] + 0.5, 
             y0=median(df_plot$mean_bam_vaf_adj[which(substr(df_plot$paper_id,2,2) == y)]), 
             y1 = median(df_plot$mean_bam_vaf_adj[which(substr(df_plot$paper_id,2,2) == y)]), 
             lty = 2, lwd = 1.5)
  }
  
  median_bam_vaf_adj[x] <- median(df_plot$mean_bam_vaf_adj[df_plot$impact != "Synonymous"])
  max_bam_vaf_adj[x] <- max(df_plot$mean_bam_vaf_adj[df_plot$impact != "Synonymous"])
  below_1_bam_vaf_adj[x] <- 100 * length(which(df_plot$mean_bam_vaf_adj[df_plot$impact != "Synonymous"] < 0.01)) / length(df_plot$mean_bam_vaf_adj[df_plot$impact != "Synonymous"])
  
  nonsyndens_cap <- ceiling(max(apply(cellfraction[,1:4,] * 100,3,rowSums)) / 10) * 10
  
  nonsyndens = cellfraction[,,x] * 100
  nonsyndens_bounds = cbind(up = rowSums(nonsyndens[,1:4]), low=rowSums(nonsyndens[,5:8]))
  aux = t(cbind(nonsyndens_bounds[,2],nonsyndens_bounds[,1]-nonsyndens_bounds[,2]))
  
  # This allows a bar to be visible when MCF is zero.
  aux[which(aux == 0)] <- 0.001
  
  pos = barplot(aux[2,], col=c("indianred3"), border=c("indianred3"), ylab="% cells non-synonymous mutation", ylim=c(0,nonsyndens_cap), offset = aux[1,], las = 1)
}  
    
  # Median unbiased VAF of non-synonymous mutations across donors (pilot exome only)
kable(median_bam_vaf_adj, digits = 5, col.names = c("Gene","Median unbiased VAF"), caption = "Median unbiased VAF of non-synonymous mutations across donors (pilot exome only)")  
 
 Median unbiased VAF of non-synonymous mutations across donors
(pilot exome only) 
 
 
 Gene 
 Median unbiased VAF 
 
 
 
 
 TNFRSF14 
 0.00197 
 
 
 CD274 
 0.00060 
 
 
 TET2 
 0.00053 
 
 
 TNFAIP3 
 0.00037 
 
 
 
  # Maximum unbiased VAF of non-synonymous mutations across donors (pilot exome only)
kable(max_bam_vaf_adj, digits = 5, col.names = c("Gene","Maximum unbiased VAF"), caption = "Maximum unbiased VAF of non-synonymous mutations across donors (pilot exome only)")  
 
 Maximum unbiased VAF of non-synonymous mutations across donors
(pilot exome only) 
 
 
 Gene 
 Maximum unbiased VAF 
 
 
 
 
 TNFRSF14 
 0.02442 
 
 
 CD274 
 0.01918 
 
 
 TET2 
 0.02373 
 
 
 TNFAIP3 
 0.00360 
 
 
 
  # Percentage of non-synonymous mutations with unbiased VAF below 1% (pilot exome only)
kable(below_1_bam_vaf_adj, digits = 1, col.names = c("Gene","% muts"), caption = "Percentage of non-synonymous mutations with unbiased VAF below 1% (pilot exome only)")  
 
 Percentage of non-synonymous mutations with unbiased VAF below
1% (pilot exome only) 
 
 
 Gene 
 % muts 
 
 
 
 
 TNFRSF14 
 90.2 
 
 
 CD274 
 97.5 
 
 
 TET2 
 94.4 
 
 
 TNFAIP3 
 100.0 
 
 
 
  # Per donor aggregated mutant cell fractions (pilot exome only)
kable(as.data.frame(colSums(aperm(cellfraction[,5:8,], c(2,1,3))) * 100), caption = "Per donor aggregated mutant cell % (pilot exome only)", digits = 2)  
 
 Per donor aggregated mutant cell % (pilot exome only) 
 
 
  
 TNFRSF14 
 CD274 
 TET2 
 TNFAIP3 
 
 
 
 
 H1 
 32.74 
 4.95 
 3.47 
 1.12 
 
 
 H2 
 2.97 
 4.44 
 0.10 
 0.20 
 
 
 H3 
 5.46 
 0.30 
 1.02 
 0.70 
 
 
 
  pilot_driver_muts_unique$sum_times_called <- NA
for(x in 1:nrow(pilot_driver_muts_unique)){
  pilot_driver_muts_unique$sum_times_called[x] <- sum(pilot_driver_muts$times_called[pilot_driver_muts$mut_site == pilot_driver_muts_unique$mut_site[x]])
}

kable(table(pilot_driver_muts_unique$gene[pilot_driver_muts_unique$sum_times_called == 1 & pilot_driver_muts_unique$impact != "Synonymous"])[pilot_exome_drivers], caption = "Unique mutations detected in only a single read bundle in exome data from pilot cohort", col.names = c("Gene","Mutation count"))  
 
 Unique mutations detected in only a single read bundle in exome
data from pilot cohort 
 
 
 Gene 
 Mutation count 
 
 
 
 
 TNFRSF14 
 50 
 
 
 CD274 
 22 
 
 
 TET2 
 11 
 
 
 TNFAIP3 
 8 
 
 
 
  kable(table(pilot_driver_muts_unique$gene[pilot_driver_muts_unique$sum_times_called > 1 & pilot_driver_muts_unique$impact != "Synonymous"])[pilot_exome_drivers], caption = "Unique mutations detected in multiple read bundles in exome data from pilot cohort", col.names = c("Gene","Mutation count"))  
 
 Unique mutations detected in multiple read bundles in exome
data from pilot cohort 
 
 
 Gene 
 Mutation count 
 
 
 
 
 TNFRSF14 
 52 
 
 
 CD274 
 18 
 
 
 TET2 
 7 
 
 
 TNFAIP3 
 3 
 
 
 
  kable(table(pilot_driver_muts_unique$gene[pilot_driver_muts_unique$sum_times_called == 1 & pilot_driver_muts_unique$impact != "Synonymous"])[pilot_exome_drivers] / table(pilot_driver_muts_unique$gene[ pilot_driver_muts_unique$impact != "Synonymous"])[pilot_exome_drivers] * 100, caption = "% unique mutations detected in only a single read bundle in exome data from pilot cohort", digits = 1, col.names = c("Gene","%"))  
 
 % unique mutations detected in only a single read bundle in
exome data from pilot cohort 
 
 
 Gene 
 % 
 
 
 
 
 TNFRSF14 
 49.0 
 
 
 CD274 
 55.0 
 
 
 TET2 
 61.1 
 
 
 TNFAIP3 
 72.7 
 
 
 
   
 
 
 5. Coverage metrics for targeted data alone from pilot cohort 
  # Combined duplex coverage in pilot cohort targeted data
cat(paste0("Cumulative targeted NanoSeq duplex coverage in the pilot cohort = ", sum(metadata[which(metadata$sample %in% pilot_targeted),]$median_duplex_cov), " dx"))  
  ## Cumulative targeted NanoSeq duplex coverage in the pilot cohort = 29584 dx  
  # Individual duplex coverage in pilot cohort targeted data
kable(metadata[which(metadata$sample %in% pilot_targeted),] %>% group_by(substr(paper_id,1,2)) %>% summarise(dx_per_donor = sum(median_duplex_cov)) %>% rename(Donor = `substr(paper_id, 1, 2)`), caption = "Per donor duplex coverage in pilot cohort targeted data", col.names = c("Donor","dx"))  
 
 Per donor duplex coverage in pilot cohort targeted
data 
 
 
 Donor 
 dx 
 
 
 
 
 H1 
 7574 
 
 
 H2 
 12047 
 
 
 H3 
 9963 
 
 
 
  # Per block duplex coverage in pilot cohort targeted data
# NB - H3b and H3c are thyroid carcinoma samples from the same donor with very little lymphocytic infiltration.
kable(metadata[which(metadata$sample %in% pilot_targeted),] %>% group_by(paper_id) %>% summarise(dx_per_donor = sum(median_duplex_cov)), caption = "Per block duplex coverage in pilot cohort targeted data", col.names = c("Block","dx"))  
 
 Per block duplex coverage in pilot cohort targeted
data 
 
 
 Block 
 dx 
 
 
 
 
 H1 
 7574 
 
 
 H2a 
 3209 
 
 
 H2b 
 2829 
 
 
 H2c 
 2895 
 
 
 H2d 
 3114 
 
 
 H3a 
 8924 
 
 
 H3b 
 412 
 
 
 H3c 
 627 
 
 
 
 
 
 6. Combined mutation counts for combined targeted and exome data
from pilot cohort 
  # Total number of non-synonymous mutations across initial 4 driver genes in combined pilot exome and targeted data
pilot_all_exome_driver_muts <- mutations[mutations$gene %in% pilot_exome_drivers & mutations$sampleID %in% pilot_all,]
pilot_all_exome_driver_muts_unique <- pilot_all_exome_driver_muts[!(duplicated(pilot_all_exome_driver_muts$mut_site)),]

combined_muts <- colSums(table(substr(pilot_all_exome_driver_muts_unique$paper_id[pilot_all_exome_driver_muts_unique$impact != "Synonymous"],1,2), pilot_all_exome_driver_muts_unique$gene[pilot_all_exome_driver_muts_unique$impact != "Synonymous"]))

kable(combined_muts[pilot_exome_drivers], col.names = c("Gene","Mutation count"), caption = "Total unique per donor non-synonymous mutations detected in combined targeted and exome data from pilot cohort")  
 
 Total unique per donor non-synonymous mutations detected in
combined targeted and exome data from pilot cohort 
 
 
 Gene 
 Mutation count 
 
 
 
 
 TNFRSF14 
 229 
 
 
 CD274 
 125 
 
 
 TET2 
 84 
 
 
 TNFAIP3 
 48 
 
 
 
  intersect_muts <- table(pilot_all_exome_driver_muts_unique$gene[
  pilot_all_exome_driver_muts_unique$mut_site %in%                                           
    intersect(pilot_all_exome_driver_muts[grepl("targeted",pilot_all_exome_driver_muts$sampleID) & pilot_all_exome_driver_muts$impact != "Synonymous",]$mut_site,
              pilot_all_exome_driver_muts[grepl("exome",pilot_all_exome_driver_muts$sampleID) & pilot_all_exome_driver_muts$impact != "Synonymous",]$mut_site)])
kable(intersect_muts[pilot_exome_drivers], col.names = c("Gene","Mutation count"), caption = "Non-synonymous mutations present in both targeted and exome pilot data")  
 
 Non-synonymous mutations present in both targeted and exome
pilot data 
 
 
 Gene 
 Mutation count 
 
 
 
 
 TNFRSF14 
 90 
 
 
 CD274 
 34 
 
 
 TET2 
 11 
 
 
 TNFAIP3 
 8 
 
 
 
  targeted_only_muts <- table(pilot_all_exome_driver_muts_unique$gene[
  pilot_all_exome_driver_muts_unique$mut_site %in%                                           
    setdiff(pilot_all_exome_driver_muts[grepl("targeted",pilot_all_exome_driver_muts$sampleID) & pilot_all_exome_driver_muts$impact != "Synonymous",]$mut_site,
            pilot_all_exome_driver_muts[grepl("exome",pilot_all_exome_driver_muts$sampleID) & pilot_all_exome_driver_muts$impact != "Synonymous",]$mut_site)])
kable(targeted_only_muts[pilot_exome_drivers], col.names = c("Gene","Mutation count"), caption = "Non-synonymous mutations present only in targeted data in pilot cohort")  
 
 Non-synonymous mutations present only in targeted data in pilot
cohort 
 
 
 Gene 
 Mutation count 
 
 
 
 
 TNFRSF14 
 127 
 
 
 CD274 
 85 
 
 
 TET2 
 66 
 
 
 TNFAIP3 
 37 
 
 
 
  exome_only_muts <- table(pilot_all_exome_driver_muts_unique$gene[
  pilot_all_exome_driver_muts_unique$mut_site %in%                                           
    setdiff(pilot_all_exome_driver_muts[grepl("exome",pilot_all_exome_driver_muts$sampleID) & pilot_all_exome_driver_muts$impact != "Synonymous",]$mut_site,
            pilot_all_exome_driver_muts[grepl("targeted",pilot_all_exome_driver_muts$sampleID) & pilot_all_exome_driver_muts$impact != "Synonymous",]$mut_site)])
kable(exome_only_muts[pilot_exome_drivers], col.names = c("Gene","Mutation count"), caption = "Non-synonymous mutations present only in exome data in pilot cohort")  
 
 Non-synonymous mutations present only in exome data in pilot
cohort 
 
 
 Gene 
 Mutation count 
 
 
 
 
 TNFRSF14 
 12 
 
 
 CD274 
 6 
 
 
 TET2 
 7 
 
 
 TNFAIP3 
 3 
 
 
 
  kable((targeted_only_muts[pilot_exome_drivers] / combined_muts[pilot_exome_drivers]) * 100, col.names = c("Gene","Mutation count"), caption = "Percentage of non-synonymous mutations seen only in targeted data (~10x deeper than exome data) from pilot cohort", digits = 1)  
 
 Percentage of non-synonymous mutations seen only in targeted
data (~10x deeper than exome data) from pilot cohort 
 
 
 Gene 
 Mutation count 
 
 
 
 
 TNFRSF14 
 55.5 
 
 
 CD274 
 68.0 
 
 
 TET2 
 78.6 
 
 
 TNFAIP3 
 77.1 
 
 
 
  if (runman) { dev.new() }

samples2plot <- pilot_all
genes2plot <- pilot_exome_drivers

muts2plot <- mutations[which(mutations$gene %in% genes2plot & mutations$sampleID %in% samples2plot),]
uniq2plot <- muts2plot[which(!(duplicated(muts2plot$mut_site))),]
donors2plot <- unique(substr(muts2plot$paper_id,1,2))

plot_rows <- 3
plot_cols <- length(genes2plot)

layout(matrix(c(1:(plot_rows*plot_cols)), nrow = plot_rows, ncol = plot_cols, byrow = F), widths = rep(4,times = length(genes2plot)))
par(mar = c(5,5.5,2,2))

nmut_array <- table(substr(uniq2plot$paper_id,1,2),
                    uniq2plot$impact,
                    uniq2plot$gene)

genes2plot = names(sort(apply(nmut_array, MARGIN = c(3), sum),decreasing=T)) # Sorting the genes in decreasing order of their number of mutations

nmuts_cap <- ceiling(max(apply(nmut_array, MARGIN = c(1,3), sum)) / 10) * 10

# Calculate cell fractions
cellfraction = array(NA, dim = c(length(donors2plot),8,length(genes2plot)), dimnames = list(donors2plot, c("mis","non","spl","ind","mislow","nonlow","spllow","indlow"),genes2plot))

for(x in 1:length(genes2plot)) {  
  for (j in 1:length(donors2plot)) {
    aux = muts2plot[which(muts2plot$gene==genes2plot[x] & substr(muts2plot$paper_id,1,2) == donors2plot[j]),c("sampleID","paper_id","impact","cellfraction","duplex_vaf")]
    per_sample_gene_cov <- coverage[which(coverage$gene == genes2plot[x]),colnames(coverage) %in% samples2plot[samples2plot %in% muts2plot$sampleID[substr(muts2plot$paper_id,1,2) == donors2plot[j]]]]
    aux$sample_gene_cov <- NA
    aux$cov_weighting <- NA
    for(k in 1:length(per_sample_gene_cov)){
      aux$sample_gene_cov[which(aux$sampleID == names(per_sample_gene_cov)[k])] <- as.numeric(per_sample_gene_cov[k])
    }
    aux$cov_weighting <- aux$sample_gene_cov / sum(per_sample_gene_cov)
    
    aux$weighted_cellfraction <- aux$cellfraction * aux$cov_weighting
    aux$weighted_duplex_vaf <- aux$duplex_vaf * aux$cov_weighting
    
    cellfraction[j,c("mis","mislow"),x] = colSums(aux[aux$impact %in% c("Missense","Start_loss","Stop_loss"),c("weighted_cellfraction","weighted_duplex_vaf")], na.rm = T)
    cellfraction[j,c("non","nonlow"),x] = colSums(aux[aux$impact=="Nonsense",c("weighted_cellfraction","weighted_duplex_vaf")], na.rm = T)
    cellfraction[j,c("spl","spllow"),x] = colSums(aux[aux$impact=="Essential_Splice",c("weighted_cellfraction","weighted_duplex_vaf")], na.rm = T)
    cellfraction[j,c("ind","indlow"),x] = colSums(aux[aux$impact=="no-SNV",c("weighted_cellfraction","weighted_duplex_vaf")], na.rm = T)
  }
}

# Object to output median adjusted bam VAF per gene across donors
median_bam_vaf_adj <- vector(mode = "numeric",length = length(genes2plot))
names(median_bam_vaf_adj) <- genes2plot
max_bam_vaf_adj <- median_bam_vaf_adj
below_1_bam_vaf_adj <- median_bam_vaf_adj

# Create three plots per donor: number of mutations, per mutation (mean across samples) unbiased VAF and mutant cell fraction
for(x in 1:length(genes2plot)){
  nmuts <- nmut_array[,,genes2plot[x]]
  subs_per_donor = t(nmuts)
  subs_per_donor = subs_per_donor[c("Synonymous","Missense","Start_loss","Stop_loss","Nonsense","Essential_Splice","no-SNV"),]
  rownames(subs_per_donor)[rownames(subs_per_donor) == "Essential_Splice"] <- "Splice"
  rownames(subs_per_donor)[rownames(subs_per_donor) == "no-SNV"] <- "Indels"
  
  colvec = c("Synonymous"="grey70","Missense"="cadetblue","Start_loss"="firebrick","Stop_loss"="red","Nonsense"="darkorchid4","Splice"="darkorchid2","Indels"="chocolate3")
  
  pos = barplot(subs_per_donor, las = 1, col=colvec, border=NA, ylim=c(0,nmuts_cap), ylab="Mutations per donor", main = genes2plot[x])
  
  if(x == 1){
    legend("topright",y=max(apply(subs_per_donor,2,sum))*1.09,legend=rownames(subs_per_donor),fill=colvec,border=NA,box.col=NA,bg="transparent")
  }
  
  # Calculate the mean unbiased VAF for mutations detected in multiple sequencing libraries from a donor
  # This avoids representing the same mutation multiple times in the scatter plot
  uniq2plot$mean_bam_vaf_adj <- NA
  for(y in 1:nrow(uniq2plot)){
    uniq2plot$mean_bam_vaf_adj[y] <- mean(pilot_all_exome_driver_muts$bam_vaf_adj[pilot_all_exome_driver_muts$mut_site == uniq2plot$mut_site[y]])
  }
  
  uniq2plot_cap <- 1.1*max(uniq2plot$mean_bam_vaf_adj)
  
  df_plot <- uniq2plot[uniq2plot$gene == genes2plot[x],]
  df_plot <- df_plot[order(df_plot$paper_id,decreasing = F),]
  df_plot$idx <- jitter(pos[as.numeric(substr(df_plot$paper_id,2,2))])
  df_plot$impact[df_plot$impact == "Essential_Splice"] <- "Splice"
  df_plot$impact[df_plot$impact == "no-SNV"] <- "Indels"
  df_plot$colour <- colvec[df_plot$impact]
  
  plot(df_plot$idx,df_plot$mean_bam_vaf_adj,col=df_plot$colour,xlab=NA, 
       xlim=c(0.2,3.6), ylim=c(0,uniq2plot_cap),ylab = "Unbiased VAF", bg="transparent", pch = 19, cex = 2, frame.plot = FALSE, axes = F)
  axis(side = 2, las = 1)
  axis(side = 1, at=pos, labels=unique(substr(df_plot$paper_id,1,2)), tick = F, pos = NA)
  
  for(y in 1:length(pos)){
    segments(x0 = pos[y] - 0.5, x1 = pos[y] + 0.5, 
             y0=median(df_plot$mean_bam_vaf_adj[which(substr(df_plot$paper_id,2,2) == y)]), 
             y1 = median(df_plot$mean_bam_vaf_adj[which(substr(df_plot$paper_id,2,2) == y)]), 
             lty = 2, lwd = 1.5)
  }
  
  median_bam_vaf_adj[x] <- median(df_plot$mean_bam_vaf_adj[df_plot$impact != "Synonymous"])
  max_bam_vaf_adj[x] <- max(df_plot$mean_bam_vaf_adj[df_plot$impact != "Synonymous"])
  below_1_bam_vaf_adj[x] <- 100 * length(which(df_plot$mean_bam_vaf_adj[df_plot$impact != "Synonymous"] < 0.01)) / length(df_plot$mean_bam_vaf_adj[df_plot$impact != "Synonymous"])
  
  nonsyndens_cap <- ceiling(max(apply(cellfraction[,1:4,] * 100,3,rowSums)) / 10) * 10
  
  nonsyndens = cellfraction[,,x] * 100
  nonsyndens_bounds = cbind(up = rowSums(nonsyndens[,1:4]), low=rowSums(nonsyndens[,5:8]))
  aux = t(cbind(nonsyndens_bounds[,2],nonsyndens_bounds[,1]-nonsyndens_bounds[,2]))
  
  # This allows a bar to be visible when MCF is zero.
  aux[which(aux == 0)] <- 0.001
  
  pos = barplot(aux[2,], col=c("indianred3"), border=c("indianred3"), ylab="% cells non-synonymous mutation", ylim=c(0,nonsyndens_cap), offset = aux[1,], las = 1)
}  
    
  # Number of non-synonymous mutations per donor across initial 4 driver genes in combined pilot exome and targeted data
kable(table(substr(pilot_all_exome_driver_muts_unique$paper_id[pilot_all_exome_driver_muts_unique$impact != "Synonymous"],1,2), pilot_all_exome_driver_muts_unique$gene[pilot_all_exome_driver_muts_unique$impact != "Synonymous"])[,pilot_exome_drivers], caption = "Number of non-synonymous mutations per donor across initial 4 driver genes in combined pilot exome and targeted data")  
 
 Number of non-synonymous mutations per donor across initial 4
driver genes in combined pilot exome and targeted data 
 
 
  
 TNFRSF14 
 CD274 
 TET2 
 TNFAIP3 
 
 
 
 
 H1 
 135 
 59 
 18 
 21 
 
 
 H2 
 45 
 56 
 36 
 10 
 
 
 H3 
 49 
 10 
 30 
 17 
 
 
 
  # Median unbiased VAF of non-synonymous mutations across donors (exome and targeted combined from pilot cohort)
kable(median_bam_vaf_adj, digits = 5, col.names = c("Gene","Median unbiased VAF"), caption = "Median unbiased VAF of non-synonymous mutations across donors (exome and targeted combined from pilot cohort)")  
 
 Median unbiased VAF of non-synonymous mutations across donors
(exome and targeted combined from pilot cohort) 
 
 
 Gene 
 Median unbiased VAF 
 
 
 
 
 TNFRSF14 
 0.00077 
 
 
 CD274 
 0.00028 
 
 
 TET2 
 0.00013 
 
 
 TNFAIP3 
 0.00019 
 
 
 
  # Maximum unbiased VAF of non-synonymous mutations across donors (exome and targeted combined from pilot cohort)
kable(max_bam_vaf_adj, digits = 5, col.names = c("Gene","Maximum unbiased VAF"), caption = "Maximum unbiased VAF of non-synonymous mutations across donors (exome and targeted combined from pilot cohort)")  
 
 Maximum unbiased VAF of non-synonymous mutations across donors
(exome and targeted combined from pilot cohort) 
 
 
 Gene 
 Maximum unbiased VAF 
 
 
 
 
 TNFRSF14 
 0.02831 
 
 
 CD274 
 0.02076 
 
 
 TET2 
 0.02349 
 
 
 TNFAIP3 
 0.00274 
 
 
 
  # Percentage of non-synonymous mutations with unbiased VAF below 1% (pilot exome and targeted combined)
kable(below_1_bam_vaf_adj, digits = 1, col.names = c("Gene","% muts"), caption = "Percentage of non-synonymous mutations with unbiased VAF below 1% (exome and targeted combined from pilot cohort)")  
 
 Percentage of non-synonymous mutations with unbiased VAF below
1% (exome and targeted combined from pilot cohort) 
 
 
 Gene 
 % muts 
 
 
 
 
 TNFRSF14 
 95.2 
 
 
 CD274 
 99.2 
 
 
 TET2 
 98.8 
 
 
 TNFAIP3 
 100.0 
 
 
 
  # Per donor aggregated mutant cell fractions (pilot exome and targeted combined)
kable(as.data.frame(colSums(aperm(cellfraction[,5:8,], c(2,1,3))) * 100), caption = "Per donor aggregated mutant cell fractions (exome and targeted combined from pilot cohort)", digits = 2)  
 
 Per donor aggregated mutant cell fractions (exome and targeted
combined from pilot cohort) 
 
 
  
 TNFRSF14 
 CD274 
 TET2 
 TNFAIP3 
 
 
 
 
 H1 
 32.55 
 3.91 
 2.50 
 0.63 
 
 
 H2 
 3.36 
 4.61 
 0.22 
 0.09 
 
 
 H3 
 4.61 
 0.14 
 0.95 
 0.55 
 
 
 
  # Calculate sum of times called across all samples from a donor for each unique mutation
pilot_all_exome_driver_muts_unique$sum_times_called <- NA
for(x in 1:nrow(pilot_all_exome_driver_muts_unique)){
  pilot_all_exome_driver_muts_unique$sum_times_called[x] <- sum(pilot_all_exome_driver_muts$times_called[pilot_all_exome_driver_muts$mut_site == pilot_all_exome_driver_muts_unique$mut_site[x]])
}

kable(table(pilot_all_exome_driver_muts_unique$gene[pilot_all_exome_driver_muts_unique$sum_times_called == 1 & pilot_all_exome_driver_muts_unique$impact != "Synonymous"])[pilot_exome_drivers], col.names = c("Gene","Mutation count"), caption = "Mutations (unique per donor) detected in only a single read bundle in combined exome and targeted data from pilot cohort")  
 
 Mutations (unique per donor) detected in only a single read
bundle in combined exome and targeted data from pilot cohort 
 
 
 Gene 
 Mutation count 
 
 
 
 
 TNFRSF14 
 77 
 
 
 CD274 
 48 
 
 
 TET2 
 63 
 
 
 TNFAIP3 
 28 
 
 
 
  kable(table(pilot_all_exome_driver_muts_unique$gene[pilot_all_exome_driver_muts_unique$sum_times_called > 1 & pilot_all_exome_driver_muts_unique$impact != "Synonymous"])[pilot_exome_drivers], col.names = c("Gene","Mutation count"), caption = "Mutations (unique per donor) detected in multiple read bundles in combined exome and targeted data from pilot cohort")  
 
 Mutations (unique per donor) detected in multiple read bundles
in combined exome and targeted data from pilot cohort 
 
 
 Gene 
 Mutation count 
 
 
 
 
 TNFRSF14 
 152 
 
 
 CD274 
 77 
 
 
 TET2 
 21 
 
 
 TNFAIP3 
 20 
 
 
 
  kable(table(pilot_all_exome_driver_muts_unique$gene[pilot_all_exome_driver_muts_unique$sum_times_called == 1 & pilot_all_exome_driver_muts_unique$impact != "Synonymous"])[pilot_exome_drivers] / table(pilot_all_exome_driver_muts_unique$gene[ pilot_all_exome_driver_muts_unique$impact != "Synonymous"])[pilot_exome_drivers] * 100, col.names = c("Gene","%"), caption = "% mutations (unique per donor) detected in only a single read bundle in combined exome and targeted data from pilot cohort", digits = 1)  
 
 % mutations (unique per donor) detected in only a single read
bundle in combined exome and targeted data from pilot cohort 
 
 
 Gene 
 % 
 
 
 
 
 TNFRSF14 
 33.6 
 
 
 CD274 
 38.4 
 
 
 TET2 
 75.0 
 
 
 TNFAIP3 
 58.3 
 
 
 
 
 
 7. Coverage metrics for extension and pilot cohort combined 
  # Duplex coverage for Exome NanoSeq of LCM samples from extension cohort
cat(paste0("Cumulative exome NanoSeq duplex coverage in the extension cohort LCM samples = ", sum(metadata[which(metadata$sample %in% extension_LCM & metadata$sequencing_type == "Exome"),]$median_duplex_cov), " dx"))  
  ## Cumulative exome NanoSeq duplex coverage in the extension cohort LCM samples = 1956 dx  
  # Targeted NanoSeq duplex coverage for extension cohort
cat(paste0("Targeted NanoSeq duplex coverage in the extension cohort = ", sum(metadata[which(metadata$sequencing_type == "Targeted" & metadata$sample %in% extension_all),]$median_duplex_cov), " dx"))  
  ## Targeted NanoSeq duplex coverage in the extension cohort = 43393 dx  
  # Combined targeted NanoSeq duplex coverage for extension and targeted cohort
cat(paste0("Cumulative targeted NanoSeq duplex coverage in the pilot and extension cohort combined = ", sum(metadata[which(metadata$sequencing_type == "Targeted"),]$median_duplex_cov), " dx"))  
  ## Cumulative targeted NanoSeq duplex coverage in the pilot and extension cohort combined = 72977 dx  
 
 
 8. dNdSshm for combined exome and targeted data in pilot and
extension cohorts 
  # dNdS results for exome data only from pilot and extension cohorts - used for annotating exome only dN/dS q-values in Fig. 2
sample_list <- metadata$sample[metadata$sequencing_type == "Exome"]

dnds_muts <- mutations[which(mutations$sampleID %in% sample_list),]
dnds_muts <- dnds_muts[which(!(duplicated(dnds_muts$mut_site))),]

dnds_cov <- rowSums(coverage[which(colnames(coverage) %in% sample_list)])
names(dnds_cov) <- coverage$gene
dnds_cov <- dnds_cov[which(dnds_cov != 0)]

# 2024-02-19 - Made decision to exclude IGLL5 and IGLL1 from dNdS analysis. Only reason these Ig genes (but not others) are in the dNdS object is they are in frame.
dnds_cov <- dnds_cov[which(!(names(dnds_cov) %in% c("IGLL5","IGLL1")))]

exome_only_dnds_shm <- dnds_shm(mutations = dnds_muts[,c("sampleID","chr","pos","ref","mut")],
                                refdb_noshm = RefCDS_noshm_exome,
                                refdb_shm = RefCDS_shm_exome,
                                dc_noshm = dnds_cov,
                                gene_list = names(dnds_cov),
                                # Exons with just the start codon need to be excluded (synonymous mutations are not possible for dNdSloc)
                                excl_shm = c("IGLL1","IGLL5","MYO1E","ZNF595","AC026202.1","EHBP1L1"),
                                excl_noshm = c("IGLL1","IGLL5"),
                                onesided = T,
                                maxcovs = 10)  
  # dNdS results for targeted data only from pilot and extension cohorts - used for annotating targeted only dN/dS q-values in Fig. 2
sample_list <- metadata$sample[metadata$sequencing_type == "Targeted"]

dnds_muts <- mutations[which(mutations$sampleID %in% sample_list),]
dnds_muts <- dnds_muts[which(!(duplicated(dnds_muts$mut_site))),]

dnds_cov <- rowSums(coverage[which(colnames(coverage) %in% sample_list)])
names(dnds_cov) <- coverage$gene
dnds_cov <- dnds_cov[which(dnds_cov != 0)]

# 2024-02-19 - Made decision to exclude IGLL5 and IGLL1 from dNdS analysis. Only reason these Ig genes (but not others) are in the dNdS object is they are in frame.
dnds_cov <- dnds_cov[which(!(names(dnds_cov) %in% c("IGLL5","IGLL1")))]

# Restrict dN/dS analysis to genes in targeted panel
dnds_cov <- dnds_cov[which(names(dnds_cov) %in% immune_genes$gene[which(immune_genes$target_type != "Hotspot")])]

targeted_only_dnds_shm <- dnds_shm(mutations = dnds_muts[,c("sampleID","chr","pos","ref","mut")],
                                   refdb_noshm = RefCDS_noshm_exome,
                                   refdb_shm = RefCDS_shm_exome,
                                   dc_noshm = dnds_cov,
                                   gene_list = names(dnds_cov),
                                   # Exons with just the start codon need to be excluded (synonymous mutations are not possible for dNdSloc)
                                   excl_shm = c("IGLL1","IGLL5","MYO1E","ZNF595","AC026202.1","EHBP1L1"),
                                   excl_noshm = c("IGLL1","IGLL5"),
                                   onesided = T,
                                   maxcovs = 10)  
  # dNdS results for combined exome and targeted data in pilot and extension cohorts (restricted to genes in targeted panel)
sample_list <- metadata$sample

dnds_muts <- mutations[which(mutations$sampleID %in% sample_list),]
dnds_muts <- dnds_muts[which(!(duplicated(dnds_muts$mut_site))),]

dnds_cov <- rowSums(coverage[which(colnames(coverage) %in% sample_list)])
names(dnds_cov) <- coverage$gene
dnds_cov <- dnds_cov[which(dnds_cov != 0)]

# 2024-02-19 - Made decision to exclude IGLL5 and IGLL1 from dNdS analysis. Only reason these Ig genes (but not others) are in the dNdS object is they are in frame.
dnds_cov <- dnds_cov[which(!(names(dnds_cov) %in% c("IGLL5","IGLL1")))]

# Restrict dN/dS analysis to genes in targeted panel
dnds_cov <- dnds_cov[which(names(dnds_cov) %in% immune_genes$gene[which(immune_genes$target_type != "Hotspot")])]

all_dnds_shm <- dnds_shm(mutations = dnds_muts[,c("sampleID","chr","pos","ref","mut")],
                         refdb_noshm = RefCDS_noshm_exome,
                         refdb_shm = RefCDS_shm_exome,
                         dc_noshm = dnds_cov,
                         gene_list = names(dnds_cov),
                         # Exons with just the start codon need to be excluded (synonymous mutations are not possible for dNdSloc)
                         excl_shm = c("IGLL1","IGLL5","MYO1E","ZNF595","AC026202.1","EHBP1L1"),
                         excl_noshm = c("IGLL1","IGLL5"),
                         onesided = T,
                         maxcovs = 10)  
  dnds_ratios <- all_dnds_shm$sel_merged[all_dnds_shm$sel_merged$qglobalpos_m < 0.1,c("gene_name","wmis_cv","wnon_cv","wspl_cv","wind_cv","wmis_loc","wnon_loc","wspl_loc","qglobalpos_m")]
dnds_ratios$qglobalpos_m <- format(dnds_ratios$qglobalpos_m, digits = 3)
for(j in c("wmis_loc","wnon_loc","wspl_loc")){
  dnds_ratios[which(dnds_ratios[,j] > 10000),j] <- Inf
}
kable(dnds_ratios, caption = "Significant genes identified from combined pilot and extension cohorts", col.names = c("Gene", "dN/dS missense (selcv nonSHM)", "dN/dS nonsense (selcv nonSHM)", "dN/dS splice (selcv nonSHM)","dN/dS indels (selcv nonSHM)","dN/dS missense (selloc SHM)","dN/dS nonsense (selloc SHM)","dN/dS splice (selloc SHM)","Q-value (pos merged)"), digits = 1, row.names = F)  
 
 Significant genes identified from combined pilot and extension
cohorts 
 
 
 
 
 
 
 
 
 
 
 
 
 
 Gene 
 dN/dS missense (selcv nonSHM) 
 dN/dS nonsense (selcv nonSHM) 
 dN/dS splice (selcv nonSHM) 
 dN/dS indels (selcv nonSHM) 
 dN/dS missense (selloc SHM) 
 dN/dS nonsense (selloc SHM) 
 dN/dS splice (selloc SHM) 
 Q-value (pos merged) 
 
 
 
 
 CD274 
 6.7 
 36.6 
 36.6 
 34.3 
 NA 
 NA 
 NA 
 0.00e+00 
 
 
 LTB 
 NA 
 NA 
 NA 
 NA 
 1.2 
 5.3 
 5.3 
 0.00e+00 
 
 
 TNFRSF14 
 16.7 
 141.1 
 141.1 
 43.3 
 NA 
 NA 
 NA 
 0.00e+00 
 
 
 TNFAIP3 
 1.2 
 12.3 
 12.3 
 6.2 
 NA 
 NA 
 NA 
 1.95e-14 
 
 
 TG 
 1.2 
 0.9 
 0.9 
 12.4 
 1.3 
 0.0 
 0.0 
 9.33e-09 
 
 
 TET2 
 1.3 
 5.1 
 5.1 
 4.3 
 NA 
 NA 
 NA 
 1.64e-08 
 
 
 IRF1 
 4.4 
 15.3 
 15.3 
 3.7 
 NA 
 NA 
 NA 
 2.57e-08 
 
 
 DNMT3A 
 2.3 
 8.0 
 8.0 
 2.8 
 NA 
 NA 
 NA 
 1.55e-06 
 
 
 CCR6 
 0.6 
 10.9 
 10.9 
 1.8 
 NA 
 NA 
 NA 
 5.58e-06 
 
 
 SOCS1 
 3.3 
 8.0 
 8.0 
 6.0 
 NA 
 NA 
 NA 
 1.56e-05 
 
 
 DUSP2 
 NA 
 NA 
 NA 
 NA 
 0.9 
 4.0 
 4.0 
 1.79e-05 
 
 
 GNA13 
 3.4 
 4.1 
 4.1 
 1.5 
 NA 
 NA 
 NA 
 7.73e-04 
 
 
 KLHL6 
 2.0 
 0.0 
 0.0 
 1.5 
 3.9 
 2.8 
 2.8 
 1.24e-03 
 
 
 RFTN1 
 1.2 
 2.8 
 2.8 
 0.5 
 1.0 
 3.8 
 3.8 
 3.35e-03 
 
 
 LYN 
 1.5 
 4.8 
 4.8 
 4.2 
 NA 
 NA 
 NA 
 8.98e-03 
 
 
 CBL 
 2.8 
 1.5 
 1.5 
 0.8 
 NA 
 NA 
 NA 
 1.22e-02 
 
 
 PTPRC 
 1.2 
 3.3 
 3.3 
 1.8 
 NA 
 NA 
 NA 
 1.74e-02 
 
 
 ARID2 
 1.0 
 3.1 
 3.1 
 1.9 
 NA 
 NA 
 NA 
 1.98e-02 
 
 
 ACTG1 
 2.4 
 4.0 
 4.0 
 0.8 
 1.9 
 2.1 
 2.1 
 2.78e-02 
 
 
 SBF1 
 1.2 
 3.8 
 3.8 
 2.6 
 NA 
 NA 
 NA 
 3.67e-02 
 
 
 RASA2 
 1.3 
 4.7 
 4.7 
 1.7 
 NA 
 NA 
 NA 
 3.67e-02 
 
 
 GRB2 
 4.7 
 2.6 
 2.6 
 1.2 
 NA 
 NA 
 NA 
 3.67e-02 
 
 
 EEF1A1 
 3.2 
 2.5 
 2.5 
 2.7 
 Inf 
 0.0 
 0.0 
 3.86e-02 
 
 
 NFKBIA 
 1.4 
 5.8 
 5.8 
 3.0 
 NA 
 NA 
 NA 
 4.41e-02 
 
 
 ZEB2 
 1.8 
 3.9 
 3.9 
 1.3 
 NA 
 NA 
 NA 
 4.55e-02 
 
 
 TNIP1 
 0.6 
 7.5 
 7.5 
 1.9 
 NA 
 NA 
 NA 
 4.55e-02 
 
 
 KRAS 
 3.4 
 0.0 
 0.0 
 3.5 
 NA 
 NA 
 NA 
 4.55e-02 
 
 
 MAP4K1 
 1.2 
 4.1 
 4.1 
 3.1 
 NA 
 NA 
 NA 
 5.89e-02 
 
 
 SH2B3 
 1.4 
 8.0 
 8.0 
 1.6 
 NA 
 NA 
 NA 
 6.59e-02 
 
 
 PTEN 
 1.6 
 2.2 
 2.2 
 5.3 
 NA 
 NA 
 NA 
 9.37e-02 
 
 
 IRS4 
 1.8 
 2.9 
 2.9 
 1.7 
 NA 
 NA 
 NA 
 9.68e-02 
 
 
 
  # Significant genes in combined targeted and exome data across index and extension cohorts
all_shm_merged <- all_dnds_shm$sel_merged
all_shm_merged <- all_shm_merged[order(all_shm_merged$wnon_cv,decreasing = T),]
all_shm_merged <- all_shm_merged[order(all_shm_merged$qglobalpos_m,decreasing = F),]

all_drivers <- all_shm_merged$gene_name[all_shm_merged$qglobalpos_m < 0.1]

# Total number of non-synonymous mutations across driver genes
all_driver_muts <- mutations[mutations$gene %in% all_drivers,]
all_driver_muts_unique <- all_driver_muts[!(duplicated(all_driver_muts$mut_site)),]

all_driver_counts <- sort(colSums(table(substr(all_driver_muts_unique$paper_id[all_driver_muts_unique$impact != "Synonymous"],1,2), all_driver_muts_unique$gene[all_driver_muts_unique$impact != "Synonymous"])), decreasing = T)

kable(all_driver_counts, col.names = c("Gene","Mutation count"), caption = "Total non-synonymous mutations (unique per donor) across driver genes in combined pilot and extension cohorts")  
 
 Total non-synonymous mutations (unique per donor) across driver
genes in combined pilot and extension cohorts 
 
 
 Gene 
 Mutation count 
 
 
 
 
 TG 
 463 
 
 
 TNFRSF14 
 269 
 
 
 LTB 
 265 
 
 
 ACTG1 
 201 
 
 
 CD274 
 201 
 
 
 DUSP2 
 173 
 
 
 TET2 
 169 
 
 
 RFTN1 
 154 
 
 
 KLHL6 
 126 
 
 
 IRS4 
 114 
 
 
 ARID2 
 107 
 
 
 PTPRC 
 100 
 
 
 ZEB2 
 93 
 
 
 TNFAIP3 
 86 
 
 
 CBL 
 77 
 
 
 SBF1 
 75 
 
 
 DNMT3A 
 70 
 
 
 GNA13 
 55 
 
 
 SOCS1 
 45 
 
 
 LYN 
 43 
 
 
 RASA2 
 42 
 
 
 IRF1 
 39 
 
 
 MAP4K1 
 37 
 
 
 EEF1A1 
 35 
 
 
 CCR6 
 32 
 
 
 PTEN 
 32 
 
 
 NFKBIA 
 30 
 
 
 SH2B3 
 29 
 
 
 KRAS 
 21 
 
 
 GRB2 
 19 
 
 
 TNIP1 
 15 
 
 
 
  # Number of non-synonymous mutations per donor across driver genes in combined targeted and exome data across index and extension cohorts
kable(table(substr(all_driver_muts_unique$paper_id[all_driver_muts_unique$impact != "Synonymous"],1,2), all_driver_muts_unique$gene[all_driver_muts_unique$impact != "Synonymous"])[,all_drivers])  
 
 
 
 
 
 
 
 
 
 
 
 
 
 
 
 
 
 
 
 
 
 
 
 
 
 
 
 
 
 
 
 
 
 
 
 
 
  
 TNFRSF14 
 CD274 
 LTB 
 TNFAIP3 
 TG 
 TET2 
 IRF1 
 DNMT3A 
 CCR6 
 SOCS1 
 DUSP2 
 GNA13 
 KLHL6 
 RFTN1 
 LYN 
 CBL 
 PTPRC 
 ARID2 
 ACTG1 
 RASA2 
 SBF1 
 GRB2 
 EEF1A1 
 NFKBIA 
 TNIP1 
 ZEB2 
 KRAS 
 MAP4K1 
 SH2B3 
 PTEN 
 IRS4 
 
 
 
 
 G1 
 5 
 7 
 4 
 0 
 5 
 1 
 0 
 0 
 0 
 0 
 3 
 1 
 1 
 2 
 0 
 2 
 3 
 1 
 2 
 0 
 1 
 2 
 0 
 1 
 0 
 1 
 0 
 0 
 3 
 0 
 1 
 
 
 G2 
 0 
 2 
 0 
 2 
 37 
 4 
 0 
 0 
 2 
 0 
 1 
 1 
 2 
 1 
 1 
 4 
 4 
 5 
 0 
 3 
 6 
 0 
 2 
 0 
 2 
 1 
 1 
 0 
 1 
 0 
 1 
 
 
 G3 
 1 
 7 
 7 
 4 
 53 
 12 
 3 
 7 
 0 
 3 
 7 
 2 
 11 
 4 
 3 
 5 
 5 
 7 
 7 
 1 
 8 
 2 
 0 
 2 
 1 
 3 
 1 
 0 
 3 
 5 
 5 
 
 
 G4 
 0 
 0 
 0 
 0 
 33 
 5 
 1 
 2 
 0 
 0 
 0 
 0 
 3 
 0 
 1 
 0 
 1 
 4 
 1 
 1 
 2 
 0 
 0 
 0 
 0 
 1 
 0 
 0 
 1 
 1 
 3 
 
 
 G5 
 7 
 2 
 1 
 7 
 20 
 9 
 2 
 3 
 2 
 5 
 3 
 4 
 4 
 3 
 2 
 3 
 2 
 2 
 0 
 1 
 2 
 2 
 2 
 2 
 0 
 1 
 0 
 2 
 0 
 0 
 3 
 
 
 H1 
 135 
 59 
 88 
 21 
 19 
 18 
 10 
 8 
 11 
 12 
 32 
 17 
 41 
 49 
 7 
 3 
 12 
 10 
 79 
 15 
 5 
 8 
 9 
 6 
 7 
 15 
 4 
 14 
 3 
 6 
 9 
 
 
 H2 
 45 
 56 
 53 
 10 
 28 
 36 
 0 
 9 
 5 
 7 
 30 
 9 
 13 
 37 
 6 
 10 
 17 
 12 
 34 
 5 
 15 
 1 
 1 
 1 
 1 
 25 
 3 
 1 
 2 
 5 
 26 
 
 
 H3 
 49 
 10 
 27 
 17 
 38 
 30 
 3 
 17 
 5 
 3 
 12 
 12 
 12 
 11 
 7 
 11 
 14 
 18 
 22 
 4 
 12 
 0 
 5 
 4 
 1 
 8 
 1 
 2 
 8 
 3 
 26 
 
 
 H4 
 0 
 0 
 8 
 2 
 27 
 6 
 0 
 4 
 0 
 0 
 4 
 1 
 3 
 4 
 0 
 2 
 5 
 9 
 2 
 0 
 5 
 0 
 1 
 1 
 0 
 5 
 0 
 1 
 0 
 0 
 9 
 
 
 H5 
 0 
 1 
 3 
 3 
 54 
 6 
 1 
 1 
 1 
 0 
 2 
 1 
 6 
 0 
 0 
 4 
 7 
 7 
 1 
 1 
 5 
 1 
 1 
 0 
 0 
 2 
 0 
 0 
 1 
 1 
 2 
 
 
 H6 
 1 
 6 
 1 
 0 
 47 
 6 
 2 
 2 
 3 
 1 
 2 
 0 
 3 
 0 
 1 
 3 
 5 
 8 
 1 
 1 
 4 
 0 
 0 
 1 
 1 
 5 
 0 
 3 
 0 
 1 
 5 
 
 
 H7 
 4 
 0 
 8 
 3 
 35 
 6 
 3 
 4 
 1 
 3 
 6 
 0 
 3 
 9 
 3 
 2 
 3 
 7 
 4 
 0 
 2 
 0 
 2 
 2 
 2 
 8 
 3 
 0 
 2 
 4 
 4 
 
 
 H8 
 15 
 41 
 55 
 12 
 29 
 16 
 6 
 4 
 0 
 11 
 47 
 5 
 18 
 26 
 11 
 25 
 10 
 10 
 44 
 8 
 4 
 2 
 7 
 7 
 0 
 9 
 6 
 13 
 2 
 4 
 13 
 
 
 H9 
 7 
 10 
 10 
 5 
 38 
 14 
 8 
 9 
 2 
 0 
 24 
 2 
 6 
 8 
 1 
 3 
 12 
 7 
 4 
 2 
 4 
 1 
 5 
 3 
 0 
 9 
 2 
 1 
 3 
 2 
 7 
 
 
 
  exome_no_shm_snv_ci <- dndscv::geneci(dndsout = exome_only_dnds_shm)
exome_no_shm_indel_ci <- geneindelci(geneindels = exome_only_dnds_shm$geneindels, theta = exome_only_dnds_shm$nbregind$theta)
exome_no_shm_gene_ci <- cbind(exome_no_shm_snv_ci,exome_no_shm_indel_ci[,c(2:ncol(exome_no_shm_indel_ci))])
exome_shm_gene_ci <- geneciloc(exome_only_dnds_shm$genemuts_shm)

targeted_no_shm_snv_ci <- dndscv::geneci(dndsout = targeted_only_dnds_shm)
targeted_no_shm_indel_ci <- geneindelci(geneindels = targeted_only_dnds_shm$geneindels, theta = targeted_only_dnds_shm$nbregind$theta)
targeted_no_shm_gene_ci <- cbind(targeted_no_shm_snv_ci,targeted_no_shm_indel_ci[,c(2:ncol(targeted_no_shm_indel_ci))])
targeted_shm_gene_ci <- geneciloc(targeted_only_dnds_shm$genemuts_shm)

all_no_shm_snv_ci <- dndscv::geneci(dndsout = all_dnds_shm)
all_no_shm_indel_ci <- geneindelci(geneindels = all_dnds_shm$geneindels, theta = all_dnds_shm$nbregind$theta)
all_no_shm_gene_ci <- cbind(all_no_shm_snv_ci,all_no_shm_indel_ci[,c(2:ncol(all_no_shm_indel_ci))])
all_shm_gene_ci <- geneciloc(all_dnds_shm$genemuts_shm)  
 
 Codondnds for combined exome and targeted data in pilot and
extension cohorts 
  load(RefCDS_noshm_only_codon)
gene_list_noshm = sapply(RefCDS_noshm_only_codon, function(x) x$gene_name)
gene_list_noshm = setdiff(gene_list_noshm, c("IGLL1","IGLL5",all_dnds_shm$sel_merged$gene_name[which(is.na(all_dnds_shm$sel_merged$pmis_cv))]))
gene_list_noshm = intersect(gene_list_noshm, names(dnds_cov))

dndsout_noshm_nocov = dndscv(mutations = unique(dnds_muts[,c("sampleID","chr","pos","ref","mut")]), gene_list = gene_list_noshm, max_muts_per_gene_per_sample = Inf, max_coding_muts_per_sample = Inf, onesided = T, refdb = RefCDS_noshm_exome, outmats = T)

## This runs codon dNdS only in exons marked as no SHM. Loses KLHL6 hotspots but otherwise more appropriate.
# Codondnds
RefCDS_noshm_only_codon_targeted <- RefCDS_noshm_only_codon[sapply(RefCDS_noshm_only_codon, function(x) x$gene_name) %in% immune_genes$gene[which(immune_genes$target_type != "Hotspot")]]
codondnds_noshm_out <- codondnds(dndsout_noshm_nocov, refcds = RefCDS_noshm_only_codon_targeted, gene_list = immune_genes$gene[which(immune_genes$target_type != "Hotspot")], method = "LNP")

rm(RefCDS_noshm_only_codon)
rm(RefCDS_noshm_only_codon_targeted)  
  kable(codondnds_noshm_out$recurcodons[which(codondnds_noshm_out$recurcodons$qval < 0.1),])  
 
 
 
 chr 
 gene 
 codon 
 freq 
 mu 
 dnds 
 pval 
 qval 
 
 
 
 
 1 
 TNFRSF14 
 M1 
 19 
 0.0358414 
 530.1133 
 0e+00 
 0.0001890 
 
 
 1 
 TNFRSF14 
 W12 
 9 
 0.0313808 
 286.8000 
 0e+00 
 0.0065060 
 
 
 12 
 FAM186A 
 Q1330 
 10 
 0.0368926 
 271.0570 
 0e+00 
 0.0065060 
 
 
 11 
 CBL 
 C404 
 6 
 0.0215240 
 278.7583 
 1e-07 
 0.0194669 
 
 
 19 
 MUC16 
 S11145 
 10 
 0.0569130 
 175.7066 
 2e-07 
 0.0301304 
 
 
 9 
 CD274 
 M1 
 9 
 0.0554505 
 162.3070 
 4e-07 
 0.0438622 
 
 
 1 
 TNFRSF14 
 C121 
 5 
 0.0222925 
 224.2904 
 7e-07 
 0.0578894 
 
 
 
 
 
 Sitednds for combined exome and targeted data in pilot and extension
cohorts 
  ## This runs site dNdS only in exons marked as no SHM. Loses KLHL6 hotspots but otherwise more appropriate.
# Sitednds
sitednds_noshm_out <- sitednds(dndsout_noshm_nocov, gene_list = gene_list_noshm, method = "LNP")  
  kable(sitednds_noshm_out$recursites[which(sitednds_noshm_out$recursites$qval < 0.1),])  
 
 
 
 
 
 
 
 
 
 
 
 
 
 
 
 
 
 
 
 chr 
 pos 
 ref 
 mut 
 gene 
 aachange 
 impact 
 ref3_cod 
 mut3_cod 
 freq 
 mu 
 dnds 
 pval 
 qval 
 
 
 
 
 12 
 50746626 
 T 
 G 
 FAM186A 
 Q1330P 
 Missense 
 CAG 
 CCG 
 10 
 0.0019330 
 5173.3231 
 0e+00 
 0.0000000 
 
 
 1 
 2488174 
 T 
 A 
 TNFRSF14 
 . 
 Essential_Splice 
 GTG 
 GAG 
 4 
 0.0017011 
 2351.4817 
 0e+00 
 0.0007658 
 
 
 1 
 2488174 
 T 
 C 
 TNFRSF14 
 . 
 Essential_Splice 
 GTG 
 GCG 
 4 
 0.0027402 
 1459.7440 
 0e+00 
 0.0032342 
 
 
 1 
 2488138 
 G 
 A 
 TNFRSF14 
 W12* 
 Nonsense 
 TGG 
 TAG 
 5 
 0.0062790 
 796.2999 
 0e+00 
 0.0041335 
 
 
 4 
 71347225 
 C 
 A 
 MUC7 
 P255Q 
 Missense 
 CCA 
 CAA 
 5 
 0.0071256 
 701.6958 
 0e+00 
 0.0057409 
 
 
 19 
 9048197 
 G 
 A 
 MUC16 
 S11145L 
 Missense 
 TCA 
 TTA 
 10 
 0.0297353 
 336.3009 
 0e+00 
 0.0079928 
 
 
 16 
 67683191 
 C 
 G 
 RLTPR 
 Q575E 
 Missense 
 CCA 
 CGA 
 3 
 0.0012907 
 2324.3458 
 0e+00 
 0.0079928 
 
 
 1 
 2488105 
 T 
 G 
 TNFRSF14 
 M1R 
 Missense 
 ATG 
 AGG 
 3 
 0.0015603 
 1922.6527 
 0e+00 
 0.0122858 
 
 
 1 
 2488105 
 T 
 C 
 TNFRSF14 
 M1T 
 Missense 
 ATG 
 ACG 
 4 
 0.0054488 
 734.1083 
 0e+00 
 0.0146195 
 
 
 19 
 9048643 
 C 
 T 
 MUC16 
 R10996R 
 Synonymous 
 GGG 
 GAG 
 6 
 0.0165123 
 363.3651 
 0e+00 
 0.0159438 
 
 
 1 
 2488174 
 T 
 G 
 TNFRSF14 
 . 
 Essential_Splice 
 GTG 
 GGG 
 3 
 0.0019760 
 1518.2529 
 0e+00 
 0.0159438 
 
 
 1 
 2488177 
 G 
 A 
 TNFRSF14 
 . 
 Essential_Splice 
 AGC 
 AAC 
 4 
 0.0061542 
 649.9636 
 0e+00 
 0.0159438 
 
 
 8 
 133883661 
 T 
 A 
 TG 
 S115T 
 Missense 
 CTC 
 CAC 
 3 
 0.0020156 
 1488.3936 
 0e+00 
 0.0159438 
 
 
 1 
 2488106 
 G 
 A 
 TNFRSF14 
 M1I 
 Missense 
 TGG 
 TAG 
 4 
 0.0062790 
 637.0399 
 0e+00 
 0.0159438 
 
 
 19 
 9048808 
 A 
 G 
 MUC16 
 S10941S 
 Synonymous 
 CTA 
 CCA 
 5 
 0.0117816 
 424.3905 
 0e+00 
 0.0160470 
 
 
 1 
 2489817 
 G 
 C 
 TNFRSF14 
 G72R 
 Missense 
 GGG 
 GCG 
 3 
 0.0022875 
 1311.4874 
 1e-07 
 0.0190612 
 
 
 1 
 2489171 
 T 
 G 
 TNFRSF14 
 Y26D 
 Missense 
 GTA 
 GGA 
 3 
 0.0025212 
 1189.9080 
 1e-07 
 0.0239046 
 
 
 1 
 2488139 
 G 
 A 
 TNFRSF14 
 W12* 
 Nonsense 
 GGA 
 GAA 
 4 
 0.0081228 
 492.4405 
 1e-07 
 0.0313824 
 
 
 1 
 2488171 
 T 
 C 
 TNFRSF14 
 L23P 
 Missense 
 CTG 
 CCG 
 3 
 0.0029151 
 1029.1313 
 1e-07 
 0.0313824 
 
 
 11 
 119148991 
 G 
 A 
 CBL 
 C404Y 
 Missense 
 TGT 
 TAT 
 4 
 0.0083147 
 481.0755 
 1e-07 
 0.0313824 
 
 
 9 
 5457165 
 C 
 T 
 CD274 
 Q47* 
 Nonsense 
 ACA 
 ATA 
 4 
 0.0106647 
 375.0702 
 3e-07 
 0.0735687 
 
 
 19 
 9048609 
 G 
 A 
 MUC16 
 L11008F 
 Missense 
 TCT 
 TTT 
 5 
 0.0198451 
 251.9509 
 4e-07 
 0.0905690 
 
 
 
 
 
 Driver discovery plot for pilot and extension cohorts combined 
  plotpanels = letters[c(1,8,9,10)]
plotwidth = length(all_drivers) / 5
plotheight = as.numeric(2.5 * length(plotpanels))

sig_shm_genes <- all_dnds_shm$sel_merged$gene_name[which((all_dnds_shm$sel_merged$qglobalpos_cv > 0.1 | is.na(all_dnds_shm$sel_merged$qglobalpos_cv)) & all_dnds_shm$sel_merged$qpos_loc < 0.1 & all_dnds_shm$sel_merged$gene_name %in% all_drivers)]
lineage_defining_genes <- "TG"

highlighted_genes <- list(setNames(object = c(rep("grey", times = length(sig_shm_genes)),rep("blue",times = length(lineage_defining_genes))), c(sig_shm_genes,lineage_defining_genes)))

driver_discovery = driverplot(
  genes2plot=list(all_drivers),
  exome_dnds_sel_cv = list(exome_only_dnds_shm$sel_merged),
  # exome_dnds_sel_cv_gene_ci = list(exome_no_shm_gene_ci),
  exome_dnds_loc = list(exome_only_dnds_shm$sel_merged),
  # exome_dnds_loc_gene_ci = list(exome_shm_gene_ci),
  targeted_dnds_sel_cv = list(targeted_only_dnds_shm$sel_merged),
  # targeted_dnds_sel_cv_gene_ci = list(targeted_no_shm_gene_ci),
  targeted_dnds_loc = list(targeted_only_dnds_shm$sel_merged),
  # targeted_dnds_loc_gene_ci = list(targeted_shm_gene_ci),
  combined_targeted_only_dnds_sel_cv = list(all_dnds_shm$sel_merged),
  # combined_targeted_only_dnds_sel_cv_gene_ci = list(all_no_shm_gene_ci),
  combined_targeted_only_dnds_loc = list(all_dnds_shm$sel_merged),
  # combined_targeted_only_dnds_loc_gene_ci = list(all_shm_gene_ci),
  driverdensity_sampleIDs = list(metadata$sample),
  full_sampleIDs = list(metadata$sample),
  muts=mutations,
  gene2dc_array = coverage, 
  min_syn_loc = 5,
  max_genes = Inf, 
  sortbyfreq = F,
  onlysignifdnds = T,
  logscale = T,
  logscale_dnds = F,
  dnds_plot_cap = 50,
  nmuts_cap = 550,
  nonsyn_mcf_cap = 12,
  max_duplex_cov = 160000,
  runman = runman,
  plotfilename = "./output/Fig2C_AllSamplesCombinedExomeTargetedDriverDiscovery.pdf",
  plotpanels = plotpanels,
  plottitles =  "All samples (exome and targeted combined)",
  highlighted_genes = highlighted_genes)  
    
 
 
 Driver discovery plot including 30 randomly selected genes not under
significant positive selection 
  # For randomly sampled genes, excluded those in driver list, those with SHM and those with 0 mutations
random_genes = sample(x = immune_genes$gene[which(!(immune_genes$gene %in% all_drivers) & (immune_genes$gene %in% all_dnds_shm$sel_merged$gene_name[which(is.na(all_dnds_shm$sel_merged$wmis_loc) & rowSums(all_dnds_shm$sel_merged[,c(2:6)]) > 0)]))],size = 30, replace = F)

# Order random genes by decreasing 
random_genes_cds <- immune_genes[which(immune_genes$gene %in% random_genes),c("gene","CDS_length")]
random_genes_cds <- random_genes_cds[order(random_genes_cds$CDS_length, decreasing = T),]

random_genes <- random_genes_cds$gene

plotpanels = letters[c(1,8,9,10)]
plotwidth = length(c(all_drivers,random_genes)) / 5
plotheight = as.numeric(2.5 * length(plotpanels))

sig_shm_genes <- all_dnds_shm$sel_merged$gene_name[which((all_dnds_shm$sel_merged$qglobalpos_cv > 0.1 | is.na(all_dnds_shm$sel_merged$qglobalpos_cv)) & all_dnds_shm$sel_merged$qpos_loc < 0.1 & all_dnds_shm$sel_merged$gene_name %in% all_drivers)]
lineage_defining_genes <- "TG"

highlighted_genes <- list(setNames(object = c(rep("grey", times = length(sig_shm_genes)),rep("blue",times = length(lineage_defining_genes))), c(sig_shm_genes,lineage_defining_genes)))

driver_discovery = driverplot(
  genes2plot=list(c(all_drivers,random_genes)),
  exome_dnds_sel_cv = list(exome_only_dnds_shm$sel_merged),
  exome_dnds_sel_cv_gene_ci = list(exome_no_shm_gene_ci),
  exome_dnds_loc = list(exome_only_dnds_shm$sel_merged),
  exome_dnds_loc_gene_ci = list(exome_shm_gene_ci),
  targeted_dnds_sel_cv = list(targeted_only_dnds_shm$sel_merged),
  targeted_dnds_sel_cv_gene_ci = list(targeted_no_shm_gene_ci),
  targeted_dnds_loc = list(targeted_only_dnds_shm$sel_merged),
  targeted_dnds_loc_gene_ci = list(targeted_shm_gene_ci),
  combined_targeted_only_dnds_sel_cv = list(all_dnds_shm$sel_merged),
  combined_targeted_only_dnds_sel_cv_gene_ci = list(all_no_shm_gene_ci),
  combined_targeted_only_dnds_loc = list(all_dnds_shm$sel_merged),
  combined_targeted_only_dnds_loc_gene_ci = list(all_shm_gene_ci),
  driverdensity_sampleIDs = list(metadata$sample),
  full_sampleIDs = list(metadata$sample),
  muts=mutations,
  gene2dc_array = coverage, 
  min_syn_loc = 5,
  max_genes = Inf, 
  sortbyfreq = F,
  onlysignifdnds = T,
  logscale = F,
  logscale_dnds = T,
  dnds_plot_cap = 50,
  nmuts_cap = 550,
  nonsyn_mcf_cap = 12,
  max_duplex_cov = 160000,
  runman = runman,
  plotfilename = "./output/DriverDiscoveryPlot_RandomGenes.pdf",
  plotpanels = plotpanels,
  plottitles =  paste0(length(all_drivers)," driver genes and ",length(random_genes)," random genes not under significant positive selection"),
  highlighted_genes = highlighted_genes)  
    
 
 
 Driver discovery from extension cohort only 
  # dNdS results for combined exome and targeted data fron extension cohort only (restricted to genes in targeted panel)
sample_list <- extension_all

dnds_muts <- mutations[which(mutations$sampleID %in% sample_list),]
dnds_muts <- dnds_muts[which(!(duplicated(dnds_muts$mut_site))),]

dnds_cov <- rowSums(coverage[which(colnames(coverage) %in% sample_list)])
names(dnds_cov) <- coverage$gene
dnds_cov <- dnds_cov[which(dnds_cov != 0)]

# 2024-02-19 - Made decision to exclude IGLL5 and IGLL1 from dNdS analysis. Only reason these Ig genes (but not others) are in the dNdS object is they are in frame.
dnds_cov <- dnds_cov[which(!(names(dnds_cov) %in% c("IGLL5","IGLL1")))]

# Restrict dN/dS analysis to genes in targeted panel
dnds_cov <- dnds_cov[which(names(dnds_cov) %in% immune_genes$gene[which(immune_genes$target_type != "Hotspot")])]

extension_only_dnds_shm <- dnds_shm(mutations = dnds_muts[,c("sampleID","chr","pos","ref","mut")],
                                    refdb_noshm = RefCDS_noshm_exome,
                                    refdb_shm = RefCDS_shm_exome,
                                    dc_noshm = dnds_cov,
                                    gene_list = names(dnds_cov),
                                    # Exons with just the start codon need to be excluded (synonymous mutations are not possible for dNdSloc)
                                    excl_shm = c("IGLL1","IGLL5","MYO1E","ZNF595","AC026202.1","EHBP1L1"),
                                    excl_noshm = c("IGLL1","IGLL5"),
                                    onesided = T,
                                    maxcovs = 10)  
  dnds_ratios <- extension_only_dnds_shm$sel_merged[extension_only_dnds_shm$sel_merged$qglobalpos_m < 0.1,c("gene_name","wmis_cv","wnon_cv","wspl_cv","wind_cv","wmis_loc","wnon_loc","wspl_loc","qglobalpos_m")]
dnds_ratios$qglobalpos_m <- format(dnds_ratios$qglobalpos_m, digits = 3)
kable(dnds_ratios, caption = "Significant genes identified from extension cohort only", col.names = c("Gene", "dN/dS missense (selcv nonSHM)", "dN/dS nonsense (selcv nonSHM)", "dN/dS splice (selcv nonSHM)","dN/dS indels (selcv nonSHM)","dN/dS missense (selloc SHM)","dN/dS nonsense (selloc SHM)","dN/dS splice (selloc SHM)","Q-value (pos merged)"), digits = 1, row.names = F)  
 
 Significant genes identified from extension cohort
only 
 
 
 
 
 
 
 
 
 
 
 
 
 
 Gene 
 dN/dS missense (selcv nonSHM) 
 dN/dS nonsense (selcv nonSHM) 
 dN/dS splice (selcv nonSHM) 
 dN/dS indels (selcv nonSHM) 
 dN/dS missense (selloc SHM) 
 dN/dS nonsense (selloc SHM) 
 dN/dS splice (selloc SHM) 
 Q-value (pos merged) 
 
 
 
 
 CD274 
 4.2 
 26.8 
 26.8 
 17.1 
 NA 
 NA 
 NA 
 0.00e+00 
 
 
 TNFRSF14 
 6.3 
 53.4 
 53.4 
 7.7 
 NA 
 NA 
 NA 
 0.00e+00 
 
 
 TG 
 1.5 
 0.9 
 0.9 
 14.4 
 0.8 
 0.0 
 0.0 
 3.17e-10 
 
 
 LTB 
 NA 
 NA 
 NA 
 NA 
 1.2 
 6.1 
 6.1 
 2.74e-06 
 
 
 IRF1 
 5.4 
 10.1 
 10.1 
 2.6 
 NA 
 NA 
 NA 
 4.72e-05 
 
 
 DUSP2 
 NA 
 NA 
 NA 
 NA 
 1.1 
 5.6 
 5.6 
 7.89e-05 
 
 
 TNFAIP3 
 1.0 
 8.5 
 8.5 
 3.4 
 NA 
 NA 
 NA 
 6.57e-04 
 
 
 SOCS1 
 4.7 
 0.0 
 0.0 
 1.5 
 NA 
 NA 
 NA 
 3.11e-03 
 
 
 CBL 
 2.9 
 0.6 
 0.6 
 0.9 
 NA 
 NA 
 NA 
 4.28e-02 
 
 
 
  # Significant genes in extension cohort
extension_drivers <- extension_only_dnds_shm$sel_merged$gene_name[extension_only_dnds_shm$sel_merged$qglobalpos_m < 0.1]

# Total number of non-synonymous mutations across driver genes
extension_driver_muts <- mutations[mutations$gene %in% extension_drivers & mutations$sampleID %in% extension_all,]
extension_driver_muts_unique <- extension_driver_muts[!(duplicated(extension_driver_muts$mut_site)),]

extension_driver_counts <- sort(colSums(table(substr(extension_driver_muts_unique$paper_id[extension_driver_muts_unique$impact != "Synonymous"],1,2), extension_driver_muts_unique$gene[extension_driver_muts_unique$impact != "Synonymous"])), decreasing = T)

kable(extension_driver_counts, col.names = c("Gene","Mutation count"), caption = "Total non-synonymous mutations (unique per donor) in extension cohort across driver genes identified in extension cohort only")  
 
 Total non-synonymous mutations (unique per donor) in extension
cohort across driver genes identified in extension cohort only 
 
 
 Gene 
 Mutation count 
 
 
 
 
 TG 
 378 
 
 
 DUSP2 
 99 
 
 
 LTB 
 97 
 
 
 CD274 
 76 
 
 
 CBL 
 53 
 
 
 TNFRSF14 
 40 
 
 
 TNFAIP3 
 38 
 
 
 IRF1 
 26 
 
 
 SOCS1 
 23 
 
 
 
 In order to address whether the signal of selection in the extension
cohort was solely being driven by donor H8, below we repeat the dN/dS
analysis on the extension cohort after excluding donor H8. 
  # dNdS results for combined exome and targeted data fron extension cohort only (restricted to genes in targeted panel)
sample_list <- extension_all[which(!(grepl("PD66718",extension_all)))]

dnds_muts <- mutations[which(mutations$sampleID %in% sample_list),]
dnds_muts <- dnds_muts[which(!(duplicated(dnds_muts$mut_site))),]

dnds_cov <- rowSums(coverage[which(colnames(coverage) %in% sample_list)])
names(dnds_cov) <- coverage$gene
dnds_cov <- dnds_cov[which(dnds_cov != 0)]

# 2024-02-19 - Made decision to exclude IGLL5 and IGLL1 from dNdS analysis. Only reason these Ig genes (but not others) are in the dNdS object is they are in frame.
dnds_cov <- dnds_cov[which(!(names(dnds_cov) %in% c("IGLL5","IGLL1")))]

# Restrict dN/dS analysis to genes in targeted panel
dnds_cov <- dnds_cov[which(names(dnds_cov) %in% immune_genes$gene[which(immune_genes$target_type != "Hotspot")])]

extension_only_excluding_H8_dnds_shm <- dnds_shm(mutations = dnds_muts[,c("sampleID","chr","pos","ref","mut")],
                                                 refdb_noshm = RefCDS_noshm_exome,
                                                 refdb_shm = RefCDS_shm_exome,
                                                 dc_noshm = dnds_cov,
                                                 gene_list = names(dnds_cov),
                                                 # Exons with just the start codon need to be excluded (synonymous mutations are not possible for dNdSloc)
                                                 excl_shm = c("IGLL1","IGLL5","MYO1E","ZNF595","AC026202.1","EHBP1L1"),
                                                 excl_noshm = c("IGLL1","IGLL5"),
                                                 onesided = T,
                                                 maxcovs = 10)  
  dnds_ratios <- extension_only_excluding_H8_dnds_shm$sel_merged[extension_only_excluding_H8_dnds_shm$sel_merged$qglobalpos_m < 0.1,c("gene_name","wmis_cv","wnon_cv","wspl_cv","wind_cv","wmis_loc","wnon_loc","wspl_loc","qglobalpos_m")]
dnds_ratios$qglobalpos_m <- format(dnds_ratios$qglobalpos_m, digits = 3)
kable(dnds_ratios, caption = "Significant genes identified from extension cohort only (excluding donor H8)", col.names = c("Gene", "dN/dS missense (selcv nonSHM)", "dN/dS nonsense (selcv nonSHM)", "dN/dS splice (selcv nonSHM)","dN/dS indels (selcv nonSHM)","dN/dS missense (selloc SHM)","dN/dS nonsense (selloc SHM)","dN/dS splice (selloc SHM)","Q-value (pos merged)"), digits = 1, row.names = F)  
 
 Significant genes identified from extension cohort only
(excluding donor H8) 
 
 
 
 
 
 
 
 
 
 
 
 
 
 Gene 
 dN/dS missense (selcv nonSHM) 
 dN/dS nonsense (selcv nonSHM) 
 dN/dS splice (selcv nonSHM) 
 dN/dS indels (selcv nonSHM) 
 dN/dS missense (selloc SHM) 
 dN/dS nonsense (selloc SHM) 
 dN/dS splice (selloc SHM) 
 Q-value (pos merged) 
 
 
 
 
 TNFRSF14 
 3.6 
 44.3 
 44.3 
 6.4 
 NA 
 NA 
 NA 
 4.32e-11 
 
 
 TG 
 1.6 
 0.8 
 0.8 
 14.8 
 0.8 
 0.0 
 0.0 
 1.04e-10 
 
 
 CD274 
 3.1 
 12.0 
 12.0 
 5.4 
 NA 
 NA 
 NA 
 3.27e-06 
 
 
 IRF1 
 4.8 
 11.4 
 11.4 
 1.5 
 NA 
 NA 
 NA 
 1.20e-03 
 
 
 LTB 
 NA 
 NA 
 NA 
 NA 
 1.1 
 6.5 
 6.5 
 5.30e-03 
 
 
 DUSP2 
 NA 
 NA 
 NA 
 NA 
 1.1 
 6.4 
 6.4 
 5.30e-03 
 
 
 DNMT3A 
 2.1 
 5.9 
 5.9 
 2.1 
 NA 
 NA 
 NA 
 8.13e-02 
 
 
 
  # Significant genes in extension cohort
extension_excluding_H8_drivers <- extension_only_excluding_H8_dnds_shm$sel_merged$gene_name[extension_only_excluding_H8_dnds_shm$sel_merged$qglobalpos_m < 0.1]

# Total number of non-synonymous mutations across driver genes
extension_excluding_H8_driver_muts <- mutations[mutations$gene %in% extension_excluding_H8_drivers & mutations$sampleID %in% extension_all[which(!(grepl("PD66718",extension_all)))],]
extension_excluding_H8_driver_muts_unique <- extension_excluding_H8_driver_muts[!(duplicated(extension_excluding_H8_driver_muts$mut_site)),]

extension_excluding_H8_driver_counts <- sort(colSums(table(substr(extension_excluding_H8_driver_muts_unique$paper_id[extension_excluding_H8_driver_muts_unique$impact != "Synonymous"],1,2), extension_excluding_H8_driver_muts_unique$gene[extension_excluding_H8_driver_muts_unique$impact != "Synonymous"])), decreasing = T)

kable(extension_excluding_H8_driver_counts, col.names = c("Gene","Mutation count"), caption = "Total non-synonymous mutations (unique per donor) in extension cohort (excluding donor H8) across driver genes identified in extension cohort (excluding donor H8) only")  
 
 Total non-synonymous mutations (unique per donor) in extension
cohort (excluding donor H8) across driver genes identified in extension
cohort (excluding donor H8) only 
 
 
 Gene 
 Mutation count 
 
 
 
 
 TG 
 349 
 
 
 DUSP2 
 52 
 
 
 LTB 
 42 
 
 
 CD274 
 35 
 
 
 DNMT3A 
 32 
 
 
 TNFRSF14 
 25 
 
 
 IRF1 
 20 
 
 
 
 
 
 
 9. Variability in mutation frequency between donors (bulk
samples) 
 
 Interdonor variability heatmap (excluding LCM samples) 
  # Define driver genes to plot
genes2plot <- c(all_drivers,"RLTPR")

# Remove LCM samples from mutant cell fraction calculation and from number of mutations as these will skew the mutant cell fraction of driver genes enriched in lymphocytes (we will plot a separate heatmap for LCM samples)
samples2plot <- metadata$sample[!(metadata$sample_type == "LCM")]

# Define mutation inputs
# All mutations in a sample for strip below heatmap
allmuts2plot <- mutations[which(mutations$sampleID %in% samples2plot),]
alluniq2plot <- allmuts2plot[which(!(duplicated(allmuts2plot$mut_site))),]

# Limit mutations to those non-synonymous mutations in driver genes
muts2plot <- mutations[which(mutations$gene %in% genes2plot & mutations$sampleID %in% samples2plot & mutations$impact != "Synonymous"),]
# Remove mutations with NA duplex VAF (only affects three indels in HLA-A) from heatmap
muts2plot <- muts2plot[!(is.na(muts2plot$duplex_vaf)),]
uniq2plot <- muts2plot[which(!(duplicated(muts2plot$mut_site))),]
donors2plot <- unique(substr(metadata$paper_id,1,2))
donor_mut_count <- table(uniq2plot$gene, substr(uniq2plot$paper_id,1,2))

# Define age and sex of donors (age will be used for ordering of donors, sex will be used for mutant cell fraction calculation)
age <- setNames(metadata$age, substr(metadata$paper_id,1,2))[colnames(donor_mut_count)]
age <- age[order(age, decreasing = F)]
sex <- substr(setNames(metadata$sex, substr(metadata$paper_id,1,2)),1,1)[colnames(donor_mut_count)]
donor_mut_count <- donor_mut_count[match(all_dnds_shm$sel_merged$gene_name[all_dnds_shm$sel_merged$gene_name %in% rownames(donor_mut_count)], rownames(donor_mut_count)),]

# Order samples by lymphocyte fraction
emseq_bulk <- emseq[emseq$sampleID %in% gsub("_exome","",gsub("_targeted","",metadata$sample[metadata$sample_type == "Bulk"])),c("paper_id","Blood_B", "Blood_T","Blood_NK")]
emseq_bulk <- emseq_bulk[!(emseq_bulk$paper_id %in% c("H3b","H3c")),]
emseq_bulk$paper_id <- substr(emseq_bulk$paper_id,1,2)
emseq_bulk$Blood_lymph <- emseq_bulk$Blood_B+emseq_bulk$Blood_T+emseq_bulk$Blood_NK
# emseq_bulk <- emseq_bulk[,-which(colnames(emseq_bulk) %in% c("Blood_B", "Blood_T"))]
emseq_bulk_agg <- aggregate(list(Blood_lymph=emseq_bulk$Blood_lymph), by=list(paper_id=emseq_bulk$paper_id), FUN="mean") 
emseq_bulk_agg <- emseq_bulk_agg[order(emseq_bulk_agg$Blood_lymph, decreasing = T),]
emseq_bulk_B <- aggregate(list(Blood_B=emseq_bulk$Blood_B), by=list(paper_id=emseq_bulk$paper_id), FUN="mean") 
emseq_bulk_T <- aggregate(list(Blood_T=emseq_bulk$Blood_T), by=list(paper_id=emseq_bulk$paper_id), FUN="mean") 

# Calculate polyclonal mutant cell fraction
cellfraction = array(NA, dim = c(length(donors2plot),8,length(genes2plot)), dimnames = list(donors2plot, c("mis","non","spl","ind","mislow","nonlow","spllow","indlow"),genes2plot))

nonsyn_lbd <- as.data.frame(array(data = NA, dim = c(length(donors2plot)*length(genes2plot),3)))
colnames(nonsyn_lbd) <- c("donor","gene","cellfraction")

for(x in 1:length(genes2plot)) {  
  for (j in 1:length(donors2plot)) {
    aux = muts2plot[which(muts2plot$gene==genes2plot[x] & substr(muts2plot$paper_id,1,2) == donors2plot[j]),c("sampleID","paper_id","impact","cellfraction","duplex_vaf")]
    per_sample_gene_cov <- coverage[which(coverage$gene == genes2plot[x]),colnames(coverage) %in% samples2plot[samples2plot %in% muts2plot$sampleID[substr(muts2plot$paper_id,1,2) == donors2plot[j]]], drop = F]
    if(nrow(aux) > 0){
      aux$sample_gene_cov <- NA
      aux$cov_weighting <- NA
      for(k in 1:length(per_sample_gene_cov)){
        aux$sample_gene_cov[which(aux$sampleID == names(per_sample_gene_cov)[k])] <- as.numeric(per_sample_gene_cov[k])
      }
      aux$cov_weighting <- aux$sample_gene_cov / sum(per_sample_gene_cov)
      
      aux$weighted_cellfraction <- aux$cellfraction * aux$cov_weighting
      aux$weighted_duplex_vaf <- aux$duplex_vaf * aux$cov_weighting
      
      cellfraction[j,c("mis","mislow"),x] = colSums(aux[aux$impact %in% c("Missense","Start_loss","Stop_loss"),c("weighted_cellfraction","weighted_duplex_vaf")], na.rm = T)
      cellfraction[j,c("non","nonlow"),x] = colSums(aux[aux$impact=="Nonsense",c("weighted_cellfraction","weighted_duplex_vaf")], na.rm = T)
      cellfraction[j,c("spl","spllow"),x] = colSums(aux[aux$impact=="Essential_Splice",c("weighted_cellfraction","weighted_duplex_vaf")], na.rm = T)
      cellfraction[j,c("ind","indlow"),x] = colSums(aux[aux$impact=="no-SNV",c("weighted_cellfraction","weighted_duplex_vaf")], na.rm = T)
    }else{
      cellfraction[j,1:8,x] = 0
    }
    nonsyn_lbd$donor[(x-1)*length(donors2plot)+j] <- donors2plot[j]
    nonsyn_lbd$gene[(x-1)*length(donors2plot)+j] <- genes2plot[x]
    nonsyn_lbd$cellfraction[(x-1)*length(donors2plot)+j] <- sum(cellfraction[j,c(5:8),x])
  }
}

# Transpose and reorganise data for plotting
grid = expand.grid(x = rownames(donor_mut_count), y = colnames(donor_mut_count))
grid$z = c(donor_mut_count)
grid_merge <- merge(x=grid,y=nonsyn_lbd, by.x=c("y","x"), by.y=c("donor","gene"))
names(grid_merge)[names(grid_merge) == "cellfraction"] <- "a"
grid_merge$y <- factor(x = grid_merge$y, levels = emseq_bulk_agg$paper_id)
grid_merge$x <- factor(x = grid_merge$x, levels = rev(genes2plot))
grid_merge$a <- as.numeric(grid_merge$a)*100

# Cap the mutant cell fraction at 10% to highlight those in the 1-10% range
grid_merge[which(grid_merge$a > 10),]$a <- 10

# Define colour scheme
color.palette = colorRampPalette(c("white", "darkorange", "plum3"))

lattice.options(axis.padding=list(factor=0.5)) #remove white border around lattice heatmap
bulk_heatmap <- lattice::levelplot(a~y*x, data = grid_merge, col.regions=color.palette(11001)[c(1,1001:11001)], scales = list(tck = c(0,0), y = list(cex=1.5), x = list(cex = 1.5, alternating = 3, rot=0)), ylab=" ", xlab = "", colorkey=list(space="top", labels=list(cex=1.5)), at = seq(from = 0, to = 10, by = 0.001), main = list(label = "Aggregate mutant cell fraction (lower bound %)", cex = 2, side=1, line=0.5, hjust=0.3)) +
  latticeExtra::as.layer(
    lattice::levelplot(z~y*x, grid_merge, col.regions=NA, scales = list(tck = c(0,0), y = list(cex=1.5), x = list(cex=1.5,rot=0)), colorkey=list(space="bottom"),
                       panel=function(...) { arg <- list(...)
                       panel.levelplot(...)
                       panel.text(arg$x, arg$y, arg$z)}))  
 
 
 Additional information plot for interdonor variability heatmap
(excluding LCM samples) 
  # Calculate duplex coverage per donor excluding LCM samples
dx_per_donor <- aggregate(metadata$median_duplex_cov[metadata$sample_type != "LCM"], by=list(Category=substr(metadata$paper_id[metadata$sample_type != "LCM"],1,2)), FUN=sum)
ndx <- setNames(dx_per_donor$x, dx_per_donor$Category)

# Calculate number of mutations per donor excluding LCM samples
nmuts <- table(substr(alluniq2plot$paper_id, 1, 2))

# Create tables to combine information for subplots
meta = rbind(ndx[colnames(donor_mut_count)],nmuts[colnames(donor_mut_count)],
             setNames(metadata$cohort, substr(metadata$paper_id,1,2))[colnames(donor_mut_count)],
             setNames(metadata$pathology, substr(metadata$paper_id,1,2))[colnames(donor_mut_count)],
             setNames(emseq_bulk_agg$Blood_lymph, emseq_bulk_agg$paper_id)[colnames(donor_mut_count)],
             setNames(emseq_bulk_B$Blood_B, emseq_bulk_B$paper_id)[colnames(donor_mut_count)],
             setNames(emseq_bulk_T$Blood_T, emseq_bulk_T$paper_id)[colnames(donor_mut_count)],
             setNames(metadata$sex, substr(metadata$paper_id,1,2))[colnames(donor_mut_count)],
             setNames(metadata$age, substr(metadata$paper_id,1,2))[colnames(donor_mut_count)])
colnames(meta) = colnames(donor_mut_count)
rownames(meta) = c("Duplex coverage","Mutations", "Cohort", "Disease", "Lymphocyte %","B cell %","T cell %","Sex", "Age")
grid2 = expand.grid(x=rownames(meta), y=colnames(meta))
grid2$z = c(meta)

# Convert data for subplots
grid2$z <- gsub("Pilot", 1, grid2$z)
grid2$z <- gsub("Extension", 2, grid2$z)
grid2$z <- gsub("Hashimoto", 3, grid2$z)
grid2$z <- gsub("Graves", 4, grid2$z)
grid2[which(grid2$x == "Lymphocyte %"),]$z <- round(as.numeric(grid2[which(grid2$x == "Lymphocyte %"),]$z)*100, digits = 0)
grid2[which(grid2$x == "B cell %"),]$z <- round(as.numeric(grid2[which(grid2$x == "B cell %"),]$z)*100, digits = 0)
grid2[which(grid2$x == "T cell %"),]$z <- round(as.numeric(grid2[which(grid2$x == "T cell %"),]$z)*100, digits = 0)
grid2$z <- gsub("Male", 5, grid2$z)
grid2$z <- gsub("Female", 6, grid2$z)

# Order by lymphocyte fraction (or age)
grid2$y <- factor(x = grid2$y, levels = emseq_bulk_agg$paper_id)
grid2 <- grid2[order(grid2$y),]

# Create dx heatmap
color.palette = colorRampPalette(c("white","grey50"))(ceiling(max(as.numeric(grid2$z[which(grid2$x == "Duplex coverage")])) / 500) + 1)
p1 <- levelplot(z~y*x, grid2[which(grid2$x == "Duplex coverage"),], col.regions= color.palette, at = seq(from = 0, to = (length(color.palette) - 1) * 500, by = 500), scales = list(tck = c(0,0), y = list(cex=1, rot = 0), x = list(rot=0)), ylab = NULL, xlab="", colorkey=FALSE,
                panel=function(...) { arg <- list(...)
                panel.levelplot(...)
                panel.text(arg$x, arg$y, arg$z, cex = 0.8)})

# Create mutations heatmap
color.palette = colorRampPalette(c("white","cadetblue3"))(ceiling(max(as.numeric(grid2$z[which(grid2$x == "Mutations")])) / 500) + 1)
p2 <- levelplot(z~y*x, grid2[which(grid2$x == "Mutations"),], col.regions=color.palette, at = seq(from = 0, to = (length(color.palette) - 1) * 500, by = 500), scales = list(tck = c(0,0), y = list(cex=1, rot = 0), x = list(rot=0)), ylab = NULL, xlab="", colorkey=FALSE,
                panel=function(...) { arg <- list(...)
                panel.levelplot(...)
                panel.text(arg$x, arg$y, arg$z, cex = 0.8)})

# Create cohort heatmap
color.palette = colorRampPalette(c("peachpuff","lightcoral"))
p3 <- levelplot(z~y*x, grid2[which(grid2$x == "Cohort"),], col.regions=color.palette, scales = list(tck = c(0,0), y = list(cex=1, rot = 0), x = list(rot=0)), ylab = NULL, xlab="", colorkey=FALSE)

# Create disease heatmap
color.palette = colorRampPalette(c("lightyellow1","palegreen1"))
p4 <- levelplot(z~y*x, grid2[which(grid2$x == "Disease"),], col.regions=color.palette, scales = list(tck = c(0,0), y = list(cex=1, rot = 0), x = list(alternating=3,rot=0)), ylab = NULL, xlab="", colorkey=FALSE)

# Create lymphocyte heatmap
color.palette = colorRampPalette(c("white","purple"))(101)
p5 <- levelplot(z~y*x, grid2[which(grid2$x == "Lymphocyte %"),], col.regions=color.palette,  at = seq(from = 0, to = 100, by = 1), scales = list(tck = c(0,0), y = list(cex=1, rot = 0), x = list(rot=0)), ylab = NULL, xlab="", colorkey=FALSE,
                panel=function(...) { arg <- list(...)
                panel.levelplot(...)
                panel.text(arg$x, arg$y, arg$z, cex = 0.8)})

# Create B cell heatmap
color.palette = colorRampPalette(c("white","cornflowerblue"))(101)
p6 <- levelplot(z~y*x, grid2[which(grid2$x == "B cell %"),], col.regions=color.palette,  at = seq(from = 0, to = 100, by = 1), scales = list(tck = c(0,0), y = list(cex=1, rot = 0), x = list(rot=0)), ylab = NULL, xlab="", colorkey=FALSE,
                panel=function(...) { arg <- list(...)
                panel.levelplot(...)
                panel.text(arg$x, arg$y, arg$z, cex = 0.8)})

# Create T cell heatmap
color.palette = colorRampPalette(c("white","orchid"))(101)
p7 <- levelplot(z~y*x, grid2[which(grid2$x == "T cell %"),], col.regions=color.palette,  at = seq(from = 0, to = 100, by = 1), scales = list(tck = c(0,0), y = list(cex=1, rot = 0), x = list(rot=0)), ylab = NULL, xlab="", colorkey=FALSE,
                panel=function(...) { arg <- list(...)
                panel.levelplot(...)
                panel.text(arg$x, arg$y, arg$z, cex = 0.8)})


# Create sex heatmap
color.palette = colorRampPalette(c("lightblue","lightpink"))
p8 <- levelplot(z~y*x, grid2[which(grid2$x == "Sex"),], col.regions=color.palette, scales = list(tck = c(0,0), y = list(cex=1, rot = 0), x = list(rot=0)), ylab = NULL, xlab="", colorkey=FALSE)

# Create age heatmap
# Subset grid2 to label with age ranges (plotted as factors)
kk <- grid2[grid2$x == "Age",]
age_factors <- paste0(seq(from = 0, to = 7),"0-",seq(from = 0, to = 7),"9")
kk$k <- NA
for(x in 1:nrow(kk)){
  kk$k[x] <- which(age_factors == kk$z[x])
}
kk$y <- factor(x = kk$y, levels = emseq_bulk_agg$paper_id)
kk <- kk[order(kk$y),]

color.palette = colorRampPalette(c("white","forestgreen"))(length(age_factors))
p9 <- levelplot(k~y*x, kk, col.regions=color.palette, at=seq(from=0,to=length(age_factors),by = 1), scales = list(tck = c(0,0), y = list(cex=1, rot = 0), x = list(rot=0)), ylab = NULL, xlab="", colorkey=FALSE,
                panel=function(...) { arg <- list(...)
                panel.levelplot(...)
                panel.text(arg$x, arg$y, labels = kk$z, col = "black", cex = 0.8)})

# Plot all the heatmaps together
if (runman) { dev.new(width=7.5, height=8) }
comb_levObj <- c(p4, p3, p8, p9, p2, p1, p7, p6, p5, bulk_heatmap, layout = c(1, 10), merge.legends = FALSE)
plot(comb_levObj, panel.height = list(c(1,1,1,1,1,1,1,1,1,length(genes2plot)),c("null")))
if (runman) { dev.copy(pdf,"./output/Fig2D_InterdonorVariabilityBulkSamples_BcellTcellAnnotated.pdf",width=7.5,height=8); dev.off() }  
    
 
 
 Legend for interdonor variability heatmap (excluding LCM
samples) 
  # Plot and save legends
par(mfrow = c(1, 4), mar = c(2,2,2,2))
if (runman) { dev.new(width=10, height=10) }
plot(NULL ,xaxt='n',yaxt='n',bty='n',ylab='',xlab='', xlim=0:1, ylim=0:1)
legend("topleft", legend =c('Male', 'Female'), pch=15, pt.cex=3, cex=1, bty='n', col = c("lightblue","lightpink"))
mtext("Sex", at=0.01, cex=1)
if (runman) { dev.copy(pdf,"./output/Fig2D_InterdonorVariabilityBulkSamples_legend1.pdf",width=10,height=10); dev.off() }

if (runman) { dev.new(width=10, height=10) }
plot(NULL ,xaxt='n',yaxt='n',bty='n',ylab='',xlab='', xlim=0:1, ylim=0:1)
legend("topleft", legend =c('Pilot', 'Extension'), pch=15, pt.cex=3, cex=1, bty='n', col = c("peachpuff", "lightcoral"))
mtext("Cohort", at=0.01, cex=1)
if (runman) { dev.copy(pdf,"./output/Fig2D_InterdonorVariabilityBulkSamples_legend2.pdf",width=10,height=10); dev.off() }

if (runman) { dev.new(width=10, height=10) }
plot(NULL ,xaxt='n',yaxt='n',bty='n',ylab='',xlab='', xlim=0:1, ylim=0:1)
legend("topleft", legend =c('Hashimoto', 'Graves'), pch=15, pt.cex=3, cex=1, bty='n', col = c("lightyellow1","palegreen1"))
mtext("Disease", at=0.01, cex=1)
if (runman) { dev.copy(pdf,"./output/Fig2D_InterdonorVariabilityBulkSamples_legend3.pdf",width=10,height=10); dev.off() }  
    
 
 
 
 10. Variability in mutation frequency between donors (LCM samples
only) 
 
 Interdonor variability heatmap (LCM samples only) 
  # Define driver genes to plot
genes2plot <- c(all_drivers,"RLTPR")

# Remove LCM samples from mutant cell fraction calculation and from number of mutations as these will skew the mutant cell fraction of driver genes enriched in lymphocytes (we will plot a separate heatmap for LCM samples)
samples2plot <- metadata$sample[(metadata$sample_type == "LCM")]

# Define mutation inputs
# All mutations in a sample for strip below heatmap
allmuts2plot <- mutations[which(mutations$sampleID %in% samples2plot),]
alluniq2plot <- allmuts2plot[which(!(duplicated(allmuts2plot$mut_site))),]

# Limit mutations to those non-synonymous mutations in driver genes
muts2plot <- mutations[which(mutations$gene %in% genes2plot & mutations$sampleID %in% samples2plot & mutations$impact != "Synonymous"),]
# Remove mutations with NA duplex VAF (only affects three indels in HLA-A) from heatmap
muts2plot <- muts2plot[!(is.na(muts2plot$duplex_vaf)),]
uniq2plot <- muts2plot[which(!(duplicated(muts2plot$mut_site))),]
donors2plot <- unique(substr(muts2plot$paper_id,1,2))
donor_mut_count <- table(uniq2plot$gene, substr(uniq2plot$paper_id,1,2))

# Define age and sex of donors (age will be used for ordering of donors, sex will be used for mutant cell fraction calculation)
age <- setNames(metadata$age, substr(metadata$paper_id,1,2))[colnames(donor_mut_count)]
age <- age[order(age, decreasing = F)]
sex <- substr(setNames(metadata$sex, substr(metadata$paper_id,1,2)),1,1)[colnames(donor_mut_count)]
donor_mut_count <- donor_mut_count[match(all_dnds_shm$sel_merged$gene_name[all_dnds_shm$sel_merged$gene_name %in% rownames(donor_mut_count)], rownames(donor_mut_count)),]

# Order samples by B-cell fraction
emseq_LCM <- emseq[emseq$sampleID %in% gsub("_exome","",gsub("_targeted","",metadata$sample[metadata$sample_type == "LCM"])),c("paper_id","Blood_B", "Blood_T","Blood_NK")]
emseq_LCM <- emseq_LCM[!(emseq_LCM$paper_id %in% c("H3b","H3c")),]
emseq_LCM$paper_id <- substr(emseq_LCM$paper_id,1,2)
emseq_LCM$Blood_lymph <- emseq_LCM$Blood_B+emseq_LCM$Blood_T+emseq_LCM$Blood_NK
emseq_LCM_agg <- aggregate(list(Blood_lymph=emseq_LCM$Blood_lymph), by=list(paper_id=emseq_LCM$paper_id), FUN="mean") 
emseq_LCM_agg <- emseq_LCM_agg[order(emseq_LCM_agg$Blood_lymph, decreasing = T),]
emseq_LCM_B <- aggregate(list(Blood_B=emseq_LCM$Blood_B), by=list(paper_id=emseq_LCM$paper_id), FUN="mean") 
emseq_LCM_T <- aggregate(list(Blood_T=emseq_LCM$Blood_T), by=list(paper_id=emseq_LCM$paper_id), FUN="mean") 

# Calculate polyclonal mutant cell fraction
cellfraction = array(NA, dim = c(length(donors2plot),8,length(genes2plot)), dimnames = list(donors2plot, c("mis","non","spl","ind","mislow","nonlow","spllow","indlow"),genes2plot))

nonsyn_lbd <- as.data.frame(array(data = NA, dim = c(length(donors2plot)*length(genes2plot),3)))
colnames(nonsyn_lbd) <- c("donor","gene","cellfraction")

for(x in 1:length(genes2plot)) {  
  for (j in 1:length(donors2plot)) {
    aux = muts2plot[which(muts2plot$gene==genes2plot[x] & substr(muts2plot$paper_id,1,2) == donors2plot[j]),c("sampleID","paper_id","impact","cellfraction","duplex_vaf")]
    per_sample_gene_cov <- coverage[which(coverage$gene == genes2plot[x]),colnames(coverage) %in% samples2plot[samples2plot %in% muts2plot$sampleID[substr(muts2plot$paper_id,1,2) == donors2plot[j]]], drop = F]
    if(nrow(aux) > 0){
      aux$sample_gene_cov <- NA
      aux$cov_weighting <- NA
      for(k in 1:length(per_sample_gene_cov)){
        aux$sample_gene_cov[which(aux$sampleID == names(per_sample_gene_cov)[k])] <- as.numeric(per_sample_gene_cov[k])
      }
      aux$cov_weighting <- aux$sample_gene_cov / sum(per_sample_gene_cov)
      
      aux$weighted_cellfraction <- aux$cellfraction * aux$cov_weighting
      aux$weighted_duplex_vaf <- aux$duplex_vaf * aux$cov_weighting
      
      cellfraction[j,c("mis","mislow"),x] = colSums(aux[aux$impact %in% c("Missense","Start_loss","Stop_loss"),c("weighted_cellfraction","weighted_duplex_vaf")], na.rm = T)
      cellfraction[j,c("non","nonlow"),x] = colSums(aux[aux$impact=="Nonsense",c("weighted_cellfraction","weighted_duplex_vaf")], na.rm = T)
      cellfraction[j,c("spl","spllow"),x] = colSums(aux[aux$impact=="Essential_Splice",c("weighted_cellfraction","weighted_duplex_vaf")], na.rm = T)
      cellfraction[j,c("ind","indlow"),x] = colSums(aux[aux$impact=="no-SNV",c("weighted_cellfraction","weighted_duplex_vaf")], na.rm = T)
    }else{
      cellfraction[j,1:8,x] = 0
    }
    nonsyn_lbd$donor[(x-1)*length(donors2plot)+j] <- donors2plot[j]
    nonsyn_lbd$gene[(x-1)*length(donors2plot)+j] <- genes2plot[x]
    nonsyn_lbd$cellfraction[(x-1)*length(donors2plot)+j] <- sum(cellfraction[j,c(5:8),x])
  }
}

# Transpose and reorganise data for plotting
grid = expand.grid(x = rownames(donor_mut_count), y = colnames(donor_mut_count))
grid$z = c(donor_mut_count)
grid_merge <- merge(x=grid,y=nonsyn_lbd, by.x=c("y","x"), by.y=c("donor","gene"))
names(grid_merge)[names(grid_merge) == "cellfraction"] <- "a"
grid_merge$y <- factor(x = grid_merge$y, levels = emseq_LCM_agg$paper_id)
grid_merge$x <- factor(x = grid_merge$x, levels = rev(genes2plot))
grid_merge$a <- as.numeric(grid_merge$a)*100

# Cap the mutant cell fraction at 10% to highlight those in the 1-10% range
grid_merge[which(grid_merge$a > 10),]$a <- 10

# Define colour scheme
color.palette = colorRampPalette(c("white", "darkorange", "plum3"))

lattice.options(axis.padding=list(factor=0.5)) #remove white border around lattice heatmap
lcm_heatmap <- lattice::levelplot(a~y*x, data = grid_merge, col.regions=color.palette(11001)[c(1,1001:11001)], scales = list(tck = c(0,0), y = list(cex=1), x = list(alternating = 3, rot=0)), ylab=" ", xlab = "", colorkey=list(space="top"), at = seq(from = 0, to = 10, by = 0.001), main = list(label = "Polyclonal mutant cell fraction (lower bound %)", cex = 1, side=1, line=0.5, hjust=0.3)) +
  latticeExtra::as.layer(
    lattice::levelplot(z~y*x, grid_merge, col.regions=NA, scales = list(tck = c(0,0), y = list(cex=1), x = list(rot=0)), colorkey=list(space="bottom"),
                       panel=function(...) { arg <- list(...)
                       panel.levelplot(...)
                       panel.text(arg$x, arg$y, arg$z)}))  
 
 
 Additional information plot for interdonor variability heatmap (LCM
samples only) 
  # Calculate duplex coverage per donor excluding LCM samples
dx_per_donor <- aggregate(metadata$median_duplex_cov[metadata$sample_type == "LCM"], by=list(Category=substr(metadata$paper_id[metadata$sample_type == "LCM"],1,2)), FUN=sum)
ndx <- setNames(dx_per_donor$x, dx_per_donor$Category)

# Calculate number of mutations per donor excluding LCM samples
nmuts <- table(substr(alluniq2plot$paper_id, 1, 2))

# Create tables to combine information for subplots
meta = rbind(ndx[colnames(donor_mut_count)],nmuts[colnames(donor_mut_count)],
             setNames(metadata$cohort, substr(metadata$paper_id,1,2))[colnames(donor_mut_count)],
             setNames(metadata$pathology, substr(metadata$paper_id,1,2))[colnames(donor_mut_count)],
             setNames(emseq_LCM_agg$Blood_lymph, emseq_LCM_agg$paper_id)[colnames(donor_mut_count)],
             setNames(emseq_LCM_B$Blood_B, emseq_LCM_B$paper_id)[colnames(donor_mut_count)],
             setNames(emseq_LCM_T$Blood_T, emseq_LCM_T$paper_id)[colnames(donor_mut_count)],
             setNames(metadata$sex, substr(metadata$paper_id,1,2))[colnames(donor_mut_count)],
             setNames(metadata$age, substr(metadata$paper_id,1,2))[colnames(donor_mut_count)])
colnames(meta) = colnames(donor_mut_count)
rownames(meta) = c("Duplex coverage","Mutations", "Cohort", "Disease", "Lymphocyte %","B cell %","T cell %","Sex", "Age")
grid2 = expand.grid(x=rownames(meta), y=colnames(meta))
grid2$z = c(meta)

# Convert data for subplots
grid2$z <- gsub("Pilot", 1, grid2$z)
grid2$z <- gsub("Extension", 2, grid2$z)
grid2$z <- gsub("Hashimoto", 3, grid2$z)
grid2$z <- gsub("Graves", 4, grid2$z)
grid2[which(grid2$x == "Lymphocyte %"),]$z <- round(as.numeric(grid2[which(grid2$x == "Lymphocyte %"),]$z)*100, digits = 0)
grid2[which(grid2$x == "B cell %"),]$z <- round(as.numeric(grid2[which(grid2$x == "B cell %"),]$z)*100, digits = 0)
grid2[which(grid2$x == "T cell %"),]$z <- round(as.numeric(grid2[which(grid2$x == "T cell %"),]$z)*100, digits = 0)
grid2$z <- gsub("Male", 5, grid2$z)
grid2$z <- gsub("Female", 6, grid2$z)

# Order by lymphocyte fraction (or age)
grid2$y <- factor(x = grid2$y, levels = emseq_LCM_agg$paper_id)
grid2 <- grid2[order(grid2$y),]

# Create dx heatmap
color.palette = colorRampPalette(c("white","grey50"))(ceiling(max(as.numeric(grid2$z[which(grid2$x == "Duplex coverage")])) / 500) + 1)
p1 <- levelplot(z~y*x, grid2[which(grid2$x == "Duplex coverage"),], col.regions= color.palette, at = seq(from = 0, to = (length(color.palette) - 1) * 500, by = 500), scales = list(tck = c(0,0), y = list(cex=1, rot = 0), x = list(rot=0)), ylab = NULL, xlab="", colorkey=FALSE,
                panel=function(...) { arg <- list(...)
                panel.levelplot(...)
                panel.text(arg$x, arg$y, arg$z, cex = 0.8)})

# Create mutations heatmap
color.palette = colorRampPalette(c("white","cadetblue3"))(ceiling(max(as.numeric(grid2$z[which(grid2$x == "Mutations")])) / 500) + 1)
p2 <- levelplot(z~y*x, grid2[which(grid2$x == "Mutations"),], col.regions=color.palette, at = seq(from = 0, to = (length(color.palette) - 1) * 500, by = 500), scales = list(tck = c(0,0), y = list(cex=1, rot = 0), x = list(rot=0)), ylab = NULL, xlab="", colorkey=FALSE,
                panel=function(...) { arg <- list(...)
                panel.levelplot(...)
                panel.text(arg$x, arg$y, arg$z, cex = 0.8)})

# Create cohort heatmap
color.palette = colorRampPalette(c("lightcoral"))
p3 <- levelplot(z~y*x, grid2[which(grid2$x == "Cohort"),], col.regions=color.palette, scales = list(tck = c(0,0), y = list(cex=1, rot = 0), x = list(rot=0)), ylab = NULL, xlab="", colorkey=FALSE)

# Create disease heatmap
color.palette = colorRampPalette(c("lightyellow1","palegreen1"))
p4 <- levelplot(z~y*x, grid2[which(grid2$x == "Disease"),], col.regions=color.palette, scales = list(tck = c(0,0), y = list(cex=1, rot = 0), x = list(alternating=3,rot=0)), ylab = NULL, xlab="", colorkey=FALSE)

# Create lymphocyte heatmap
color.palette = colorRampPalette(c("white","purple"))(101)
p5 <- levelplot(z~y*x, grid2[which(grid2$x == "Lymphocyte %"),], col.regions=color.palette,  at = seq(from = 0, to = 100, by = 1), scales = list(tck = c(0,0), y = list(cex=1, rot = 0), x = list(rot=0)), ylab = NULL, xlab="", colorkey=FALSE,
                panel=function(...) { arg <- list(...)
                panel.levelplot(...)
                panel.text(arg$x, arg$y, arg$z, cex = 0.8)})

# Create B cell heatmap
color.palette = colorRampPalette(c("white","cornflowerblue"))(101)
p6 <- levelplot(z~y*x, grid2[which(grid2$x == "B cell %"),], col.regions=color.palette,  at = seq(from = 0, to = 100, by = 1), scales = list(tck = c(0,0), y = list(cex=1, rot = 0), x = list(rot=0)), ylab = NULL, xlab="", colorkey=FALSE,
                panel=function(...) { arg <- list(...)
                panel.levelplot(...)
                panel.text(arg$x, arg$y, arg$z, cex = 0.8)})

# Create T cell heatmap
color.palette = colorRampPalette(c("white","orchid"))(101)
p7 <- levelplot(z~y*x, grid2[which(grid2$x == "T cell %"),], col.regions=color.palette,  at = seq(from = 0, to = 100, by = 1), scales = list(tck = c(0,0), y = list(cex=1, rot = 0), x = list(rot=0)), ylab = NULL, xlab="", colorkey=FALSE,
                panel=function(...) { arg <- list(...)
                panel.levelplot(...)
                panel.text(arg$x, arg$y, arg$z, cex = 0.8)})


# Create sex heatmap
color.palette = colorRampPalette(c("lightblue","lightpink"))
p8 <- levelplot(z~y*x, grid2[which(grid2$x == "Sex"),], col.regions=color.palette, scales = list(tck = c(0,0), y = list(cex=1, rot = 0), x = list(rot=0)), ylab = NULL, xlab="", colorkey=FALSE)

# Create age heatmap
# Subset grid2 to label with age ranges (plotted as factors)
kk <- grid2[grid2$x == "Age",]
age_factors <- paste0(seq(from = 0, to = 7),"0-",seq(from = 0, to = 7),"9")
kk$k <- NA
for(x in 1:nrow(kk)){
  kk$k[x] <- which(age_factors == kk$z[x])
}
kk$y <- factor(x = kk$y, levels = emseq_LCM_agg$paper_id)
kk <- kk[order(kk$y),]

color.palette = colorRampPalette(c("white","forestgreen"))(length(age_factors))
p9 <- levelplot(k~y*x, kk, col.regions=color.palette, at=seq(from=0,to=length(age_factors),by = 1), scales = list(tck = c(0,0), y = list(cex=1, rot = 0), x = list(rot=0)), ylab = NULL, xlab="", colorkey=FALSE,
                panel=function(...) { arg <- list(...)
                panel.levelplot(...)
                panel.text(arg$x, arg$y, labels = kk$z, col = "black", cex = 0.8)})

# Plot all the heatmaps together (to have the y-axis horizontal, only the first plot p5 needs to have the argument)
if (runman) { dev.new(width=4.5, height=12) }
comb_levObj <- c(p4, p3, p8, p9, p2, p1, p7, p6, p5, lcm_heatmap, layout = c(1, 10), merge.legends = FALSE)
plot(comb_levObj, panel.height = list(c(1,1,1,1,1,1,1,1,1,length(genes2plot)),c("null")))
if (runman) { dev.copy(pdf,"./output/EDF1C_InterdonorVariability_LCM_BcellTcellAnnotated.pdf",width=4.5,height=12); dev.off() }  
    
 
 
 Legend for interdonor variability heatmap (LCM samples only) 
  # Plot and save legends
par(mfrow = c(1, 4), mar = c(2,2,2,2))
if (runman) { dev.new(width=10, height=10) }
plot(NULL ,xaxt='n',yaxt='n',bty='n',ylab='',xlab='', xlim=0:1, ylim=0:1)
legend("topleft", legend =c('Male', 'Female'), pch=15, pt.cex=3, cex=1, bty='n', col = c("lightblue","lightpink"))
mtext("Sex", at=0.01, cex=1)
if (runman) { dev.copy(pdf,"./output/EDF1C_InterdonorVariability_LCM_legend1.pdf",width=10,height=10); dev.off() }

if (runman) { dev.new(width=10, height=10) }
plot(NULL ,xaxt='n',yaxt='n',bty='n',ylab='',xlab='', xlim=0:1, ylim=0:1)
legend("topleft", legend =c('Pilot', 'Extension'), pch=15, pt.cex=3, cex=1, bty='n', col = c("peachpuff", "lightcoral"))
mtext("Cohort", at=0.01, cex=1)
if (runman) { dev.copy(pdf,"./output/EDF1C_InterdonorVariability_LCM_legend2.pdf",width=10,height=10); dev.off() }

if (runman) { dev.new(width=10, height=10) }
plot(NULL ,xaxt='n',yaxt='n',bty='n',ylab='',xlab='', xlim=0:1, ylim=0:1)
legend("topleft", legend =c('Hashimoto', 'Graves'), pch=15, pt.cex=3, cex=1, bty='n', col = c("lightyellow1","palegreen1"))
mtext("Disease", at=0.01, cex=1)
if (runman) { dev.copy(pdf,"./output/EDF1C_InterdonorVariability_LCM_legend3.pdf",width=10,height=10); dev.off() }  
    
 
 
 
 11. Mutation distribution plots across coding region (compared to
COSMIC) 
 
 Coding mutation distribution plots 
  dir.create("output/driver_plots", showWarnings = FALSE)

# genes2plot <- c("TNFRSF14","CD274","CCR6","CBL")
#
## To make the R markdown quicker, four selected genes are chosen above. In order to generate mutation distribution plots for all driver genes, run the following:
## RLTPR hotspot is significant by site dNdS only (not gene-level dNdS)
genes2plot <- c(all_drivers,"RLTPR")

for(i in 1:length(genes2plot)){
  gene <- genes2plot[i]
  sample_list <- metadata$sample
  title = "All"
  
  plotfilename = paste0("output/driver_plots/geneplot_",gene,"_coding")
  
  coding_mutation_plot(gene = gene,
                       mutations = mutations,
                       sample_list = sample_list,
                       title = title,
                       gene_coverage_path = paste0("./input/per_gene_per_site_cov/",gene,".tsv"),
                       cosmic_dir_path = "./input/cosmic_data/",
                       interpro_domain_dir_path = "./input/interpro_domain/",
                       immune_genes = immune_genes,
                       plotfilename = plotfilename,
                       runman = runman)
}  
                                  
 
 
 Noncoding mutation distribution plots 
  # genes2plot <- c("TNFRSF14","CD274","CCR6","CBL")
#
## To make the R markdown quicker, four selected genes are chosen above. In order to generate mutation distribution plots for all driver genes, run the following:
## RLTPR hotspot is significant by site dNdS only (not gene-level dNdS)

genes2plot <- c(all_drivers,"RLTPR")

for(i in 1:length(genes2plot)){
  gene <- genes2plot[i]
  sample_list <- metadata$sample
  title = "All"
  
  plotfilename = paste0("output/driver_plots/geneplot_",gene,"_noncoding")
  
  full_gene_mutation_plot(gene = gene,
                          mutations = mutations,
                          sample_list = sample_list,
                          combined_per_sample_cov = coverage,
                          title = title,
                          gene_coverage_path = paste0("./input/per_gene_per_site_cov/",gene,".tsv"),
                          immune_genes = immune_genes,
                          plotfilename = plotfilename,
                          runman = runman)
}  
                                  
 
 
 
 12. Withingenednds 
 
 TNFRSF14 withingenedNdS 
  # The disulphide bridges in TNFRSF14 were annotated based on:
# TNFRSF14: https://www.uniprot.org/uniprotkb/Q92956/entry

# Make additional regions file for disulphide cysteines vs other missense mutations in separate cysteine-rich domains
gene = "TNFRSF14"

load(RefCDS_GRCh37_NSX)
ref_cds_gene_list <- NULL
for(i in 1:length(RefCDS)){
  ref_cds_gene_list <- c(ref_cds_gene_list,RefCDS[[i]]$gene_name)
}

gene_idx <- which(ref_cds_gene_list == gene)

exon_coord <- as.data.frame(RefCDS[[gene_idx]]$intervals_cds)
exon_coord_vec <- NULL
for(x in 1:nrow(exon_coord)){
  exon_coord_vec <- c(exon_coord_vec,exon_coord$V1[x]:exon_coord$V2[x])
}

TNFRSF14_domain <- jsonlite::fromJSON(txt = TNFRSF14_domain_json)$feature
TNFRSF14_CRD_pos_list <- vector("list", nrow(TNFRSF14_domain))

for(x in 1:nrow(TNFRSF14_domain)){
  CRD_aa <- c(TNFRSF14_domain$location$start$value[TNFRSF14_domain$description == paste("TNFR-Cys",x)]:
                TNFRSF14_domain$location$end$value[TNFRSF14_domain$description ==  paste("TNFR-Cys",x)])
  CRD_cDNA <- rep(((CRD_aa - 1) * 3), each = 3) + 1:3
  TNFRSF14_CRD_pos_list[[x]] <- exon_coord_vec[CRD_cDNA]
}

TNFRSF14_ptm <- jsonlite::fromJSON(txt = TNFRSF14_ptm_json)$feature
TNFRSF14_ptm <- TNFRSF14_ptm[TNFRSF14_ptm$type == "Disulfide bond",]
TNFRSF14_disulf_aa <- sort(unique(c(TNFRSF14_ptm$location$start$value,TNFRSF14_ptm$location$end$value)))
TNFRSF14_disulf_cDNA <- rep(((TNFRSF14_disulf_aa - 1) * 3), each = 3) + 1:3
TNFRSF14_disulf_pos <- exon_coord_vec[TNFRSF14_disulf_cDNA]

TNFRSF14_disulf_CRD <- NULL
for(x in 1:length(TNFRSF14_disulf_pos)){
  for(y in 1:3){
    if(TNFRSF14_disulf_pos[x] %in% TNFRSF14_CRD_pos_list[[y]]){
      TNFRSF14_disulf_CRD[x] <- y
    }
  }
}

for(x in 1:3){
  TNFRSF14_CRD_pos_list[[x]] <- TNFRSF14_CRD_pos_list[[x]][which(!(TNFRSF14_CRD_pos_list[[x]]) %in% TNFRSF14_disulf_pos)]
}

# Create customised withingenednds regions_chr for cysteines involved in disulphide bridges
disulf_regions <- as.data.frame(array(data = NA, dim = c(length(TNFRSF14_disulf_pos),6)))
colnames(disulf_regions) <- c("chr","start","end","wname","impacts","layered")

disulf_regions$chr <- RefCDS[[gene_idx]]$chr
disulf_regions$start <- TNFRSF14_disulf_pos
disulf_regions$end <- TNFRSF14_disulf_pos
disulf_regions$wname <- paste0("wdisulphide_bridges_CRD",TNFRSF14_disulf_CRD)
disulf_regions$impacts <- "Missense"
disulf_regions$layered <- 1

# Create customised withingenednds regions_chr for cysteine-rich domains
CRD_regions <- as.data.frame(array(data = NA, dim = c(length(unlist(TNFRSF14_CRD_pos_list)),6)))
colnames(CRD_regions) <- c("chr","start","end","wname","impacts","layered")

CRD_regions$chr <- RefCDS[[gene_idx]]$chr
CRD_regions$start <- unlist(TNFRSF14_CRD_pos_list)
CRD_regions$end <- unlist(TNFRSF14_CRD_pos_list)
wname_vec <- NULL
for(x in 1:length(TNFRSF14_CRD_pos_list)){
  wname_vec <- c(wname_vec,rep(paste0("wCRD",x), times = length(TNFRSF14_CRD_pos_list[[x]])))
}
CRD_regions$wname <- wname_vec
CRD_regions$impacts <- "Missense"
CRD_regions$layered <- 1

# Create customised withingenednds regions_chr for cysteines not involved in disulphide bridges
cys_residues <- which(seqinr::translate(as.vector(RefCDS[[gene_idx]]$seq_cds), numcode = 1) == "C")
cys_cDNA <- rep(((cys_residues - 1) * 3), each = 3) + 1:3
cys_pos_list <- exon_coord_vec[cys_cDNA]
cys_pos_list <- cys_pos_list[which(!(cys_pos_list %in% TNFRSF14_disulf_pos))]

nondisulf_cys_regions <- as.data.frame(array(data = NA, dim = c(length(unlist(cys_pos_list)),6)))
colnames(nondisulf_cys_regions) <- c("chr","start","end","wname","impacts","layered")

nondisulf_cys_regions$chr <- RefCDS[[gene_idx]]$chr
nondisulf_cys_regions$start <- cys_pos_list
nondisulf_cys_regions$end <- cys_pos_list
nondisulf_cys_regions$wname <- "wcysteines_not_in_disulphide_bridges"
nondisulf_cys_regions$impacts <- "Missense"
nondisulf_cys_regions$layered <- 1

custom_regions <- rbind(disulf_regions,CRD_regions,nondisulf_cys_regions)

withingenednds_custom(gene = "TNFRSF14", 
                      per_gene_cov_dir = per_gene_cov_dir,
                      mutations = mutations,
                      refcds_path = RefCDS_GRCh37_NSX,
                      gene_panel = immune_genes$gene[which(immune_genes$target_type != "Hotspot")],
                      samples = metadata$sample,
                      custom_regions = custom_regions,
                      genomeFile = genomeFile)

# Save output
nbfix_TNFRSF14 <- nbfix_gene
rm(nbfix_gene)

nbfix_TNFRSF14_plot <- nbfix_TNFRSF14[grepl(paste(c("wmis", "wcys", "wdisulphide_bridges_", "wCRD"),collapse="|"), nbfix_TNFRSF14$name),]
nbfix_TNFRSF14_plot$name <- gsub("wdisulphide_bridges_CRD1", "Cys-Cys (CRD1)", nbfix_TNFRSF14_plot$name)
nbfix_TNFRSF14_plot$name <- gsub("wdisulphide_bridges_CRD2", "Cys-Cys (CRD2)", nbfix_TNFRSF14_plot$name)
nbfix_TNFRSF14_plot$name <- gsub("wdisulphide_bridges_CRD3", "Cys-Cys (CRD3)", nbfix_TNFRSF14_plot$name)
nbfix_TNFRSF14_plot$name <- gsub("wCRD1", "Missense (CRD1)", nbfix_TNFRSF14_plot$name)
nbfix_TNFRSF14_plot$name <- gsub("wCRD2", "Missense (CRD2)", nbfix_TNFRSF14_plot$name)
nbfix_TNFRSF14_plot$name <- gsub("wCRD3", "Missense (CRD3)", nbfix_TNFRSF14_plot$name)
nbfix_TNFRSF14_plot$name <- gsub("wmis", "All missense", nbfix_TNFRSF14_plot$name)
nbfix_TNFRSF14_plot$name <- gsub("wcysteines_not_in_disulphide_bridges", "Cys (free)", nbfix_TNFRSF14_plot$name)

nbfix_TNFRSF14_plot$name <- factor(x = nbfix_TNFRSF14_plot$name, levels = c("All missense", "Cys (free)", "Cys-Cys (CRD1)", "Missense (CRD1)", "Cys-Cys (CRD2)", "Missense (CRD2)", "Cys-Cys (CRD3)", "Missense (CRD3)"))

nbfix_plotter(nbfix = nbfix_TNFRSF14_plot, gene = "TNFRSF14", runman = runman, fig.width = 8, fig.height = 8)  
    
 
 
 CD274 withingenedNdS 
  # The disulphide bridges in CD274 were annotated based on:
# CD274: https://www.uniprot.org/uniprotkb/Q9NZQ7/entry 

# Make additional regions file for disulphide cysteines vs other missense mutations in separate cysteine-rich domains
gene = "CD274"
gene_idx <- which(ref_cds_gene_list == gene)

exon_coord <- as.data.frame(RefCDS[[gene_idx]]$intervals_cds)
exon_coord_vec <- NULL
for(x in 1:nrow(exon_coord)){
  exon_coord_vec <- c(exon_coord_vec,exon_coord$V1[x]:exon_coord$V2[x])
}

CD274_ptm <- jsonlite::fromJSON(txt = CD274_ptm_json)$feature
CD274_ptm <- CD274_ptm[CD274_ptm$type == "Disulfide bond",]
CD274_disulf_aa <- sort(unique(c(CD274_ptm$location$start$value,CD274_ptm$location$end$value)))
CD274_disulf_cDNA <- rep(((CD274_disulf_aa - 1) * 3), each = 3) + 1:3
CD274_disulf_pos <- exon_coord_vec[CD274_disulf_cDNA]

# Create customised withingenednds regions_chr for cysteines involved in disulphide bridges
disulf_regions <- as.data.frame(array(data = NA, dim = c(length(CD274_disulf_pos),6)))
colnames(disulf_regions) <- c("chr","start","end","wname","impacts","layered")

disulf_regions$chr <- RefCDS[[gene_idx]]$chr
disulf_regions$start <- CD274_disulf_pos
disulf_regions$end <- CD274_disulf_pos
disulf_regions$wname <- paste0("wdisulphide_bridges")
disulf_regions$impacts <- "Missense"
disulf_regions$layered <- 1

# Create customised withingenednds regions_chr for cysteines not involved in disulphide bridges
cys_residues <- which(seqinr::translate(as.vector(RefCDS[[gene_idx]]$seq_cds), numcode = 1) == "C")
cys_cDNA <- rep(((cys_residues - 1) * 3), each = 3) + 1:3
cys_pos_list <- exon_coord_vec[cys_cDNA]
cys_pos_list <- cys_pos_list[which(!(cys_pos_list %in% CD274_disulf_pos))]

nondisulf_cys_regions <- as.data.frame(array(data = NA, dim = c(length(unlist(cys_pos_list)),6)))
colnames(nondisulf_cys_regions) <- c("chr","start","end","wname","impacts","layered")

nondisulf_cys_regions$chr <- RefCDS[[gene_idx]]$chr
nondisulf_cys_regions$start <- cys_pos_list
nondisulf_cys_regions$end <- cys_pos_list
nondisulf_cys_regions$wname <- "wcysteines_not_in_disulphide_bridges"
nondisulf_cys_regions$impacts <- "Missense"
nondisulf_cys_regions$layered <- 1 

custom_regions <- rbind(disulf_regions,nondisulf_cys_regions)

withingenednds_custom(gene = "CD274", 
                      per_gene_cov_dir = per_gene_cov_dir,
                      mutations = mutations,
                      refcds_path = RefCDS_GRCh37_NSX,
                      gene_panel = immune_genes$gene[which(immune_genes$target_type != "Hotspot")],
                      samples = metadata$sample,
                      custom_regions = custom_regions,
                      genomeFile = genomeFile)

# Save output
nbfix_CD274 <- nbfix_gene
rm(nbfix_gene)

# Modify output for plotting
nbfix_CD274_plot <- nbfix_CD274[grepl(paste(c("wmis", "wdisulphide_bridges", "wcysteines_not_in_disulphide_bridges"),collapse="|"), nbfix_CD274$name),]
nbfix_CD274_plot$name <- gsub("wmis", "All missense", nbfix_CD274_plot$name)
nbfix_CD274_plot$name <- gsub("wdisulphide_bridges", "Cys-Cys", nbfix_CD274_plot$name)
nbfix_CD274_plot$name <- gsub("wcysteines_not_in_disulphide_bridges", "Cys (free)", nbfix_CD274_plot$name)

nbfix_CD274_plot$name <- factor(x = nbfix_CD274_plot$name, levels = c("All missense", "Cys (free)", "Cys-Cys"))

nbfix_plotter(nbfix = nbfix_CD274_plot, gene = "CD274", runman = runman, fig.width = 2.2, fig.height = 4)  
    
 
 
 CBL withingenedNdS 
  # The RING finger and Linker domain of CBL were annotated based on:
# CBL: https://www.uniprot.org/uniprotkb/P22681/entry

# Make additional regions file for RING finger and link domain vs other missense mutations in CBL
gene = "CBL"
gene_idx <- which(ref_cds_gene_list == gene)

exon_coord <- as.data.frame(RefCDS[[gene_idx]]$intervals_cds)
exon_coord_vec <- NULL
for(x in 1:nrow(exon_coord)){
  exon_coord_vec <- c(exon_coord_vec,exon_coord$V1[x]:exon_coord$V2[x])
}

CBL_domains <- jsonlite::fromJSON(txt = CBL_domain_json)$feature
CBL_domains <- CBL_domains[CBL_domains$description %in% c("Linker","RING-type"),]

CBL_RING_aa <- c(min(CBL_domains$location$start$value):max(CBL_domains$location$end$value))
CBL_RING_cDNA <- rep(((CBL_RING_aa - 1) * 3), each = 3) + 1:3
CBL_RING_pos <- exon_coord_vec[CBL_RING_cDNA]

# Create customised withingenednds regions_chr for cysteines involved in disulphide bridges
RING_regions <- as.data.frame(array(data = NA, dim = c(length(CBL_RING_pos),6)))
colnames(RING_regions) <- c("chr","start","end","wname","impacts","layered")

RING_regions$chr <- RefCDS[[gene_idx]]$chr
RING_regions$start <- CBL_RING_pos
RING_regions$end <- CBL_RING_pos
RING_regions$wname <- paste0("wRING_and_linker")
RING_regions$impacts <- "Missense"
RING_regions$layered <- 1

withingenednds_custom(gene = "CBL", 
                      per_gene_cov_dir = per_gene_cov_dir,
                      mutations = mutations,
                      refcds_path = RefCDS_GRCh37_NSX,
                      gene_panel = immune_genes$gene[which(immune_genes$target_type != "Hotspot")],
                      samples = metadata$sample,
                      custom_regions = RING_regions,
                      genomeFile = genomeFile)

# Save output
nbfix_CBL <- nbfix_gene
rm(nbfix_gene)

# Modify output for plotting 
nbfix_CBL_plot <- nbfix_CBL[grepl(paste(c("wmis", "wRING_and_linker"),collapse="|"), nbfix_CBL$name),]
nbfix_CBL_plot$name <- gsub("wmis", "All missense", nbfix_CBL_plot$name)
nbfix_CBL_plot$name <- gsub("wRING_and_linker", "RING-linker", nbfix_CBL_plot$name)

nbfix_CBL_plot$name <- factor(x = nbfix_CBL_plot$name, levels = c("All missense", "RING-linker"))

nbfix_plotter(nbfix = nbfix_CBL_plot, gene = "CBL", runman = runman, fig.width = 2, fig.height = 5.5)  
    
 
 
 CCR6 withingenedNdS 
  # Nonsense mutations in the final exon of CCR6 was performed without customised input (default inclusion in withingenednds as wnonlastex)
withingenednds_custom(gene = "CCR6",
                      per_gene_cov_dir = per_gene_cov_dir,
                      mutations = mutations,
                      refcds_path = RefCDS_GRCh37_NSX,
                      gene_panel = immune_genes$gene[which(immune_genes$target_type != "Hotspot")],
                      samples = metadata$sample,
                      custom_regions = NULL,
                      genomeFile = genomeFile)

# Save output
nbfix_CCR6 <- nbfix_gene
rm(nbfix_gene)

# Modify output for plotting (CCR6 has a single coding exon, so wnon produces NA unlike wnonlastex)
nbfix_CCR6_plot <- nbfix_CCR6[grepl(paste(c("wmis", "wnonlastex"),collapse="|"), nbfix_CCR6$name),]
nbfix_CCR6_plot$name <- gsub("wmis", "All missense", nbfix_CCR6_plot$name)
nbfix_CCR6_plot$name <- gsub("wnonlastex", "Nonsense last exon", nbfix_CCR6_plot$name)
nbfix_CCR6_plot$name <- factor(x = nbfix_CCR6_plot$name, levels = c("All missense", "Nonsense last exon"))

nbfix_plotter(nbfix = nbfix_CCR6_plot, gene = "CCR6", runman = runman, fig.width = 2, fig.height = 5.5)  
    
 
 
 
 13. Pairwise comparison between Hashimoto and Graves samples 
  genes2compare <- immune_genes$gene[which(immune_genes$target_type != "Hotspot")]
genes2plot <- c(all_drivers,"RLTPR")
hashi_bulk_IDs <- metadata$sample[which(metadata$pathology == "Hashimoto" & metadata$sample_type == "Bulk")]
graves_bulk_IDs <- metadata$sample[which(metadata$pathology == "Graves" & metadata$sample_type == "Bulk")]

sig_shm_genes <- all_dnds_shm$sel_merged$gene_name[which((all_dnds_shm$sel_merged$qglobalpos_cv > 0.1 | is.na(all_dnds_shm$sel_merged$qglobalpos_cv)) & all_dnds_shm$sel_merged$qpos_loc < 0.1 & all_dnds_shm$sel_merged$gene_name %in% genes2plot)]
lineage_defining_genes <- "TG"

highlight_colour <- vector()

for(y in 1:length(genes2plot)){
  if(genes2plot[y] %in% sig_shm_genes){
    highlight_colour[y] <- "grey"
  }else if(genes2plot[y] %in% lineage_defining_genes){
    highlight_colour[y] <- "blue"
  }else{
    highlight_colour[y] <- "black"
  }
}
names(highlight_colour) <- genes2plot

driverdensity_sampleIDs <- vector("list", 2)
full_sampleIDs <- vector("list", 2)
combined_targeted_only_dnds_sel_cv <- vector("list",2)
plottitles <- vector("list",2)
temp_muts <- vector("list",2)
unique_temp_muts <- vector("list",2)
temp_cov <- vector("list",2)
dnds_out <- vector("list",2)
pairwise_dnds <- vector("list",2)

driverdensity_sampleIDs[[1]] <- hashi_bulk_IDs
driverdensity_sampleIDs[[2]] <- graves_bulk_IDs

full_sampleIDs[[1]] <- hashi_bulk_IDs
full_sampleIDs[[2]] <- graves_bulk_IDs

plottitles[[1]] <- "Hashimoto bulk samples"
plottitles[[2]] <- "Graves bulk samples"

input_muts <- mutations[,!(colnames(mutations) == "paper_id")]
input_muts$cell_type <- "aitd"

# Run dNdS on Hashimoto bulk samples alone
sample_list <- hashi_bulk_IDs

dnds_muts <- mutations[which(mutations$sampleID %in% sample_list),]
dnds_muts <- dnds_muts[which(!(duplicated(dnds_muts$mut_site))),]

dnds_cov <- rowSums(coverage[which(colnames(coverage) %in% sample_list)])
names(dnds_cov) <- coverage$gene
dnds_cov <- dnds_cov[which(dnds_cov != 0)]
dnds_cov <- dnds_cov[which(!(names(dnds_cov) %in% c("IGLL5","IGLL1")))]
dnds_cov <- dnds_cov[which(names(dnds_cov) %in% immune_genes$gene[which(immune_genes$target_type != "Hotspot")])]

hashimoto_bulk_dnds_shm <- dnds_shm(mutations = dnds_muts[,c("sampleID","chr","pos","ref","mut")],
                                    refdb_noshm = RefCDS_noshm_exome,
                                    refdb_shm = RefCDS_shm_exome,
                                    dc_noshm = dnds_cov,
                                    gene_list = names(dnds_cov),
                                    # Exons with just the start codon need to be excluded (synonymous mutations are not possible for dNdSloc)
                                    excl_shm = c("IGLL1","IGLL5","MYO1E","ZNF595","AC026202.1","EHBP1L1"),
                                    excl_noshm = c("IGLL1","IGLL5"),
                                    onesided = T,
                                    maxcovs = 10)

# Run dNdS on Graves bulk samples alone
sample_list <- graves_bulk_IDs

dnds_muts <- mutations[which(mutations$sampleID %in% sample_list),]
dnds_muts <- dnds_muts[which(!(duplicated(dnds_muts$mut_site))),]

dnds_cov <- rowSums(coverage[which(colnames(coverage) %in% sample_list)])
names(dnds_cov) <- coverage$gene
dnds_cov <- dnds_cov[which(dnds_cov != 0)]
dnds_cov <- dnds_cov[which(!(names(dnds_cov) %in% c("IGLL5","IGLL1")))]
dnds_cov <- dnds_cov[which(names(dnds_cov) %in% immune_genes$gene[which(immune_genes$target_type != "Hotspot")])]

grave_bulk_dnds_shm <- dnds_shm(mutations = dnds_muts[,c("sampleID","chr","pos","ref","mut")],
                                    refdb_noshm = RefCDS_noshm_exome,
                                    refdb_shm = RefCDS_shm_exome,
                                    dc_noshm = dnds_cov,
                                    gene_list = names(dnds_cov),
                                    # Exons with just the start codon need to be excluded (synonymous mutations are not possible for dNdSloc)
                                    excl_shm = c("IGLL1","IGLL5","MYO1E","ZNF595","AC026202.1","EHBP1L1"),
                                    excl_noshm = c("IGLL1","IGLL5"),
                                    onesided = T,
                                    maxcovs = 10)

combined_targeted_only_dnds_sel_cv[[1]] <- hashimoto_bulk_dnds_shm$sel_merged
combined_targeted_only_dnds_sel_cv[[2]] <- grave_bulk_dnds_shm$sel_merged

input_cov <- coverage[coverage$gene %in% immune_genes$gene[which(immune_genes$target_type != "Hotspot")],]

for(j in 1:2){
  temp_muts[[j]] <- input_muts[which(input_muts$sampleID %in% driverdensity_sampleIDs[[j]]),]
  unique_temp_muts[[j]] <- temp_muts[[j]][which(!(duplicated(temp_muts[[j]]$mut_site))),]
  temp_cov[[j]] <- rowSums(input_cov[which(colnames(input_cov) %in% driverdensity_sampleIDs[[j]])])
  names(temp_cov[[j]]) <- input_cov$gene
  temp_cov[[j]] <- temp_cov[[j]][which(temp_cov[[j]] != 0)]
  temp_cov[[j]] <- temp_cov[[j]][which(!(names(temp_cov[[j]]) %in% c("IGLL1","IGLL5")))]
  
  # NB - Need to run dNdS again as may restrict to different donor list (i.e. individuals aged ≥50)
  # NB - Discuss with Iñigo - pairwise dNdS uses regular dNdS rather than dNdSshm (as genes with nothing but SHM excluded from gene_muts such as LTB)
  dnds_temp <- dndscv(mutations = unique_temp_muts[[j]][,c(1:5)], gene_list = names(temp_cov[[j]]), max_muts_per_gene_per_sample = Inf, max_coding_muts_per_sample = Inf, onesided = T, dc = temp_cov[[j]], refdb = RefCDS_GRCh37_NSX, maxcovs = 10)
  
  kc <- as.vector(dnds_temp$sel_cv$gene_name[which(dnds_temp$sel_cv$qallsubs_cv<0.01)])
  
  dnds_out[[j]] <- dndscv(mutations = unique_temp_muts[[j]][,c(1:5)], gene_list = names(temp_cov[[j]]), max_muts_per_gene_per_sample = Inf, max_coding_muts_per_sample = Inf, onesided = T, dc = temp_cov[[j]], refdb = RefCDS_GRCh37_NSX, kc = kc, maxcovs = 10)
}

pairwise_dnds[[1]] <- pairwise_dNdScv(dnds_out[[1]], dnds_out[[2]], genestotest = genes2compare)
pairwise_dnds[[2]] <- pairwise_dNdScv(dnds_out[[2]], dnds_out[[1]], genestotest = genes2compare)

pairwise_dnds[[1]] <- pairwise_dnds[[1]][pairwise_dnds[[1]]$gene_name %in% genes2plot,]
pairwise_dnds[[2]] <- pairwise_dnds[[2]][pairwise_dnds[[2]]$gene_name %in% genes2plot,]

cell_frac_comp = compplot(
  comp_list = list(c("Hashimoto","Graves")),
  genes2plot = list(genes2plot),
  highlight_colour = list(highlight_colour),
  driverdensity_sampleIDs = list(driverdensity_sampleIDs),
  full_sampleIDs = list(full_sampleIDs),
  muts = input_muts, 
  combined_targeted_only_dnds_sel_cv = list(combined_targeted_only_dnds_sel_cv),
  targeted_dnds_sel_cv = NULL,
  gene2dc_array = input_cov,
  max_duplex_cov = 160000,
  min_mcf_upper = 4.5,
  pairwise_dnds = list(pairwise_dnds),
  runman = runman,
  plottitles = list(plottitles),
  plotfilename = "output/Hashi_Graves_PairwiseComparison.pdf",
  plotwidth = 8,
  plotheight = 8)  
    
 
 
 14. Comparison to normal lymphocytes 
 
 Load normal lymphocyte data 
  normal_muts <- read.table(normal_mut_file, sep = "\t", stringsAsFactors = F, header = T)
normal_cov <- read.table(normal_cov_file, sep = "\t", stringsAsFactors = F, header = T)
normal_metadata <- read.table(normal_metadata_file, sep = "\t", stringsAsFactors = F, header = T)  
  normal_muts$mut_site <- paste(normal_muts$donor,normal_muts$chr,normal_muts$pos,normal_muts$ref,normal_muts$mut,sep = "_")

normal_males <- normal_metadata$sample[which(normal_metadata$sex == "Male")]
normal_muts$cellfraction = normal_muts$duplex_vaf * 2
normal_muts$cellfraction[normal_muts$chr %in% c("X","Y") & normal_muts$sampleID %in% normal_males] = normal_muts$duplex_vaf[normal_muts$chr %in% c("X","Y") & normal_muts$sampleID %in% normal_males]

normal_muts$bam_mut_adj = pmax(0,normal_muts$bam_mut-normal_muts$times_called)
normal_muts$bam_cov_adj = normal_muts$bam_cov-normal_muts$duplex_cov
normal_muts$bam_vaf_adj = normal_muts$bam_mut_adj / normal_muts$bam_cov_adj
normal_muts$bam_adj_cellfraction = normal_muts$bam_vaf_adj * 2
normal_muts$bam_adj_cellfraction[normal_muts$chr %in% c("X","Y") & normal_muts$sampleID %in% normal_males] = normal_muts$bam_vaf_adj[normal_muts$chr %in% c("X","Y") & normal_muts$sampleID %in% normal_males]  
 
 
 Run pairwise comparison between normal lymphocyte data and
thyroid-infiltrating lymphocytes 
  # Limit to individuals aged >50 in normal lymphocyte cohort due to large age range
# This makes identification of disease-specific genes very stringent 
# (i.e. higher MCF in disease than pure memory cell populations of older individuals)
sorted_blood_age_cutoff <- 50
lymphocyte_fraction <- 0.25
genes2compare <- immune_genes$gene[which(immune_genes$target_type != "Hotspot")]
genes2plot <- c(all_drivers,"RLTPR")

# Comparisons (for bulk, all lymphocyte rich)
# 1. Bulk AITD (>25% lymphocytes) vs PBMC memory B cells  (main)
# 2. Bulk AITD (>25% lymphocytes) vs PBMC memory CD4+ T cells (supp)
# 3. Bulk AITD (>25% lymphocytes) vs PBMC memory CD8+ T cells (supp)
comp_list <- c("B_mem","T_CD4_mem","T_CD8_mem")

# Limit to bulk (not LCM) and samples with lymphocyte fraction >25% in EMseq
aitd_bulk_lymph_IDs <- metadata$sample[metadata$sample_type == "Bulk" & substr(metadata$sample,1,8) %in% substr(emseq$sampleID[(emseq$Blood_B + emseq$Blood_T) > lymphocyte_fraction & emseq$sampleID %in% substr(metadata$sample[metadata$sample_type == "Bulk"],1,16)],1,8)]

for(x in 1:length(comp_list)){
  normal_celltype_IDs <- normal_metadata$sample[grepl(comp_list[x],normal_metadata$cell_type)]
  normal_celltype_age_IDs <- normal_metadata$sample[grepl(comp_list[x],normal_metadata$cell_type) & normal_metadata$age >= sorted_blood_age_cutoff]
  
  sample_list <- normal_celltype_IDs
  
  dnds_muts <- normal_muts[which(normal_muts$sampleID %in% sample_list),]
  dnds_muts <- dnds_muts[which(!(duplicated(dnds_muts$mut_site))),]
  
  dnds_cov <- rowSums(normal_cov[which(colnames(normal_cov) %in% sample_list)])
  names(dnds_cov) <- normal_cov$gene
  dnds_cov <- dnds_cov[which(dnds_cov != 0)]
  
  # 2024-02-19 - Made decision to exclude IGLL5 and IGLL1 from dNdS analysis. Only reason these Ig genes (but not others) are in the dNdS object is they are in frame.
  dnds_cov <- dnds_cov[which(!(names(dnds_cov) %in% c("IGLL5","IGLL1")))]
  
  # Restrict dN/dS analysis to genes in targeted panel
  dnds_cov <- dnds_cov[which(names(dnds_cov) %in% immune_genes$gene[which(immune_genes$target_type != "Hotspot")])]
  
  if(comp_list[x] == "B_mem"){
    normal_dnds_shm <- dnds_shm(mutations = dnds_muts[,c("sampleID","chr","pos","ref","mut")],
                                refdb_noshm = RefCDS_noshm_exome,
                                refdb_shm = RefCDS_shm_exome,
                                dc_noshm = dnds_cov,
                                gene_list = names(dnds_cov),
                                # Exons with just the start codon need to be excluded (synonymous mutations are not possible for dNdSloc)
                                excl_shm = c("IGLL1","IGLL5","MYO1E","ZNF595","AC026202.1","EHBP1L1"),
                                excl_noshm = c("IGLL1","IGLL5"),
                                onesided = T,
                                maxcovs = 10)
  }else{
    normal_dnds_cv <- dndscv(mutations = dnds_muts[,c("sampleID","chr","pos","ref","mut")], gene_list = names(dnds_cov), refdb = RefCDS_GRCh37_NSX, max_muts_per_gene_per_sample = Inf, max_coding_muts_per_sample = Inf, maxcovs = 10, onesided = T, dc = dnds_cov)
    
    normal_kc <- normal_dnds_cv$sel_cv$gene_name[normal_dnds_cv$sel_cv$qsubpos_cv < 0.01]
    
    normal_dnds_cv <- dndscv(mutations = dnds_muts[,c("sampleID","chr","pos","ref","mut")], gene_list = names(dnds_cov), refdb = RefCDS_GRCh37_NSX, max_muts_per_gene_per_sample = Inf, max_coding_muts_per_sample = Inf, maxcovs = 10, onesided = T, dc = dnds_cov, kc = normal_kc)
  }
  
  sig_shm_genes <- all_dnds_shm$sel_merged$gene_name[which((all_dnds_shm$sel_merged$qglobalpos_cv > 0.1 | is.na(all_dnds_shm$sel_merged$qglobalpos_cv)) & all_dnds_shm$sel_merged$qpos_loc < 0.1 & all_dnds_shm$sel_merged$gene_name %in% genes2plot)]
  lineage_defining_genes <- "TG"
  
  highlight_colour <- vector()
  
  for(y in 1:length(genes2plot)){
    if(genes2plot[y] %in% sig_shm_genes){
      highlight_colour[y] <- "grey"
    }else if(genes2plot[y] %in% lineage_defining_genes){
      highlight_colour[y] <- "blue"
    }else{
      highlight_colour[y] <- "black"
    }
  }
  names(highlight_colour) <- genes2plot
  
  driverdensity_sampleIDs <- vector("list", 2)
  full_sampleIDs <- vector("list", 2)
  combined_targeted_only_dnds_sel_cv <- vector("list",2)
  plottitles <- vector("list",2)
  temp_muts <- vector("list",2)
  unique_temp_muts <- vector("list",2)
  temp_cov <- vector("list",2)
  dnds_out <- vector("list",2)
  pairwise_dnds <- vector("list",2)
  
  driverdensity_sampleIDs[[1]] <- aitd_bulk_lymph_IDs
  driverdensity_sampleIDs[[2]] <- normal_celltype_age_IDs
  
  full_sampleIDs[[1]] <- metadata$sample
  full_sampleIDs[[2]] <- normal_celltype_IDs
  
  plottitles[[1]] <- paste0("Autoimmune thyroid\n(>",lymphocyte_fraction * 100,"% lymphocyte fraction)")
  plottitles[[2]] <- paste0("Normal PBMCs - ",comp_list[x],"\n(Individuals aged >",sorted_blood_age_cutoff,")")
  
  input_muts <- mutations[,!(colnames(mutations) == "paper_id")]
  input_muts$cell_type <- "aitd"
  input_muts <- rbind(input_muts,normal_muts[,colnames(input_muts)])
  
  combined_targeted_only_dnds_sel_cv[[1]] <- all_dnds_shm$sel_merged
  
  if(comp_list[x] == "B_mem"){
    combined_targeted_only_dnds_sel_cv[[2]] <- normal_dnds_shm$sel_merged
  }else{
    combined_targeted_only_dnds_sel_cv[[2]] <- normal_dnds_cv$sel_cv
  }
  
  input_cov <- coverage[coverage$gene %in% immune_genes$gene[which(immune_genes$target_type != "Hotspot")],]
  input_cov_2 <- normal_cov[normal_cov$gene %in% immune_genes$gene[which(immune_genes$target_type != "Hotspot")],]
  rownames(input_cov_2) <- input_cov_2$gene
  input_cov <- cbind(input_cov,input_cov_2[input_cov$gene,!(colnames(input_cov_2) == "gene")])
  
  for(j in 1:2){
    temp_muts[[j]] <- input_muts[which(input_muts$sampleID %in% driverdensity_sampleIDs[[j]]),]
    unique_temp_muts[[j]] <- temp_muts[[j]][which(!(duplicated(temp_muts[[j]]$mut_site))),]
    temp_cov[[j]] <- rowSums(input_cov[which(colnames(input_cov) %in% driverdensity_sampleIDs[[j]])])
    names(temp_cov[[j]]) <- input_cov$gene
    temp_cov[[j]] <- temp_cov[[j]][which(temp_cov[[j]] != 0)]
    temp_cov[[j]] <- temp_cov[[j]][which(!(names(temp_cov[[j]]) %in% c("IGLL1","IGLL5")))]
    
    # NB - Need to run dNdS again as may restrict to different donor list (i.e. individuals aged ≥50)
    # NB - Discuss with Iñigo - pairwise dNdS uses regular dNdS rather than dNdSshm (as genes with nothing but SHM excluded from gene_muts such as LTB)
    dnds_temp <- dndscv(mutations = unique_temp_muts[[j]][,c(1:5)], gene_list = names(temp_cov[[j]]), max_muts_per_gene_per_sample = Inf, max_coding_muts_per_sample = Inf, onesided = T, dc = temp_cov[[j]], refdb = RefCDS_GRCh37_NSX, maxcovs = 10)
    
    kc <- as.vector(dnds_temp$sel_cv$gene_name[which(dnds_temp$sel_cv$qallsubs_cv<0.01)])
    
    dnds_out[[j]] <- dndscv(mutations = unique_temp_muts[[j]][,c(1:5)], gene_list = names(temp_cov[[j]]), max_muts_per_gene_per_sample = Inf, max_coding_muts_per_sample = Inf, onesided = T, dc = temp_cov[[j]], refdb = RefCDS_GRCh37_NSX, kc = kc, maxcovs = 10)
  }
  
  pairwise_dnds[[1]] <- pairwise_dNdScv(dnds_out[[1]], dnds_out[[2]], genestotest = genes2compare)
  pairwise_dnds[[2]] <- pairwise_dNdScv(dnds_out[[2]], dnds_out[[1]], genestotest = genes2compare)
  
  pairwise_dnds[[1]] <- pairwise_dnds[[1]][pairwise_dnds[[1]]$gene_name %in% genes2plot,]
  pairwise_dnds[[2]] <- pairwise_dnds[[2]][pairwise_dnds[[2]]$gene_name %in% genes2plot,]
  
  cell_frac_comp = compplot(
    comp_list = list(c("aitd",comp_list[x])),
    genes2plot = list(genes2plot),
    highlight_colour = list(highlight_colour),
    driverdensity_sampleIDs = list(driverdensity_sampleIDs),
    full_sampleIDs = list(full_sampleIDs),
    muts = input_muts, 
    combined_targeted_only_dnds_sel_cv = list(combined_targeted_only_dnds_sel_cv),
    targeted_dnds_sel_cv = NULL,
    gene2dc_array = input_cov,
    max_duplex_cov = 160000,
    min_mcf_upper = 4.5,
    pairwise_dnds = list(pairwise_dnds),
    runman = runman,
    plottitles = list(plottitles),
    plotfilename = paste0("output/Fig3D_EDF4_NonSynonymousMutantFractionComparison_HashimotoLymphocyteRich_NormalPBMC_",comp_list[x],".pdf"),
    plotwidth = 8,
    plotheight = 8)
}  
     
 
 
 
 15. Comparison to other control samples 
 
 Load other control datasets 
  lcm_lymphocyte_muts <- read.table(lcm_lymphocyte_mut_file, sep = "\t", stringsAsFactors = F, header = T)
lcm_lymphocyte_cov <- read.table(lcm_lymphocyte_cov_file, sep = "\t", stringsAsFactors = F, header = T)
lcm_lymphocyte_metadata <- read.table(lcm_lymphocyte_metadata_file, sep = "\t", stringsAsFactors = F, header = T)

lymph_node_muts <- read.table(lymph_node_mut_file, sep = "\t", stringsAsFactors = F, header = T)
lymph_node_cov <- read.table(lymph_node_cov_file, sep = "\t", stringsAsFactors = F, header = T)
lymph_node_metadata <- read.table(lymph_node_metadata_file, sep = "\t", stringsAsFactors = F, header = T)

spleen_muts <- read.table(spleen_mut_file, sep = "\t", stringsAsFactors = F, header = T)
spleen_cov <- read.table(spleen_cov_file, sep = "\t", stringsAsFactors = F, header = T)
spleen_metadata <- read.table(spleen_metadata_file, sep = "\t", stringsAsFactors = F, header = T)

tonsillitis_muts <- read.table(tonsillitis_mut_file, sep = "\t", stringsAsFactors = F, header = T)
tonsillitis_cov <- read.table(tonsillitis_cov_file, sep = "\t", stringsAsFactors = F, header = T)
tonsillitis_metadata <- read.table(tonsillitis_metadata_file, sep = "\t", stringsAsFactors = F, header = T)

thyroid_goitre_muts <- read.table(thyroid_goitre_mut_file, sep = "\t", stringsAsFactors = F, header = T)
thyroid_goitre_cov <- read.table(thyroid_goitre_cov_file, sep = "\t", stringsAsFactors = F, header = T)
thyroid_goitre_metadata <- read.table(thyroid_goitre_metadata_file, sep = "\t", stringsAsFactors = F, header = T)  
  lcm_lymphocyte_muts$donor <- substr(lcm_lymphocyte_muts$sampleID,1,7)
lcm_lymphocyte_muts$mut_site <- paste(lcm_lymphocyte_muts$donor,lcm_lymphocyte_muts$chr,lcm_lymphocyte_muts$pos,lcm_lymphocyte_muts$ref,lcm_lymphocyte_muts$mut,sep = "_")
lcm_lymphocyte_males <- lcm_lymphocyte_metadata$sample[which(lcm_lymphocyte_metadata$sex == "Male")]
lcm_lymphocyte_muts$cellfraction = lcm_lymphocyte_muts$duplex_vaf * 2
lcm_lymphocyte_muts$cellfraction[lcm_lymphocyte_muts$chr %in% c("X","Y") & lcm_lymphocyte_muts$sampleID %in% lcm_lymphocyte_males] = lcm_lymphocyte_muts$duplex_vaf[lcm_lymphocyte_muts$chr %in% c("X","Y") & lcm_lymphocyte_muts$sampleID %in% lcm_lymphocyte_males]

lcm_lymphocyte_muts$bam_mut_adj = pmax(0,lcm_lymphocyte_muts$bam_mut-lcm_lymphocyte_muts$times_called)
lcm_lymphocyte_muts$bam_cov_adj = lcm_lymphocyte_muts$bam_cov-lcm_lymphocyte_muts$duplex_cov
lcm_lymphocyte_muts$bam_vaf_adj = lcm_lymphocyte_muts$bam_mut_adj / lcm_lymphocyte_muts$bam_cov_adj
lcm_lymphocyte_muts$bam_adj_cellfraction = lcm_lymphocyte_muts$bam_vaf_adj * 2
lcm_lymphocyte_muts$bam_adj_cellfraction[lcm_lymphocyte_muts$chr %in% c("X","Y") & lcm_lymphocyte_muts$sampleID %in% lcm_lymphocyte_males] = lcm_lymphocyte_muts$bam_vaf_adj[lcm_lymphocyte_muts$chr %in% c("X","Y") & lcm_lymphocyte_muts$sampleID %in% lcm_lymphocyte_males]

lymph_node_muts$donor <- substr(lymph_node_muts$sampleID,1,7)
lymph_node_muts$mut_site <- paste(lymph_node_muts$donor,lymph_node_muts$chr,lymph_node_muts$pos,lymph_node_muts$ref,lymph_node_muts$mut,sep = "_")
lymph_node_males <- lymph_node_metadata$sample[which(lymph_node_metadata$sex == "Male")]
lymph_node_muts$cellfraction = lymph_node_muts$duplex_vaf * 2
lymph_node_muts$cellfraction[lymph_node_muts$chr %in% c("X","Y") & lymph_node_muts$sampleID %in% lymph_node_males] = lymph_node_muts$duplex_vaf[lymph_node_muts$chr %in% c("X","Y") & lymph_node_muts$sampleID %in% lymph_node_males]

lymph_node_muts$bam_mut_adj = pmax(0,lymph_node_muts$bam_mut-lymph_node_muts$times_called)
lymph_node_muts$bam_cov_adj = lymph_node_muts$bam_cov-lymph_node_muts$duplex_cov
lymph_node_muts$bam_vaf_adj = lymph_node_muts$bam_mut_adj / lymph_node_muts$bam_cov_adj
lymph_node_muts$bam_adj_cellfraction = lymph_node_muts$bam_vaf_adj * 2
lymph_node_muts$bam_adj_cellfraction[lymph_node_muts$chr %in% c("X","Y") & lymph_node_muts$sampleID %in% lymph_node_males] = lymph_node_muts$bam_vaf_adj[lymph_node_muts$chr %in% c("X","Y") & lymph_node_muts$sampleID %in% lymph_node_males]

spleen_muts$donor <- substr(spleen_muts$sampleID,1,7)
spleen_muts$mut_site <- paste(spleen_muts$donor,spleen_muts$chr,spleen_muts$pos,spleen_muts$ref,spleen_muts$mut,sep = "_")
spleen_males <- spleen_metadata$sample[which(spleen_metadata$sex == "Male")]
spleen_muts$cellfraction = spleen_muts$duplex_vaf * 2
spleen_muts$cellfraction[spleen_muts$chr %in% c("X","Y") & spleen_muts$sampleID %in% spleen_males] = spleen_muts$duplex_vaf[spleen_muts$chr %in% c("X","Y") & spleen_muts$sampleID %in% spleen_males]

spleen_muts$bam_mut_adj = pmax(0,spleen_muts$bam_mut-spleen_muts$times_called)
spleen_muts$bam_cov_adj = spleen_muts$bam_cov-spleen_muts$duplex_cov
spleen_muts$bam_vaf_adj = spleen_muts$bam_mut_adj / spleen_muts$bam_cov_adj
spleen_muts$bam_adj_cellfraction = spleen_muts$bam_vaf_adj * 2
spleen_muts$bam_adj_cellfraction[spleen_muts$chr %in% c("X","Y") & spleen_muts$sampleID %in% spleen_males] = spleen_muts$bam_vaf_adj[spleen_muts$chr %in% c("X","Y") & spleen_muts$sampleID %in% spleen_males]

tonsillitis_muts$donor <- substr(tonsillitis_muts$sampleID,1,7)
tonsillitis_muts$mut_site <- paste(tonsillitis_muts$donor,tonsillitis_muts$chr,tonsillitis_muts$pos,tonsillitis_muts$ref,tonsillitis_muts$mut,sep = "_")
tonsillitis_males <- tonsillitis_metadata$sample[which(tonsillitis_metadata$sex == "Male")]
tonsillitis_muts$cellfraction = tonsillitis_muts$duplex_vaf * 2
tonsillitis_muts$cellfraction[tonsillitis_muts$chr %in% c("X","Y") & tonsillitis_muts$sampleID %in% tonsillitis_males] = tonsillitis_muts$duplex_vaf[tonsillitis_muts$chr %in% c("X","Y") & tonsillitis_muts$sampleID %in% tonsillitis_males]

tonsillitis_muts$bam_mut_adj = pmax(0,tonsillitis_muts$bam_mut-tonsillitis_muts$times_called)
tonsillitis_muts$bam_cov_adj = tonsillitis_muts$bam_cov-tonsillitis_muts$duplex_cov
tonsillitis_muts$bam_vaf_adj = tonsillitis_muts$bam_mut_adj / tonsillitis_muts$bam_cov_adj
tonsillitis_muts$bam_adj_cellfraction = tonsillitis_muts$bam_vaf_adj * 2
tonsillitis_muts$bam_adj_cellfraction[tonsillitis_muts$chr %in% c("X","Y") & tonsillitis_muts$sampleID %in% tonsillitis_males] = tonsillitis_muts$bam_vaf_adj[tonsillitis_muts$chr %in% c("X","Y") & tonsillitis_muts$sampleID %in% tonsillitis_males]

thyroid_goitre_muts$donor <- substr(thyroid_goitre_muts$sampleID,1,7)
thyroid_goitre_muts$mut_site <- paste(thyroid_goitre_muts$donor,thyroid_goitre_muts$chr,thyroid_goitre_muts$pos,thyroid_goitre_muts$ref,thyroid_goitre_muts$mut,sep = "_")
thyroid_goitre_males <- thyroid_goitre_metadata$sample[which(thyroid_goitre_metadata$sex == "Male")]
thyroid_goitre_muts$cellfraction = thyroid_goitre_muts$duplex_vaf * 2
thyroid_goitre_muts$cellfraction[thyroid_goitre_muts$chr %in% c("X","Y") & thyroid_goitre_muts$sampleID %in% thyroid_goitre_males] = thyroid_goitre_muts$duplex_vaf[thyroid_goitre_muts$chr %in% c("X","Y") & thyroid_goitre_muts$sampleID %in% thyroid_goitre_males]

thyroid_goitre_muts$bam_mut_adj = pmax(0,thyroid_goitre_muts$bam_mut-thyroid_goitre_muts$times_called)
thyroid_goitre_muts$bam_cov_adj = thyroid_goitre_muts$bam_cov-thyroid_goitre_muts$duplex_cov
thyroid_goitre_muts$bam_vaf_adj = thyroid_goitre_muts$bam_mut_adj / thyroid_goitre_muts$bam_cov_adj
thyroid_goitre_muts$bam_adj_cellfraction = thyroid_goitre_muts$bam_vaf_adj * 2
thyroid_goitre_muts$bam_adj_cellfraction[thyroid_goitre_muts$chr %in% c("X","Y") & thyroid_goitre_muts$sampleID %in% thyroid_goitre_males] = thyroid_goitre_muts$bam_vaf_adj[thyroid_goitre_muts$chr %in% c("X","Y") & thyroid_goitre_muts$sampleID %in% thyroid_goitre_males]  
 
 
 Run pairwise comparison between other control sample data and
thyroid-infiltrating lymphocytes 
  lymphocyte_fraction <- 0.25
genes2compare <- immune_genes$gene[which(immune_genes$target_type != "Hotspot" & immune_genes$chr != "Y")]
genes2plot <- c(all_drivers,"RLTPR")

# Comparisons (for bulk, all lymphocyte rich)
# 1. Bulk AITD (>25% lymphocytes) vs LCM lymphocytes
# 2. Bulk AITD (>25% lymphocytes) vs lymph nodes
# 3. Bulk AITD (>25% lymphocytes) vs spleen
# 4. Bulk AITD (>25% lymphocytes) vs tonsillitis
# 5. Bulk AITD (>25% lymphocytes) vs thyroid goitre
comp_list <- c("lcm_lymphocyte","lymph_node","spleen","tonsillitis","thyroid_goitre")
plot_titles <- c("LCM lymphocyte aggregates","Lymph node","Spleen","Tonsillitis","Thyroid goitre")

# Limit to bulk (not LCM) and samples with lymphocyte fraction >25% in EMseq
aitd_bulk_lymph_IDs <- metadata$sample[metadata$sample_type == "Bulk" & substr(metadata$sample,1,8) %in% substr(emseq$sampleID[(emseq$Blood_B + emseq$Blood_T) > lymphocyte_fraction & emseq$sampleID %in% substr(metadata$sample[metadata$sample_type == "Bulk"],1,16)],1,8)]

for(x in 1:length(comp_list)){
  other_normal_muts <- eval(parse(text = paste0(comp_list[x],"_muts")))
  other_normal_metadata <- eval(parse(text = paste0(comp_list[x],"_metadata")))
  other_normal_cov <- eval(parse(text = paste0(comp_list[x],"_cov")))
  
  other_normal_IDs <- other_normal_metadata$sample
  
  sample_list <- other_normal_IDs
  
  dnds_muts <- other_normal_muts[which(other_normal_muts$sampleID %in% sample_list),]
  dnds_muts <- dnds_muts[which(!(duplicated(dnds_muts$mut_site))),]
  
  dnds_cov <- rowSums(other_normal_cov[which(colnames(other_normal_cov) %in% sample_list)])
  names(dnds_cov) <- other_normal_cov$gene
  dnds_cov <- dnds_cov[which(dnds_cov != 0)]
  
  # 2024-02-19 - Made decision to exclude IGLL5 and IGLL1 from dNdS analysis. Only reason these Ig genes (but not others) are in the dNdS object is they are in frame.
  dnds_cov <- dnds_cov[which(!(names(dnds_cov) %in% c("IGLL5","IGLL1")))]
  
  # Restrict dN/dS analysis to genes in targeted panel
  dnds_cov <- dnds_cov[which(names(dnds_cov) %in% immune_genes$gene[which(immune_genes$target_type != "Hotspot")])]
  
  other_normal_dnds_shm <- dnds_shm(mutations = dnds_muts[,c("sampleID","chr","pos","ref","mut")],
                                    refdb_noshm = RefCDS_noshm_exome,
                                    refdb_shm = RefCDS_shm_exome,
                                    dc_noshm = dnds_cov,
                                    gene_list = names(dnds_cov),
                                    # Exons with just the start codon need to be excluded (synonymous mutations are not possible for dNdSloc)
                                    excl_shm = c("IGLL1","IGLL5","MYO1E","ZNF595","AC026202.1","EHBP1L1"),
                                    excl_noshm = c("IGLL1","IGLL5"),
                                    onesided = T,
                                    maxcovs = 10)
  
  sig_shm_genes <- all_dnds_shm$sel_merged$gene_name[which((all_dnds_shm$sel_merged$qglobalpos_cv > 0.1 | is.na(all_dnds_shm$sel_merged$qglobalpos_cv)) & all_dnds_shm$sel_merged$qpos_loc < 0.1 & all_dnds_shm$sel_merged$gene_name %in% genes2plot)]
  lineage_defining_genes <- "TG"
  
  highlight_colour <- vector()
  
  for(y in 1:length(genes2plot)){
    if(genes2plot[y] %in% sig_shm_genes){
      highlight_colour[y] <- "grey"
    }else if(genes2plot[y] %in% lineage_defining_genes){
      highlight_colour[y] <- "blue"
    }else{
      highlight_colour[y] <- "black"
    }
  }
  names(highlight_colour) <- genes2plot
  
  driverdensity_sampleIDs <- vector("list", 2)
  full_sampleIDs <- vector("list", 2)
  combined_targeted_only_dnds_sel_cv <- vector("list",2)
  plottitles <- vector("list",2)
  temp_muts <- vector("list",2)
  unique_temp_muts <- vector("list",2)
  temp_cov <- vector("list",2)
  dnds_out <- vector("list",2)
  pairwise_dnds <- vector("list",2)
  
  driverdensity_sampleIDs[[1]] <- aitd_bulk_lymph_IDs
  driverdensity_sampleIDs[[2]] <- other_normal_IDs
  
  full_sampleIDs[[1]] <- metadata$sample
  full_sampleIDs[[2]] <- other_normal_IDs
  
  plottitles[[1]] <- paste0("Autoimmune thyroid\n(>",lymphocyte_fraction * 100,"% lymphocyte fraction)")
  plottitles[[2]] <- plot_titles[x]
  
  input_muts <- mutations[,!(colnames(mutations) == "paper_id")]
  input_muts <- rbind(input_muts,other_normal_muts[,colnames(input_muts)])
  
  combined_targeted_only_dnds_sel_cv[[1]] <- all_dnds_shm$sel_merged
  combined_targeted_only_dnds_sel_cv[[2]] <- other_normal_dnds_shm$sel_merged
  
  input_cov <- coverage[coverage$gene %in% immune_genes$gene[which(immune_genes$target_type != "Hotspot")],]
  input_cov_2 <- other_normal_cov[other_normal_cov$gene %in% immune_genes$gene[which(immune_genes$target_type != "Hotspot")],]
  rownames(input_cov_2) <- input_cov_2$gene
  input_cov <- cbind(input_cov,input_cov_2[input_cov$gene,!(colnames(input_cov_2) == "gene")])
  
  for(j in 1:2){
    temp_muts[[j]] <- input_muts[which(input_muts$sampleID %in% driverdensity_sampleIDs[[j]]),]
    unique_temp_muts[[j]] <- temp_muts[[j]][which(!(duplicated(temp_muts[[j]]$mut_site))),]
    temp_cov[[j]] <- rowSums(input_cov[which(colnames(input_cov) %in% driverdensity_sampleIDs[[j]])])
    names(temp_cov[[j]]) <- input_cov$gene
    temp_cov[[j]] <- temp_cov[[j]][which(temp_cov[[j]] != 0)]
    temp_cov[[j]] <- temp_cov[[j]][which(!(names(temp_cov[[j]]) %in% c("IGLL1","IGLL5")))]
    
    # NB - Need to run dNdS again as may restrict to different donor list (i.e. individuals aged ≥50)
    # NB - Discuss with Iñigo - pairwise dNdS uses regular dNdS rather than dNdSshm (as genes with nothing but SHM excluded from gene_muts such as LTB)
    dnds_temp <- dndscv(mutations = unique_temp_muts[[j]][,c(1:5)], gene_list = names(temp_cov[[j]]), max_muts_per_gene_per_sample = Inf, max_coding_muts_per_sample = Inf, onesided = T, dc = temp_cov[[j]], refdb = RefCDS_GRCh37_NSX, maxcovs = 10)
    
    kc <- as.vector(dnds_temp$sel_cv$gene_name[which(dnds_temp$sel_cv$qallsubs_cv<0.01)])
    
    dnds_out[[j]] <- dndscv(mutations = unique_temp_muts[[j]][,c(1:5)], gene_list = names(temp_cov[[j]]), max_muts_per_gene_per_sample = Inf, max_coding_muts_per_sample = Inf, onesided = T, dc = temp_cov[[j]], refdb = RefCDS_GRCh37_NSX, kc = kc, maxcovs = 10)
  }
  
  pairwise_dnds[[1]] <- pairwise_dNdScv(dnds_out[[1]], dnds_out[[2]], genestotest = genes2compare)
  pairwise_dnds[[2]] <- pairwise_dNdScv(dnds_out[[2]], dnds_out[[1]], genestotest = genes2compare)
  
  pairwise_dnds[[1]] <- pairwise_dnds[[1]][pairwise_dnds[[1]]$gene_name %in% genes2plot,]
  pairwise_dnds[[2]] <- pairwise_dnds[[2]][pairwise_dnds[[2]]$gene_name %in% genes2plot,]
  
  cell_frac_comp = compplot(
    comp_list = list(c("aitd",comp_list[x])),
    genes2plot = list(genes2plot),
    highlight_colour = list(highlight_colour),
    driverdensity_sampleIDs = list(driverdensity_sampleIDs),
    full_sampleIDs = list(full_sampleIDs),
    muts = input_muts, 
    combined_targeted_only_dnds_sel_cv = list(combined_targeted_only_dnds_sel_cv),
    targeted_dnds_sel_cv = NULL,
    gene2dc_array = input_cov,
    max_duplex_cov = 160000,
    min_mcf_upper = 4.5,
    pairwise_dnds = list(pairwise_dnds),
    runman = runman,
    plottitles = list(plottitles),
    plotfilename = paste0("output/EDF4_NonSynonymousMutantFractionComparison_HashimotoLymphocyteRich_other_normal_",comp_list[x],".pdf"),
    plotwidth = 8,
    plotheight = 8)
}  
       
  plot_categories <- c("AITD_LymphocyteAbove25Pct","PBMC_Bmem_AgedOver50","Spleen","LymphNode","Tonsillitis","LCM_Lymphoid_Aggregates","ThyroidGoitre")

genes2plot <- c("TNFRSF14","CD274")

lymphocyte_fraction <- 0.25
sorted_blood_age_cutoff <- 50

plot_sample_lists <- list()

plot_sample_lists[[1]] <- metadata$sample[metadata$sample_type == "Bulk" & substr(metadata$sample,1,8) %in% substr(emseq$sampleID[(emseq$Blood_B + emseq$Blood_T) > lymphocyte_fraction & emseq$sampleID %in% substr(metadata$sample[metadata$sample_type == "Bulk"],1,16)],1,8)]
plot_sample_lists[[2]] <- normal_metadata$sample[normal_metadata$age >= sorted_blood_age_cutoff & grepl("B_mem",normal_metadata$cell_type)]
plot_sample_lists[[3]] <- spleen_metadata$sample
plot_sample_lists[[4]] <- lymph_node_metadata$sample
plot_sample_lists[[5]] <- tonsillitis_metadata$sample
plot_sample_lists[[6]] <- lcm_lymphocyte_metadata$sample
plot_sample_lists[[7]] <- thyroid_goitre_metadata$sample

plot_donor_lists <- list()
for(j in 1:length(plot_categories)){
  plot_donor_lists[[j]] <- unique(substr(plot_sample_lists[[j]],1,7))
}

plot_mut_vec <- c("mutations","normal_muts","spleen_muts","lymph_node_muts","tonsillitis_muts","lcm_lymphocyte_muts","thyroid_goitre_muts")
plot_cov_vec <- c("coverage","normal_cov","spleen_cov","lymph_node_cov","tonsillitis_cov","lcm_lymphocyte_cov","thyroid_goitre_cov")

per_donor_cell_fraction <- array(data = NA, dim = c(length(unlist(plot_donor_lists)),7,length(genes2plot)))
dimnames(per_donor_cell_fraction) <- list(paste(rep(c(1:length(plot_categories)),times = lengths(plot_donor_lists)),unlist(plot_donor_lists),sep = "_"),c("category_no","category","donor","gene","duplexcov","cellfraction_low","cellfraction_high"),genes2plot)

for(x in 1:length(plot_categories)){
  for(y in 1:length(genes2plot)){
    per_donor_cell_fraction[which(grepl(paste0(x,"_"),unlist(dimnames(per_donor_cell_fraction)[1]))),"category_no",y] <- x
    per_donor_cell_fraction[which(grepl(paste0(x,"_"),unlist(dimnames(per_donor_cell_fraction)[1]))),"category",y] <- plot_categories[x]
    per_donor_cell_fraction[which(grepl(paste0(x,"_"),unlist(dimnames(per_donor_cell_fraction)[1]))),"donor",y] <- plot_donor_lists[[x]]
    per_donor_cell_fraction[which(grepl(paste0(x,"_"),unlist(dimnames(per_donor_cell_fraction)[1]))),"gene",y] <- genes2plot[y]
    for(z in 1:length(plot_donor_lists[[x]])){
      donor_sample_list <- plot_sample_lists[[x]][which(substr(plot_sample_lists[[x]],1,7) == plot_donor_lists[[x]][z])]
      muts2plot <- eval(parse(text = plot_mut_vec[x]))
      muts2plot <- muts2plot[which(muts2plot$sampleID %in% donor_sample_list & muts2plot$gene == genes2plot[y] & muts2plot$impact != "Synonymous"),,drop = F]
      per_sample_gene_cov <- eval(parse(text = plot_cov_vec[x]))
      per_sample_gene_cov <- per_sample_gene_cov[which(per_sample_gene_cov$gene == genes2plot[y]),colnames(per_sample_gene_cov) %in% donor_sample_list,drop = F]
      
      per_donor_cell_fraction[which(grepl(paste0(x,"_"),unlist(dimnames(per_donor_cell_fraction)[1])))[z],"duplexcov",y] <- sum(per_sample_gene_cov)
      if(nrow(muts2plot) > 0){
      aux <- muts2plot[,c("sampleID","cellfraction","duplex_vaf"),drop = F]
      aux$sample_gene_cov <- NA
      aux$cov_weighting <- NA
      for(k in 1:length(per_sample_gene_cov)){
        aux$sample_gene_cov[which(aux$sampleID == names(per_sample_gene_cov)[k])] <- as.numeric(per_sample_gene_cov[k])
      }
      aux$cov_weighting <- aux$sample_gene_cov / sum(per_sample_gene_cov)
      
      aux$weighted_cellfraction <- aux$cellfraction * aux$cov_weighting
      aux$weighted_duplex_vaf <- aux$duplex_vaf * aux$cov_weighting
      
      per_donor_cell_fraction[which(grepl(paste0(x,"_"),unlist(dimnames(per_donor_cell_fraction)[1])))[z],"cellfraction_low",y] <- sum(aux$weighted_duplex_vaf)
      per_donor_cell_fraction[which(grepl(paste0(x,"_"),unlist(dimnames(per_donor_cell_fraction)[1])))[z],"cellfraction_high",y] <- sum(aux$weighted_cellfraction)
      } else{
        per_donor_cell_fraction[which(grepl(paste0(x,"_"),unlist(dimnames(per_donor_cell_fraction)[1])))[z],"cellfraction_low",y] <- 0
      per_donor_cell_fraction[which(grepl(paste0(x,"_"),unlist(dimnames(per_donor_cell_fraction)[1])))[z],"cellfraction_high",y] <- 0
      }
    }
  }
}

per_donor_cell_fraction_df <- data.frame(array(data = NA, dim = c(dim(per_donor_cell_fraction)[1]*dim(per_donor_cell_fraction)[3],dim(per_donor_cell_fraction)[2])))
for(j in 1:length(genes2plot)){
    if(j == 1){
      colnames(per_donor_cell_fraction_df) <- colnames(per_donor_cell_fraction[,,1])
      per_donor_cell_fraction_df[1:nrow(per_donor_cell_fraction[,,1]),] <- per_donor_cell_fraction[,,j]
    }else{
      per_donor_cell_fraction_df[(((j - 1) * nrow(per_donor_cell_fraction[,,1])) + 1):(j * nrow(per_donor_cell_fraction[,,1])),] <- per_donor_cell_fraction[,,j]
    }
}

per_donor_cell_fraction_df$category_no <- as.numeric(per_donor_cell_fraction_df$category_no)
per_donor_cell_fraction_df$duplexcov <- as.numeric(per_donor_cell_fraction_df$duplexcov)
per_donor_cell_fraction_df$cellfraction_low <- as.numeric(per_donor_cell_fraction_df$cellfraction_low)
per_donor_cell_fraction_df$cellfraction_high <- as.numeric(per_donor_cell_fraction_df$cellfraction_high)

per_category_cell_fraction_df <- data.frame(array(data = NA, dim = c(length(plot_categories)*length(genes2plot),5)))
colnames(per_category_cell_fraction_df) <- c("category_no","category","gene","cellfraction_low","cellfraction_high")
for(j in 1:length(genes2plot)){
  for(k in 1:length(plot_categories)){
    per_category_cell_fraction_df$category_no[(j-1)*length(plot_categories) + k] <- k
    per_category_cell_fraction_df$category[(j-1)*length(plot_categories) + k] <- plot_categories[k]
    per_category_cell_fraction_df$gene[(j-1)*length(plot_categories) + k] <- genes2plot[j]
    aux <- per_donor_cell_fraction_df[which(per_donor_cell_fraction_df$category_no == k & per_donor_cell_fraction_df$gene == genes2plot[j]),]
    aux$cov_weighting <- aux$duplexcov / sum(aux$duplexcov)
    aux$weighted_cellfraction_low <- aux$cellfraction_low * aux$cov_weighting
    aux$weighted_cellfraction_high <- aux$cellfraction_high * aux$cov_weighting
    per_category_cell_fraction_df$cellfraction_low[(j-1)*length(plot_categories) + k] <- sum(aux$weighted_cellfraction_low)
    per_category_cell_fraction_df$cellfraction_high[(j-1)*length(plot_categories) + k] <- sum(aux$weighted_cellfraction_high)
  }
}

per_donor_cell_fraction_df$midpoint <- 100 * ((per_donor_cell_fraction_df$cellfraction_high + per_donor_cell_fraction_df$cellfraction_low) / 2)

aux <- array(data = NA, dim = c(length(genes2plot),length(plot_categories),2))
dimnames(aux) <- list(genes2plot,1:length(plot_categories),c("cellfraction_low","cellfraction_high"))
for(j in 1:length(genes2plot)){
  for(k in 1:length(plot_categories)){
    aux[j,k,1] <- 100 * per_category_cell_fraction_df[which(per_category_cell_fraction_df$category_no == k & per_category_cell_fraction_df$gene == genes2plot[j]),"cellfraction_low"]
    aux[j,k,2] <- 100 * per_category_cell_fraction_df[which(per_category_cell_fraction_df$category_no == k & per_category_cell_fraction_df$gene == genes2plot[j]),"cellfraction_high"]
    if(aux[j,k,1] <= 0.1){
      aux[j,k,1] <- 0.1
    }
    if(aux[j,k,2] <= 0.1){
      aux[j,k,2] <- 0.105
    }
  }
}

per_donor_cell_fraction_df$midpoint[per_donor_cell_fraction_df$midpoint < 0.1] <- 0.1

if (runman) { dev.new(width=10, height=10) }
par(mar = c(15.1,4.1,4.1,2.1))
pos = barplot(aux[,,2], names = plot_categories, beside = T,las = 2, col = c("firebrick","steelblue"), border = NA, ylim = c(0.08,52), ylab = "Mutant cell fraction", log = "y")
barplot(aux[,,1], beside = T,las = 2, col = c(rep("white", times = 2)), names = plot_categories, ylim = c(0.08,52), add = T, border = "white", log = "y")
for(j in 1:length(plot_categories)){
  for(k in 1:length(genes2plot)){
      points(x = jitter(pos[k,per_donor_cell_fraction_df[which(per_donor_cell_fraction_df$category_no == j & per_donor_cell_fraction_df$gene == genes2plot[k]),"category_no"]], amount = 0.2),y = per_donor_cell_fraction_df[which(per_donor_cell_fraction_df$category_no == j & per_donor_cell_fraction_df$gene == genes2plot[k]),"midpoint"], pch=21,bg=c("firebrick","steelblue")[k],col = "black",cex = 1)
  }
}
text(x = pos[1,ncol(pos)], y = rep(50,times = length(genes2plot)) * c(0.8^seq(from = 1,to = length(genes2plot), by = 1)), labels = genes2plot, col=c("firebrick","steelblue"))  
    
  if (runman) { dev.copy(pdf,"./output/Fig3C_MutantCellFractionControlCohorts.pdf",width=10,height=10); dev.off() }  
  aitd_mut_b_cell_fraction <- per_donor_cell_fraction_df[which(per_donor_cell_fraction_df$category == "AITD_LymphocyteAbove25Pct"),]

aitd_mut_b_cell_fraction$paper_id <- NA
aitd_mut_b_cell_fraction$estimated_B_cell_prop <- NA

for(j in 1:nrow(aitd_mut_b_cell_fraction)){
  aitd_mut_b_cell_fraction$paper_id[j] <- unique(substr(metadata$paper_id[which(metadata$donor == aitd_mut_b_cell_fraction$donor[j])],1,2))
  aitd_mut_b_cell_fraction$estimated_B_cell_prop[j] <- mean(emseq_bulk$Blood_B[which(emseq_bulk$paper_id == aitd_mut_b_cell_fraction$paper_id[j])])
}
aitd_mut_b_cell_fraction$mut_B_cell_fraction_low <- 100 * (aitd_mut_b_cell_fraction$cellfraction_low / aitd_mut_b_cell_fraction$estimated_B_cell_prop)
aitd_mut_b_cell_fraction$mut_B_cell_fraction_mid <- 100 * ((aitd_mut_b_cell_fraction$midpoint / 100) / aitd_mut_b_cell_fraction$estimated_B_cell_prop)
aitd_mut_b_cell_fraction$mut_B_cell_fraction_high <- 100 * (aitd_mut_b_cell_fraction$cellfraction_high / aitd_mut_b_cell_fraction$estimated_B_cell_prop)  
  # Lower bound of mutant cell fraction shown here given high frequency of homozygous TNFRSF14 mutations in PTA data
tnfrsf14_mut_b_cell <- aitd_mut_b_cell_fraction[which(aitd_mut_b_cell_fraction$gene == "TNFRSF14"),c("paper_id","mut_B_cell_fraction_low")]
kable(tnfrsf14_mut_b_cell, digits = 1, col.names = c("Donor","Mutant B cell fraction (lower bound)"))  
 
 
 
 Donor 
 Mutant B cell fraction (lower bound) 
 
 
 
 
 H1 
 100.2 
 
 
 H2 
 17.7 
 
 
 H3 
 13.6 
 
 
 G1 
 3.5 
 
 
 G3 
 0.4 
 
 
 G5 
 10.3 
 
 
 H7 
 1.5 
 
 
 H8 
 4.0 
 
 
 H9 
 2.7 
 
 
 
  # Lower bound of mutant cell fraction shown here given high frequency of homozygous TNFRSF14 mutations in PTA data
cd274_mut_b_cell <- aitd_mut_b_cell_fraction[which(aitd_mut_b_cell_fraction$gene == "CD274"),c("paper_id","mut_B_cell_fraction_low")]
kable(cd274_mut_b_cell, digits = 1, col.names = c("Donor","Mutant B cell fraction (lower bound)"), row.names = F)  
 
 
 
 Donor 
 Mutant B cell fraction (lower bound) 
 
 
 
 
 H1 
 12.0 
 
 
 H2 
 24.3 
 
 
 H3 
 0.4 
 
 
 G1 
 9.1 
 
 
 G3 
 0.8 
 
 
 G5 
 0.5 
 
 
 H7 
 0.0 
 
 
 H8 
 13.3 
 
 
 H9 
 2.3 
 
 
 
 
 
 
 16a. Spatial mapping 
 Four donors were studied further with laser capture microdissection
and whole exome sequencing. We then piled up variants called in matched
bulk samples with NanoSeq, in the microdissected whole exomes. This
enables the spatial mapping of mutations. 
 Run function across all mutations to map these to whole exomes that
have been piled up gene-wide from microdissections. This function
creates a folder for each donor in the directory:
./outputs/spatial_mapping/ 
 Genes with pileup data: TNFRSF14, CD274, CCR6, TET2, CBL (PD66718
only), DNMT3A (PD66718 only) 
 
 G5 (PD66711a) TNFRSF14 (selected) 
  # Spatial plot for selected mutations
muts_of_interest <-  mutations[mutations$mut_site %in% c("PD66711_1_2494337_T_A", "PD66711_1_2489254_C_CTGCCCCA", "PD66711_1_2488174_T_G"),]
muts_of_interest <- muts_of_interest[!duplicated(muts_of_interest$mut_site),]

spatial_mapper(LCM_record_file = LCM_record_file, 
               muts_of_interest = muts_of_interest,
               gene = "TNFRSF14",
               pileups_dir = pileups_dir,
               donor = "PD66711",
               outline_file = PD66711a_outline_file,
               cuts_file = PD66711a_cuts_file,
               show_plot = T)  
     
 
 
 H3a (PD63126b) TNFRSF14 (selected) 
  # Spatial plot for selected mutations
muts_of_interest <-  mutations[mutations$mut_site %in% c("PD63126_1_2488106_G_A", "PD63126_1_2488152_A_T", "PD63126_1_2488105_T_C", "PD63126_1_2489808_G_T"),]
muts_of_interest <- muts_of_interest[!duplicated(muts_of_interest$mut_site),]

spatial_mapper(LCM_record_file = LCM_record_file, 
               muts_of_interest = muts_of_interest,
               gene = "TNFRSF14",
               pileups_dir = pileups_dir,
               donor = "PD63126",
               outline_file = PD63126b_outline_file,
               cuts_file = PD63126b_cuts_file,
               show_plot = T)  
      
 
 
 H3a (PD63126b) TET2 (selected) 
  # Spatial plot for selected mutations
muts_of_interest <-  mutations[mutations$mut_site %in% c("PD63126_4_106197014_CA_C", "PD63126_4_106156881_CA_C"),]
muts_of_interest <- muts_of_interest[!duplicated(muts_of_interest$mut_site),]

spatial_mapper(LCM_record_file = LCM_record_file, 
               muts_of_interest = muts_of_interest,
               gene = "TET2",
               pileups_dir = pileups_dir,
               donor = "PD63126",
               outline_file = PD63126b_outline_file,
               cuts_file = PD63126b_cuts_file,
               show_plot = T)  
    
 
 
 H1 (PD63118b) TNFRSF14 (selected) 
  # Spatial plot for selected mutations
muts_of_interest <-  mutations[mutations$mut_site %in% c("PD63118_1_2488106_G_A", "PD63118_1_2489171_T_G", "PD63118_1_2489173_T_C", "PD63118_1_2488138_G_A", "PD63118_1_2488139_G_A", "PD63118_1_2489850_T_A", "PD63118_1_2491360_T_C"),]
muts_of_interest <- muts_of_interest[!duplicated(muts_of_interest$mut_site),]

spatial_mapper(LCM_record_file = LCM_record_file, 
               muts_of_interest = muts_of_interest,
               gene = "TNFRSF14",
               pileups_dir = pileups_dir,
               donor = "PD63118",
               outline_file = PD63118b_outline_file,
               cuts_file = PD63118b_cuts_file,
               show_plot = T)  
         
 
 
 H1 (PD63118b) TET2 (selected) 
  # Spatial plot for selected mutations
muts_of_interest <-  mutations[mutations$mut_site %in% c("PD63118_4_106156069_C_T"),]
muts_of_interest <- muts_of_interest[!duplicated(muts_of_interest$mut_site),]

spatial_mapper(LCM_record_file = LCM_record_file, 
               muts_of_interest = muts_of_interest,
               gene = "TET2",
               pileups_dir = pileups_dir,
               donor = "PD63118",
               outline_file = PD63118b_outline_file,
               cuts_file = PD63118b_cuts_file,
               show_plot = T)  
    
 
 
 H8 (PD66718b) DNMT3A (selected) 
  # Spatial plot for selected mutations
muts_of_interest <-  mutations[mutations$mut_site %in% c("PD66718_2_25457242_C_T"),]
muts_of_interest <- muts_of_interest[!duplicated(muts_of_interest$mut_site),]

spatial_mapper(LCM_record_file = LCM_record_file, 
               muts_of_interest = muts_of_interest,
               gene = "DNMT3A",
               pileups_dir = pileups_dir,
               donor = "PD66718",
               outline_file = PD66718b_outline_file,
               cuts_file = PD66718b_cuts_file,
               show_plot = T)  
    
 
 
 
 16b. Spatial mapping - Batch 2 (proximal to 5K Xenium data) 
 
 H1 (PD63118b) TNFRSF14 (selected) 
  # Spatial plot for selected mutations
muts_of_interest <-  mutations[mutations$mut_site %in% c("PD63118_1_2488104_A_T",
                                                         "PD63118_1_2488105_T_A",
                                                         "PD63118_1_2488138_G_A",
                                                         "PD63118_1_2489171_T_G",
                                                         "PD63118_1_2489173_T_C",
                                                         "PD63118_1_2489850_T_A",
                                                         "PD63118_1_2492129_TG_T",
                                                         "PD63118_1_2492131_G_T"),]
muts_of_interest <- muts_of_interest[!duplicated(muts_of_interest$mut_site),]

spatial_mapper(LCM_record_file = LCM_record_batch_2_file, 
               muts_of_interest = muts_of_interest,
               gene = "TNFRSF14",
               pileups_dir = pileups_batch_2_dir,
               donor = "PD63118",
               outline_file = PD63118b_batch_2_outline_file,
               cuts_file = PD63118b_batch_2_cuts_file,
               show_plot = T,
               restrict_to_lymphocytes = F)  
          ### H1
(PD63118b) CCR6 
  # Spatial plot for selected mutations
muts_of_interest <-  mutations[mutations$mut_site %in% c("PD63118_6_167550778_G_T"),]
muts_of_interest <- muts_of_interest[!duplicated(muts_of_interest$mut_site),]

spatial_mapper(LCM_record_file = LCM_record_batch_2_file, 
               muts_of_interest = muts_of_interest,
               gene = "CCR6",
               pileups_dir = pileups_batch_2_dir,
               donor = "PD63118",
               outline_file = PD63118b_batch_2_outline_file,
               cuts_file = PD63118b_batch_2_cuts_file,
               show_plot = T,
               restrict_to_lymphocytes = F)  
    
 
 
 H3 (PD63126b) TNFRSF14 (selected) 
  # Spatial plot for selected mutations
muts_of_interest <-  mutations[mutations$mut_site %in% c("PD63126_1_2488174_T_A",
                                                         "PD63126_1_2488174_T_A",
                                                         "PD63126_1_2488152_A_T",
                                                         "PD63126_1_2489220_G_C",
                                                         "PD63126_1_2493255_G_A"),]
muts_of_interest <- muts_of_interest[!duplicated(muts_of_interest$mut_site),]

spatial_mapper(LCM_record_file = LCM_record_batch_2_file, 
               muts_of_interest = muts_of_interest,
               gene = "TNFRSF14",
               pileups_dir = pileups_batch_2_dir,
               donor = "PD63126",
               outline_file = PD63126b_batch_2_outline_file,
               cuts_file = PD63126b_batch_2_cuts_file,
               show_plot = T,
               restrict_to_lymphocytes = F)  
     
 
 
 
 17. Single nucleus DNA sequencing from donor H1 
 For each nucleus, we generated a PTA sequencing library. We initially
carried out targeted sequencing using the same panel of 725 genes used
for targeted NanoSeq. For a subset of nuclei (all cells from the first
plate, solely B cells from the second plate), we also carried out whole
genome sequencing. The targeted PTA data is referred to as “DNAhyb”. 
 Mutations called by targeted NanoSeq were genotyped in the DNAHyb PTA
data. The filtered mutations table shows those genotyped mutations with
convincing support (i.e. VAF ≥ 0.25 & total depth ≥ 10). 
  pta_additional_annotation_H1 <- read.table(pta_additional_annotation_H1_file, sep = "\t", stringsAsFactors = F, header = T)
pta_dna_hyb_mutation_H1 <- read.table(pta_dna_hyb_mutation_H1_file, sep = "\t", stringsAsFactors = F, header = T)  
 A small number of mutations were also identified by manually
reviewing TNFRSF14 and CD274 loci in IGV. These rescued mutations are
merged with the genotyped targeted NanoSeq mutations. 
  pta_dna_hyb_rescued_muts_H1 <- read_xlsx(pta_dna_hyb_rescued_muts_H1_file)
pta_dna_hyb_mutation_H1 <- rbind(pta_dna_hyb_mutation_H1, pta_dna_hyb_rescued_muts_H1)  
 CD274 intron 1 splice site mutations are not annotated by dNdScv as
they lie outside the coding region but are still expected to be high
impact mutations. Manually change the annotation for a CD274 intron 1
splice site mutation to include it in the heatmap. 
  pta_dna_hyb_mutation_H1$gene[which(pta_dna_hyb_mutation_H1$chr == "9" & pta_dna_hyb_mutation_H1$pos == 5456097)] <- "CD274"
pta_dna_hyb_mutation_H1$impact[which(pta_dna_hyb_mutation_H1$chr == "9" & pta_dna_hyb_mutation_H1$pos == 5456097)] <- "Essential_Splice"  
 Cell 41 has a mutation affecting the polypyrimidine tract in intron 1
of CD274. This is predicted to disrupt splicing with high confidence by
SpliceAI (acceptor loss delta score = 0.92). Therefore, we have also
manually changed the annotation for this mutation to include it in the
heatmap. 
  pta_dna_hyb_mutation_H1$gene[which(pta_dna_hyb_mutation_H1$chr == "9" & pta_dna_hyb_mutation_H1$pos == 5456084)] <- "CD274"
pta_dna_hyb_mutation_H1$impact[which(pta_dna_hyb_mutation_H1$chr == "9" & pta_dna_hyb_mutation_H1$pos == 5456084)] <- "Essential_Splice"  
 Immune loci captured by targeted panel: TCRA: 14:22180451-23021069
TCRB: 7: 142000802-142511002 IGH: 14:106053233-106586330 IGLL5:
22:23230233-23237874 
 Mature B cells should have a significant proportion of genotyped
mutations occurring within heavy and light chain Ig loci. We include the
full V(D)J chromosomal regions for identifying genotyped Ig SHM
mutations in the code below, as the captured region can act as an anchor
for other portions of the Ig locus that are not directly captured. We
therefore consider the Ig loci to be: 
 Full immune loci IGH: 14:106053226-107288019 IGL:
22:22380474-23265203 
  pta_additional_annotation_H1$total_muts <- NA
pta_additional_annotation_H1$muts_in_immune_loci <- NA

for(x in 1:nrow(pta_additional_annotation_H1)){
  temp_muts <- pta_dna_hyb_mutation_H1[which(gsub("_dnahyb.*","",pta_dna_hyb_mutation_H1$id) == pta_additional_annotation_H1$well_ID[x]),]
  
  pta_additional_annotation_H1$total_muts[x] <- nrow(temp_muts)
  pta_additional_annotation_H1$muts_in_immune_loci[x] <- length(which(((temp_muts$chr == "14" & temp_muts$pos >= 106053226 & temp_muts$pos <= 107288019)|(temp_muts$chr == "22" & temp_muts$pos >= 22380474 & temp_muts$pos <= 23265203))))
}

pta_additional_annotation_H1$prop_immune_loci <- pta_additional_annotation_H1$muts_in_immune_loci / pta_additional_annotation_H1$total_muts
pta_additional_annotation_H1$prop_immune_loci[which(is.nan(pta_additional_annotation_H1$prop_immune_loci))] <- 0

pta_additional_annotation_H1$celltype_SHM <- NA
pta_additional_annotation_H1$celltype_SHM[pta_additional_annotation_H1$prop_immune_loci >= 0.1 & pta_additional_annotation_H1$muts_in_immune_loci >= 3] <- "Mature B cell"
pta_additional_annotation_H1$celltype_SHM[(pta_additional_annotation_H1$prop_immune_loci < 0.1 & pta_additional_annotation_H1$prop_immune_loci > 0) | (pta_additional_annotation_H1$muts_in_immune_loci < 3 & pta_additional_annotation_H1$muts_in_immune_loci > 0)] <- "Uncertain"
pta_additional_annotation_H1$celltype_SHM[pta_additional_annotation_H1$muts_in_immune_loci == 0] <- "Not mature B cell"

ggplot(pta_additional_annotation_H1[!(is.nan(pta_additional_annotation_H1$prop_immune_loci)),]) +
  geom_point((aes(x = muts_in_immune_loci, y = prop_immune_loci, color = celltype_VDJ_recomb)), alpha = 0.5) +
  geom_vline(xintercept = 3, colour = "red", lty = 2) +
  geom_hline(yintercept = 0.1, colour = "red", lty = 2) +
  theme_bw() +
  theme(panel.grid.major = element_blank(), panel.grid.minor = element_blank()) +
  scale_x_continuous(expand = c(0,1), limits = c(0,80)) +
  scale_y_continuous(expand = c(0,0.01), limits = c(0,1)) +
  labs(x = "Number of immune loci mutations", y = "Proportion of genotyped \n mutations in immune loci") +
  facet_wrap(~ celltype_VDJ_recomb)  
    
  ggplot(pta_additional_annotation_H1) +
  geom_point((aes(x = productive_heavy_chain_read_support, y = productive_TCRA_read_support, color = celltype_SHM)), alpha = 0.5) +
  theme_bw() +
  theme(panel.grid.major = element_blank(), panel.grid.minor = element_blank()) +
  labs(x = "Reads supporting productive \n Ig heavy chain rearrangement", y = "Reads supporting productive \n TCRA rearrangement", color = "SHM cell type") +
  facet_wrap(~ celltype_SHM)  
    
  ggplot(pta_additional_annotation_H1) +
  geom_point((aes(x = productive_heavy_chain_read_support, y = productive_TCRA_read_support, color = celltype_VDJ_recomb)), alpha = 0.5) +
  theme_bw() +
  theme(panel.grid.major = element_blank(), panel.grid.minor = element_blank()) +
  labs(x = "Reads supporting productive Ig heavy chain rearrangement", y = "Reads supporting productive \n TCRA rearrangement", color = "V(D)J cell type") +
  facet_wrap(~ celltype_VDJ_recomb)  
    
  ggplot(pta_additional_annotation_H1) +
  geom_point((aes(x = median_dna_hyb_cov, y = mean_csr_coverage_hyb, color = celltype_VDJ_recomb)), alpha = 0.5) +
  theme_bw() +
  theme(panel.grid = element_blank()) +
  scale_x_continuous(limits = c(0,500)) +
  labs(x = "Median DNA Hyb depth", y = "CSR depth", color = "V(D)J cell type") +
  geom_hline(yintercept = 1, lty = 2, color = "red") +
  facet_wrap(~celltype_VDJ_recomb)  
    
  pta_additional_annotation_H1$csr <- NA
pta_additional_annotation_H1$csr[which(pta_additional_annotation_H1$mean_csr_coverage_hyb >= 1)] <- 0
pta_additional_annotation_H1$csr[which(pta_additional_annotation_H1$mean_csr_coverage_hyb < 1)] <- 1  
  pta_dna_hyb_mutation_H1$cell_id <- NA 
for(x in 1:nrow(pta_additional_annotation_H1)){
  pta_dna_hyb_mutation_H1$cell_id[which(gsub("_dnahyb.*","",pta_dna_hyb_mutation_H1$id) == pta_additional_annotation_H1$well_ID[x])] <-  pta_additional_annotation_H1$cell_ID[x]
}

pta_dna_hyb_mutation_H1 <- pta_dna_hyb_mutation_H1[order(pta_dna_hyb_mutation_H1$cell_id, decreasing = F),]  
  pta_dna_hyb_mutation_H1$mut_site <- paste(pta_dna_hyb_mutation_H1$chr,pta_dna_hyb_mutation_H1$pos,pta_dna_hyb_mutation_H1$ref,pta_dna_hyb_mutation_H1$alt,sep = "_")
pta_dna_hyb_mutation_H1$merged_impact <- paste(pta_dna_hyb_mutation_H1$gene,pta_dna_hyb_mutation_H1$aachange,pta_dna_hyb_mutation_H1$impact,sep = "_")

pta_dna_hyb_mutation_H1$heatmap_annot <- NA

for(x in 1:nrow(pta_dna_hyb_mutation_H1)){
  if(!(is.na(pta_dna_hyb_mutation_H1$impact[x]))){
    if(pta_dna_hyb_mutation_H1$impact[x] %in% c("Missense","Nonsense","Synonymous")){
      pta_dna_hyb_mutation_H1$heatmap_annot[x] <- paste(pta_dna_hyb_mutation_H1$gene[x], pta_dna_hyb_mutation_H1$aachange[x], sep = "_")
    }else if(pta_dna_hyb_mutation_H1$impact[x] =="Essential_Splice"){
      pta_dna_hyb_mutation_H1$heatmap_annot[x] <- paste(pta_dna_hyb_mutation_H1$gene[x],"Splice",  sep = "_")
    }else if(pta_dna_hyb_mutation_H1$type[x] == "del"){
      pta_dna_hyb_mutation_H1$heatmap_annot[x] <- paste0(pta_dna_hyb_mutation_H1$gene[x],"_Del_",nchar(pta_dna_hyb_mutation_H1$ref[x]) - 1,"bp")
    }else if(pta_dna_hyb_mutation_H1$type[x] == "ins"){
      pta_dna_hyb_mutation_H1$heatmap_annot[x] <- paste0(pta_dna_hyb_mutation_H1$gene[x],"_Ins_",nchar(pta_dna_hyb_mutation_H1$alt[x]) - 1,"bp")
    }else{
      pta_dna_hyb_mutation_H1$heatmap_annot[x] <- paste0(pta_dna_hyb_mutation_H1$gene[x],"_",pta_dna_hyb_mutation_H1$type[x])
    }
  }
}

heatmap_annot_vec <- unique(pta_dna_hyb_mutation_H1$heatmap_annot)
for(x in 1:length(heatmap_annot_vec)){
  mut_site_vec <- unique(pta_dna_hyb_mutation_H1$mut_site[which(pta_dna_hyb_mutation_H1$heatmap_annot == heatmap_annot_vec[x])])
  if(length(mut_site_vec) > 1){
    for(y in 1:length(mut_site_vec)){
      pta_dna_hyb_mutation_H1$heatmap_annot[which(pta_dna_hyb_mutation_H1$mut_site == mut_site_vec[y])] <- paste0(heatmap_annot_vec[x],"_#",y)
    }
  }
}  
  empty_frame <- as.data.frame(array(data = NA, dim = c(length(unique(pta_additional_annotation_H1$cell_ID)),1)))
colnames(empty_frame) <- "cell_id"
empty_frame$cell_id <- unique(pta_additional_annotation_H1$cell_ID)

color.palette = colorRampPalette(c("white", "navy"))
wgs.palette = colorRampPalette(c("white","lightgoldenrod1"))
vdj.color.palette = colorRampPalette(c("lightcyan","lightcoral","palegreen1"))
shm.color.palette = colorRampPalette(c("white","peachpuff","lightyellow1"))
hap.color.palette = colorRampPalette(c("white","lightpink","lightblue"))
csr.color.palette = colorRampPalette(c("white","thistle"))
sv.color.palette = colorRampPalette(c("white","firebrick"))
vj.color.palette = colorRampPalette(c("white","springgreen4"))
cdr3.color.palette = colorRampPalette(c("moccasin","navajowhite4"))
breakpoint.color.palette = colorRampPalette(c("white","darkmagenta"))
antibody.color.palette = colorRampPalette(c("red","white"))

multicell_clades <- unique(pta_additional_annotation_H1$clade_ID[duplicated(pta_additional_annotation_H1$clade_ID)])
multicell_clade_lims <- vector(length = 2*length(multicell_clades))

for(x in 1:length(multicell_clades)){
  multicell_clade_lims[x] <- -1 * (max(pta_additional_annotation_H1$cell_ID[pta_additional_annotation_H1$clade_ID == multicell_clades[x]]) + 0.5)
  multicell_clade_lims[x + length(multicell_clades)] <- -1 * (min(pta_additional_annotation_H1$cell_ID[pta_additional_annotation_H1$clade_ID == multicell_clades[x]]) - 0.5)
}

multicell_clade_lims <- sort(unique(multicell_clade_lims))


subclades <- unique(pta_additional_annotation_H1$subclade_ID)
subclades <- subclades[which(subclades != "")]
subclade_lims <- vector(length = length(subclades))

for(x in 1:length(subclade_lims)){
  subclade_lims[x] <- -1 * (max(pta_additional_annotation_H1$cell_ID[pta_additional_annotation_H1$subclade_ID == subclades[x]]) + 0.5)
}

subclade_lims <- subclade_lims[which(!(subclade_lims %in% multicell_clade_lims))]  
  pta_cell_type <- reshape2::melt(pta_additional_annotation_H1[,c("cell_ID","wgs_sequencing","celltype_VDJ_recomb","celltype_SHM","csr")], id.vars = "cell_ID")
pta_cell_type$value[which(pta_cell_type$value %in% c("not lymphocyte","Uncertain"))] <- 0
pta_cell_type$value[which(pta_cell_type$value %in% c("B cell","Mature B cell"))]  <- 1
pta_cell_type$value[which(pta_cell_type$value %in% c("alpha-beta T cell","Not mature B cell"))]  <- 2

pta_cell_type$value <- as.numeric(pta_cell_type$value)

# Create WGS heatmap
wgs_sequenced = levelplot(value~variable*-cell_ID, pta_cell_type[which(pta_cell_type$variable == "wgs_sequencing"),], col.regions=wgs.palette, scales = list(tck = c(0,0), y = list(cex=1, at = seq(from = -1, to = -1 * nrow(empty_frame), by = -1), labels = c(1:nrow(empty_frame))), x = list(alternating = 3, rot = 90)), ylab = NULL, xlab="", colorkey=FALSE, 
                          panel=function(...) { arg <- list(...)
                          panel.levelplot(...)
                          panel.abline(h = multicell_clade_lims)
                          panel.abline(h = subclade_lims, lty = 2)})


# Create SHM heatmap
cell_type_SHM = levelplot(value~variable*-cell_ID, pta_cell_type[which(pta_cell_type$variable == "celltype_SHM"),], col.regions=shm.color.palette, scales = list(tck = c(0,0), y = list(cex=1, at = seq(from = -1, to = -1 * nrow(empty_frame), by = -1), labels = c(1:nrow(empty_frame))), x = list(alternating = 3, rot = 90)), ylab = NULL, xlab="", colorkey=FALSE, 
                          panel=function(...) { arg <- list(...)
                          panel.levelplot(...)
                          panel.abline(h = multicell_clade_lims)
                          panel.abline(h = subclade_lims, lty = 2)})

# Create VDJ heatmap
cell_type_VDJ = levelplot(value~variable*-cell_ID, pta_cell_type[which(pta_cell_type$variable == "celltype_VDJ_recomb"),], col.regions=vdj.color.palette, scales = list(tck = c(0,0), y = list(cex=1, at = seq(from = -1, to = -1 * nrow(empty_frame), by = -1), labels = c(1:nrow(empty_frame))), x = list(alternating = 3, rot = 90)), ylab = NULL, xlab="", colorkey=FALSE,
                          panel=function(...) { arg <- list(...)
                          panel.levelplot(...)
                          panel.abline(h = multicell_clade_lims)
                          panel.abline(h = subclade_lims, lty = 2)})

# Create CSR heatmap
cell_type_csr = levelplot(value~variable*-cell_ID, pta_cell_type[which(pta_cell_type$variable == "csr"),], col.regions=csr.color.palette, scales = list(tck = c(0,0), y = list(cex=1, at = seq(from = -1, to = -1 * nrow(empty_frame), by = -1), labels = c(1:nrow(empty_frame))), x = list(alternating = 3, rot = 90)), ylab = NULL, xlab="", colorkey=FALSE,
                          panel=function(...) { arg <- list(...)
                          panel.levelplot(...)
                          panel.abline(h = multicell_clade_lims)
                          panel.abline(h = subclade_lims, lty = 2)})  
  pta_1p_loh <- reshape2::melt(pta_additional_annotation_H1[,c("cell_ID","chr1p_haplotype","chr1p_breakpoint")], id.vars = "cell_ID")
pta_1p_loh$value[which(pta_1p_loh$value == "-")] <- 0
pta_1p_loh$value[which(pta_1p_loh$value == "A")] <- 1
pta_1p_loh$value[which(pta_1p_loh$value == "B")] <- 2

pta_1p_loh$value <- as.numeric(pta_1p_loh$value)

# Create haplotype heatmap
chr1p_loh_hap = levelplot(value~variable*-cell_ID, pta_1p_loh[which(pta_1p_loh$variable == "chr1p_haplotype"),], col.regions=hap.color.palette, scales = list(tck = c(0,0), y = list(cex=1, at = seq(from = -1, to = -1 * nrow(empty_frame), by = -1), labels = c(1:nrow(empty_frame))), x = list(alternating = 3, rot = 90)), ylab = NULL, xlab="", colorkey=FALSE, 
                          , panel=function(...) { arg <- list(...)
                          panel.levelplot(...)
                          panel.abline(h = multicell_clade_lims)
                          panel.abline(h = subclade_lims, lty = 2)})

# Create breakpoint heatmap
chr1p_loh_breakpoint = levelplot(value~variable*-cell_ID, pta_1p_loh[which(pta_1p_loh$variable == "chr1p_breakpoint"),], col.regions=breakpoint.color.palette(2431), scales = list(tck = c(0,0), y = list(cex=1, at = seq(from = -1, to = -1 * nrow(empty_frame), by = -1), labels = c(1:nrow(empty_frame))), x = list(alternating = 3, rot = 90)), ylab = NULL, xlab="", colorkey=list(space="top"), at = seq(from = 0, to = 121535434, by = 50000),  main = list(label = "chr 1p CN-LOH breakpoint"),
                                 panel=function(...) { arg <- list(...)
                                 panel.levelplot(...)
                                 panel.abline(h = multicell_clade_lims)
                                 panel.abline(h = subclade_lims, lty = 2)})  
  pta_TNFRSF14_muts <- pta_dna_hyb_mutation_H1[which(pta_dna_hyb_mutation_H1$gene == "TNFRSF14"),c("cell_id","heatmap_annot","alt_vaf")]
pta_TNFRSF14_muts$heatmap_annot <- gsub("TNFRSF14_","",pta_TNFRSF14_muts$heatmap_annot)
pta_TNFRSF14_mut_array <- reshape2::dcast(pta_TNFRSF14_muts, cell_id ~ heatmap_annot, value.var = "alt_vaf")
pta_TNFRSF14_mut_array <- pta_TNFRSF14_mut_array[,c("cell_id",unique(pta_TNFRSF14_muts$heatmap_annot))]

pta_TNFRSF14_mut_array <- left_join(empty_frame, pta_TNFRSF14_mut_array, by = "cell_id")
pta_TNFRSF14_muts <- reshape2::melt(pta_TNFRSF14_mut_array, id.var = "cell_id")
pta_TNFRSF14_muts$value[is.na(pta_TNFRSF14_muts$value)] <- 0 

TNFRSF14_vaf_heatmap <- lattice::levelplot(value~variable*-cell_id, data = pta_TNFRSF14_muts, col.regions=color.palette(1000), scales = list(tck = c(0,0), y = list(cex=1, at = seq(from = -1, to = -1 * nrow(empty_frame), by = -1), labels = c(1:nrow(empty_frame))), x = list(alternating = 3, rot = 90)), ylab="Cell ID", xlab = "TNFRSF14", colorkey=list(space="top"), at = seq(from = 0, to = 1, by = 0.001), main = list(label = "VAF"), panel=function(...) { arg <- list(...)
panel.levelplot(...)
panel.abline(h = multicell_clade_lims)
panel.abline(h = subclade_lims, lty = 2)}, aspect = ncol(pta_TNFRSF14_mut_array))  
  pta_CD274_muts <- pta_dna_hyb_mutation_H1[which(pta_dna_hyb_mutation_H1$gene == "CD274"),c("cell_id","heatmap_annot","alt_vaf")]
pta_CD274_muts$heatmap_annot <- gsub("CD274_","",pta_CD274_muts$heatmap_annot)
pta_CD274_mut_array <- reshape2::dcast(pta_CD274_muts, cell_id ~ heatmap_annot, value.var = "alt_vaf")
pta_CD274_mut_array <- pta_CD274_mut_array[,c("cell_id",unique(pta_CD274_muts$heatmap_annot))]

pta_CD274_mut_array <- left_join(empty_frame, pta_CD274_mut_array, by = "cell_id")
pta_CD274_muts <- reshape2::melt(pta_CD274_mut_array, id.var = "cell_id")
pta_CD274_muts$value[is.na(pta_CD274_muts$value)] <- 0 

CD274_vaf_heatmap <- lattice::levelplot(value~variable*-cell_id, data = pta_CD274_muts, col.regions=color.palette(1000), scales = list(tck = c(0,0), y = list(cex=1, at = seq(from = -1, to = -1 * nrow(empty_frame), by = -1), labels = c(1:nrow(empty_frame))), x = list(alternating = 3, rot = 90)), ylab="Cell ID", xlab = "CD274", colorkey=list(space="top"), at = seq(from = 0, to = 1, by = 0.001), main = list(label = "VAF"), panel=function(...) { arg <- list(...)
panel.levelplot(...)
panel.abline(h = multicell_clade_lims)
panel.abline(h = subclade_lims, lty = 2)})  
  pta_other_gene_muts <- pta_dna_hyb_mutation_H1[which(pta_dna_hyb_mutation_H1$gene %in% c("TET2","KLHL6","BRAF","CXCR3","RRAGC","RASA2","PIK3CD","PLCG2") & pta_dna_hyb_mutation_H1$impact != "Synonymous"),c("cell_id","heatmap_annot","alt_vaf")]
pta_other_gene_mut_array <- reshape2::dcast(pta_other_gene_muts, cell_id ~ heatmap_annot, value.var = "alt_vaf")
pta_other_gene_mut_array <- pta_other_gene_mut_array[,c("cell_id",unique(pta_other_gene_muts$heatmap_annot))]

pta_other_gene_mut_array <- pta_other_gene_mut_array[,c(1,
                                                        which(grepl("TET2",colnames(pta_other_gene_mut_array))),
                                                        which(grepl("BRAF",colnames(pta_other_gene_mut_array))),
                                                        which(grepl("PIK3CD",colnames(pta_other_gene_mut_array))),
                                                        which(grepl("PLCG2",colnames(pta_other_gene_mut_array))),
                                                        which(grepl("RRAGC",colnames(pta_other_gene_mut_array))),
                                                        which(grepl("KLHL6",colnames(pta_other_gene_mut_array))),
                                                        which(grepl("CXCR3",colnames(pta_other_gene_mut_array))),
                                                        which(grepl("RASA2",colnames(pta_other_gene_mut_array))))]

pta_other_gene_mut_array <- left_join(empty_frame, pta_other_gene_mut_array, by = "cell_id")
pta_other_gene_muts <- reshape2::melt(pta_other_gene_mut_array, id.var = "cell_id")
pta_other_gene_muts$value[is.na(pta_other_gene_muts$value)] <- 0 

other_gene_heatmap <- lattice::levelplot(value~variable*-cell_id, data = pta_other_gene_muts, col.regions=color.palette(1000), scales = list(tck = c(0,0), y = list(cex=1, at = seq(from = -1, to = -1 * nrow(empty_frame), by = -1), labels = c(1:nrow(empty_frame))), x = list(alternating = 3, rot = 90)), ylab="Cell ID", xlab = "Selected genes", colorkey=list(space="top", at = seq(from = 0, to = 1, by = 0.001), title = "VAF"), main = list(label = "VAF"), panel=function(...) { arg <- list(...)
panel.levelplot(...)
panel.abline(h = multicell_clade_lims)
panel.abline(h = subclade_lims, lty = 2)})  
  sv_categories <- unique(unlist(strsplit(pta_additional_annotation_H1$additional_structural_variants, split = "; ")))
sv_array <- as.data.frame(array(data = 0, dim = c(nrow(empty_frame),length(sv_categories))))

for(x in 1:length(sv_categories)){
  for(y in 1:nrow(empty_frame)){
    if(grepl(sv_categories[x],pta_additional_annotation_H1$additional_structural_variants[y])){
      sv_array[y,x] <- 1
    }
  }
}

sv_categories <- gsub(" ","_",sv_categories)
colnames(sv_array) <- sv_categories

sv_array$cell_id <- 1:nrow(sv_array)

sv_tidy <- reshape2::melt(sv_array, id.var = "cell_id")

other_sv_heatmap <- lattice::levelplot(value~variable*-cell_id, data = sv_tidy, col.regions=sv.color.palette, scales = list(tck = c(0,0), y = list(cex=1, at = seq(from = -1, to = -1 * nrow(empty_frame), by = -1), labels = c(1:nrow(empty_frame))), x = list(alternating = 3, rot = 90)), ylab="Cell ID", xlab = "Other SVs", colorkey=FALSE, panel=function(...) { arg <- list(...)
panel.levelplot(...)
panel.abline(h = multicell_clade_lims)
panel.abline(h = subclade_lims, lty = 2)})  
  pta_antibody_info <- reshape2::melt(pta_additional_annotation_H1[,c("cell_ID","human_antibody_TPO_EC50","human_antibody_TG_EC50")], id.vars = "cell_ID")

# Set the EC50 value for cells in which no antibody was synthesised to -1
pta_antibody_info$value[which(pta_antibody_info$cell_ID %in% pta_additional_annotation_H1$cell_ID[which(pta_additional_annotation_H1$human_antibody_id == "")])] <- "-1"

# Set the EC50 value for cells in which no binding was detected to 10
pta_antibody_info$value[which(is.na(pta_antibody_info$value))] <- 5

pta_antibody_info$value <- as.numeric(pta_antibody_info$value)

# Create human antibody synthesis heatmap

human_antibody_ec50_heatmap <- lattice::levelplot(value~variable*-cell_ID, data = pta_antibody_info, col.regions=c("#D3D3D3",antibody.color.palette(1000)), scales = list(tck = c(0,0), y = list(cex=1, at = seq(from = -1, to = -1 * nrow(empty_frame), by = -1), labels = c(1:nrow(empty_frame))), x = list(alternating = 3, rot = 90)), ylab="Cell ID", xlab = "TNFRSF14", colorkey=list(space="top"), at = c(-1,seq(from = 0, to = 5, by = 0.005)), main = list(label = "VAF"), panel=function(...) { arg <- list(...)
panel.levelplot(...)
panel.abline(h = multicell_clade_lims)
panel.abline(h = subclade_lims, lty = 2)}, aspect = ncol(pta_TNFRSF14_mut_array))  
  pta_additional_annotation_H1$heavy_V_consensus <- NA
pta_additional_annotation_H1$heavy_J_consensus <- NA
pta_additional_annotation_H1$TCRA_V_consensus <- NA
pta_additional_annotation_H1$TCRA_J_consensus <- NA

for(x in 1:nrow(pta_additional_annotation_H1)){
  heavy_V_temp <- gsub("\\*.*","",unlist(strsplit(pta_additional_annotation_H1$productive_heavy_chain_V[x], split = ",")))
  heavy_V_temp <- gsub("IGHV","",heavy_V_temp)
  if(length(unique(heavy_V_temp)) == 1){
    pta_additional_annotation_H1$heavy_V_consensus[x] <- unique(heavy_V_temp)
  }else if(length(heavy_V_temp) > 2){
    pta_additional_annotation_H1$heavy_V_consensus[x] <- names(table(heavy_V_temp)[which(table(heavy_V_temp) == max(table(heavy_V_temp)))])
  }else if(length(unique(heavy_V_temp)) == 2){
    sb <- stri_sub(unique(heavy_V_temp)[2], 1, 1:nchar(unique(heavy_V_temp)[2]))
    sstr <- na.omit(stri_extract_all_coll(unique(heavy_V_temp)[1], sb, simplify=TRUE))
    if(length(sstr[which.max(nchar(sstr))]) > 0){
      pta_additional_annotation_H1$heavy_V_consensus[x] <- sstr[which.max(nchar(sstr))]
    }else{
      pta_additional_annotation_H1$heavy_V_consensus[x] <- ""
    }
  }
  
  
  heavy_J_temp <- gsub("\\*.*","",unlist(strsplit(pta_additional_annotation_H1$productive_heavy_chain_J[x], split = ",")))
  heavy_J_temp <- gsub("IGHJ","",heavy_J_temp)
  if(length(unique(heavy_J_temp)) == 1){
    pta_additional_annotation_H1$heavy_J_consensus[x] <- unique(heavy_J_temp)
  }else if(length(heavy_J_temp) > 2){
    pta_additional_annotation_H1$heavy_J_consensus[x] <- names(table(heavy_J_temp)[which(table(heavy_J_temp) == max(table(heavy_J_temp)))])
  }else if(length(unique(heavy_J_temp)) == 2){
    sb <- stri_sub(unique(heavy_J_temp)[2], 1, 1:nchar(unique(heavy_J_temp)[2]))
    sstr <- na.omit(stri_extract_all_coll(unique(heavy_J_temp)[1], sb, simplify=TRUE))
    if(length(sstr[which.max(nchar(sstr))]) > 0){
      pta_additional_annotation_H1$heavy_J_consensus[x] <- sstr[which.max(nchar(sstr))]
    }else{
      pta_additional_annotation_H1$heavy_J_consensus[x] <- ""
    }
  }
  
  TCRA_V_temp <- gsub("\\*.*","",unlist(strsplit(pta_additional_annotation_H1$productive_TCRA_chain_V[x], split = ",")))
  TCRA_V_temp <- gsub("TRAV","",TCRA_V_temp)
  if(length(unique(TCRA_V_temp)) == 1){
    pta_additional_annotation_H1$TCRA_V_consensus[x] <- unique(TCRA_V_temp)
  }else if(length(TCRA_V_temp) > 2){
    pta_additional_annotation_H1$TCRA_V_consensus[x] <- names(table(TCRA_V_temp)[which(table(TCRA_V_temp) == max(table(TCRA_V_temp)))])
  }else if(length(unique(TCRA_V_temp)) == 2){
    sb <- stri_sub(unique(TCRA_V_temp)[2], 1, 1:nchar(unique(TCRA_V_temp)[2]))
    sstr <- na.omit(stri_extract_all_coll(unique(TCRA_V_temp)[1], sb, simplify=TRUE))
    if(length(sstr[which.max(nchar(sstr))]) > 0){
      pta_additional_annotation_H1$TCRA_V_consensus[x] <- sstr[which.max(nchar(sstr))]
    }else{
      pta_additional_annotation_H1$TCRA_V_consensus[x] <- ""
    }
  }
  
  TCRA_J_temp <- gsub("\\*.*","",unlist(strsplit(pta_additional_annotation_H1$productive_TCRA_chain_J[x], split = ",")))
  TCRA_J_temp <- gsub("TRAJ","",TCRA_J_temp)
  if(length(unique(TCRA_J_temp)) == 1){
    pta_additional_annotation_H1$TCRA_J_consensus[x] <- unique(TCRA_J_temp)
  }else if(length(TCRA_J_temp) > 2){
    pta_additional_annotation_H1$TCRA_J_consensus[x] <- names(table(TCRA_J_temp)[which(table(TCRA_J_temp) == max(table(TCRA_J_temp)))])
  }else if(length(unique(TCRA_J_temp)) == 2){
    sb <- stri_sub(unique(TCRA_J_temp)[2], 1, 1:nchar(unique(TCRA_J_temp)[2]))
    sstr <- na.omit(stri_extract_all_coll(unique(TCRA_J_temp)[1], sb, simplify=TRUE))
    if(length(sstr[which.max(nchar(sstr))]) > 0){
      pta_additional_annotation_H1$TCRA_J_consensus[x] <- sstr[which.max(nchar(sstr))]
    }else{
      pta_additional_annotation_H1$TCRA_J_consensus[x] <- ""
    }
  }
}

pta_additional_annotation_H1$TCRA_V_consensus <- gsub("/DV.*","",pta_additional_annotation_H1$TCRA_V_consensus)

pta_additional_annotation_H1$TCRA_V_consensus[which(pta_additional_annotation_H1$TCRA_V_consensus %in% names(table(pta_additional_annotation_H1$TCRA_V_consensus)[which(table(pta_additional_annotation_H1$TCRA_V_consensus) == 1)]))] <- "Unique"

pta_additional_annotation_H1$TCRA_J_consensus[which(pta_additional_annotation_H1$TCRA_J_consensus %in% names(table(pta_additional_annotation_H1$TCRA_J_consensus)[which(table(pta_additional_annotation_H1$TCRA_J_consensus) == 1)]))] <- "Unique"

pta_additional_annotation_H1$heavy_V_consensus[which(pta_additional_annotation_H1$heavy_V_consensus %in% names(table(pta_additional_annotation_H1$heavy_V_consensus)[which(table(pta_additional_annotation_H1$heavy_V_consensus) == 1)]))] <- "Unique"  
  igh_v_categories <- unique(pta_additional_annotation_H1$heavy_V_consensus)
igh_v_categories <- gsub("_","-",mixedsort(gsub("-","_",igh_v_categories)))
igh_v_categories <- igh_v_categories[which(igh_v_categories != "")]
igh_v_categories <- igh_v_categories[which(igh_v_categories != "-")]

igh_v_array <- as.data.frame(array(data = 0, dim = c(nrow(empty_frame),length(igh_v_categories))))

for(x in 1:length(igh_v_categories)){
  for(y in 1:nrow(empty_frame)){
    if(igh_v_categories[x] == pta_additional_annotation_H1$heavy_V_consensus[y]){
      igh_v_array[y,x] <- log2(pta_additional_annotation_H1$productive_heavy_chain_read_support[y])
    }
  }
}

colnames(igh_v_array) <- igh_v_categories

igh_v_array$cell_id <- 1:nrow(igh_v_array)

igh_v_tidy <- reshape2::melt(igh_v_array, id.var = "cell_id")

igh_v_heatmap <- lattice::levelplot(value~variable*-cell_id, data = igh_v_tidy, col.regions=vj.color.palette(1000), scales = list(tck = c(0,0), y = list(cex=1, at = seq(from = -1, to = -1 * nrow(empty_frame), by = -1), labels = c(1:nrow(empty_frame))), x = list(alternating = 3, rot = 90)), ylab="Cell ID", xlab = "", colorkey=list(space="top"), at = seq(from = 0, to = 10, by = 0.01), main = list(label = "log2 V(D)J read support"), panel=function(...) { arg <- list(...)
panel.levelplot(...)
panel.abline(h = multicell_clade_lims)
panel.abline(h = subclade_lims, lty = 2)})  
  igh_j_categories <- unique(pta_additional_annotation_H1$heavy_J_consensus)
igh_j_categories <- gsub("_","-",mixedsort(gsub("-","_",igh_j_categories)))
igh_j_categories <- igh_j_categories[which(igh_j_categories != "")]
igh_j_categories <- igh_j_categories[which(igh_j_categories != "-")]

igh_j_array <- as.data.frame(array(data = 0, dim = c(nrow(empty_frame),length(igh_j_categories))))

for(x in 1:length(igh_j_categories)){
  for(y in 1:nrow(empty_frame)){
    if(igh_j_categories[x] == pta_additional_annotation_H1$heavy_J_consensus[y]){
      igh_j_array[y,x] <- log2(pta_additional_annotation_H1$productive_heavy_chain_read_support[y])
    }
  }
}

colnames(igh_j_array) <- igh_j_categories

igh_j_array$cell_id <- 1:nrow(igh_j_array)

igh_j_tidy <- reshape2::melt(igh_j_array, id.var = "cell_id")

igh_j_heatmap <- lattice::levelplot(value~variable*-cell_id, data = igh_j_tidy, col.regions=vj.color.palette(1000), scales = list(tck = c(0,0), y = list(cex=1, at = seq(from = -1, to = -1 * nrow(empty_frame), by = -1), labels = c(1:nrow(empty_frame))), x = list(alternating = 3, rot = 90)), ylab="Cell ID", xlab = "", colorkey=list(space="top"), at = seq(from = 0, to = 10, by = 0.01), main = list(label = "log2 V(D)J read support"), panel=function(...) { arg <- list(...)
panel.levelplot(...)
panel.abline(h = multicell_clade_lims)
panel.abline(h = subclade_lims, lty = 2)})  
  pta_IgH_CDR3_input <- pta_additional_annotation_H1[,c("cell_ID","productive_heavy_chain_CDR3_nt_length")]
# pta_IgH_CDR3_input$productive_heavy_chain_CDR3_nt_length[which(pta_additional_annotation_H1$celltype_VDJ_recomb != "B cell")] <- NA
pta_IgH_CDR3_input$productive_heavy_chain_CDR3_nt_length[which(pta_IgH_CDR3_input$productive_heavy_chain_CDR3_nt_length == "-")] <- NA

pta_IgH_CDR3_input$productive_heavy_chain_CDR3_nt_length <- as.numeric(pta_IgH_CDR3_input$productive_heavy_chain_CDR3_nt_length)

pta_IgH_CDR3_input$productive_heavy_chain_CDR3_nt_length <- pta_IgH_CDR3_input$productive_heavy_chain_CDR3_nt_length / 3

colnames(pta_IgH_CDR3_input) <- c("cell_ID","Ig_heavy_CDR3_length")

pta_IgH_CDR3 <- reshape2::melt(pta_IgH_CDR3_input, id.vars = "cell_ID")
pta_IgH_CDR3$value[pta_IgH_CDR3$value == "-"] <- 0
pta_IgH_CDR3$value <- as.numeric(pta_IgH_CDR3$value)


# Create haplotype heatmap
pta_IgH_CDR3_heatmap = levelplot(value~variable*-cell_ID, pta_IgH_CDR3, col.regions=cdr3.color.palette(30), scales = list(tck = c(0,0), y = list(cex=1, at = seq(from = -1, to = -1 * nrow(empty_frame), by = -1), labels = c(1:nrow(empty_frame))), x = list(alternating = 3, rot = 90)), ylab = NULL, xlab="", colorkey=list(space="top"), at = seq(from = 0, to = 29, by = 1), main = list(label = "CDR3 aa length"), 
                                 , panel=function(...) { arg <- list(...)
                                 panel.levelplot(...)
                                 panel.abline(h = multicell_clade_lims)
                                 panel.abline(h = subclade_lims, lty = 2)})  
  tcra_v_categories <- unique(pta_additional_annotation_H1$TCRA_V_consensus)
tcra_v_categories <- gsub("_","-",mixedsort(gsub("-","_",tcra_v_categories)))
tcra_v_categories <- tcra_v_categories[which(tcra_v_categories != "")]
tcra_v_categories <- tcra_v_categories[which(tcra_v_categories != "-")]

tcra_v_array <- as.data.frame(array(data = 0, dim = c(nrow(empty_frame),length(tcra_v_categories))))

for(x in 1:length(tcra_v_categories)){
  for(y in 1:nrow(empty_frame)){
    if(tcra_v_categories[x] == pta_additional_annotation_H1$TCRA_V_consensus[y]){
      tcra_v_array[y,x] <- log2(pta_additional_annotation_H1$productive_TCRA_read_support[y])
    }
  }
}

colnames(tcra_v_array) <- tcra_v_categories

tcra_v_array$cell_id <- 1:nrow(tcra_v_array)

tcra_v_tidy <- reshape2::melt(tcra_v_array, id.var = "cell_id")

tcra_v_heatmap <- lattice::levelplot(value~variable*-cell_id, data = tcra_v_tidy, col.regions=vj.color.palette(1000), scales = list(tck = c(0,0), y = list(cex=1, at = seq(from = -1, to = -1 * nrow(empty_frame), by = -1), labels = c(1:nrow(empty_frame))), x = list(alternating = 3, rot = 90)), ylab="Cell ID", xlab = "", colorkey=list(space="top"), at = seq(from = 0, to = 10, by = 0.01), main = list(label = "log2 V(D)J read support"), panel=function(...) { arg <- list(...)
panel.levelplot(...)
panel.abline(h = multicell_clade_lims)
panel.abline(h = subclade_lims, lty = 2)})  
  tcra_j_categories <- unique(pta_additional_annotation_H1$TCRA_J_consensus)
tcra_j_categories <- gsub("_","-",mixedsort(gsub("-","_",tcra_j_categories)))
tcra_j_categories <- tcra_j_categories[which(tcra_j_categories != "")]
tcra_j_categories <- tcra_j_categories[which(tcra_j_categories != "-")]

tcra_j_array <- as.data.frame(array(data = 0, dim = c(nrow(empty_frame),length(tcra_j_categories))))

for(x in 1:length(tcra_j_categories)){
  for(y in 1:nrow(empty_frame)){
    if(tcra_j_categories[x] == pta_additional_annotation_H1$TCRA_J_consensus[y]){
      tcra_j_array[y,x] <- log2(pta_additional_annotation_H1$productive_TCRA_read_support[y])
    }
  }
}

colnames(tcra_j_array) <- tcra_j_categories

tcra_j_array$cell_id <- 1:nrow(tcra_j_array)

tcra_j_tidy <- reshape2::melt(tcra_j_array, id.var = "cell_id")

tcra_j_heatmap <- lattice::levelplot(value~variable*-cell_id, data = tcra_j_tidy, col.regions=vj.color.palette(1000), scales = list(tck = c(0,0), y = list(cex=1, at = seq(from = -1, to = -1 * nrow(empty_frame), by = -1), labels = c(1:nrow(empty_frame))), x = list(alternating = 3, rot = 90)), ylab="Cell ID", xlab = "", colorkey=list(space="top"), at = seq(from = 0, to = 10, by = 0.01), main = list(label = "log2 V(D)J read support"), panel=function(...) { arg <- list(...)
panel.levelplot(...)
panel.abline(h = multicell_clade_lims)
panel.abline(h = subclade_lims, lty = 2)})  
  pta_TCRA_CDR3_input <- pta_additional_annotation_H1[,c("cell_ID","TCRA_CDR3_nt_length")]
# pta_TCRA_CDR3_input$TCRA_CDR3_nt_length[which(pta_additional_annotation_H1$celltype_VDJ_recomb != "alpha-beta T cell")] <- NA
pta_TCRA_CDR3_input$TCRA_CDR3_nt_length[which(pta_TCRA_CDR3_input$TCRA_CDR3_nt_length == "-")] <- NA

pta_TCRA_CDR3_input$TCRA_CDR3_nt_length <- as.numeric(pta_TCRA_CDR3_input$TCRA_CDR3_nt_length)

pta_TCRA_CDR3_input$TCRA_CDR3_nt_length <- pta_TCRA_CDR3_input$TCRA_CDR3_nt_length / 3

colnames(pta_TCRA_CDR3_input) <- c("cell_ID","TCRA_CDR3_length")

pta_TCRA_CDR3 <- reshape2::melt(pta_TCRA_CDR3_input, id.vars = "cell_ID")
pta_TCRA_CDR3$value[pta_TCRA_CDR3$value == "-"] <- 0
pta_TCRA_CDR3$value <- as.numeric(pta_TCRA_CDR3$value)

# Create haplotype heatmap
pta_TCRA_CDR3_heatmap = levelplot(value~variable*-cell_ID, pta_TCRA_CDR3, col.regions=cdr3.color.palette(30), scales = list(tck = c(0,0), y = list(cex=1, at = seq(from = -1, to = -1 * nrow(empty_frame), by = -1), labels = c(1:nrow(empty_frame))), x = list(alternating = 3, rot = 90)), ylab = NULL, xlab="", colorkey=list(space="top"), at = seq(from = 0, to = 29, by = 1), main = list(label = "CDR3 aa length"), 
                                  , panel=function(...) { arg <- list(...)
                                  panel.levelplot(...)
                                  panel.abline(h = multicell_clade_lims)
                                  panel.abline(h = subclade_lims, lty = 2)})  
  comb_levObj_all <- c(wgs_sequenced,cell_type_VDJ,cell_type_SHM,cell_type_csr,chr1p_loh_hap,chr1p_loh_breakpoint,TNFRSF14_vaf_heatmap,CD274_vaf_heatmap,other_gene_heatmap,other_sv_heatmap, igh_v_heatmap, igh_j_heatmap,pta_IgH_CDR3_heatmap, tcra_v_heatmap, tcra_j_heatmap, pta_TCRA_CDR3_heatmap,human_antibody_ec50_heatmap, layout = c(17, 1),  merge.legends = FALSE)

if (runman) { dev.new(width=15, height=20) }
plot(comb_levObj_all, panel.width = list(c(1,1,1,1,1,1,ncol(pta_TNFRSF14_mut_array) - 1,ncol(pta_CD274_mut_array) - 1,ncol(pta_other_gene_mut_array) - 1,ncol(sv_array) - 1,ncol(igh_v_array) - 1,ncol(igh_j_array) - 1,1,ncol(tcra_v_array) - 1,ncol(tcra_j_array) - 1,1,2),c("null")))  
    
  if (runman) { dev.copy(pdf,"./output/PTA_Full_heatmap.pdf",width=16,height=20); dev.off() }  
  comb_levObj_main <- c(chr1p_loh_hap,chr1p_loh_breakpoint,TNFRSF14_vaf_heatmap,CD274_vaf_heatmap,other_gene_heatmap,other_sv_heatmap,human_antibody_ec50_heatmap, layout = c(7, 1),  merge.legends = FALSE)

if (runman) { dev.new(width=14, height=20) }
plot(comb_levObj_main, panel.width = list(c(1,1,ncol(pta_TNFRSF14_mut_array) - 1,ncol(pta_CD274_mut_array) - 1,ncol(pta_other_gene_mut_array) - 1,ncol(sv_array) - 1,2),c("null")))  
    
  if (runman) { dev.copy(pdf,"./output/PTA_MainText_heatmap.pdf",width=14,height=20); dev.off() }  
 
 
 18. Mutational signature analysis and phylogenetic tree from de novo
WGS calls for donor H1 PTA data 
  # set ggplot presets
theme_set(theme_classic())

celltype_cols <-
  c("non-naive B" = "#306b9b",
    "naive B" = "#449842",
    "alpha-beta T" = "#981c1e",
    "not B or T" = "#999999FF")

mut_lvls <-
  c("TNFRSF14 + CD274 mut", "TNFRSF14 mut", "CD274 mut",
    "TNFRSF14 WT + other mut")

cell_annots <- pta_additional_annotation_H1 %>%
  dplyr::mutate(
    celltype = dplyr::case_when(
      celltype_SHM == "Mature B cell" ~ "non-naive B",
      celltype_VDJ_recomb == "B cell" ~ "naive B",
      celltype_VDJ_recomb == "alpha-beta T cell" ~ "alpha-beta T",
      TRUE ~ "not B or T"
    ) %>% factor(levels = names(celltype_cols)),
    mut = dplyr::case_when(
      cell_ID %in% 42:47 ~ "TNFRSF14 WT + other mut",
      !biallelic_TNFRSF14 %in% c("WT", "FALSE_Het") &
        !biallelic_CD274 %in% c("WT", "FALSE_Het") ~ "TNFRSF14 + CD274 mut",
      !biallelic_TNFRSF14 %in% c("WT", "FALSE_Het") ~ "TNFRSF14 mut",
      !biallelic_CD274 %in% c("WT", "FALSE_Het") ~ "CD274 mut",
      TRUE ~ NA_character_
    ) %>% factor(levels = mut_lvls),
    celltype_col = celltype_cols[celltype]) %>%
  dplyr::arrange(celltype, mut) %>%
  tidyr::unite(celltype_mut, mut, celltype, sep = " ", na.rm = TRUE,
               remove = FALSE) %>%
  dplyr::mutate(celltype_mut = forcats::fct_inorder(celltype_mut)) %>%
  # create labels with newlines
  dplyr::mutate(
    celltype_mut_label = dplyr::case_when(
      celltype_mut == "TNFRSF14 + CD274 mut non-naive B" ~ "TNFRSF14 +\nCD274 mut\nnon-naive B",
      celltype_mut == "TNFRSF14 mut non-naive B" ~ "TNFRSF14 mut\nnon-naive B",
      celltype_mut == "TNFRSF14 WT + other mut non-naive B" ~ "TNFRSF14 WT\n+ other mut\nnon-naive B",
      TRUE ~ as.character(celltype_mut)),
    celltype_mut_label = forcats::fct_inorder(celltype_mut_label))  
 
 Plot phylogenetic tree with driver mutations in TNFRSF14 and CD274
annotated 
  # load tree
tree <- ape::read.tree(pta_H1_de_novo_WGS_calls_phylogeny_file)

# create tree df
tree_df <- as.data.frame(ggtree::fortify(tree))

# get tip colours
tip_cols <-
  cell_annots %>%
  dplyr::slice(match(as.numeric(tree$tip.label), cell_ID)) %>%
  dplyr::pull(celltype_col)

# get heatmap drivers
muts <- pta_dna_hyb_mutation_H1 %>%
  mutate(well_ID = gsub("_dna.*", "", id)) %>%
  inner_join(cell_annots %>% dplyr::select(well_ID, cell_ID), by = join_by(well_ID))

# function: plot drivers of interest on tree
plot_drivers_on_tree <- function(genes_of_interest) {

  # get heatmap drivers
  p_dat <-
    muts %>%
    filter(gene %in% genes_of_interest) %>%
    mutate(well_ID = gsub("_dna.*", "", id)) %>%
    inner_join(cell_annots %>% dplyr::select(well_ID, cell_ID)) %>%
    # reorder genes by number of mutations
    dplyr::add_count(gene) %>%
    dplyr::mutate(gene = forcats::fct_reorder(gene, -n)) %>%
    # for klhl6 hotspot, separate 13/14 mutation from the 10 mutation
    dplyr::mutate(
      chr = dplyr::case_when(gene == "KLHL6" & cell_ID == 10 ~ "sep",
                            TRUE ~ chr))

  # get mrca edge of each shared mutations
  tips_ls <-
    p_dat %>%
    split(.$gene) %>%
    purrr::map(function(gene_df) {
      gene_df %>%
        group_by(chr, pos, ref, alt) %>%
        group_split() %>%
        purrr::map(~ as.character(.x$cell_ID))
    }) %>% purrr::map(unique)

  # get gene colours
  gene_cols <-
    RColorBrewer::brewer.pal(n = length(tips_ls), name = "Set3") %>%
    setNames(names(tips_ls))
  gene_cols[c("TNFRSF14", "CD274")] <- c("orange", "purple")

  # plot tree
  par(family = "sans")
  plot(tree, cex = 0.65, font = 1, tip.color = tip_cols, label.offset = 0.003 * max(tree_df$x))
  coords <- get("last_plot.phylo", envir = .PlotPhyloEnv)

  # start list of edges and track plotting
  edges <- list()
  edge_counts <- integer(0)  # track number of genes already plotted on each edge
  edge_gene_plotted <- character(0)  # track which (edge, gene) pairs already plotted

  purrr::walk2(names(tips_ls), tips_ls, function(gene, gene_tips) {

    edges[[gene]] <<- list()

    purrr::walk(gene_tips, function(tips) {

      # get edge before tip or mrca node
      if (length(tips) == 1) {
        mrca_node <- which(tree$tip.label == tips)
        edge <- which(tree$edge[, 2] == mrca_node)
      } else {
        mrca_node <- getMRCA(tree, tips)
        edge <- which(tree$edge[, 2] == mrca_node)

        # make sure edge descends to tips only
        desc_tips <- extract.clade(tree, mrca_node)$tip.label
        if (!all(desc_tips %in% tips)) {
          # if not, then get edge before each tip
          edge <-
            tips %>%
            purrr::map(function(tip) {
              which(tree$edge[, 2] == which(tree$tip.label == tip))
            }) %>%
            unlist()
        }
      }

      # add edge(s) to list of edges
      edges[[gene]] <<- c(edges[[gene]], list(edge))

      for (e in edge) {
        # skip if this (edge, gene) combination was already plotted
        edge_gene_key <- paste(e, gene, sep = "_")
        if (edge_gene_key %in% edge_gene_plotted) next
        edge_gene_plotted <<- c(edge_gene_plotted, edge_gene_key)
        
        # edge coordinates (parent -> child)
        x0 <- coords$xx[tree$edge[e, 1]]
        y0 <- coords$yy[tree$edge[e, 1]]
        x1 <- coords$xx[tree$edge[e, 2]]
        y1 <- coords$yy[tree$edge[e, 2]]
        
        # get the current count for this edge and increment
        edge_key <- as.character(e)
        if (is.na(edge_counts[edge_key])) {
          edge_counts[edge_key] <<- 0
        }
        current_count <- edge_counts[edge_key]
        edge_counts[edge_key] <<- current_count + 1
        
        # shift each successive gene further (alternating left/right from center)
        # count 0 -> shift 0 (centered), count 1 -> shift right, count 2 -> shift left, etc.
        base_shift <- 0.6 * strwidth("M", cex = 1)
        if (current_count == 0) {
          x_shift <- 0
        } else {
          # alternate: 1 -> +1, 2 -> -1, 3 -> +2, 4 -> -2, etc.
          direction <- ifelse(current_count %% 2 == 1, 1, -1)
          magnitude <- ceiling(current_count / 2)
          x_shift <- base_shift * magnitude * direction
        }
        
        x_mid <- (x0 + x1) / 2 + x_shift
        y_mid <- y1

        # add points
        points(x_mid, y_mid, pch = 19, col = gene_cols[gene], cex = 1)
      }

    })

  })

  # add axis and legend
  axisPhylo(side = 1, backward = FALSE)
  legend("bottomright",
        legend = c("Driver mutation", names(gene_cols), "Cell type",
                    names(celltype_cols)),
        text.font = c(2, rep(3, length(gene_cols)), 2,
                      rep(1, length(celltype_cols))),
        col = c(NA, gene_cols, rep("black", length(celltype_cols) + 1)),
        pch = c(NA, rep(19, length(gene_cols)), NA,
                rep(95, length(celltype_cols))),
        pt.cex = 1,
        cex = 0.7,
        bty = "n",
        text.col = c(rep("black", length(gene_cols) + 2), celltype_cols))
}

plot_drivers_on_tree(genes_of_interest = c("TNFRSF14", "CD274"))  
    
 
 
 Plot phylogenetic tree with all driver mutations annotated in the
heatmap annotated 
  plot_drivers_on_tree(genes_of_interest = c("TNFRSF14", "CD274", "BRAF", "TET2", "PIK3CD", "PLCG2", "RRAGC", "KLHL6", "CXCR3", "RASA2"))  
    
 
 
 Load signature exposures 
  # define the final sigs
final_sigs <-
  c("SBS1", "SBS5", "SBS9", "SBS17a", "SBS17b", "SBS85",
    "machado_2022_SBSblood", "petljak_2019_ScF")

# define sig colours
sig_cols <-
  c("black", RColorBrewer::brewer.pal(n = length(final_sigs), "Set2")) %>%
  setNames(c("indels", final_sigs))

# load sigfit exposures with indels
sf_exp_w_indels <- readRDS(pta_H1_de_novo_sigfit_exposures_file)  
 
 
 Phylogenetic tree with signature exposures (tips coloured by
celltype) 
  # create tree
plot(tree, cex = 0.65, label.offset = 0.003 * max(tree_df$x),
     tip.color = celltype_cols, font = 1)

# for each sample, draw rectangles showing signature proportions
for (sample in colnames(sf_exp_w_indels)) {
  n <- as.numeric(substr(sample, 9, nchar(sample)))
  x_end <- tree_df$x[n]
  x_start <- tree_df$x[tree_df$parent[n]]
  x_intv <- x_end - x_start
  y <- node.height(tree)[n]
  tipnum <- sum(tree_df$isTip)

  # stack signature exposures proportionally along branch length
  for (s in rownames(sf_exp_w_indels)) {
    x_end <- x_start + sf_exp_w_indels[s, sample] * x_intv
    rect(ybottom = y - min(0.015 * tipnum, 0.3),
         ytop = y + min(0.015 * tipnum, 0.3),
         xleft = x_start, xright = x_end, col = sig_cols[s], lwd = 0.25)
    x_start <- x_end
  }
}

# axis and legend
axisPhylo(side = 1, backward = FALSE)
legend("topright",
       legend = c("Signature", names(sig_cols),
                  "Cell type", names(celltype_cols)),
       text.font = c(2, rep(1, length(sig_cols)), 2,
                     rep(1, length(celltype_cols))),
       col = c(NA, sig_cols, rep("black", length(celltype_cols) + 1)),
       pch = c(NA, rep(19, length(sig_cols)), NA,
                  rep(95, length(celltype_cols))),
       pt.cex = 1,
       cex = 0.7,
       bty = "n",
       text.col = c(rep("black", length(sig_cols) + 2), celltype_cols))  
    
 
 
 Phylogenetic tree with signature exposures (tips coloured by
celltype and mutation status) 
  # define celltype_mut tip colours
celltype_mut_cols <-
  RColorBrewer::brewer.pal(n = length(levels(cell_annots$celltype_mut)),
                           name = "Dark2") %>%
  setNames(levels(cell_annots$celltype_mut))
celltype_mut_cols[c("naive B", "alpha-beta T", "not B or T")] <- "grey"
tip_cols <-
  cell_annots %>%
  dplyr::slice(match(as.numeric(tree$tip.label), cell_ID)) %>%
  dplyr::pull(celltype_mut) %>%
  {celltype_mut_cols[.]}

# create tree
plot(tree, cex = 0.65, label.offset = 0.003 * max(tree_df$x),
     tip.color = tip_cols, font = 1)

# for each sample, draw rectangles showing signature proportions
for (sample in colnames(sf_exp_w_indels)) {
  n <- as.numeric(substr(sample, 9, nchar(sample)))
  x_end <- tree_df$x[n]
  x_start <- tree_df$x[tree_df$parent[n]]
  x_intv <- x_end - x_start
  y <- node.height(tree)[n]
  tipnum <- sum(tree_df$isTip)

  # stack signature exposures proportionally along branch length
  for (s in rownames(sf_exp_w_indels)) {
    x_end <- x_start + sf_exp_w_indels[s, sample] * x_intv
    rect(ybottom = y - min(0.015 * tipnum, 0.3),
         ytop = y + min(0.015 * tipnum, 0.3),
         xleft = x_start, xright = x_end, col = sig_cols[s], lwd = 0.25)
    x_start <- x_end
  }
}

# axis and legend
axisPhylo(side = 1, backward = FALSE)
legend("topright",
       legend = c("Signature", names(sig_cols),
                  "Cell type and mutant status", names(celltype_mut_cols)),
       text.font = c(2, rep(1, length(sig_cols)), 2,
                     rep(1, length(celltype_mut_cols))),
       col = c(NA, sig_cols, rep("black", length(celltype_mut_cols) + 1)),
       pch = c(NA, rep(19, length(sig_cols)), NA,
                  rep(95, length(celltype_mut_cols))),
       pt.cex = 1,
       cex = 0.55,
       bty = "n",
       text.col = c(rep("black", length(sig_cols) + 2), celltype_mut_cols))  
   
### Load trinucleotide matrix 
  # define mutation type colours
mut_type_cols <-
  c("C>A" = "dodgerblue", "C>G" = "black", "C>T" = "red",
    "T>A" = "grey70", "T>C" = "olivedrab3", "T>G" = "plum2")

# load trinuc mut matrix
trinuc_mut_mat <- read.table(pta_H1_de_novo_trinuc_mut_mat_hdp_file, check.names = FALSE)

# get all nodes leading to each tip for aggregating per cell
nodes_to_tips <-
  lapply(1:Ntip(tree), function(tip) {
    nodepath(tree, from = tip, to = Ntip(tree) + 1)
  }) %>%
  setNames(tree$tip.label) %>%
  tibble::enframe(name = "cell_ID", value = "node") %>%
  tidyr::unnest(cols = node)

# prepare plotting data
p_dat <-
  trinuc_mut_mat %>%
  tibble::as_tibble(rownames = "sample") %>%
  dplyr::mutate(node = as.numeric(gsub("Patient_", "", sample))) %>%
  dplyr::left_join(nodes_to_tips, by = join_by(node)) %>%
  dplyr::group_by(cell_ID) %>%
  dplyr::summarise(dplyr::across(dplyr::all_of(colnames(trinuc_mut_mat)), sum)) %>%
  dplyr::left_join(cell_annots %>% dplyr::mutate(cell_ID = as.character(cell_ID)), by = join_by(cell_ID))  
 
 
 Trinucleotide spectrum per celltype 
  p_dat %>%
  dplyr::group_by(celltype) %>%
  dplyr::summarise(dplyr::across(dplyr::all_of(colnames(trinuc_mut_mat)), sum)) %>%
  tidyr::pivot_longer(cols = -c("celltype")) %>%
  dplyr::mutate(trinuc = paste0(substr(name, 5, 5), substr(name, 1, 1), substr(name, 7, 7)),
                mut_type = substr(name, 1, 3)) %>%
  ggplot(aes(x = trinuc, y = value, fill = mut_type)) +
  geom_col() +
  scale_fill_manual(values = mut_type_cols) +
  facet_grid(celltype ~ mut_type, scales = "free", space = "free_x") +
  theme_classic() +
  theme(axis.text.x = element_text(family = "mono"),
        axis.title.x = element_blank(), axis.title.y = element_blank(),
        panel.border = element_rect(colour = "black", fill = NA, linewidth = 0.7),
        strip.background = element_rect(colour = "black", fill = NA, linewidth = 0.7),
        legend.position = "none", strip.text.y = element_text(angle = 90)) +
  guides(x = guide_axis(angle = -90)) +
  scale_y_continuous(expand = expansion(mult = c(0, 0.05)))  
    
 
 
 Trinucleotide spectrum per celltype and mutation status 
  p_dat %>%
  dplyr::group_by(celltype_mut_label) %>%
  dplyr::summarise(dplyr::across(dplyr::all_of(colnames(trinuc_mut_mat)), sum)) %>%
  tidyr::pivot_longer(cols = -c("celltype_mut_label")) %>%
  dplyr::mutate(trinuc = paste0(substr(name, 5, 5), substr(name, 1, 1), substr(name, 7, 7)),
                mut_type = substr(name, 1, 3)) %>%
  ggplot(aes(x = trinuc, y = value, fill = mut_type)) +
  geom_col() +
  scale_fill_manual(values = mut_type_cols) +
  facet_grid(celltype_mut_label ~ mut_type, scales = "free", space = "free_x") +
  theme_classic() +
  theme(axis.text.x = element_text(family = "mono"),
        axis.title.x = element_blank(), axis.title.y = element_blank(),
        panel.border = element_rect(colour = "black", fill = NA, linewidth = 0.7),
        strip.background = element_rect(colour = "black", fill = NA, linewidth = 0.7),
        legend.position = "none", strip.text.y = element_text(angle = 90)) +
  guides(x = guide_axis(angle = -90)) +
  scale_y_continuous(expand = expansion(mult = c(0, 0.05)))  
    
 
 
 Signature exposures per cell 
  # load exposures with filled-in branches
per_branch_exp_final <- readRDS(pta_H1_de_novo_per_branch_exposures_file)

# get exposures per cell
exp_per_cell <-
  nodes_to_tips %>%
  dplyr::left_join(per_branch_exp_final, by = c("node")) %>%
  # calculate mutations per exposure
  tidyr::pivot_longer(cols = dplyr::all_of(c("indels", final_sigs)),
                      names_to = "signature", values_to = "exposure") %>%
  dplyr::mutate(n_muts_exp = exposure * branch.length) %>%
  dplyr::group_by(cell_ID, signature) %>%
  dplyr::summarise(n_muts_exp = sum(n_muts_exp)) %>%
  # calculate mutations per cell
  dplyr::group_by(cell_ID) %>%
  dplyr::mutate(total_n_muts = sum(n_muts_exp)) %>%
  dplyr::ungroup() %>%
  # add cell annots
  dplyr::left_join(cell_annots %>% dplyr::mutate(cell_ID = as.character(cell_ID))) %>%
  # factor axes
  dplyr::mutate(cell_ID = forcats::fct_reorder(cell_ID, -total_n_muts),
                signature = factor(signature, levels = rev(c("indels", final_sigs)))) %>%
  # calculate proportions and numbers of mutations
  dplyr::mutate(`n mutations` = n_muts_exp,
                `% mutations` = n_muts_exp / total_n_muts) %>%
  tidyr::pivot_longer(cols = c("n mutations", "% mutations"),
                      names_to = "metric", values_to = "value") %>%
  dplyr::mutate(metric = factor(metric, levels = c("n mutations", "% mutations")))

# plot
exp_per_cell %>%
  ggplot(aes(x = cell_ID, y = value, fill = signature)) +
  geom_col(width = 1.05) +
  scale_fill_manual(values = sig_cols) +
  guides(x = guide_axis(angle = 90)) +
  theme_classic() +
  theme(axis.title.x = element_blank(), axis.title.y = element_blank(),
        panel.spacing.y = unit(0.6, "lines"),
        panel.border = element_rect(colour = "black", fill = NA, linewidth = 0.7),
        strip.background = element_rect(colour = "black", fill = NA, linewidth = 0.7),
        strip.text.y = element_text(angle = 90)) +
  scale_y_continuous(expand = expansion(mult = c(0, 0))) +
  scale_x_discrete(expand = c(0, 0)) +
  ggh4x::facet_nested(metric ~ celltype_mut_label, scales = "free", space = "free_x") +
  ggtitle("by celltype and TNFRSF14/CD274 status")  
    
 
 
 Per signature mutation burden across cell types and mutation
status 
  exp_per_cell <- readRDS(pta_H1_de_novo_exposures_per_cell_file)  %>%
  split(.$signature)

one_cell_per_clade <- vector()
wgs_clades <- unique(pta_additional_annotation_H1$clade_ID[which(pta_additional_annotation_H1$wgs_sequencing == 1)])

pta_annotation_reordered <- rbind(pta_additional_annotation_H1[which(pta_additional_annotation_H1$reconstruction_confidence == "high"),],pta_additional_annotation_H1[which(pta_additional_annotation_H1$reconstruction_confidence == "low"),],pta_additional_annotation_H1[which(pta_additional_annotation_H1$reconstruction_confidence == ""),])

for(j in 1:length(wgs_clades)){
  one_cell_per_clade <- c(one_cell_per_clade,pta_annotation_reordered$cell_ID[which(pta_annotation_reordered$clade_ID == wgs_clades[j])][1])
}

exp_per_clade <- exp_per_cell
for(j in 1:length(exp_per_clade)){
  exp_per_clade[[j]] <- exp_per_clade[[j]][which(exp_per_clade[[j]]$tip_id %in% one_cell_per_clade & exp_per_clade[[j]]$metric == "n mutations"),]
}

p_dat_per_clade <- p_dat[which(p_dat$cell_ID %in% one_cell_per_clade),]

purrr::map2(names(exp_per_clade), exp_per_clade, function(sig, p_dat_per_clade) {
  p <-
    p_dat_per_clade %>%
    ggplot(aes(x = celltype_mut, y = n_muts_exp)) +
    geom_boxplot(outliers = F) +
    geom_jitter(height = 0, width = 0.2)
  my_comparisons <-
    p_dat_per_clade %>%
    dplyr::filter(celltype_maturity == "mature B", celltype_mut != "mature B") %>%
    dplyr::pull(celltype_mut) %>%
    unique() %>%
    as.character() %>%
    purrr::map(~ c("mature B", .x))
  p +
    ggpubr::stat_compare_means(comparisons = my_comparisons,
                               method = "wilcox.test",
                               label = "p.format",
                               method.args = list(alternative = "two.sided")) +
    annotate("text", x = Inf, y = Inf, label = "Wilcoxon test", 
             hjust = 1.1, vjust = 1.5, size = 5, fontface = "italic") +
    ggtitle(sig) +
    theme_bw() +
    theme(axis.text.x = element_text(angle = 45, vjust = 1, hjust = 1), panel.grid = element_blank())
  if (runman) {ggsave(paste0("./output/wilcoxon_",sig,".pdf"), width = 8, height = 10)}
})  
  ## [[1]]
## NULL
## 
## [[2]]
## NULL
## 
## [[3]]
## NULL
## 
## [[4]]
## NULL
## 
## [[5]]
## NULL
## 
## [[6]]
## NULL
## 
## [[7]]
## NULL
## 
## [[8]]
## NULL
## 
## [[9]]
## NULL  
  shm_per_cell <- read.table(pta_H1_reconstructed_BCR_shm_counts_file, sep = "\t", stringsAsFactors = F, header = T)

shm_per_clade <- shm_per_cell[which(shm_per_cell$well_id %in% p_dat_per_clade$well_ID),]

cell_annots <-
  exp_per_clade[[1]] %>%
  dplyr::transmute(well_id = cell_id, celltype_mut)

my_comparisons = list(c("TNFRSF14 +\nCD274 mut\nmature B","mature B"),
                      c("TNFRSF14 mut\nmature B","mature B"),
                      c("TNFRSF14 WT\n+ other mut\nmature B","mature B"))

shm_per_clade %>%
  dplyr::left_join(cell_annots, by = join_by(well_id)) %>%
    dplyr::filter(celltype_mut != "non-mature\nB") %>%
  ggplot(aes(x = celltype_mut, y = shm_fraction)) +
  geom_boxplot(outliers = F) +
  geom_jitter(height = 0, width = 0.2) +
  ggpubr::stat_compare_means(comparisons = my_comparisons,
                             method = "wilcox.test",
                             label = "p.format",
                             method.args = list(alternative = "two.sided")) +
  annotate("text", x = Inf, y = Inf, label = "Wilcoxon test", 
           hjust = 1.1, vjust = 1.5, size = 3, fontface = "italic") +
  ggtitle("SHM fraction") +
  theme_bw() +
  labs(x = "Non-naive B cells",y = "Fraction of bases with SHM") +
  theme(axis.text.x = element_text(angle = 45, vjust = 1, hjust = 1), panel.grid = element_blank())  
    
  if (runman) {ggsave(paste0("./output/reconstructed_BCR_shm_fraction.pdf"), width = 5, height = 8)}  
 
 
 
 19. ELISA results for synthesised antibodies from donor H1 
  synthesised_antibody_ELISA_H1 <- read.table(synthesised_antibody_ELISA_file, sep = "\t", stringsAsFactors = F, header = T)

synthesised_antibody_ELISA_H1_melt <- reshape2::melt(data = synthesised_antibody_ELISA_H1, idvar = c("CellID","ExternalID"))
synthesised_antibody_ELISA_H1_melt$variable <- gsub(pattern = "PrimaryAntibody", replacement = "",x = synthesised_antibody_ELISA_H1_melt$variable)
synthesised_antibody_ELISA_H1_melt$variable <- gsub(pattern = "nM", replacement = "",x = synthesised_antibody_ELISA_H1_melt$variable)
synthesised_antibody_ELISA_H1_melt$variable <- as.numeric(synthesised_antibody_ELISA_H1_melt$variable)

# Set 0 nM concentration to a value that can be shown on log scale
synthesised_antibody_ELISA_H1_melt$variable[which(synthesised_antibody_ELISA_H1_melt$variable == 0)] <- 10^-3  
  antigen = c("TPO","TG")
antigen_full_name = c("Thyroid peroxidase","Thyroglobulin")
antigen_max_OD450 = c(1,2.5)
control_antibody_data <- synthesised_antibody_ELISA_H1_melt[which(grepl("control",synthesised_antibody_ELISA_H1_melt$CellID)),]

control_antibody_list <- unique(control_antibody_data$CellID)
control_antibody_list <- sort(control_antibody_list, decreasing = T)

control_color_list <- c("grey26","grey24","grey22","grey20","grey48","grey46","grey44","grey42","grey40","grey68","grey66","grey64","grey62","grey60","firebrick4","firebrick3","steelblue4","steelblue3","steelblue2","steelblue1")

for(k in 1:length(antigen)){
  control_drm_list <- list()
  
  for(j in 1:length(control_antibody_list)){
    control_drm_list[[j]] <- drm(value ~ variable, data = control_antibody_data[which(control_antibody_data$CellID == control_antibody_list[j] & control_antibody_data$Antigen == antigen[k]),], fct = LL.4(names = c("Slope", "Lower Limit", "Upper Limit", "ED50")))
  }
  
  if (runman) { dev.new(width=5, height=5) }
  plot(x = c(0.001,100),y = c(0,antigen_max_OD450[k]), type = "n", log = "x", xlab = "Primary antibody concentration (nM)", ylab = "OD450", axes = F, main = paste(antigen_full_name[k],"ELISA \n (control antibodies)"))
  axis(side = 1, labels = c("0","0.01","0.1","1","10","100"), at = c(0.001,0.01,0.1,1,10,100))
  axis(side = 2, labels = seq(from = 0, to = antigen_max_OD450[k], by = antigen_max_OD450[k]/5), at = seq(from = 0, to = antigen_max_OD450[k], by = antigen_max_OD450[k]/5))
  
  for(j in 1:length(control_antibody_list)){
    plot(control_drm_list[[j]], broken = FALSE, lwd=2, 
         cex=1.2, cex.axis=1.2, cex.lab=1.2, ylim=c(0,1), col = control_color_list[j], add = T)
  }
  if (runman) { dev.copy(pdf,paste0("./output/",antigen[k],"_ELISA_controls.pdf"),width=5,height=5); dev.off() }
}  
      
  antigen = c("TPO","TG")
antigen_full_name = c("Thyroid peroxidase","Thyroglobulin")
antigen_max_OD450 = c(1,2.5)
test_antibody_data <- synthesised_antibody_ELISA_H1_melt[which(!(grepl("control",synthesised_antibody_ELISA_H1_melt$CellID)) & !(grepl("rabbit",synthesised_antibody_ELISA_H1_melt$CellID))),]

test_antibody_data <- test_antibody_data[order(test_antibody_data$value, decreasing = T),]

test_antibody_list <- unique(test_antibody_data$CellID)
test_antibody_list <- rev(test_antibody_list)

test_color_list <- rev(c("springgreen4","grey20","darkorchid4","darkorchid3","darkorchid2","orchid3","orchid2","grey22","orchid1","grey24","grey26","grey28","grey30","grey32","grey34","grey36","grey38","grey40","grey42","grey44","grey46","grey48","grey50","grey52","grey54","grey56","grey56","grey58","grey60","grey62","grey64","grey66"))

for(k in 1:length(antigen)){
  test_drm_list <- list()
  
  for(j in 1:length(test_antibody_list)){
    test_drm_list[[j]] <- drm(value ~ variable, data = test_antibody_data[which(test_antibody_data$CellID == test_antibody_list[j] & test_antibody_data$Antigen == antigen[k]),], fct = LL.4(names = c("Slope", "Lower Limit", "Upper Limit", "ED50")))
  }
  
  if (runman) { dev.new(width=5, height=5) }
  plot(x = c(0.001,100),y = c(0,antigen_max_OD450[k]), type = "n", log = "x", xlab = "Primary antibody concentration (nM)", ylab = "OD450", axes = F, main = paste(antigen_full_name[k],"ELISA \n (donor H1 antibodies - human)"))
  axis(side = 1, labels = c("0","0.01","0.1","1","10","100"), at = c(0.001,0.01,0.1,1,10,100))
  axis(side = 2, labels = seq(from = 0, to = antigen_max_OD450[k], by = antigen_max_OD450[k]/5), at = seq(from = 0, to = antigen_max_OD450[k], by = antigen_max_OD450[k]/5))
  
  for(j in 1:length(test_antibody_list)){
    plot(test_drm_list[[j]], broken = FALSE, lwd=2, 
         cex=1.2, cex.axis=1.2, cex.lab=1.2, ylim=c(0,1), col = test_color_list[j], add = T)
  }
  if (runman) { dev.copy(pdf,paste0("./output/",antigen[k],"_ELISA_donor_H1.pdf"),width=5,height=5); dev.off() }
}  
      
  antigen = c("TPO","TG")
antigen_full_name = c("Thyroid peroxidase","Thyroglobulin")
antigen_max_OD450 = c(1.5,2.5)
test_antibody_data_rabbit <- synthesised_antibody_ELISA_H1_melt[which(!(grepl("control",synthesised_antibody_ELISA_H1_melt$CellID)) & grepl("rabbit",synthesised_antibody_ELISA_H1_melt$CellID)),]

test_antibody_data_rabbit <- test_antibody_data_rabbit[order(test_antibody_data_rabbit$value, decreasing = T),]
test_antibody_data_rabbit$CellID <- gsub("_rabbit_IgG1","",test_antibody_data_rabbit$CellID)

test_antibody_list_rabbit <- unique(test_antibody_data_rabbit$CellID)
test_antibody_list_rabbit <- rev(test_antibody_list_rabbit)

test_color_list_rabbit <- test_color_list
names(test_color_list_rabbit) <- test_antibody_list
test_color_list_rabbit <- test_color_list_rabbit[test_antibody_list_rabbit]

for(k in 1:length(antigen)){
  test_drm_list_rabbit <- list()
  
  for(j in 1:length(test_antibody_list_rabbit)){
    test_drm_list_rabbit[[j]] <- drm(value ~ variable, data = test_antibody_data_rabbit[which(test_antibody_data_rabbit$CellID == test_antibody_list_rabbit[j] & test_antibody_data_rabbit$Antigen == antigen[k]),], fct = LL.4(names = c("Slope", "Lower Limit", "Upper Limit", "ED50")))
  }
  
  if (runman) { dev.new(width=5, height=5) }
  plot(x = c(0.001,100),y = c(0,antigen_max_OD450[k]), type = "n", log = "x", xlab = "Primary antibody concentration (nM)", ylab = "OD450", axes = F, main = paste(antigen_full_name[k],"ELISA \n (donor H1 antibodies - rabbit)"))
  axis(side = 1, labels = c("0","0.01","0.1","1","10","100"), at = c(0.001,0.01,0.1,1,10,100))
  axis(side = 2, labels = seq(from = 0, to = antigen_max_OD450[k], by = antigen_max_OD450[k]/5), at = seq(from = 0, to = antigen_max_OD450[k], by = antigen_max_OD450[k]/5))
  
  for(j in 1:length(test_antibody_list_rabbit)){
    plot(test_drm_list_rabbit[[j]], broken = FALSE, lwd=2, 
         cex=1.2, cex.axis=1.2, cex.lab=1.2, ylim=c(0,1), col = test_color_list_rabbit[j], add = T)
  }
  if (runman) { dev.copy(pdf,paste0("./output/",antigen[k],"_ELISA_donor_H1_rabbit.pdf"),width=5,height=5); dev.off() }
}  
      
 
 
 20. Single nucleus DNA sequencing from donors H2 and H8 
 For each nucleus, we generated a PTA sequencing library. For the
nuclei from donors H2 and H8, we only carried out targeted sequencing
(i.e. no WGS) using the same panel of 725 genes used for targeted
NanoSeq. Mutations called by targeted NanoSeq were genotyped in the
DNAHyb PTA data. The filtered mutations table shows those genotyped
mutations with convincing support (i.e. VAF ≥ 0.25 & total depth ≥
10). 
  pta_additional_annotation_H2_H8 <- read.table(pta_additional_annotation_H2_H8_file, sep = "\t", stringsAsFactors = F, header = T)
pta_dna_hyb_mutation_H2 <- read.table(pta_dna_hyb_mutation_H2_file, sep = "\t", stringsAsFactors = F, header = T)
pta_dna_hyb_mutation_H8 <- read.table(pta_dna_hyb_mutation_H8_file, sep = "\t", stringsAsFactors = F, header = T)
pta_dna_hyb_mutation_H2_H8 <- rbind(pta_dna_hyb_mutation_H2,pta_dna_hyb_mutation_H8)

pta_dna_hyb_mutation_H2_H8$type <- NA
pta_dna_hyb_mutation_H2_H8$type[which(nchar(pta_dna_hyb_mutation_H2_H8$ref) == 1 & nchar(pta_dna_hyb_mutation_H2_H8$alt) == 1)] <- "snv"
pta_dna_hyb_mutation_H2_H8$type[which(nchar(pta_dna_hyb_mutation_H2_H8$ref) > nchar(pta_dna_hyb_mutation_H2_H8$alt))] <- "del"
pta_dna_hyb_mutation_H2_H8$type[which(nchar(pta_dna_hyb_mutation_H2_H8$ref) < nchar(pta_dna_hyb_mutation_H2_H8$alt))] <- "ins"
pta_dna_hyb_mutation_H2_H8$type[which(nchar(pta_dna_hyb_mutation_H2_H8$ref) == nchar(pta_dna_hyb_mutation_H2_H8$alt) & nchar(pta_dna_hyb_mutation_H2_H8$ref) != 1)] <- "mnv"  
 Rescued structural variants affecting CD274 in donor H8. It is often
difficult to be certain that individual SVs are genuine in targeted
sequencing data as unlikely to observe breakpoint. However, these are
particularly compelling as they occur across multiple cells within a
clade. 
  pta_dna_hyb_rescued_muts_H8 <- read_xlsx(pta_dna_hyb_rescued_muts_H8_file)
pta_dna_hyb_mutation_H2_H8 <- rbind(pta_dna_hyb_mutation_H2_H8, pta_dna_hyb_rescued_muts_H8)  
  pta_additional_annotation_H2_H8$total_muts <- NA
pta_additional_annotation_H2_H8$muts_in_immune_loci <- NA

for(x in 1:nrow(pta_additional_annotation_H2_H8)){
  temp_muts <- pta_dna_hyb_mutation_H2_H8[which(gsub("_dnahyb.*","",pta_dna_hyb_mutation_H2_H8$id) == pta_additional_annotation_H2_H8$well_ID[x]),]
  
  pta_additional_annotation_H2_H8$total_muts[x] <- nrow(temp_muts)
  pta_additional_annotation_H2_H8$muts_in_immune_loci[x] <- length(which(((temp_muts$chr == "14" & temp_muts$pos >= 106053226 & temp_muts$pos <= 107288019)|(temp_muts$chr == "22" & temp_muts$pos >= 22380474 & temp_muts$pos <= 23265203))))
}

pta_additional_annotation_H2_H8$prop_immune_loci <- pta_additional_annotation_H2_H8$muts_in_immune_loci / pta_additional_annotation_H2_H8$total_muts
pta_additional_annotation_H2_H8$prop_immune_loci[which(is.nan(pta_additional_annotation_H2_H8$prop_immune_loci))] <- 0

pta_additional_annotation_H2_H8$celltype_SHM <- NA
pta_additional_annotation_H2_H8$celltype_SHM[pta_additional_annotation_H2_H8$prop_immune_loci >= 0.1 & pta_additional_annotation_H2_H8$muts_in_immune_loci >= 3] <- "Mature B cell"
pta_additional_annotation_H2_H8$celltype_SHM[(pta_additional_annotation_H2_H8$prop_immune_loci < 0.1 & pta_additional_annotation_H2_H8$prop_immune_loci > 0) | (pta_additional_annotation_H2_H8$muts_in_immune_loci < 3 & pta_additional_annotation_H2_H8$muts_in_immune_loci > 0)] <- "Uncertain"
pta_additional_annotation_H2_H8$celltype_SHM[pta_additional_annotation_H2_H8$muts_in_immune_loci == 0] <- "Not mature B cell"

ggplot() +
  geom_point(data = pta_additional_annotation_H2_H8[!(is.nan(pta_additional_annotation_H2_H8$prop_immune_loci)),], aes(x = muts_in_immune_loci, y = prop_immune_loci, color = celltype_VDJ_recomb), alpha = 0.5) +
  facet_grid(substr(x = pta_additional_annotation_H2_H8$cell_ID[!(is.nan(pta_additional_annotation_H2_H8$prop_immune_loci))],start = 1,stop = 2) ~ celltype_VDJ_recomb) +
  geom_vline(data = pta_additional_annotation_H2_H8[!(is.nan(pta_additional_annotation_H2_H8$prop_immune_loci)),], aes(xintercept = 3), colour = "red", lty = 2) +
  geom_hline(data = pta_additional_annotation_H2_H8[!(is.nan(pta_additional_annotation_H2_H8$prop_immune_loci)),], aes(yintercept = 0.1), colour = "red", lty = 2) +
  theme_bw() +
  theme(panel.grid.major = element_blank(), panel.grid.minor = element_blank()) +
  scale_x_continuous(expand = c(0,1), limits = c(0,50)) +
  scale_y_continuous(expand = c(0,0.01), limits = c(0,1)) +
  labs(x = "Number of immune loci mutations", y = "Proportion of genotyped \n mutations in immune loci", color = "V(D)J cell type")  
    
  ggplot(pta_additional_annotation_H2_H8) +
  geom_point(aes(x = productive_heavy_chain_read_support, y = productive_TCRA_read_support, color = celltype_SHM), alpha = 0.5) +
  theme_bw() +
  theme(panel.grid.major = element_blank(), panel.grid.minor = element_blank()) +
  labs(x = "Reads supporting productive \n Ig heavy chain rearrangement", y = "Reads supporting productive \n TCRA rearrangement", color = "SHM cell type") +
  facet_grid(substr(x = pta_additional_annotation_H2_H8$cell_ID[!(is.nan(pta_additional_annotation_H2_H8$prop_immune_loci))],start = 1,stop = 2) ~ celltype_SHM)  
    
  ggplot(pta_additional_annotation_H2_H8) +
  geom_point((aes(x = productive_heavy_chain_read_support, y = productive_TCRA_read_support, color = celltype_VDJ_recomb)), alpha = 0.5) +
  theme_bw() +
  theme(panel.grid.major = element_blank(), panel.grid.minor = element_blank()) +
  labs(x = "Reads supporting productive Ig heavy chain rearrangement", y = "Reads supporting productive \n TCRA rearrangement", color = "V(D)J cell type") +
  facet_grid(substr(x = pta_additional_annotation_H2_H8$cell_ID[!(is.nan(pta_additional_annotation_H2_H8$prop_immune_loci))],start = 1,stop = 2) ~ celltype_VDJ_recomb)  
    
  ggplot(pta_additional_annotation_H2_H8) +
  geom_point((aes(x = median_dna_hyb_cov, y = mean_csr_coverage_hyb, color = celltype_VDJ_recomb)), alpha = 0.5) +
  theme_bw() +
  theme(panel.grid = element_blank()) +
  scale_x_continuous(limits = c(0,1000)) +
  labs(x = "Median DNA Hyb depth", y = "CSR depth", color = "V(D)J cell type") +
  facet_grid(substr(x = pta_additional_annotation_H2_H8$cell_ID[!(is.nan(pta_additional_annotation_H2_H8$prop_immune_loci))],start = 1,stop = 2) ~ celltype_VDJ_recomb) + 
  geom_hline(aes(yintercept = 1), lty = 2, color = "red")  
    
  pta_additional_annotation_H2_H8$csr <- NA
pta_additional_annotation_H2_H8$csr[which(pta_additional_annotation_H2_H8$mean_csr_coverage_hyb >= 1)] <- 0
pta_additional_annotation_H2_H8$csr[which(pta_additional_annotation_H2_H8$mean_csr_coverage_hyb < 1)] <- 1  
  pta_dna_hyb_mutation_H2_H8$paper_cell_id <- NA
pta_dna_hyb_mutation_H2_H8$paper_id <- NA
pta_dna_hyb_mutation_H2_H8$cell_id <- NA

for(x in 1:nrow(pta_dna_hyb_mutation_H2_H8)){
  pta_dna_hyb_mutation_H2_H8$paper_cell_id[which(gsub("_dnahyb.*","",pta_dna_hyb_mutation_H2_H8$id) == pta_additional_annotation_H2_H8$well_ID[x])] <-  pta_additional_annotation_H2_H8$cell_ID[x]
}

pta_dna_hyb_mutation_H2_H8$paper_id <- gsub("_.*","",pta_dna_hyb_mutation_H2_H8$paper_cell_id)
pta_dna_hyb_mutation_H2_H8$cell_id <- as.numeric(gsub(".*_","",pta_dna_hyb_mutation_H2_H8$paper_cell_id))

pta_dna_hyb_mutation_H2_H8 <- pta_dna_hyb_mutation_H2_H8[order(pta_dna_hyb_mutation_H2_H8$paper_id,pta_dna_hyb_mutation_H2_H8$cell_id, decreasing = F),]  
  pta_dna_hyb_mutation_H2_H8$mut_site <- paste(pta_dna_hyb_mutation_H2_H8$chr,pta_dna_hyb_mutation_H2_H8$pos,pta_dna_hyb_mutation_H2_H8$ref,pta_dna_hyb_mutation_H2_H8$alt,sep = "_")
pta_dna_hyb_mutation_H2_H8$merged_impact <- paste(pta_dna_hyb_mutation_H2_H8$gene,pta_dna_hyb_mutation_H2_H8$aachange,pta_dna_hyb_mutation_H2_H8$impact,sep = "_")

pta_dna_hyb_mutation_H2_H8$heatmap_annot <- NA

for(x in 1:nrow(pta_dna_hyb_mutation_H2_H8)){
  if(!(is.na(pta_dna_hyb_mutation_H2_H8$impact[x]))){
    if(pta_dna_hyb_mutation_H2_H8$impact[x] %in% c("Missense","Nonsense","Synonymous")){
      pta_dna_hyb_mutation_H2_H8$heatmap_annot[x] <- paste(pta_dna_hyb_mutation_H2_H8$gene[x], pta_dna_hyb_mutation_H2_H8$aachange[x], sep = "_")
    }else if(pta_dna_hyb_mutation_H2_H8$impact[x] =="Essential_Splice"){
      pta_dna_hyb_mutation_H2_H8$heatmap_annot[x] <- paste(pta_dna_hyb_mutation_H2_H8$gene[x],"Splice",  sep = "_")
    }else if(pta_dna_hyb_mutation_H2_H8$type[x] == "del"){
      pta_dna_hyb_mutation_H2_H8$heatmap_annot[x] <- paste0(pta_dna_hyb_mutation_H2_H8$gene[x],"_Del_",nchar(pta_dna_hyb_mutation_H2_H8$ref[x]) - 1,"bp")
    }else if(pta_dna_hyb_mutation_H2_H8$type[x] == "ins"){
      pta_dna_hyb_mutation_H2_H8$heatmap_annot[x] <- paste0(pta_dna_hyb_mutation_H2_H8$gene[x],"_Ins_",nchar(pta_dna_hyb_mutation_H2_H8$alt[x]) - 1,"bp")
    }else{
      pta_dna_hyb_mutation_H2_H8$heatmap_annot[x] <- paste0(pta_dna_hyb_mutation_H2_H8$gene[x],"_",pta_dna_hyb_mutation_H2_H8$type[x])
    }
  }
}

heatmap_annot_vec <- unique(pta_dna_hyb_mutation_H2_H8$heatmap_annot)
for(x in 1:length(heatmap_annot_vec)){
  mut_site_vec <- unique(pta_dna_hyb_mutation_H2_H8$mut_site[which(pta_dna_hyb_mutation_H2_H8$heatmap_annot == heatmap_annot_vec[x])])
  if(length(mut_site_vec) > 1){
    for(y in 1:length(mut_site_vec)){
      pta_dna_hyb_mutation_H2_H8$heatmap_annot[which(pta_dna_hyb_mutation_H2_H8$mut_site == mut_site_vec[y])] <- paste0(heatmap_annot_vec[x],"_#",y)
    }
  }
}  
  donors <- c("H2","H8")

for(z in 1:length(donors)){
  pta_additional_annotation_single_donor <- pta_additional_annotation_H2_H8[which(substr(pta_additional_annotation_H2_H8$cell_ID,1,2) == donors[z]),]
  pta_dna_hyb_mutation_single_donor <- pta_dna_hyb_mutation_H2_H8[which(pta_dna_hyb_mutation_H2_H8$paper_id == donors[z]),]
  
  pta_additional_annotation_single_donor$cell_ID <- as.numeric(gsub(paste0(donors[z],"_"),"",pta_additional_annotation_single_donor$cell_ID))
  
  empty_frame <- as.data.frame(array(data = NA, dim = c(length(unique(pta_additional_annotation_single_donor$cell_ID)),1)))
  colnames(empty_frame) <- "cell_id"
  empty_frame$cell_id <- unique(pta_additional_annotation_single_donor$cell_ID)
  
  color.palette = colorRampPalette(c("white", "navy"))
  wgs.palette = colorRampPalette(c("white","lightgoldenrod1"))
  vdj.color.palette = colorRampPalette(c("lightcyan","lightcoral","palegreen1"))
  shm.color.palette = colorRampPalette(c("white","peachpuff","lightyellow1"))
  hap.color.palette = colorRampPalette(c("white","lightpink","lightblue"))
  csr.color.palette = colorRampPalette(c("white","thistle"))
  sv.color.palette = colorRampPalette(c("white","firebrick"))
  vj.color.palette = colorRampPalette(c("white","springgreen4"))
  cdr3.color.palette = colorRampPalette(c("moccasin","navajowhite4"))
  breakpoint.color.palette = colorRampPalette(c("white","darkmagenta"))
  antibody.color.palette = colorRampPalette(c("red","white"))
  
  multicell_clades <- unique(pta_additional_annotation_single_donor$clade_ID[duplicated(pta_additional_annotation_single_donor$clade_ID)])
  multicell_clade_lims <- vector(length = 2*length(multicell_clades))
  
  if(length(multicell_clade_lims) > 0){
    for(x in 1:length(multicell_clades)){
      multicell_clade_lims[x] <- -1 * (max(pta_additional_annotation_single_donor$cell_ID[pta_additional_annotation_single_donor$clade_ID == multicell_clades[x]]) + 0.5)
      multicell_clade_lims[x + length(multicell_clades)] <- -1 * (min(pta_additional_annotation_single_donor$cell_ID[pta_additional_annotation_single_donor$clade_ID == multicell_clades[x]]) - 0.5)
    }
    multicell_clade_lims <- sort(unique(multicell_clade_lims))
  }
  
  pta_additional_annotation_single_donor$subclade_ID[is.na(pta_additional_annotation_single_donor$subclade_ID)] <- ""
  
  subclades <- unique(pta_additional_annotation_single_donor$subclade_ID)
  subclades <- subclades[which(subclades != "")]
  subclade_lims <- vector(length = length(subclades))
  
  if(length(subclade_lims) > 0){
    for(x in 1:length(subclade_lims)){
      subclade_lims[x] <- -1 * (max(pta_additional_annotation_single_donor$cell_ID[pta_additional_annotation_single_donor$subclade_ID == subclades[x]]) + 0.5)
    }
    
    subclade_lims <- subclade_lims[which(!(subclade_lims %in% multicell_clade_lims))]
  }
  
  pta_cell_type <- reshape2::melt(pta_additional_annotation_single_donor[,c("cell_ID","celltype_VDJ_recomb","celltype_SHM","csr")], id.vars = "cell_ID")
  pta_cell_type$value[which(pta_cell_type$value %in% c("doublet","not lymphocyte","Uncertain"))] <- 0
  pta_cell_type$value[which(pta_cell_type$value %in% c("B cell","Mature B cell"))]  <- 1
  pta_cell_type$value[which(pta_cell_type$value %in% c("alpha-beta T cell","Not mature B cell"))]  <- 2
  
  pta_cell_type$value <- as.numeric(pta_cell_type$value)
  
  # Create SHM heatmap
  cell_type_SHM = levelplot(value~variable*-cell_ID, pta_cell_type[which(pta_cell_type$variable == "celltype_SHM"),], col.regions=shm.color.palette, scales = list(tck = c(0,0), y = list(cex=1, at = seq(from = -1, to = -1 * nrow(empty_frame), by = -1), labels = c(1:nrow(empty_frame))), x = list(alternating = 3, rot = 90)), ylab = NULL, xlab="", colorkey=FALSE, 
                            panel=function(...) { arg <- list(...)
                            panel.levelplot(...)
                            panel.abline(h = multicell_clade_lims)
                            panel.abline(h = subclade_lims, lty = 2)})
  
  # Create VDJ heatmap
  cell_type_VDJ = levelplot(value~variable*-cell_ID, pta_cell_type[which(pta_cell_type$variable == "celltype_VDJ_recomb"),], col.regions=vdj.color.palette, scales = list(tck = c(0,0), y = list(cex=1, at = seq(from = -1, to = -1 * nrow(empty_frame), by = -1), labels = c(1:nrow(empty_frame))), x = list(alternating = 3, rot = 90)), ylab = NULL, xlab="", colorkey=FALSE,
                            panel=function(...) { arg <- list(...)
                            panel.levelplot(...)
                            panel.abline(h = multicell_clade_lims)
                            panel.abline(h = subclade_lims, lty = 2)})
  
  # Create CSR heatmap
cell_type_csr = levelplot(value~variable*-cell_ID, pta_cell_type[which(pta_cell_type$variable == "csr"),], col.regions=csr.color.palette, scales = list(tck = c(0,0), y = list(cex=1, at = seq(from = -1, to = -1 * nrow(empty_frame), by = -1), labels = c(1:nrow(empty_frame))), x = list(alternating = 3, rot = 90)), ylab = NULL, xlab="", colorkey=FALSE,
                          panel=function(...) { arg <- list(...)
                          panel.levelplot(...)
                          panel.abline(h = multicell_clade_lims)
                          panel.abline(h = subclade_lims, lty = 2)})
  
  pta_TNFRSF14_muts <- pta_dna_hyb_mutation_single_donor[which(pta_dna_hyb_mutation_single_donor$gene == "TNFRSF14"),c("cell_id","heatmap_annot","alt_vaf")]
  pta_TNFRSF14_muts$heatmap_annot <- gsub("TNFRSF14_","",pta_TNFRSF14_muts$heatmap_annot)
  pta_TNFRSF14_mut_array <- reshape2::dcast(pta_TNFRSF14_muts, cell_id ~ heatmap_annot, value.var = "alt_vaf")
  pta_TNFRSF14_mut_array <- pta_TNFRSF14_mut_array[,c("cell_id",unique(pta_TNFRSF14_muts$heatmap_annot))]
  
  pta_TNFRSF14_mut_array <- left_join(empty_frame, pta_TNFRSF14_mut_array, by = "cell_id")
  pta_TNFRSF14_muts <- reshape2::melt(pta_TNFRSF14_mut_array, id.var = "cell_id")
  pta_TNFRSF14_muts$value[is.na(pta_TNFRSF14_muts$value)] <- 0 
  
  TNFRSF14_vaf_heatmap <- lattice::levelplot(value~variable*-cell_id, data = pta_TNFRSF14_muts, col.regions=color.palette(1000), scales = list(tck = c(0,0), y = list(cex=1, at = seq(from = -1, to = -1 * nrow(empty_frame), by = -1), labels = c(1:nrow(empty_frame))), x = list(alternating = 3, rot = 90)), ylab="Cell ID", xlab = "TNFRSF14", colorkey=list(space="top"), at = seq(from = 0, to = 1, by = 0.001), main = list(label = "VAF"), panel=function(...) { arg <- list(...)
  panel.levelplot(...)
  panel.abline(h = multicell_clade_lims)
  panel.abline(h = subclade_lims, lty = 2)}, aspect = ncol(pta_TNFRSF14_mut_array))
  
  pta_CD274_muts <- pta_dna_hyb_mutation_single_donor[which(pta_dna_hyb_mutation_single_donor$gene == "CD274"),c("cell_id","heatmap_annot","alt_vaf")]
  
  if(nrow(pta_CD274_muts) > 0){
    pta_CD274_muts$heatmap_annot <- gsub("CD274_","",pta_CD274_muts$heatmap_annot)
    pta_CD274_mut_array <- reshape2::dcast(pta_CD274_muts, cell_id ~ heatmap_annot, value.var = "alt_vaf")
    pta_CD274_mut_array <- pta_CD274_mut_array[,c("cell_id",unique(pta_CD274_muts$heatmap_annot))]
    
    pta_CD274_mut_array <- left_join(empty_frame, pta_CD274_mut_array, by = "cell_id")
    pta_CD274_muts <- reshape2::melt(pta_CD274_mut_array, id.var = "cell_id")
    pta_CD274_muts$value[is.na(pta_CD274_muts$value)] <- 0 
    
    CD274_vaf_heatmap <- lattice::levelplot(value~variable*-cell_id, data = pta_CD274_muts, col.regions=color.palette(1000), scales = list(tck = c(0,0), y = list(cex=1, at = seq(from = -1, to = -1 * nrow(empty_frame), by = -1), labels = c(1:nrow(empty_frame))), x = list(alternating = 3, rot = 90)), ylab="Cell ID", xlab = "CD274", colorkey=list(space="top"), at = seq(from = 0, to = 1, by = 0.001), main = list(label = "VAF"), panel=function(...) { arg <- list(...)
    panel.levelplot(...)
    panel.abline(h = multicell_clade_lims)
    panel.abline(h = subclade_lims, lty = 2)})
  }
  
  pta_other_gene_muts <- pta_dna_hyb_mutation_single_donor[which(pta_dna_hyb_mutation_single_donor$gene %in% c("DNMT3A","TET2","RASA2") & pta_dna_hyb_mutation_single_donor$impact != "Synonymous"),c("cell_id","heatmap_annot","alt_vaf")]
  
  if(nrow(pta_other_gene_muts) > 0){
    pta_other_gene_mut_array <- reshape2::dcast(pta_other_gene_muts, cell_id ~ heatmap_annot, value.var = "alt_vaf")
    pta_other_gene_mut_array <- pta_other_gene_mut_array[,c("cell_id",unique(pta_other_gene_muts$heatmap_annot))]
    
    pta_other_gene_mut_array <- pta_other_gene_mut_array[,c(1,
                                                            which(grepl("DNMT3A",colnames(pta_other_gene_mut_array))),
                                                            which(grepl("TET2",colnames(pta_other_gene_mut_array))),
                                                            which(grepl("RASA2",colnames(pta_other_gene_mut_array))))]
    
    pta_other_gene_mut_array <- left_join(empty_frame, pta_other_gene_mut_array, by = "cell_id")
    pta_other_gene_muts <- reshape2::melt(pta_other_gene_mut_array, id.var = "cell_id")
    pta_other_gene_muts$value[is.na(pta_other_gene_muts$value)] <- 0 
    
    other_gene_heatmap <- lattice::levelplot(value~variable*-cell_id, data = pta_other_gene_muts, col.regions=color.palette(1000), scales = list(tck = c(0,0), y = list(cex=1, at = seq(from = -1, to = -1 * nrow(empty_frame), by = -1), labels = c(1:nrow(empty_frame))), x = list(alternating = 3, rot = 90)), ylab="Cell ID", xlab = "Selected genes", colorkey=list(space="top", at = seq(from = 0, to = 1, by = 0.001), title = "VAF"), main = list(label = "VAF"), panel=function(...) { arg <- list(...)
    panel.levelplot(...)
    panel.abline(h = multicell_clade_lims)
    panel.abline(h = subclade_lims, lty = 2)})
  }
  
  pta_additional_annotation_single_donor$heavy_V_consensus <- NA
  pta_additional_annotation_single_donor$heavy_J_consensus <- NA
  pta_additional_annotation_single_donor$TCRA_V_consensus <- NA
  pta_additional_annotation_single_donor$TCRA_J_consensus <- NA
  
  for(x in 1:nrow(pta_additional_annotation_single_donor)){
    heavy_V_temp <- gsub("\\*.*","",unlist(strsplit(pta_additional_annotation_single_donor$productive_heavy_chain_V[x], split = ",")))
    heavy_V_temp <- gsub("IGHV","",heavy_V_temp)
    if(length(unique(heavy_V_temp)) == 1){
      pta_additional_annotation_single_donor$heavy_V_consensus[x] <- unique(heavy_V_temp)
    }else if(length(heavy_V_temp) > 2){
      pta_additional_annotation_single_donor$heavy_V_consensus[x] <- names(table(heavy_V_temp)[which(table(heavy_V_temp) == max(table(heavy_V_temp)))])
    }else if(length(unique(heavy_V_temp)) == 2){
      sb <- stri_sub(unique(heavy_V_temp)[2], 1, 1:nchar(unique(heavy_V_temp)[2]))
      sstr <- na.omit(stri_extract_all_coll(unique(heavy_V_temp)[1], sb, simplify=TRUE))
      if(length(sstr[which.max(nchar(sstr))]) > 0){
        pta_additional_annotation_single_donor$heavy_V_consensus[x] <- sstr[which.max(nchar(sstr))]
      }else{
        pta_additional_annotation_single_donor$heavy_V_consensus[x] <- ""
      }
    }
    
    
    heavy_J_temp <- gsub("\\*.*","",unlist(strsplit(pta_additional_annotation_single_donor$productive_heavy_chain_J[x], split = ",")))
    heavy_J_temp <- gsub("IGHJ","",heavy_J_temp)
    if(length(unique(heavy_J_temp)) == 1){
      pta_additional_annotation_single_donor$heavy_J_consensus[x] <- unique(heavy_J_temp)
    }else if(length(heavy_J_temp) > 2){
      pta_additional_annotation_single_donor$heavy_J_consensus[x] <- names(table(heavy_J_temp)[which(table(heavy_J_temp) == max(table(heavy_J_temp)))])
    }else if(length(unique(heavy_J_temp)) == 2){
      sb <- stri_sub(unique(heavy_J_temp)[2], 1, 1:nchar(unique(heavy_J_temp)[2]))
      sstr <- na.omit(stri_extract_all_coll(unique(heavy_J_temp)[1], sb, simplify=TRUE))
      if(length(sstr[which.max(nchar(sstr))]) > 0){
        pta_additional_annotation_single_donor$heavy_J_consensus[x] <- sstr[which.max(nchar(sstr))]
      }else{
        pta_additional_annotation_single_donor$heavy_J_consensus[x] <- ""
      }
    }
    
    TCRA_V_temp <- gsub("\\*.*","",unlist(strsplit(pta_additional_annotation_single_donor$productive_TCRA_chain_V[x], split = ",")))
    TCRA_V_temp <- gsub("TRAV","",TCRA_V_temp)
    if(length(unique(TCRA_V_temp)) == 1){
      pta_additional_annotation_single_donor$TCRA_V_consensus[x] <- unique(TCRA_V_temp)
    }else if(length(TCRA_V_temp) > 2){
      pta_additional_annotation_single_donor$TCRA_V_consensus[x] <- names(table(TCRA_V_temp)[which(table(TCRA_V_temp) == max(table(TCRA_V_temp)))])
    }else if(length(unique(TCRA_V_temp)) == 2){
      sb <- stri_sub(unique(TCRA_V_temp)[2], 1, 1:nchar(unique(TCRA_V_temp)[2]))
      sstr <- na.omit(stri_extract_all_coll(unique(TCRA_V_temp)[1], sb, simplify=TRUE))
      if(length(sstr[which.max(nchar(sstr))]) > 0){
        pta_additional_annotation_single_donor$TCRA_V_consensus[x] <- sstr[which.max(nchar(sstr))]
      }else{
        pta_additional_annotation_single_donor$TCRA_V_consensus[x] <- ""
      }
    }
    
    TCRA_J_temp <- gsub("\\*.*","",unlist(strsplit(pta_additional_annotation_single_donor$productive_TCRA_chain_J[x], split = ",")))
    TCRA_J_temp <- gsub("TRAJ","",TCRA_J_temp)
    if(length(unique(TCRA_J_temp)) == 1){
      pta_additional_annotation_single_donor$TCRA_J_consensus[x] <- unique(TCRA_J_temp)
    }else if(length(TCRA_J_temp) > 2){
      pta_additional_annotation_single_donor$TCRA_J_consensus[x] <- names(table(TCRA_J_temp)[which(table(TCRA_J_temp) == max(table(TCRA_J_temp)))])
    }else if(length(unique(TCRA_J_temp)) == 2){
      sb <- stri_sub(unique(TCRA_J_temp)[2], 1, 1:nchar(unique(TCRA_J_temp)[2]))
      sstr <- na.omit(stri_extract_all_coll(unique(TCRA_J_temp)[1], sb, simplify=TRUE))
      if(length(sstr[which.max(nchar(sstr))]) > 0){
        pta_additional_annotation_single_donor$TCRA_J_consensus[x] <- sstr[which.max(nchar(sstr))]
      }else{
        pta_additional_annotation_single_donor$TCRA_J_consensus[x] <- ""
      }
    }
  }
  
  pta_additional_annotation_single_donor$TCRA_V_consensus <- gsub("/DV.*","",pta_additional_annotation_single_donor$TCRA_V_consensus)
  
  pta_additional_annotation_single_donor$TCRA_V_consensus[which(pta_additional_annotation_single_donor$TCRA_V_consensus %in% names(table(pta_additional_annotation_single_donor$TCRA_V_consensus)[which(table(pta_additional_annotation_single_donor$TCRA_V_consensus) == 1)]))] <- "Unique"
  
  pta_additional_annotation_single_donor$TCRA_J_consensus[which(pta_additional_annotation_single_donor$TCRA_J_consensus %in% names(table(pta_additional_annotation_single_donor$TCRA_J_consensus)[which(table(pta_additional_annotation_single_donor$TCRA_J_consensus) == 1)]))] <- "Unique"
  
  pta_additional_annotation_single_donor$heavy_V_consensus[which(pta_additional_annotation_single_donor$heavy_V_consensus %in% names(table(pta_additional_annotation_single_donor$heavy_V_consensus)[which(table(pta_additional_annotation_single_donor$heavy_V_consensus) == 1)]))] <- "Unique"
  
  igh_v_categories <- unique(pta_additional_annotation_single_donor$heavy_V_consensus)
  igh_v_categories <- gsub("_","-",mixedsort(gsub("-","_",igh_v_categories)))
  igh_v_categories <- igh_v_categories[which(igh_v_categories != "")]
  igh_v_categories <- igh_v_categories[which(igh_v_categories != "-")]
  
  igh_v_array <- as.data.frame(array(data = 0, dim = c(nrow(empty_frame),length(igh_v_categories))))
  
  for(x in 1:length(igh_v_categories)){
    for(y in 1:nrow(empty_frame)){
      if(igh_v_categories[x] == pta_additional_annotation_single_donor$heavy_V_consensus[y]){
        igh_v_array[y,x] <- log2(pta_additional_annotation_single_donor$productive_heavy_chain_read_support[y])
      }
    }
  }
  
  colnames(igh_v_array) <- igh_v_categories
  
  igh_v_array$cell_id <- 1:nrow(igh_v_array)
  
  igh_v_tidy <- reshape2::melt(igh_v_array, id.var = "cell_id")
  
  igh_v_heatmap <- lattice::levelplot(value~variable*-cell_id, data = igh_v_tidy, col.regions=vj.color.palette(1000), scales = list(tck = c(0,0), y = list(cex=1, at = seq(from = -1, to = -1 * nrow(empty_frame), by = -1), labels = c(1:nrow(empty_frame))), x = list(alternating = 3, rot = 90)), ylab="Cell ID", xlab = "", colorkey=list(space="top"), at = seq(from = 0, to = 10, by = 0.01), main = list(label = "log2 V(D)J read support"), panel=function(...) { arg <- list(...)
  panel.levelplot(...)
  panel.abline(h = multicell_clade_lims)
  panel.abline(h = subclade_lims, lty = 2)})
  
  igh_j_categories <- unique(pta_additional_annotation_single_donor$heavy_J_consensus)
  igh_j_categories <- gsub("_","-",mixedsort(gsub("-","_",igh_j_categories)))
  igh_j_categories <- igh_j_categories[which(igh_j_categories != "")]
  igh_j_categories <- igh_j_categories[which(igh_j_categories != "-")]
  
  igh_j_array <- as.data.frame(array(data = 0, dim = c(nrow(empty_frame),length(igh_j_categories))))
  
  for(x in 1:length(igh_j_categories)){
    for(y in 1:nrow(empty_frame)){
      if(igh_j_categories[x] == pta_additional_annotation_single_donor$heavy_J_consensus[y]){
        igh_j_array[y,x] <- log2(pta_additional_annotation_single_donor$productive_heavy_chain_read_support[y])
      }
    }
  }
  
  colnames(igh_j_array) <- igh_j_categories
  
  igh_j_array$cell_id <- 1:nrow(igh_j_array)
  
  igh_j_tidy <- reshape2::melt(igh_j_array, id.var = "cell_id")
  
  igh_j_heatmap <- lattice::levelplot(value~variable*-cell_id, data = igh_j_tidy, col.regions=vj.color.palette(1000), scales = list(tck = c(0,0), y = list(cex=1, at = seq(from = -1, to = -1 * nrow(empty_frame), by = -1), labels = c(1:nrow(empty_frame))), x = list(alternating = 3, rot = 90)), ylab="Cell ID", xlab = "", colorkey=list(space="top"), at = seq(from = 0, to = 10, by = 0.01), main = list(label = "log2 V(D)J read support"), panel=function(...) { arg <- list(...)
  panel.levelplot(...)
  panel.abline(h = multicell_clade_lims)
  panel.abline(h = subclade_lims, lty = 2)})
  
  pta_IgH_CDR3_input <- pta_additional_annotation_single_donor[,c("cell_ID","productive_heavy_chain_CDR3_nt_length")]
  pta_IgH_CDR3_input$productive_heavy_chain_CDR3_nt_length[which(pta_additional_annotation_single_donor$productive_heavy_chain_read_support <= 1)] <- "-"
  
  pta_IgH_CDR3_input$productive_heavy_chain_CDR3_nt_length[which(pta_IgH_CDR3_input$productive_heavy_chain_CDR3_nt_length == "-")] <- NA
  
  pta_IgH_CDR3_input$productive_heavy_chain_CDR3_nt_length <- as.numeric(pta_IgH_CDR3_input$productive_heavy_chain_CDR3_nt_length)
  
  pta_IgH_CDR3_input$productive_heavy_chain_CDR3_nt_length <- pta_IgH_CDR3_input$productive_heavy_chain_CDR3_nt_length / 3
  
  colnames(pta_IgH_CDR3_input) <- c("cell_ID","Ig_heavy_CDR3_length")
  
  pta_IgH_CDR3 <- reshape2::melt(pta_IgH_CDR3_input, id.vars = "cell_ID")
  pta_IgH_CDR3$value[pta_IgH_CDR3$value == "-"] <- 0
  pta_IgH_CDR3$value <- as.numeric(pta_IgH_CDR3$value)
  
  
  # Create haplotype heatmap
  pta_IgH_CDR3_heatmap = levelplot(value~variable*-cell_ID, pta_IgH_CDR3, col.regions=cdr3.color.palette(30), scales = list(tck = c(0,0), y = list(cex=1, at = seq(from = -1, to = -1 * nrow(empty_frame), by = -1), labels = c(1:nrow(empty_frame))), x = list(alternating = 3, rot = 90)), ylab = NULL, xlab="", colorkey=list(space="top"), at = seq(from = 0, to = 29, by = 1), main = list(label = "CDR3 aa length"), 
                                   , panel=function(...) { arg <- list(...)
                                   panel.levelplot(...)
                                   panel.abline(h = multicell_clade_lims)
                                   panel.abline(h = subclade_lims, lty = 2)})
  
  tcra_v_categories <- unique(pta_additional_annotation_single_donor$TCRA_V_consensus)
  tcra_v_categories <- gsub("_","-",mixedsort(gsub("-","_",tcra_v_categories)))
  tcra_v_categories <- tcra_v_categories[which(tcra_v_categories != "")]
  tcra_v_categories <- tcra_v_categories[which(tcra_v_categories != "-")]
  
  tcra_v_array <- as.data.frame(array(data = 0, dim = c(nrow(empty_frame),length(tcra_v_categories))))
  
  for(x in 1:length(tcra_v_categories)){
    for(y in 1:nrow(empty_frame)){
      if(tcra_v_categories[x] == pta_additional_annotation_single_donor$TCRA_V_consensus[y]){
        tcra_v_array[y,x] <- log2(pta_additional_annotation_single_donor$productive_TCRA_read_support[y])
      }
    }
  }
  
  colnames(tcra_v_array) <- tcra_v_categories
  
  tcra_v_array$cell_id <- 1:nrow(tcra_v_array)
  
  tcra_v_tidy <- reshape2::melt(tcra_v_array, id.var = "cell_id")
  
  tcra_v_heatmap <- lattice::levelplot(value~variable*-cell_id, data = tcra_v_tidy, col.regions=vj.color.palette(1000), scales = list(tck = c(0,0), y = list(cex=1, at = seq(from = -1, to = -1 * nrow(empty_frame), by = -1), labels = c(1:nrow(empty_frame))), x = list(alternating = 3, rot = 90)), ylab="Cell ID", xlab = "", colorkey=list(space="top"), at = seq(from = 0, to = 10, by = 0.01), main = list(label = "log2 V(D)J read support"), panel=function(...) { arg <- list(...)
  panel.levelplot(...)
  panel.abline(h = multicell_clade_lims)
  panel.abline(h = subclade_lims, lty = 2)})
  
  tcra_j_categories <- unique(pta_additional_annotation_single_donor$TCRA_J_consensus)
  tcra_j_categories <- gsub("_","-",mixedsort(gsub("-","_",tcra_j_categories)))
  tcra_j_categories <- tcra_j_categories[which(tcra_j_categories != "")]
  tcra_j_categories <- tcra_j_categories[which(tcra_j_categories != "-")]
  
  tcra_j_array <- as.data.frame(array(data = 0, dim = c(nrow(empty_frame),length(tcra_j_categories))))
  
  for(x in 1:length(tcra_j_categories)){
    for(y in 1:nrow(empty_frame)){
      if(tcra_j_categories[x] == pta_additional_annotation_single_donor$TCRA_J_consensus[y]){
        tcra_j_array[y,x] <- log2(pta_additional_annotation_single_donor$productive_TCRA_read_support[y])
      }
    }
  }
  
  colnames(tcra_j_array) <- tcra_j_categories
  
  tcra_j_array$cell_id <- 1:nrow(tcra_j_array)
  
  tcra_j_tidy <- reshape2::melt(tcra_j_array, id.var = "cell_id")
  
  tcra_j_heatmap <- lattice::levelplot(value~variable*-cell_id, data = tcra_j_tidy, col.regions=vj.color.palette(1000), scales = list(tck = c(0,0), y = list(cex=1, at = seq(from = -1, to = -1 * nrow(empty_frame), by = -1), labels = c(1:nrow(empty_frame))), x = list(alternating = 3, rot = 90)), ylab="Cell ID", xlab = "", colorkey=list(space="top"), at = seq(from = 0, to = 10, by = 0.01), main = list(label = "log2 V(D)J read support"), panel=function(...) { arg <- list(...)
  panel.levelplot(...)
  panel.abline(h = multicell_clade_lims)
  panel.abline(h = subclade_lims, lty = 2)})
  
  pta_TCRA_CDR3_input <- pta_additional_annotation_single_donor[,c("cell_ID","TCRA_CDR3_nt_length")]
  pta_TCRA_CDR3_input$TCRA_CDR3_nt_length[which(pta_additional_annotation_single_donor$productive_TCRA_read_support <= 1)] <- NA
  
  pta_TCRA_CDR3_input$TCRA_CDR3_nt_length[which(pta_TCRA_CDR3_input$TCRA_CDR3_nt_length == "-")] <- NA
  
  pta_TCRA_CDR3_input$TCRA_CDR3_nt_length <- as.numeric(pta_TCRA_CDR3_input$TCRA_CDR3_nt_length)
  
  pta_TCRA_CDR3_input$TCRA_CDR3_nt_length <- pta_TCRA_CDR3_input$TCRA_CDR3_nt_length / 3
  
  colnames(pta_TCRA_CDR3_input) <- c("cell_ID","TCRA_CDR3_length")
  
  pta_TCRA_CDR3 <- reshape2::melt(pta_TCRA_CDR3_input, id.vars = "cell_ID")
  pta_TCRA_CDR3$value[pta_TCRA_CDR3$value == "-"] <- 0
  pta_TCRA_CDR3$value <- as.numeric(pta_TCRA_CDR3$value)
  
  # Create haplotype heatmap
  pta_TCRA_CDR3_heatmap = levelplot(value~variable*-cell_ID, pta_TCRA_CDR3, col.regions=cdr3.color.palette(30), scales = list(tck = c(0,0), y = list(cex=1, at = seq(from = -1, to = -1 * nrow(empty_frame), by = -1), labels = c(1:nrow(empty_frame))), x = list(alternating = 3, rot = 90)), ylab = NULL, xlab="", colorkey=list(space="top"), at = seq(from = 0, to = 29, by = 1), main = list(label = "CDR3 aa length"), 
                                    , panel=function(...) { arg <- list(...)
                                    panel.levelplot(...)
                                    panel.abline(h = multicell_clade_lims)
                                    panel.abline(h = subclade_lims, lty = 2)})
  
  if(nrow(pta_other_gene_muts) > 0 & nrow(pta_CD274_muts) > 0){
    comb_levObj_all <- c(cell_type_VDJ,cell_type_SHM,cell_type_csr,TNFRSF14_vaf_heatmap,CD274_vaf_heatmap,other_gene_heatmap,igh_v_heatmap,igh_j_heatmap,pta_IgH_CDR3_heatmap,tcra_v_heatmap,tcra_j_heatmap,pta_TCRA_CDR3_heatmap, layout = c(12, 1),  merge.legends = FALSE)
    if (runman) { dev.new(width=15, height=20) }
    plot(comb_levObj_all, panel.width = list(c(1,1,1,ncol(pta_TNFRSF14_mut_array) - 1,ncol(pta_CD274_mut_array) - 1,ncol(pta_other_gene_mut_array) - 1,ncol(igh_v_array) - 1,ncol(igh_j_array) - 1,1,ncol(tcra_v_array) - 1,ncol(tcra_j_array) - 1,1),c("null")))
    if (runman) { dev.copy(pdf,paste0("./output/PTA_Full_heatmap_",donors[z],".pdf"),width=16,height=20); dev.off() }
  }else if(nrow(pta_other_gene_muts) == 0 & nrow(pta_CD274_muts) > 0){
      comb_levObj_all <- c(cell_type_VDJ,cell_type_SHM,cell_type_csr,TNFRSF14_vaf_heatmap,CD274_vaf_heatmap,igh_v_heatmap,igh_j_heatmap,pta_IgH_CDR3_heatmap,tcra_v_heatmap,tcra_j_heatmap,pta_TCRA_CDR3_heatmap, layout = c(11, 1),  merge.legends = FALSE)
    if (runman) { dev.new(width=15, height=20) }
    plot(comb_levObj_all, panel.width = list(c(1,1,1,ncol(pta_TNFRSF14_mut_array) - 1,ncol(pta_CD274_mut_array) - 1,ncol(igh_v_array) - 1,ncol(igh_j_array) - 1,1,ncol(tcra_v_array) - 1,ncol(tcra_j_array) - 1,1),c("null")))
    if (runman) { dev.copy(pdf,paste0("./output/PTA_Full_heatmap_",donors[z],".pdf"),width=16,height=20); dev.off() }
  }else if(nrow(pta_other_gene_muts) == 0 & nrow(pta_CD274_muts) == 0){
    comb_levObj_all <- c(cell_type_VDJ,cell_type_SHM,cell_type_csr,TNFRSF14_vaf_heatmap,igh_v_heatmap,igh_j_heatmap,pta_IgH_CDR3_heatmap,tcra_v_heatmap,tcra_j_heatmap,pta_TCRA_CDR3_heatmap, layout = c(10, 1),  merge.legends = FALSE)
    if (runman) { dev.new(width=15, height=20) }
    plot(comb_levObj_all, panel.width = list(c(1,1,1,ncol(pta_TNFRSF14_mut_array) - 1,ncol(igh_v_array) - 1,ncol(igh_j_array) - 1,1,ncol(tcra_v_array) - 1,ncol(tcra_j_array) - 1,1),c("null")))
    if (runman) { dev.copy(pdf,paste0("./output/PTA_Full_heatmap_",donors[z],".pdf"),width=16,height=20); dev.off() }
  }
}  
      
 
 
 21. R studio session information 
  pander(sessionInfo())  
  R version 4.5.0 (2025-04-11)  
  Platform:  aarch64-apple-darwin20 
  locale: 
en_US.UTF-8||en_US.UTF-8||en_US.UTF-8||C||en_US.UTF-8||en_US.UTF-8 
  attached base packages:   stats4 ,
 stats ,  graphics ,  grDevices ,  utils ,
 datasets ,  methods  and  base  
  other attached packages: 
 Rsamtools(v.2.26.0) ,  Biostrings(v.2.78.0) ,
 XVector(v.0.50.0) ,  ggpubr(v.0.6.2) ,
 ggh4x(v.0.3.1) ,  ggtree(v.4.0.4) ,
 ape(v.5.8-1) ,  pander(v.0.6.6) ,  drc(v.3.0-1) ,
 gtools(v.3.9.5) ,  stringi(v.1.8.7) ,
 XML(v.3.99-0.20) ,  ggforce(v.0.5.0) ,
 jsonlite(v.2.0.0) ,  MASS(v.7.3-65) ,
 vcfR(v.1.15.0) ,  knitr(v.1.51) ,
 latticeExtra(v.0.6-31) ,  lattice(v.0.22-6) ,
 RColorBrewer(v.1.1-3) ,  viridis(v.0.6.5) ,
 viridisLite(v.0.4.3) ,  patchwork(v.1.3.2) ,
 scales(v.1.4.0) ,  dndscv(v.0.0.1.0) ,
 lubridate(v.1.9.4) ,  forcats(v.1.0.1) ,
 stringr(v.1.6.0) ,  dplyr(v.1.2.0) ,
 purrr(v.1.2.1) ,  readr(v.2.1.6) ,
 tidyr(v.1.3.2) ,  tibble(v.3.3.1) ,
 ggplot2(v.4.0.2) ,  tidyverse(v.2.0.0) ,
 GenomicRanges(v.1.62.1) ,  Seqinfo(v.1.0.0) ,
 IRanges(v.2.44.0) ,  S4Vectors(v.0.48.0) ,
 BiocGenerics(v.0.56.0) ,  generics(v.0.1.4)  and
 readxl(v.1.4.5)  
  loaded via a namespace (and not attached): 
 rstudioapi(v.0.18.0) ,  magrittr(v.2.0.4) ,
 TH.data(v.1.1-5) ,  farver(v.2.1.2) ,
 rmarkdown(v.2.30) ,  ragg(v.1.5.0) ,
 fs(v.1.6.6) ,  vctrs(v.0.7.2) ,
 rstatix(v.0.7.3) ,  htmltools(v.0.5.9) ,
 plotrix(v.3.8-13) ,  broom(v.1.0.12) ,
 cellranger(v.1.1.0) ,  Formula(v.1.2-5) ,
 gridGraphics(v.0.5-1) ,  sass(v.0.4.10) ,
 bslib(v.0.10.0) ,  htmlwidgets(v.1.6.4) ,
 plyr(v.1.8.9) ,  sandwich(v.3.1-1) ,
 zoo(v.1.8-15) ,  cachem(v.1.1.0) ,
 lifecycle(v.1.0.5) ,  pkgconfig(v.2.0.3) ,
 Matrix(v.1.7-3) ,  R6(v.2.6.1) ,
 fastmap(v.1.2.0) ,  digest(v.0.6.39) ,
 aplot(v.0.2.9) ,  textshaping(v.1.0.4) ,
 vegan(v.2.7-2) ,  labeling(v.0.4.3) ,
 timechange(v.0.4.0) ,  httr(v.1.4.7) ,
 polyclip(v.1.10-7) ,  abind(v.1.4-8) ,
 mgcv(v.1.9-1) ,  compiler(v.4.5.0) ,
 bit64(v.4.6.0-1) ,  fontquiver(v.0.2.1) ,
 withr(v.3.0.2) ,  backports(v.1.5.0) ,
 S7(v.0.2.1) ,  BiocParallel(v.1.44.0) ,
 carData(v.3.0-6) ,  ggsignif(v.0.6.4) ,
 rappdirs(v.0.3.4) ,  permute(v.0.9-8) ,
 tools(v.4.5.0) ,  otel(v.0.2.0) ,  glue(v.1.8.0) ,
 nlme(v.3.1-168) ,  grid(v.4.5.0) ,
 reshape2(v.1.4.5) ,  cluster(v.2.1.8.1) ,
 ade4(v.1.7-23) ,  seqinr(v.4.2-36) ,
 gtable(v.0.3.6) ,  tzdb(v.0.5.0) ,
 pinfsc50(v.1.3.0) ,  hms(v.1.1.4) ,
 car(v.3.1-5) ,  pillar(v.1.11.1) ,
 vroom(v.1.7.0) ,  yulab.utils(v.0.2.4) ,
 splines(v.4.5.0) ,  tweenr(v.2.0.3) ,
 treeio(v.1.34.0) ,  bit(v.4.6.0) ,
 survival(v.3.8-3) ,  deldir(v.2.0-4) ,
 tidyselect(v.1.2.1) ,  fontLiberation(v.0.1.0) ,
 fontBitstreamVera(v.0.1.1) ,  gridExtra(v.2.3) ,
 xfun(v.0.57) ,  UCSC.utils(v.1.6.1) ,
 lazyeval(v.0.2.2) ,  ggfun(v.0.2.0) ,
 yaml(v.2.3.12) ,  evaluate(v.1.0.5) ,
 codetools(v.0.2-20) ,  interp(v.1.1-6) ,
 gdtools(v.0.4.4) ,  ggplotify(v.0.1.3) ,
 cli(v.3.6.5) ,  systemfonts(v.1.3.1) ,
 jquerylib(v.0.1.4) ,  GenomeInfoDb(v.1.46.2) ,
 dichromat(v.2.0-0.1) ,  Rcpp(v.1.1.1) ,
 png(v.0.1-8) ,  parallel(v.4.5.0) ,
 poilog(v.0.4.2.1) ,  jpeg(v.0.1-11) ,
 bitops(v.1.0-9) ,  mvtnorm(v.1.3-3) ,
 tidytree(v.0.4.7) ,  ggiraph(v.0.9.3) ,
 crayon(v.1.5.3) ,  rlang(v.1.1.7)  and
 multcomp(v.1.4-29)  
 


 
 

 

 

 

 

 
 

 

 
 

 
 
